# Establishment and investigation of a surgical model of hypothyroidism in Wistar rats

**DOI:** 10.1371/journal.pone.0340302

**Published:** 2026-01-20

**Authors:** Yue Wang, Jiayu Zhu, Fang Yu, Han Gao, Yuanyuan Fu, Chunyu Chu, Chao Yu, Zhonghui Li, Xiao Liu, Meng Wang, Yuan Liu, Luming Zheng, Qingqing He

**Affiliations:** 1 First Clinical Medical College, Shandong University of Traditional Chinese Medicine, Jinan, China; 2 Department of Thyroid and Breast Surgery, The 960th Hospital of PLA Joint Logistics Support Force, Jinan, Shandong, China; 3 The 960th Hospital of PLA Joint Logistics Support Force, Jinan, Shandong, China; 4 Chinese Medicine College, Shandong University of Traditional Chinese Medicine, Jinan, China; 5 School of Public Health, Shandong First Medical University and Shandong Academy of Medical Sciences, Jinan, Shandong, China; 6 Clinical Medicine College, Shandong Second Medical University, Weifang, China; 7 Department of Obstetrics and Gynecology, The 960th Hospital of PLA Joint Logistics Support Force, Jinan, Shandong, China; 8 Basic Medical Laboratory, The 960th Hospital of PLA Joint Logistics Support Force, Jinan, Shandong, China; Emory University School of Medicine, UNITED STATES OF AMERICA

## Abstract

**Background:**

The purpose of this study was to investigate the surgical model of hypothyroidism in rats and the feasibility and stability of the model.

**Methods:**

Forty-eight adult male Wistar rats were randomly divided into 12 sham-operated rats (control group) and 36 operated rats (18 rats in the group with total thyroidectomy and 18 rats in the group with subtotal thyroidectomy). Thyroid-stimulating hormone (TSH), free thyroxine (FT4), and free triiodothyronine (FT3) serum concentrations were measured prior to surgery and on postoperative days 14, 28 and 90; blood calcium (Ca), blood phosphorus (P), and parathyroid hormone (PTH) concentrations were measured on postoperative day 10; and thyroglobulin (Tg) concentrations were measured on postoperative day 60. The postoperative behavioral status of the rats was comprehensively evaluated on the basis of behavioral indicators.

**Results:**

There was no significant difference in preoperative TSH, FT4, or FT3 between the control and surgical groups (*p* > 0.05). On postoperative day 10, there were no significant differences in Ca, P, or PTH levels among the groups (*p* > 0.05). On postoperative days 14, 28 and 90, the TSH level was greater, whereas the FT4 and FT3 levels were lower in the surgical group (*p* < 0.05). On the 60th postoperative day, the Tg was significantly lower in the surgical group (*p* < 0.05).

**Conclusion:**

Surgical removal of the thyroid gland can be used to successfully establish a hypothyroid rat model that is stable and reliable and can provide a model basis for hypothyroidism-related research.

## Introduction

Hypothyroidism is a relatively common endocrine disease, and the prevalence of hypothyroidism is gradually increasing globally, affecting approximately 10% of the global population [1,2]. In terms of morbidity, the prevalence is greater in women than in men and increases with age [3]. Risk factors include female sex, advanced age, genetic history, and personal history of autoimmune disease [4]. Hypothyroidism, caused primarily by insufficient thyroid hormones (THs) production or inappropriate action in target tissues, can affect virtually all systems and major organs of the body, and its clinical symptoms are associated primarily with a reduced metabolic rate and decreased sympathetic excitability. Hypothyroidism can occur at all ages, with varying degrees of negative impact on the patients’ vital activities, quality of life, and overall well-being.

The common causes of hypothyroidism are autoimmune diseases, thyroid surgery, radioactive iodine therapy [5]. The diversity of the causes of hypothyroidism determines the differences in animal models of hypothyroidism, in which methods that have been applied to animal experimentation include the use of anti-thyroid drugs, surgical partial or total removal of the thyroid gland, radioactive ^131^I treatment, dietary restriction of iodine intake, genetic modification, and immuno-induction. Propylthiouracil (PTU) is often used in rodent hypothyroidism modeling and behavioral abnormality studies [6,7]. However, this method has several shortcomings: first, it is difficult to accurately control the concentration and dose of the drug; second, the modeling process is time-consuming; and third, it is difficult to maintain the hypothyroidism status stably for a long period of time after successful modeling. In addition, hypothyroidism caused by antithyroid drugs is not a common cause of clinical hypothyroidism in patients, which limits its application to some extent, and drug-induced hypothyroidism is relatively rare [6,7]. The optimization and in-depth exploration of surgical models have become the focus and direction of research on rat models.

Surgical modeling has the advantages of stable model and high success rate [8], however, in surgical modeling studies, the surgical process and methods used in rats are rarely described in detail, which makes it difficult to widely apply and popularize the surgical models. In addition, no studies have constructed a hypothyroidism model by removing the thyroid tissue of rats through the electrocoagulation pen hemostat. From the perspective of experimental operations and testing, rats are moderately sized and easy to handle. Their thyroid glands are readily identifiable and removable during surgery. Additionally, rats exhibit complex behaviors, facilitating the observation of preoperative and postoperative changes and the effects of hypothyroidism on behavior. Therefore, male Wistar rats were selected as the research subjects in this study to introduce and explore the surgical modeling method for hypothyroidism in animals in detail, and to provide a basis for later clinical treatment and efficacy assessment.

## Materials and methods

### Experimental animals and breeding conditions

Forty-eight healthy male SPF-grade Wistar rats with body masses of 220−240 g were used in this study, these rats were purchased from Jinan Pengyue Laboratory Animal Breeding Center (License number: SCXK (LU) 2022 0006), and all the rats were kept in the Laboratory Animal Center of the 960th Hospital of the People's Liberation Army (PLA), Laboratory Unit Use License No. SYXK (LU) 2022 0031. This study was approved by the 960th Hospital of the PLA under ethical number (2024) Research Ethics Review No. (102). The rats were housed in an environment that conformed to the standards of 12 h/12 h light, 45%−50% relative humidity, and 22−23°C of ambient temperature, were fed ordinary maintenance feed (License No. SCXK (Jin) 2020-0004) and ordinary pure water, and were allowed to eat and drink freely.

### Main reagents and instruments

Curved forceps, tissue scissors, scissors, ophthalmic forceps, needle holders, sterile skin preparation kits, medical absorbable surgical sutures, and electrocoagulation pen hemostats, which were purchased from Marek Company (220 V, low-grade mode); 1.25% ready-to-use tribromoethanol solution, purchased from Dalian Bogreen Biotechnology Co. Ltd.; 75% povidone-iodine solution, alcohol, sterile gauze, sterile cotton balls; 5.5-gauge scalpel needles, various sizes of syringes; TSH Enzyme-linked immunosorbent assay (ELISA) Kit, Rat Free Thyroid Hormone (FT4) ELISA Kit, Rat Free Triiodothyronine (FT3) ELISA Kit, Rat Parathyroid Hormone (PTH) ELISA Kit, Rat Thyroglobulin (Tg) ELISA Kit, ADS-W-LZ005 Blood Phosphorus Test Kit (Phosphomolybdic Acid Method), ADS-W-D010 Calcium Test Kit (Phosphomolybdic Acid Method), all of the above kits were purchased from Jiangsu Aidisheng Biological Technology Co. Ltd.; 10% formalin solution; centrifuges, multichannel pipette guns and tips, electric thermostat, and enzyme labeling apparatus; 10% formalin solution; centrifuge, multichannel pipette gun and gun head, electric thermostat, enzyme labeler, deionized water; homemade field box, video analysis system software purchased from Shanghai Jiliang Software Technology Co.

### Experimental design and randomization

Prior to surgery, all rats were marked with unique identification ear tags. The allocation sequence was generated by the first author using a computer-based random number generator. To ensure allocation concealment, the sequence assignments were placed in sequentially numbered, opaque sealed envelopes maintained by a laboratory administrator not involved in the study. An experimental assistant, who was not part of the surgical or assessment team, opened the next sequentially numbered envelope only after each animal had been anesthetized and prepared for surgery. The surgical team remained blinded to group assignment until this moment, immediately before the initial incision, when the assistant verbally communicated the group assignment to the lead surgeon. This timing was necessary for surgical safety as the different procedures (sham operation, subtotal thyroidectomy, and total thyroidectomy) required different surgical approaches. A total of 48 rats were successfully randomized following this procedure into three groups: sham operation group (W-Sham, n = 12), total thyroidectomy group (W-SI, n = 18), and subtotal thyroidectomy group (W-SII, n = 18), with no exclusions or reassignments occurring after randomization.

### Surgical procedures

The rats were weighed before anesthesia, and the amount of anesthetic required was calculated at a dose of 25 ml per kilogram body weight (25 mL/kg). The rats were anesthetized by an intraperitoneal injection of tribromoethanol at an appropriate dose using a 10 mL syringe fitted with a 0.7 × 32TWLB needle. The anesthesia status of the rats was closely monitored, and after confirming that the rats had been successfully anesthetized, the rats were fixed on a surgical table using cotton thread, the hair in the surgical area of the neck was carefully trimmed with scissors, and the remaining hair was finely trimmed via a sterile skin preparation tool. A sterile cotton ball was placed on the back of the rat's neck to keep its neck in an overextended state and better expose the surgical field. In accordance with the principles of aseptic surgery for animals, the methods include hand washing and disinfection and wearing sterile gloves, routine disinfection of the operative area via iodine-vapor cotton balls, laying sterile cave towels, and careful observation to ensure airway patency throughout the surgical process.

In the W-Sham group, the rat sternum and trachea were used as anatomical landmarks, and a median longitudinal neck incision (approximately 3 cm) was made between the sternal notch and the mandible. The skin, subcutaneous tissue and mandibular glands were incised layer by layer, and the cervical vastus and anterior cervical muscle groups were bluntly separated. After a round needle was passed through to separate the anterior cervical musculature, an electrocoagulation pen hemostat was used to stop the bleeding, which was retracted and fixed to fully expose the thyroid gland. After confirming the morphological integrity of the thyroid gland, the muscle layer and subcutaneous tissues were closed continuously in layers with 4-0 absorbable sutures, the mandibular glands were reset and then closed intradermally, and the operative area was disinfected with iodine-vapor cotton balls. Throughout the procedure, to avoid thermal injury from the electrocoagulation pen hemostat, its power was adjusted to the minimum level required for selective thyroid tissue cutting (voltage: 220 V; duration per use: 2 s; cut mode), while surrounding structures were protected with saline-moistened sterile cotton balls.

In the W-SII group, after the thyroid gland was exposed via the above methods: 1) In the left lobe treatment, the left anterior cervical muscles were retracted laterally. The true capsule of the thyroid was meticulously dissected via fine ophthalmic dissecting forceps. The superior and inferior pole vessels were coagulated and divided via an electrocoagulation pen hemostat. For the superior thyroid vascular pedicle, a microdissection technique was applied immediately adjacent to the glandular capsule; individual superior pole vascular branches were identified and ligated. The inferior pole vessels were managed distal to the thyroid capsule. Meticulous care was taken throughout the dissection to avoid injury to the recurrent laryngeal nerve (RLN) and the parathyroid glands. 2) Isthmus treatment: The connective tissue between the thyroid isthmus and the trachea was bluntly separated via ophthalmic microforceps, followed by complete resection of the isthmus (noting that this structure was absent in some rats). 3) Right lobe treatment: Following exposure of the right lobe via an identical approach, the posterior capsular area surrounding the Berry ligament attachment was preserved. Selective distal nutrient vascular branches within this preserved tissue were retained to maintain its micro-circulatory perfusion.

In the W-SI group, on the basis of complete resection of the left lobe and isthmus, total resection of the right lobe was added. The right lobe was completely removed according to the above surgical methods, with special attention given to the protection of the RLN and parathyroid gland. After complete resection, we ensured that there was no residual gland, and the rest of the treatments were the same as those used in the subtotal thyroidectomy group.

### Postoperative care

**Fig 1:**
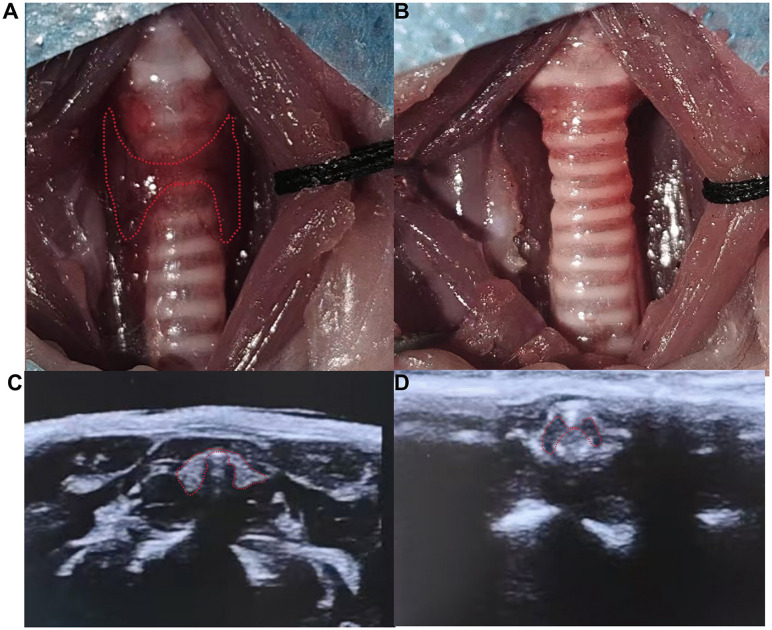
Preoperative and postoperative thyroid visual field exposure and ultrasound exploration. **(A)** Intraoperative exposure of thyroid tissue. **(B)** Visual field performance after total thyroidectomy. **(C)** Preoperative thyroid ultrasound imaging. **(D)** Ultrasound imaging after total thyroidectomy.

After the completion of surgery (Fig 1; S1 Fig), warming measures were applied to each experimental animal, and the animals that had not yet awakened were placed individually and closely observed until they awakened; then, they were placed in separate cages. After surgery, normal chow and pure water were provided to the rats in the W-Sham group, whereas water containing 0.9% calcium gluconate was provided to the rats in the W-SI and W-SII groups. To alleviate postoperative pain, all surgical animals received ibuprofen (20 mg/kg, administered by drinking water) immediately after surgery and at 24 h and 48 h postoperatively. The same analgesic regimen was synchronously implemented in W-Sham group animals [9,10].

### Tissue sample collection and processing

Two hours later, the weight of the removed thyroid tissue was recorded. In the surgical group, 6 rat thyroid tissues were randomly selected for immersion in 10% formalin solution for Hematoxylin and eosin (H&E) staining verification.

### Blood collection methods and steps

**Fig 2:**
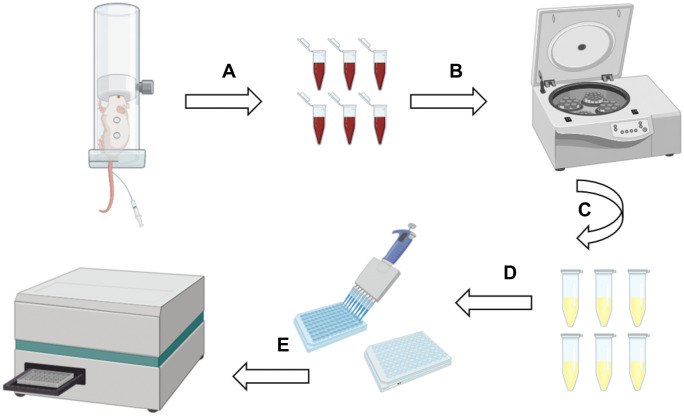
Flowchart of blood sample collection, serum isolation and testing. **(A)** Tail vein blood collection. **(B, C)** Blood was centrifuged to obtain the serum. **(D, E)** The optical density (OD) in the enzyme-linked immunosorbent assay (ELISA) was measured using a microplate reader.

The rats were secured in a fixator with the tail exposed. The tail vein was identified and disinfected with alcohol, followed by evaporation. A 5.5 scalpel needle was inserted parallel to the tail, and needle placement was confirmed by palpation before connecting to a 5 ml syringe. Blood was collected under steady negative pressure. Hemostasis was achieved using sterile gauze pressure, and the blood was promptly transferred to a centrifuge tube. After standing for 2 hours, samples were centrifuged to isolate serum, which was stored at –20 °C (Fig 2). Throughout the procedure, rats were closely monitored for reactions and vital signs, and kept warm and restrained to minimize tail tension and stress in compliance with animal welfare guidelines.

### Explanation of changes in blood sample size

In the W-SII group, two rats died, leading to a reduced effective sample size at days 14 and 90 post-operation. Serum levels of Ca, P, and PTH were assessed 10 days after surgery, while thyroid function and related parameters were evaluated at mid-term time points (28 and 60 days). Statistical analysis was performed on serum samples randomly selected from 8 control and 10 surgical animals. The sample size was determined through a combination of a priori power analysis, which indicated a large effect size (Cohen's f = 0.65), and practical constraints related to animal health and strict ethical guidelines [11]. Systemic hypothyroidism markedly reduces tail vein blood flow in rats, making sufficient blood collection difficult and often necessitating repeated punctures. In adherence to the 3R principles (Refinement-Optimization) [12] and to minimize stress or injury from repeated procedures that could confound serum measurements, the final sample size was prudently limited. This approach ensured both ethical compliance and scientific reliability.

### Observation indices

Throughout the study, body weight changes were closely monitored, and hair growth, food and water intake, as well as behavioral activity were regularly observed. To systematically evaluate thyroid function and related parameters, multiple biochemical assays were performed at designated time points: Serum levels of TSH, FT4, and FT3 were measured preoperatively and on postoperative day 14 to confirm the success of hypothyroidism modeling. On day 10 post-operation, PTH, Ca, and P were assessed in randomly selected rats from each group. Subsequent measurements of TSH, FT4, and FT3 were conducted on days 28 and 90 post-surgery. Additionally, Tg levels were evaluated on day 60 to assess residual thyroid tissue and regenerative repair.

### Behavioral tests

(1) The Open Field Test (OFT) was conducted in a brown wooden square arena (100 cm × 100 cm × 40 cm). Prior to testing, the rats were acclimatized to the experimental room for 30 minutes. During formal testing, individual rats were placed in the central zone of the arena and allowed to explore freely for 5 minutes. Their movement trajectories were recorded throughout the session using an via camera connected to an animal behavior analysis system (Shanghai Jiliang Software Technology Co., Ltd.). The arena was thoroughly cleaned with 75% ethanol between trials to eliminate olfactory cues. All video recordings were analyzed by researchers blinded to experimental groups [13].
(2) The Elevated Plus Maze Test (EPMT) consists of four arms: two open arms and two enclosed arms. The maze comprises two open arms (50 × 10 × 0.6 cm with edges) and two enclosed arms (50 × 10 × 30 cm with walls), featuring nonreflective surfaces. These arms extend from a central platform (10 × 10 cm) positioned 50 cm above ground. After adapting to the environment for one minute, the rats were allowed to freely explore the maze under natural light for five minutes. The maze was recorded via camera and analyzed via software. Following each animal's trial, the maze was thoroughly cleaned with 75% ethanol to eliminate odor residues and prevent interference in subsequent experiments [14].
(3) The Forced Swimming Test (FST) used a transparent cylindrical apparatus (20 cm in diameter, 50 cm in height) filled with 27°C water at a depth of 35 cm. Fresh water was administered to each rat before testing to eliminate residual odor interference. During formal trials, the rats were placed alone in the water and forced to swim continuously for 6 minutes. The study focused on recording the immobility time (i.e., cessation of struggling and maintenance of the head-levitated floating state) during the final 4 minutes. Longer durations typically indicate more pronounced depressive-like behaviors. The entire experiment was conducted in a quiet, uniformly lit environment to minimize external disturbance [15,16].

### H&E staining

Histological staining with hematoxylin and eosin was performed according to standard protocols. Briefly, paraffin-embedded tissue sections were deparaffinized in xylene and rehydrated through a graded ethanol series (100%, 95%, 85%, and 70%). The nuclei were stained with hematoxylin for 5–15 minutes, followed by rinsing under running tap water. Differentiation was carried out in 1% acid ethanol for several seconds, and sections were then blued in warm water or a weak alkaline solution. After additional washing in running water, cytoplasmic staining was performed using eosin for 1–3 minutes. Subsequently, the sections were dehydrated through a graded ethanol series, cleared in xylene, and finally mounted with neutral balsam. This staining method clearly highlights nuclei in blue-purple and cytoplasm/extracellular matrix in pink, enabling detailed morphological analysis [17].

### Statistical methods

Data analysis was performed via SPSS 22.0. Data were expressed as mean±standard deviation (SD) or median (interquartile range, IQR). For weight, serological indices, and behavioral metrics, normality was first assessed via the Shapiro-Wilk test. When all groups satisfied normality, homogeneity of variance was evaluated via Levene’s test. If homogeneity was confirmed, one-way ANOVA was employed to examine intergroup differences; significant results (*p* < 0.05) were subjected to post hoc pairwise comparisons via Tukey’s HSD (equal sample sizes) or Tukey-Kramer (unequal sample sizes). For normally distributed data with heterogeneous variance, Welch’s ANOVA was used to test overall group differences, with significant outcomes followed by Games-Howell post hoc analysis. When normality was violated in ≥1 group, the Kruskal-Wallis test was used to assess group differences; where significant (*p* < 0.05), Dunn's test with Bonferroni correction was applied for pairwise comparisons to control Type I error inflation, reporting adjusted *p*-values. However, for the purpose of consistent presentation and interpretation across Behavioral Tests (OFT, EPMT), data from these specific tests are uniformly presented as median with interquartile range and analyzed using the Kruskal-Wallis test, regardless of the normality test outcome for individual datasets. All analyses adopted α = 0.05 as the significance threshold. Graphical representations and associated statistics were generated via GraphPad Prism 10.1.2 (GraphPad Software).

## Results

### Changes in body weight

**Fig 3:**
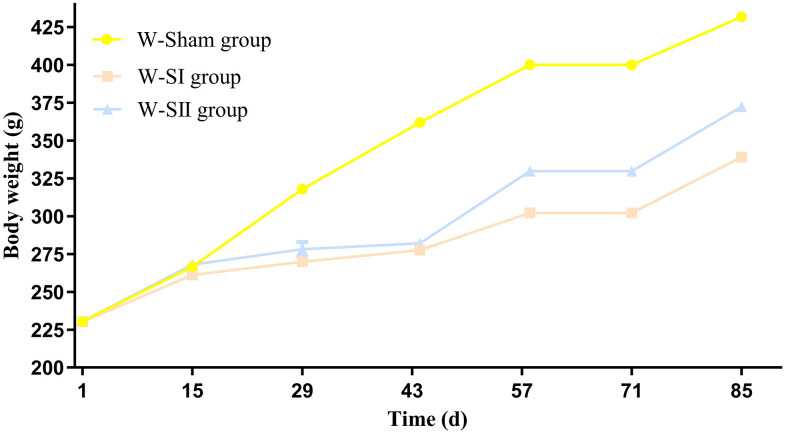
Line graph of body weight changes in the rats. Day 1 represents the preoperative baseline weight. Postoperative time points are indicated at days 15 (14 days post-op), 29, 43, 57, 71, and 85.

Significant time-dependent differences in body weight changes were observed among the W-Sham, W-SI, and W-SII groups (Fig 3). No significant intergroup differences were detected on postoperative days 1 and 15 by one-way ANOVA (Day 1: F(2, 45) = 0.024, *p* = 0.977; Day 15: F(2, 35) = 0.781, *p* = 0.464). However, by day 29, a significant overall difference emerged (one-way ANOVA, F(2, 43) = 5.84, *p* = 0.006). Post-hoc analysis revealed that both the W-SI group (*p* = 0.005) and the W-SII group (*p* = 0.029) showed markedly lower body weights than the W-Sham controls did. The difference was further amplified by day 43 (*p* < 0.001) and remained statistically significant on days 57, 71, and 85. At this terminal time point, the W-SI group presented the most pronounced weight loss compared with the W-Sham group (*p* < 0.001), followed by the W-SII group (*p* < 0.001), and a significant difference was also confirmed between the two surgical groups (*p* < 0.001). Overall, the W-Sham group presented 102.73% weight gain (+236.67 g), which was significantly greater than those of the W-SI (+62.88%; + 144.69 g) and W-SII (+78.53%; + 180.96 g) groups.

### Serological test results and analysis

**Fig 4:**
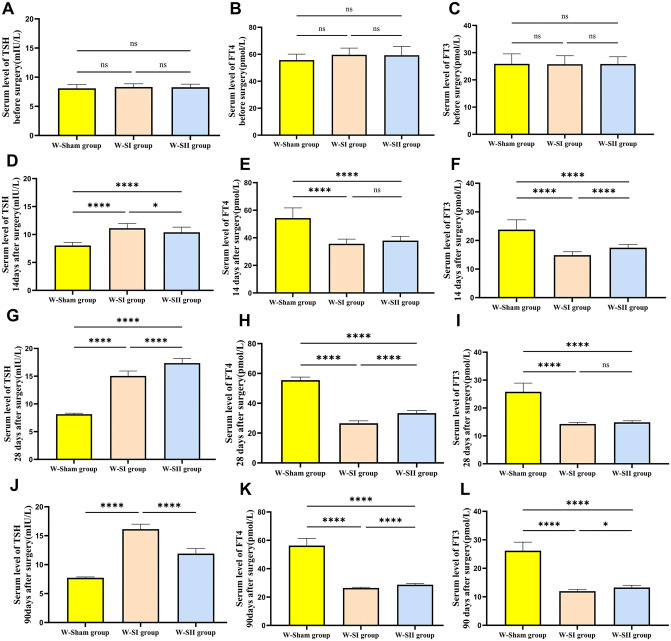
Serum thyroid hormone levels at postoperative time points. **(A, B, C)** Preoperative levels of TSH, FT4, and FT3. **(D, E, F)** Levels at 14 days post-surgery. **(G, H, I)** Levels at 28 days post-surgery. **(J, K, L)** Levels at 90 days post-op. Data are presented as mean±SD. Statistical analysis was performed by one-way ANOVA (panels A, C, D, E, F, G, H, I, J) or non-parametric test with Bonferroni correction (panels B, K, **L)**. By “ns” represents *p-*value greater than 0.05;^**^p-value between 0.001 and 0.01; ^****^p-value < 0.0001.

**Fig 5:**
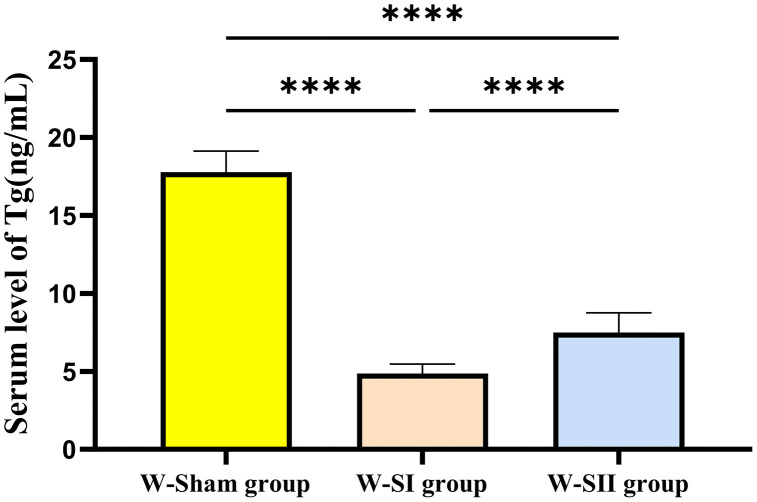
Serum thyroglobulin (Tg) concentrations at 60 days post-surgery. The sham operation group (W-Sham group) (n = 8), the total thyroidectomy group (W-SⅠ group) (n = 10), and the subtotal thyroidectomy group (W-SⅡ group) (n = 10) were used. Each bar represents the mean±SD of each group (one-way ANOVA, ^****^p-value < 0.0001).

The serum concentrations of TSH, FT4 and FT3 were measured via blood collection from the tail vein on the 1st day before and 14th day after the operation. On the 1st day before the operation, there was no statistically significant difference between the rats in each group (*p* > 0.05), and on the 14th day after the operation, the TSH levels in the W-SI and W-SII groups were greater than those in the W-Sham group, and the levels of FT4 and FT3 were significantly lower than those in the W-Sham group. There was a significant difference (*p* < 0.05), with reference to the modeling success criteria of H. Chunping (serum TSH levels were significantly increased and FT4 levels were significantly decreased in the model group compared with the control group, both *p* < 0.05) [18], and the model was considered successful. To verify the stability of the model, a random sample was taken to test the TSH, FT4 and FT3 indices of the rats on the 28th and 90th days after the operation. Graphical and tabular representations were used to show that TSH, FT4, and FT3 concentrations at different times (Fig 4; Table 1). The Tg test was carried out on the 60th day after the operation, and the Tg levels of the W-SI and W-SII groups were significantly lower than those of the W-Sham group (*p* < 0.05) (Fig 5).

**Table 1:** Serum TSH, FT4 and FT3 concentrations.

Group	TSH(mIU/L)	FT4(pmol/L)	FT3(pmol/L)
Before surgery			
W-Sham group (n = 12)	8.08 ± 0.65	55.69 ± 4.42	25.90 ± 3.65
W-SI group (n = 18)	8.32 ± 0.54	59.65 ± 4.95	25.76 ± 3.12
W-SⅡ group (n = 18)	8.29 ± 0.52	59.32 ± 6.51	25.85 ± 2.66
*p-value*	*p*_a_ = 0.565; *p*_b_ = 0.617	*p*_a_ = 0.177; *p*_b_ = 0.249	*p*_a_ = 0.991; *p*_b_ = 0.999
14 days after surgery			
W-Sham group (n = 12)	8.03 ± 0.56	54.29 ± 7.40	23.77 ± 3.45
W-SI group (n = 18)	11.13 ± 0.86	35.66 ± 3.78	14.87 ± 1.74
W-SⅡ group (n = 16)	10.38 ± 0.93	37.95 ± 3.12	17.47 ± 1.06
*p-value*	*p*_a_ = 0.000; *p*_b_ = 0.000	*p*_a_ = 0.000; *p*_b_ = 0.000	*p*_a_ = 0.000; *p*_b_ = 0.000
28 days after surgery			
W-Sham group (n = 8)	8.15 ± 0.18	55.50 ± 2.09	25.81 ± 3.13
W-SI group (n = 10)	14.87 ± 0.94	26.58 ± 1.61	14.26 ± 0.57
W-SⅡ group (n = 10)	12.72 ± 0.68	33.40 ± 1.66	14.86 ± 0.57
*p-value*	*p*_a_ = 0.000; *p*_b_ = 0.000	*p*_a_ = 0.000; *p*_b_ = 0.000	*p*_a_ = 0.000; *p*_b_ = 0.000
90 days after surgery			
W-Sham group (n = 12)	7.74 ± 0.14	56.33 ± 5.07	25.72 ± 2.99
W-SI group (n = 18)	16.14 ± 0.86	26.43 ± 0.54	11.66 ± 0.61
W-SⅡ group (n = 16)	11.90 ± 0.87	28.69 ± 0.96	12.93 ± 0.76
*p-value*	*p*_a_ = 0.000; *p*_b_ = 0.000	*p*_a_ = 0.000; *p*_b_ = 0.000	*p*_a_ = 0.000; *p*_b_ = 0.000

Note: Thyroid-stimulating hormone (TSH), free thyroxine (FT4), free triiodothyronine (FT3). The sham operation group (W-Sham group), the total thyroidectomy group (W-SⅠ group), and the subtotal thyroidectomy group (W-SⅡ group). The notation *p*_a_ is defined as the *p*-value from the comparison of the W-SⅠ group with the W-Sham control group, and *p*_b_ as the *p*-value from the comparison of the W-SⅡ group with the W-Sham control group, respectively. Data were presented as mean±SD.

**Fig 6:**
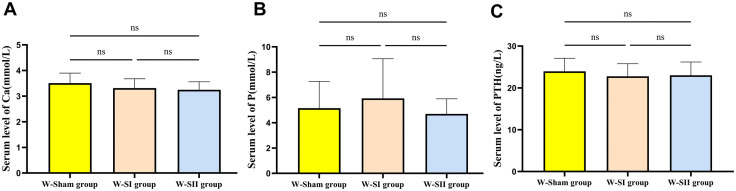
Serum level of Calcium (Ca), phosphorus (P) and parathyroid hormone (PTH). The sham operation group (W-Sham group) (n = 8), the total thyroidectomy group (W-SⅠ group) (n = 10), and the subtotal thyroidectomy group (W-SⅡ group) (n = 10). Each bar represents the mean±SD of each group (Ca, P by one-way ANOVA; PTH by non-parametric test. By “ns” represents *p* > 0.05).

The test data at 10 days after surgery showed that, while the mean/median values of blood Ca and PTH levels in the W-SI and W-SII groups were numerically lower than those in the W-Sham group (Fig 6; Table 2), this difference did not reach statistical significance. Serum Ca levels demonstrated normality (*p* > 0.200) and homogeneity of variances (F(2,25) = 0.276, *p* = 0.761). One-way ANOVA indicated no significant intergroup differences (F (2,25) = 1.261, *p* = 0.301, Fig 6A). Serum P data adhered to normality (*p* > 0.200) and variance homogeneity (F (2,25) = 2.372, *p* = 0.114). One-way ANOVA indicated no significant intergroup differences (F (2,25) = 0.721, *p* = 0.496, Fig 6B). For PTH, the W-SI group violated normality assumptions (Shapiro-Wilk test, *p* = 0.047), necessitating Kruskal-Wallis test which showed no significant intergroup variation (*p* = 0.438, Fig 6C). Collectively, no significant differences were observed in serum levels of Ca, P, or PTH among the surgical and control groups.

**Table 2:** Serum Ca, P and PTH 10 days after surgery.

Group	Ca (mmol/L)	P (mmol/L)	PTH (ng/L)
W-Sham group	3.51 ± 0.39	5.16 ± 2.11	23.96 ± 3.15
W-SI group	3.31 ± 0.36	5.92 ± 3.13	22.77 ± 3.02
W-SⅡ group	3.25 ± 0.31	4.67 ± 1.19	23.02 ± 3.17

Note: Calcium (Ca); phosphorus (P); parathyroid hormone (PTH). The sham operation group (W-Sham group), the total thyroidectomy group (W-SⅠ group), the subtotal thyroidectomy group (W-SⅡ group). ns represents *p* > 0.05. Data were presented as mean±SD.

### Linear correlation between weight of thyroidectomy and thyroid function

**Fig 7:**
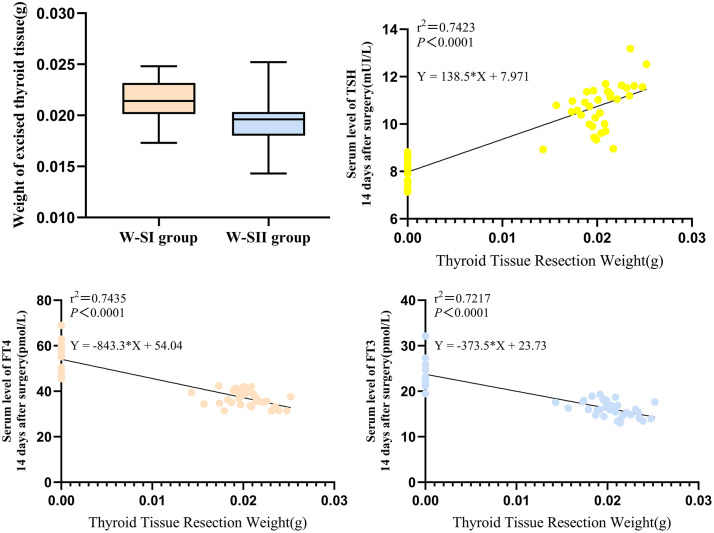
Linear correlation between weight of thyroidectomy and thyroid function. Linear relationship between weight of thyroidectomy and Thyroid-stimulating hormone (TSH), free thyroxine (FT4), free triiodothyronine (FT3).

Quantitative analysis demonstrated statistically significant differences in thyroid tissue weight between the two surgical approaches (Fig 7). The W-SI group showed a 11.2% higher removal weight (0.0214 ± 0.0017 g, n = 18) compared to the W-SII group (0.0193 ± 0.0019 g, n = 16) (*p* = 0.007, non-paired t-test), with an inter-group difference of −0.0022 g (95% CI: −0.0037 to-0.0006) and an effect size η^2^ = 0.206. Analysis of thyroid tissue weight and thyroid function revealed: increased resected weight significantly elevated TSH (r^2^ = 0.7423), while dose-dependent inhibition of FT4 (r^2^ = 0.7435) and FT3 (r^2^ = 0.7217) was observed. Notably, the rate of FT4 reduction (−843.3 pmol/L·g ⁻ ^2^) was 2.24 times that of FT3 (−373.5 pmol/L·g ⁻^1^), indicating FT4 as a more sensitive biomarker for hypothyroidism.The extent of surgical resection was an important cause of the difference in thyroid function among different groups of rats.

### Results of behavioral tests

**Fig 8:**
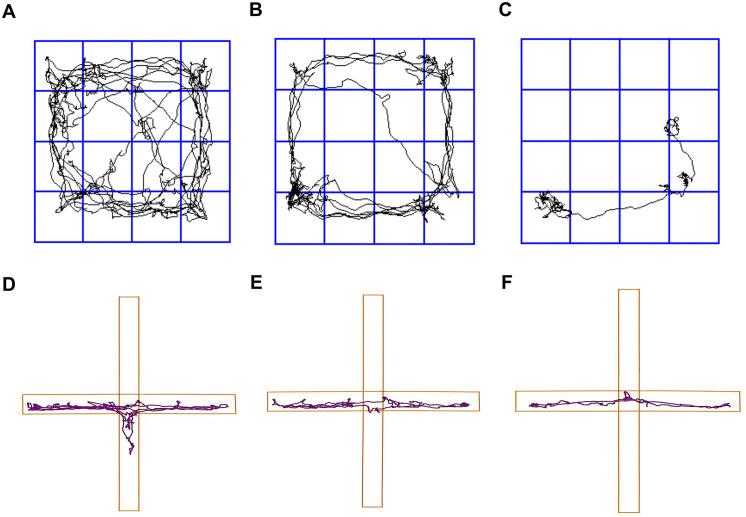
Roadmap of some of the rats’ Open Field Test (OFT) and Elevated Plus Maze Test (EPMT) trajectories. **(A, D)** The sham operation group (W-Sham group). (B, E) the total thyroidectomy group (W-SⅠ group). (C, F) the subtotal thyroidectomy group (W-SⅡ group).

**Fig 9:**
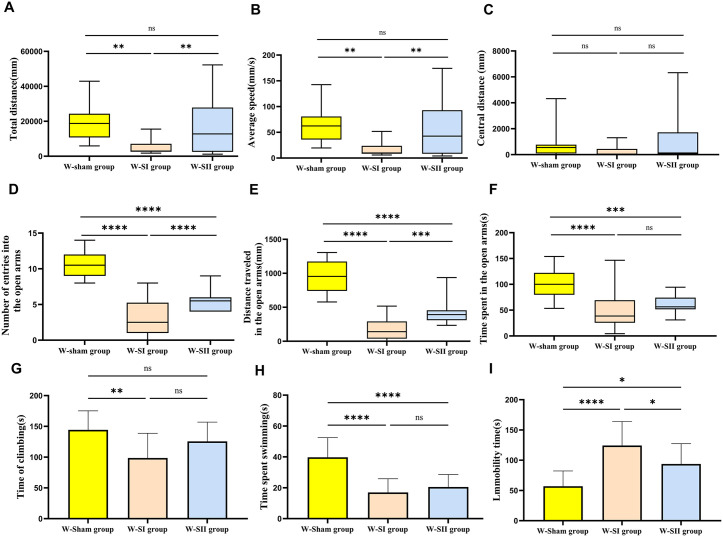
Results of behavioral tests. The sham operation group (W-Sham group) (n = 12), the total thyroidectomy group (W-SⅠ group) (n = 18), the subtotal thyroidectomy group (W-SⅡ group) (n = 16). Open Field Test (OFT) and Elevated Plus Maze Test (EPMT) data were presented as box plots depicting the median (central line) and interquartile range (IQR; bounds of the box) (by non-parametric test and were corrected by Bonferroni). Forced Swimming Test (FST) data were presented as mean±SD (by one-way ANOVA). By “ns” represents *p-*value greater than 0.05; ^*^p-value between 0.01 and 0.05; ^**^p-value between 0.001 and 0.01; ^***^p-value between 0.0001 and 0.001; ^****^p-value < 0.0001).

Postoperatively, we conducted behavioral assessments using the OFT, EPMT, and FST. The movement trajectory diagrams (Fig 8) and corresponding test data results (Fig 9; Table 3) were obtained through a data analysis system. Results from OFT and EPMT were presented using median values with interquartile range, while FST-related outcomes were expressed as mean±SD. In the OFT, the W-Sham group exhibited a significantly greater total distance traveled and higher average speed compared to the W-SI group (*p* = 0.005). The W-SI group also demonstrated reduced locomotor activity relative to the W-SⅡ group (*p* = 0.006). No significant differences were observed between the W-Sham and W-SⅡ groups. Center distance did not differ among groups, suggesting comparable exploratory behavior. In the EPMT, the W-Sham group spent significantly more time in, traveled a greater distance in, and entered more frequently the open arms than both the W-SI group (*p* < 0.001) and the W-SⅡ group (*p* ≤ 0.003). The W-SⅡ group also showed increased open-arm distance and entries compared to the W-SI group (*p* = 0.002 and *p* = 0.003, respectively), indicating lower anxiety-like behavior in the W-Sham group. In the FST, the W-Sham group displayed significantly longer struggling and swimming times (*p* < 0.003) and shorter immobility time than both the W-SI (*p* < 0.001) and W-SⅡ groups (*p* = 0.020). The W-SI group also exhibited significantly longer immobility than the W-SⅡ group (*p* = 0.035), reflecting more pronounced despair-like behavior. In conclusion, the W-Sham group demonstrated the highest levels of active stress-coping and the least anxiety-like behavior, whereas the W-SI group showed the highest passive coping and greatest behavioral despair. The W-SⅡ group presented an intermediate behavioral phenotype across tests.

**Table 3:** Key behavioral parameters assessed in the Open Field Test (OFT) , Elevated Plus Maze Test (EPMT) and Forced Swimming Test (FST).

Project indicators	Group		
	W-Sham group (n = 12)	W-SI group (n = 18)	W-SⅡ group (n = 16)
Total distance(mm)	18763.65 (10798.073-24323.81)	2982.08 (2553.64-7121.57)	12788.86 (2547.88-27896.62)
Average speed(mm/s)	62.34 (35.96-80.81)	9.94 (8.49-23.74)	42.63 (8.49-92.99)
Central distance (mm)	550.63 (92.46-769.63)	9.74 (0-442.29)	130.48 (47.97-1726.04)
Number of entries into the open arms	10.50 (12.00-9.00)	2.50 (5.25-1.00)	5.50 (6.00-4.00)
Distance traveled in the open arms(mm)	954.57 (1173.43-740.36)	140.59 (292.46-37.92)	392.49 (455.86-309.04)
Time spent in the open arms(s)	100.04 (122.22-79.68)	38.52 (69.32-25.58)	56.44 (74.26-51.55)
Time of climbing(s)	144.41 ± 30.79	98.70 ± 40.04	125.65 ± 31.31
Time spent swimming(s)	39.75 ± 12.74	16.98 ± 8.86	20.51 ± 8.04
Immobility time(s)	56.93 ± 25.25	124.31 ± 39.71	93.82 ± 33.56

Note: The sham operation group (W-Sham group), the total thyroidectomy group (W-SⅠ group), the subtotal thyroidectomy group (W-SⅡ group). Results from the OFT and EPMT were expressed as median and interquartile range (IQR), FST were expressed as mean±SD. By “ns” represents *p-*value greater than 0.05; ^*^p-value between 0.01 and 0.05; ^**^p-value between 0.001 and 0.01; ^***^p-value between 0.0001 and 0.001; ^****^p-value < 0.0001.

### H&E staining results

**Fig 10:**
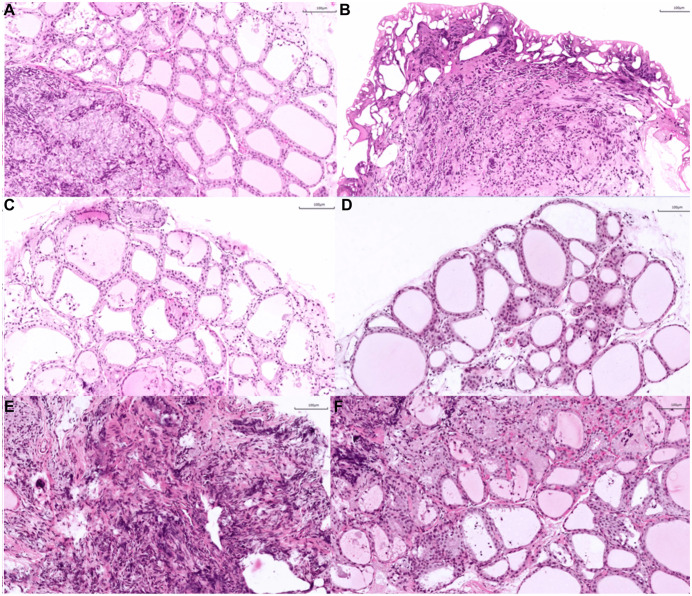
Hematoxylin and eosin (H&E) staining picture. All images were captured at 20 × magnification. **(A)** Normal morphology of thyroid follicular epithelial cells. **(B, C)** Clear visualization of the thyroid capsule. **(D)** The thyroid isthmus is presented. **(E, F)** Altered thyroid tissue morphology following cauterization. Scale bar = 100 um. Samples A, B, and C were derived from the W-SI group, while samples D, E, and F were derived from the W-SII group.

Histological examination with H&E staining of the thyroid tissue resected from the rats revealed round follicles containing abundant colloid, with capillaries and scattered parafollicular cells visible between the follicles—consistent with typical thyroid morphology. An intact capsule was observed under the microscope. Focal electrocautery-induced alterations were also noted in some areas, characterized by loss of normal architectural features. In addition, a small amount of parathyroid tissue was identified adjacent to the main thyroid structure (Fig 10) [17].

### Postoperative survival of animals and analysis

During the experimental observation period, no anesthetic or postoperative complication-related deaths occurred in the W-Sham and W-SI groups of rats during the intraoperative period or at 90 days postoperatively, with an overall survival rate of 100%. However, 2 rats in the W-SII group died. One of these rats died intraoperatively; the other died early in the morning of postoperative day 1 and showed marked swelling of the neck. Thus, the mortality rate in the W-Sham and W-SI groups was 0, whereas the mortality rate in the W-SII group was 11.1%. At 7 days after the operation, the rats in the W-SI and W-SII groups showed a decrease in diet and water intake as well as defecation compared with the preoperative period, as well as slow hair growth, slow movement and reduced activity, but these conditions were gradually recovered after the operation. During the whole observation process, no infection occurred in all animals, and the surgical incision healed well. It is worth noting that all experimental animals showed no signs of surgical site infection, and the incision healing process was as expected.

## Discussion

Hypothyroidism is classified by lesion location into primary, central, and peripheral forms [19–22], with diverse etiologies including, Hashimoto’s thyroiditis, pharmacological agents, thyroidectomy, post-radioiodine (^131^I) therapy, pituitary or hypothalamic surgery, and rarely consumptive hypothyroidism secondary to gastrointestinal stromal tumors [19]; interactions between pharmaceuticals, environmental pollutants, industrial chemicals, and thyroid function may also contribute [21]. Pathologically, impaired THs synthesis and secretion reduce systemic metabolic activity, manifesting clinically as a diminished metabolic rate and sympathetic tone, as a reduced basal metabolic rate, bradycardia, hypothermia, weight gain, fatigue, lethargy, cognitive impairment, and depression [21]. Untreated disease significantly elevates morbidity, mortality [3], and cardiovascular events, whereas therapeutic intervention substantially reduces myocardial infarction, stroke, heart failure, cardiovascular mortality [23,24], and overall mortality. As a chronic condition requiring lifelong management [3], hypothyroidism underscores the critical need for stable rodent models to elucidate its pathophysiology and advance therapeutics.

Various methods have been developed to establish animal models of hypothyroidism, each with distinct advantages and limitations. Surgical thyroidectomy accurately replicates clinical hypothyroidism with high stability, though it requires advanced technical skills and carries risks related to anesthesia and invasive procedures [25]. Pharmacological approaches using antithyroid drugs such as MMI or PTU are convenient and cost-effective, but their effects are often reversible and may involve off-target organ toxicity [26]. Low-iodine diet models are useful for studying naturally occurring hypothyroidism, particularly in neurodevelopmental contexts, but require extended periods to induce hypothyroidism and exhibit considerable individual variability [27]. Additionally, with widespread iodized salt use, the incidence of clinical and subclinical hypothyroidism caused by iodine deficiency has significantly decreased [28]. Radioiodine ablation and genetic models offer high specificity but are associated with technical complexity, high cost, and limited applicability [29,30]. The choice of modeling strategy should be aligned with specific research objectives and practical constraints (summarized in Table 4).

**Table 4:** Advantages and disadvantages of different modeling methods and literature review.

Modeling methods	Method or mechanism	Advantages	Disadvantages	Scope of application
Surgical modeling	Electrocautery damage to thyroid tissue [31], ligation of blood vessels [8], pituitary gland removal [32] or surgical removal of thyroid gland [33]	Stable and long-lasting model, accurate simulation of pathology	Survival can be compromised by high anesthesia risk, difficult operation, traumatic surgery, and longer postoperative recovery time	A study of a model for long-term stabilization of hypothyroidism
Drug modeling	Antithyroid drugs: MMI [34]/PTU [26,35,36]	Simple operation and low cost	Wide variation in drug concentrations, reversible symptoms after discontinuation, may affect other organ functions, difficult to maintain hypothyroidism in the long term	Short-term hypothyroidism mechanism study or drug intervention trial
Modeling of an iodine deficiency diet	Low iodine feed feeding [27]	simulation of the natural pathogenesis of iodine deficiency	Long molding period and individual differences, difficult to accurately control the degree of hypothyroidism, need to be combined with other methods, the scope of application is narrower	Studies on the effects of hypothyroidism on the central nervous system
Radiological modeling	Radioactive ^131^I treatment [29]	Precise destruction of thyroid cells	Only for radioactive iodine related research, complicated operation, requires specialized personnel to operate	Simulation of the effect of radioiodine therapy on thyroid function
Genetic modelling	Gene editing techniques , e.g., Tshr mutation [34], Cntn6 knockout [37]	Targeting specific genes for editing or knockout with clear mechanisms	High technical difficulty, high cost, long experimental period	Study on the mechanism of congenital hypothyroidism or specific gene dysfunction

Note: Comparison of five common methodologies for establishing hypothyroidism models in research, detailing their operational mechanisms, key benefits, limitations, and recommended applications. MMI, methimazole; PTU, propylthiouracil; ^131^I, radioactive iodine-131.

The successful establishment of this animal model relies heavily on specialized anatomical knowledge and sophisticated surgical techniques to ensure smooth operation completion and minimize damage to the rat RLN, parathyroid glands, trachea, and esophagus [38]. This experiment employed an electrocoagulation pen hemostat for cutting or cauterization procedures. Compared with traditional suture ligation of blood vessels, this method is safer and more reliable. Not only does it effectively control bleeding and maintain clear surgical vision, but it also significantly reduces the risk of postoperative hemorrhagic asphyxia caused by intense neck movements and mortality hazards from accidental RLN ligation, leading to respiratory failure. Xie et al. [39] established a hypothyroidism model by ligating thyroid arteries and veins followed by dissection. Seven animals died intraoperatively; 7 days and 28 days post-surgery were 85% and 80%, respectively. All deaths involved hyperpnea preceded by wheezing, attributed to accidental RLN ligation or traction during surgery [39]. Compared with other experimental methods, our approach markedly improved model animal survival rates (94.4% in the surgical group).

Surgical technique critically influences the stability and accuracy of thyroid dysfunction models. Precise resection margins are essential, and the use of an electrocoagulation pen hemostat is vital for hemorrhage control and visual clarity, directly affecting resection accuracy. Modeling subclinical hypothyroidism requires technically challenging partial resections, where subtle differences in resection extent determine whether complete or subclinical hypothyroidism is achieved, significantly impacting experimental comparability. Additionally, surgical approach affects complication rates and animal welfare. Protection of the RLN and parathyroid glands is crucial, and while energy devices aid hemostasis, improper use may increase tissue injury [40]. Variations in surgical skills not only directly affect the accuracy and stability of model establishment but also ultimately affect the welfare level of experimental animals and the overall reliability of research data through their significant impact on postoperative complication rates and animal survival status [40].

During the experiment, one rat died due to anesthesia complications during surgery, and another succumbed on postoperative day 1 from tracheal compression caused by a neck hematoma resulting from postoperative hemorrhage. It is speculated that the cause of death was hemorrhage induced by excessive postoperative activity, and given the confined space of the neck, the resulting blood clot compressed the trachea, leading to asphyxia. Given the rich blood supply of thyroid tissue and the high physiological similarity between rats and humans, postoperative hemorrhage is a serious complication, mandating meticulous hemostasis during surgery, and before closing the incision, the wound must be irrigated with sterile saline solution and carefully explored for active bleeding points. These outcomes highlight the importance of carefully titrating anesthesia based on individual animal responses and implementing thorough intraoperative hemostasis. Furthermore, close postoperative monitoring and timely intervention are essential to improving survival rates in rat thyroidectomy models.

Tail vein blood collection is the most commonly used non-anaesthetic blood collection method [41]. Similarly, Charlès [42] noted that the success rate of tail vein blood collection in rats under anesthesia was low at 25%. Therefore, in this study, the tail vein blood collection method without anesthesia was used to obtain sufficient blood samples for testing to verify the success of the hypothyroidism model in rats.

The thyroid gland is the sole organ responsible for producing thyroid hormones. Surgical removal of the thyroid results in the loss of this hormone source and disrupts feedback regulation within the hypothalamic-pituitary-thyroid axis, ultimately leading to hypothyroidism [43]. In numerous studies on animal models of hypothyroidism, preoperative baseline thyroid function assessment in rats has often been neglected. This research addressed this gap by incorporating preoperative serological evaluations, which revealed statistically equivalent TSH, FT4, and FT3 levels across all groups. This ensured uniform baseline conditions, thereby minimizing initial variability and improving the comparability and reliability of the results. Postoperative analysis at 14 days revealed characteristic hypothyroidism serum markers: elevated TSH and decreased FT4/FT3 levels in the surgical groups. These changes aligned with human clinical manifestations [44] and trends observed in other modeling approaches [8,26,45], confirming successful hypothyroidism model establishment at this stage. Notably, these serological trends remained stable at 28 and 90 days post-surgery, indicating excellent sustainability and reproducibility. Similarly, Logothetopoulos and Clark [46,47] reported that after most of the thyroid gland is removed, compensatory hypertrophy occurs in residual thyroid tissues, but for at least 4 months after surgery, thyroid function cannot be normalized. Linear regression revealed a strong correlation between thyroid tissue mass removed and hormone levels, with FT4 emerging as a more responsive indicator than FT3. In both clinical and animal studies, TSH and FT4 have consistently been the preferred indicators for diagnosing thyroid dysfunction [48]. The extent of surgical resection was an important cause of the difference in thyroid function among the different groups of rats. This strong correlation validates the precise controllability of surgical modeling, providing a quantifiable baseline for subsequent research interventions.

As a key protein synthesized and secreted by the thyroid under TSH stimulation, Tg is clinically used as a crucial biomarker for follow-up after total thyroidectomy—with typically very low or undetectable serum levels postsurgery [44]. Tg levels decreased in proportion to the extent of resection, supporting its utility as a sensitive biomarker of residual thyroid function.

In rats, the parathyroid glands are anatomically closely related to the thyroid lobes, which share a common cystic structure and are entirely dependent on the thyroid vascular system for blood supply. This close anatomical relationship makes these patients highly susceptible to direct mechanical injury or ischemic damage during thyroidectomy, significantly increasing the risk of postoperative hypoparathyroidism and secondary hypocalcemia [49]. Xie et al. found that supplementing drinking water with 0.1% calcium gluconate prevented significant differences in postoperative blood calcium between sham and surgical groups, indicating its potential to compensate for hypocalcemia following parathyroid injury [50]. Unfortunately, PTH levels were not evaluated in that study, limiting the interpretation of calcium homeostasis and the direct impact of the intervention on parathyroid gland activity. Georg et al. reported that serum phosphate and PTH levels show significant 24-hour fluctuations [51]. Therefore, critical factors influencing serum indicator measurements include blood collection timing, postoperative calcium supplementation protocols, and the activity status of the rats. Notably, no significant differences in PTH, Ca, or P levels were detected between groups, likely due to preserved parathyroid function and possible compensatory mechanisms [50].

Weight loss in rats following thyroidectomy is attributed primarily to thyroid hormone deficiency and the consequent systemic metabolic disturbances [19]. As THs are central regulators of energy metabolism and thermogenesis, the absence of THs markedly reduces the basal metabolic rate and overall energy expenditure [52]. Concurrently, hypothyroidism leads to decreased appetite and reduced food intake. Moreover, thyroid hormone loss impairs gastrointestinal motility and nutrient absorption, reducing the efficiency of nutrient utilization [53,54]. Together, these mechanisms result in significant weight loss. Consistent with these findings, Baki et al. reported that hypothyroidism induces weight reduction in animals [45], which aligns with the present findings.

THs deficiency is closely associated with mood disorders [8,21]. Hypothyroidism negatively affects language, cognitive function, memory capacity, and auditory-visual ability to varying degrees [44]; reduces cognitive performance [55,56]; and increases anxiety and depressive behaviors [13].

The thyroidectomized rats showed reduced travel distance and less center time in the OFT, reflecting decreased exploration and locomotor activity, likely due to hypothyroidism-induced metabolic and muscular impairment [57]. The reduced center activity also suggests increased anxiety-like behavior [58,59]. Similarly, in the EPMT, thyroidectomized rats exhibited a significant reduction in both the time spent in and the entries into the open arms, suggesting heightened anxiety-like behavior. This effect may be associated with dysregulated neurotransmitter systems modulated by THs [21,36]. Altered GABAergic signaling and Hypothalamic-Pituitary-Thyroid (HPT) axis dysfunction may further disrupt emotional and stress responses [60,61]. In the FST, prolonged immobility indicated depression-like behavior [62], associated with reduced monoamine function and neural plasticity [63,64]. In summary, our study demonstrated that hypothyroidism induced by thyroidectomy significantly affects behavioral performance in rats, manifesting as reduced locomotor activity, increased anxiety-like behaviors, and enhanced depression-like behaviors, which is consistent with previously published observations [13,62]. These behavioral alterations not only underscore the crucial role of thyroid hormones in regulating central nervous system excitability, emotional homeostasis, and the stress response but also suggest that hypothyroidism may contribute to behavioral abnormalities through multiple intertwined mechanisms, including disrupted energy metabolism, compromised neuroendocrine function, and altered neural plasticity.

Several limitations should be acknowledged. The small size of rat thyroid glands limited dynamic imaging of compensatory hyperplasia in residual tissue. Future studies may incorporate high-frequency micro-ultrasound to monitor postoperative thyroid remodeling. Additionally, comparative studies involving other modeling approaches (e.g., chemical induction) and inclusion of female subjects are warranted to evaluate generalizability and translational relevance.

## Conclusion

In conclusion, both total and subtotal thyroidectomy in rats successfully induced significant hypothyroid states, with the total resection group exhibiting more severe manifestations. This surgical approach provides a stable, reproducible, and controllable model of hypothyroidism that is well-suited for long-term pathophysiological and behavioral investigations. The model effectively recapitulates key clinical features of the disorder, thereby offering a valuable platform for future research into mechanism-based interventions.

## Supporting information

S1 Fig Schematic diagram of thyroidectomy. (A) Fixed skin preparation and antisepsis and sterile draping; (B) A longitudinal incision along the midline of the neck; (C) Layered dissection of the anterior cervical muscles; (D) Adequate exposure of the thyroid gland; (E, F) Precise dissection and hemostasis using an electrocautery pencil; (G) Complete resection of the thyroid tissue followed by exploration of the surgical field for active bleeding; (H) Confirmation of hemostasis prior to layered closure of the incision; (I) Complete excision of thyroid tissue specimens.
(TIF)

S1 Data Experiment-related data. (PDF)

S2 File Support information-experiment overview. (DOCX)
