# Supplementary material for: Establishment and investigation of a surgical model of hypothyroidism in Wistar rats
Source: PLoS One. 2026 Jan 20;21(1):e0340302. doi: 10.1371/journal.pone.0340302 (PMC12818658; doi:10.1371/journal.pone.0340302)
Supplement: S1 Data — (PDF) [file pone.0340302.s002.pdf]

| | | | | | |
|---|---|---|---|---|---|
| 0.06 | 0.361 | 0.564 | 1.066 | 1.728 | 2.524 |
| 0 | 0.301 | 0.504 | 1.006 | 1.668 | 2.464 |
| 0 | 1 | 2 | 4 | 8 | 16 |

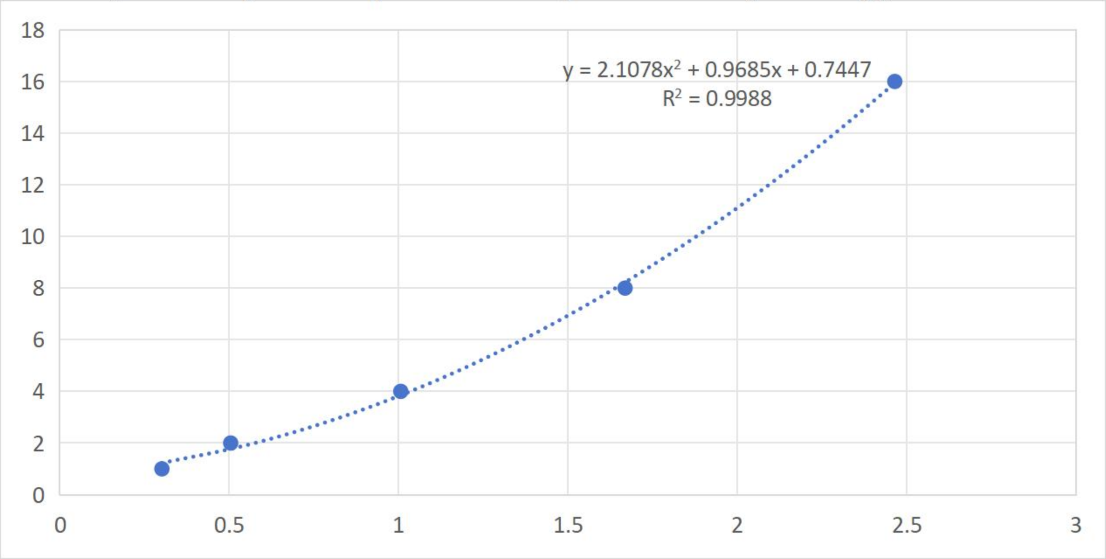


| Sample I | IOD （450r | Blank Coı | Dilution Fact | Concentration |
|---|---|---|---|---|
| 7 | 1.182 | 1.122 | 4.484832695 | 22.42416348 |
| 8 | 1.513 | 1.453 | 6.60193683 | 33.00968415 |
| 9 | 1.447 | 1.387 | 6.142929798 | 30.71464899 |
| 10 | 1.329 | 1.269 | 5.368045416 | 26.84022708 |
| 11 | 1.425 | 1.365 | 5.994008155 | 29.97004078 |
| 12 | 1.182 | 1.122 | 4.484832695 | 22.42416348 |
| 19 | 1.307 | 1.247 | 5.23006747 | 26.15033735 |
| 20 | 1.265 | 1.205 | 4.972320795 | 24.86160398 |
| 21 | 1.318 | 1.258 | 5.298801399 | 26.494007 |
| 22 | 1.204 | 1.144 | 4.611217741 | 23.0560887 |
| 23 | 1.172 | 1.112 | 4.428059443 | 22.14029722 |
| 24 | 1.18 | 1.120 | 4.47344432 | 22.3672216 |
| 25 | 1.093 | 1.033 | 3.994370694 | 19.97185347 |
| 26 | 1.305 | 1.245 | 5.217625195 | 26.08812598 |
| 27 | 1.158 | 1.098 | 4.349285111 | 21.74642556 |
| 28 | 1.243 | 1.183 | 4.840278414 | 24.20139207 |
| 29 | 1.422 | 1.362 | 5.973858743 | 29.86929372 |
| 30 | 1.347 | 1.287 | 5.482454078 | 27.41227039 |
| 31 | 1.415 | 1.355 | 5.926990995 | 29.63495498 |
| 32 | 1.089 | 1.029 | 3.97311156 | 19.8655578 |
| 33 | 1.361 | 1.301 | 5.572382888 | 27.86191444 |
| 34 | 1.326 | 1.266 | 5.349110097 | 26.74555048 |
| 35 | 1.366 | 1.306 | 5.604700561 | 28.0235028 |
| 36 | 1.284 | 1.224 | 5.087999373 | 25.43999686 |
| 37 | 1.32 | 1.260 | 5.31135328 | 26.5567664 |
| 38 | 1.392 | 1.332 | 5.774451347 | 28.87225674 |
| 39 | 1.157 | 1.097 | 4.34368999 | 21.71844995 |
| 40 | 1.148 | 1.088 | 4.293523603 | 21.46761802 |
| 41 | 1.293 | 1.233 | 5.143325654 | 25.71662827 |

| | | | | |
|---|---|---|---|---|
| 42 | 1.374 | 1.314 | 5.656628049 | 28.28314024 |
| 43 | 1.287 | 1.227 | 5.106403526 | 25.53201763 |
| 44 | 1.291 | 1.231 | 5.131001416 | 25.65500708 |
| 45 | 1.159 | 1.099 | 4.354884448 | 21.77442224 |
| 46 | 1.348 | 1.288 | 5.488850163 | 27.44425082 |
| 47 | 1.372 | 1.312 | 5.643620883 | 28.21810442 |
| 48 | 1.339 | 1.279 | 5.43143716 | 27.1571858 |
| 49 | 1.152 | 1.092 | 4.315777619 | 21.5788881 |
| 50 | 1.238 | 1.178 | 4.810553335 | 24.05276668 |
| 51 | 1.46 | 1.400 | 6.231888 | 31.15944 |
| 52 | 1.356 | 1.296 | 5.540170605 | 27.70085302 |
| 53 | 1.371 | 1.311 | 5.637123624 | 28.18561812 |
| 54 | 1.266 | 1.206 | 4.978371201 | 24.891856 |
| 55 | 1.205 | 1.145 | 4.617010995 | 23.08505498 |
| 56 | 1.156 | 1.096 | 4.338099085 | 21.69049542 |
| 57 | 1.254 | 1.194 | 4.906044561 | 24.5302228 |
| 58 | 1.319 | 1.259 | 5.305075232 | 26.52537616 |
| 59 | 1.39 | 1.330 | 5.76129242 | 28.8064621 |
| 60 | 1.235 | 1.175 | 4.792768875 | 23.96384438 |

| | | | | | |
|---|---|---|---|---|---|
| 0.058 | 0.342 | 0.558 | 1.06 | 1.722 | 2.508 |
| 0 | 0.284 | 0.5 | 1.002 | 1.664 | 2.45 |
| 0 | 1 | 2 | 4 | 8 | 16 |

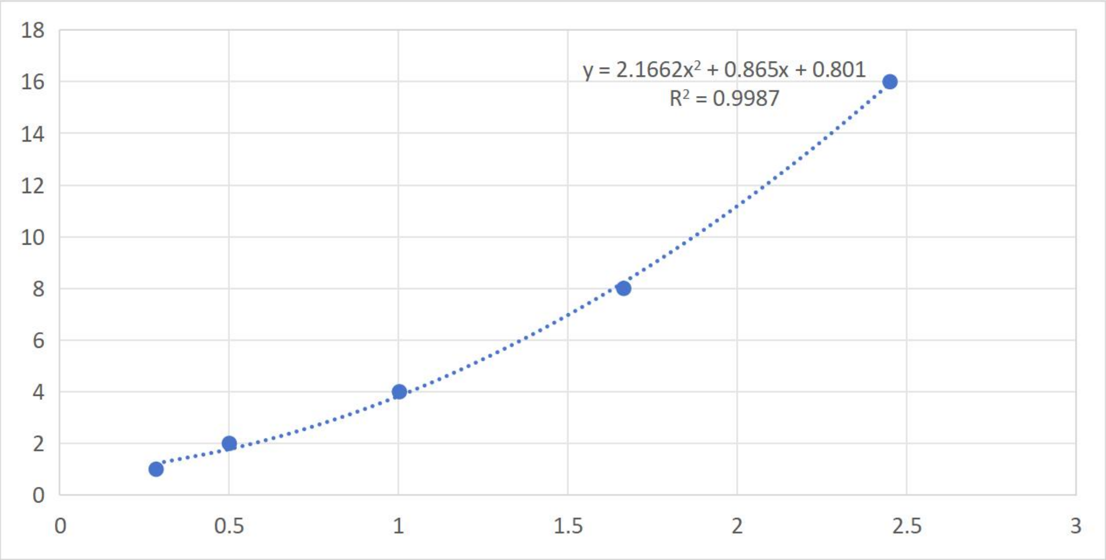


| Sample | IIOD （450ı | Blank Coı | Dilution Fact | Concentration |
|---|---|---|---|---|
| 7 | 1.182 | 1.124 | 4.509985091 | 22.54992546 |
| 8 | 1.513 | 1.455 | 6.645474555 | 33.22737278 |
| 9 | 1.447 | 1.389 | 6.18178015 | 30.90890075 |
| 10 | 1.329 | 1.271 | 5.399783294 | 26.99891647 |
| 11 | 1.425 | 1.367 | 6.031409112 | 30.15704556 |
| 12 | 1.182 | 1.124 | 4.509985091 | 22.54992546 |
| 19 | 1.307 | 1.249 | 5.260659166 | 26.30329583 |
| 20 | 1.265 | 1.207 | 5.000881304 | 25.00440652 |
| 21 | 1.318 | 1.26 | 5.32995912 | 26.6497956 |
| 22 | 1.204 | 1.146 | 4.637195119 | 23.1859756 |

| | | | | |
|---|---|---|---|---|
| 23 | 1.172 | 1.114 | 4.452855535 | 22.26427768 |
| 24 | 1.18 | 1.122 | 4.498524521 | 22.4926226 |
| 25 | 1.093 | 1.035 | 4.016762595 | 20.08381298 |
| 26 | 1.305 | 1.247 | 5.248115496 | 26.24057748 |
| 27 | 1.158 | 1.1 | 4.373602 | 21.86801 |
| 28 | 1.243 | 1.185 | 4.867857195 | 24.33928598 |
| 29 | 1.422 | 1.364 | 6.011066435 | 30.05533218 |
| 30 | 1.347 | 1.289 | 5.51517179 | 27.57585895 |
| 31 | 1.415 | 1.357 | 5.963751824 | 29.81875912 |
| 32 | 1.089 | 1.031 | 3.995401118 | 19.97700559 |
| 33 | 1.361 | 1.303 | 5.605888856 | 28.02944428 |
| 34 | 1.326 | 1.268 | 5.380688349 | 26.90344174 |
| 35 | 1.366 | 1.308 | 5.638493597 | 28.19246798 |
| 36 | 1.284 | 1.226 | 5.117453231 | 25.58726616 |
| 37 | 1.32 | 1.262 | 5.342615433 | 26.71307716 |
| 38 | 1.392 | 1.334 | 5.809784207 | 29.04892104 |
| 39 | 1.157 | 1.099 | 4.367973526 | 21.83986763 |
| 40 | 1.148 | 1.09 | 4.31751222 | 21.5875611 |
| 41 | 1.293 | 1.235 | 5.173217395 | 25.86608698 |
| 42 | 1.374 | 1.316 | 5.690886467 | 28.45443234 |
| 43 | 1.287 | 1.229 | 5.136002294 | 25.68001147 |
| 44 | 1.291 | 1.233 | 5.160795032 | 25.80397516 |
| 45 | 1.159 | 1.101 | 4.379234806 | 21.89617403 |
| 46 | 1.348 | 1.29 | 5.52162342 | 27.6081171 |
| 47 | 1.372 | 1.314 | 5.677762255 | 28.38881128 |
| 48 | 1.339 | 1.281 | 5.463714718 | 27.31857359 |
| 49 | 1.152 | 1.094 | 4.339896143 | 21.69948072 |
| 50 | 1.238 | 1.18 | 4.83791688 | 24.1895844 |
| 51 | 1.46 | 1.402 | 6.271621385 | 31.35810692 |
| 52 | 1.356 | 1.298 | 5.573392425 | 27.86696212 |
| 53 | 1.371 | 1.313 | 5.671206648 | 28.35603324 |
| 54 | 1.266 | 1.208 | 5.006977677 | 25.03488838 |
| 55 | 1.205 | 1.147 | 4.643027216 | 23.21513608 |
| 56 | 1.156 | 1.098 | 4.362349385 | 21.81174692 |
| 57 | 1.254 | 1.196 | 4.934107139 | 24.6705357 |
| 58 | 1.319 | 1.261 | 5.33628511 | 26.68142555 |
| 59 | 1.39 | 1.332 | 5.796504029 | 28.98252014 |
| 60 | 1.235 | 1.177 | 4.82000468 | 24.1000234 |

| | | | | | |
|---|---|---|---|---|---|
| 0.062 | 0.358 | 0.56 | 1.064 | 1.726 | 2.518 |
| 0 | 0.296 | 0.498 | 1.002 | 1.664 | 2.456 |
| 0 | 1 | 2 | 4 | 8 | 16 |

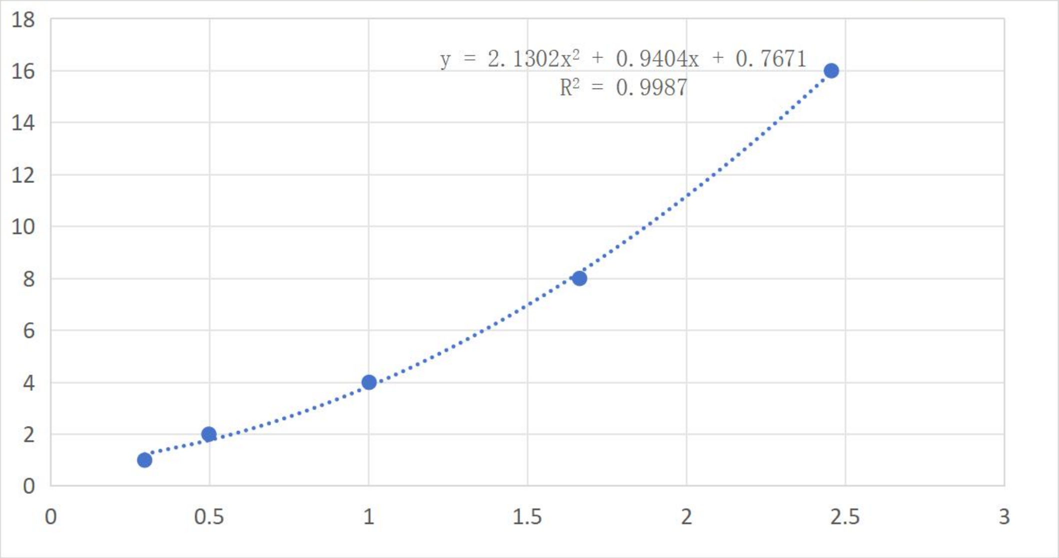


| Sample | IIOD （450r | Blank Co | Dilution Factc | Concentration |
|---|---|---|---|---|
| 7 | 1.191 | 1.129 | 4.544051858 | 22.72025929 |
| 8 | 1.51 | 1.448 | 6.595198061 | 32.9759903 |
| 9 | 1.45 | 1.388 | 6.176299229 | 30.88149614 |
| 10 | 1.326 | 1.264 | 5.359177619 | 26.7958881 |
| 11 | 1.422 | 1.36 | 5.98606192 | 29.9303096 |
| 12 | 1.188 | 1.126 | 4.526819855 | 22.63409928 |
| 19 | 1.311 | 1.249 | 5.26477373 | 26.32386865 |
| 20 | 1.200 | 1.138 | 4.595977929 | 22.97988964 |
| 21 | 1.315 | 1.253 | 5.289854372 | 26.44927186 |
| 22 | 1.207 | 1.145 | 4.636603455 | 23.18301728 |
| 23 | 1.176 | 1.114 | 4.458275279 | 22.2913764 |
| 24 | 1.184 | 1.122 | 4.503903497 | 22.51951748 |
| 25 | 1.120 | 1.058 | 4.146512393 | 20.73256196 |
| 26 | 1.312 | 1.25 | 5.2710375 | 26.3551875 |
| 27 | 1.162 | 1.1 | 4.379082 | 21.89541 |
| 28 | 1.250 | 1.188 | 4.890740189 | 24.45370094 |
| 29 | 1.420 | 1.358 | 5.972601353 | 29.86300676 |
| 30 | 1.355 | 1.293 | 5.54440994 | 27.7220497 |
| 31 | 1.408 | 1.346 | 5.892195823 | 29.46097912 |
| 32 | 1.098 | 1.036 | 4.027689539 | 20.1384477 |
| 33 | 1.366 | 1.304 | 5.615607763 | 28.07803882 |
| 34 | 1.320 | 1.258 | 5.321301033 | 26.60650516 |
| 35 | 1.361 | 1.299 | 5.58318121 | 27.91590605 |
| 36 | 1.292 | 1.23 | 5.14657158 | 25.7328579 |
| 37 | 1.326 | 1.264 | 5.359177619 | 26.7958881 |
| 38 | 1.402 | 1.34 | 5.85222312 | 29.2611156 |
| 39 | 1.150 | 1.088 | 4.311866669 | 21.55933334 |
| 40 | 1.152 | 1.09 | 4.32302662 | 21.6151331 |

| | | | | |
|---|---|---|---|---|
| 41 | 1.301 | 1.239 | 5.202370354 | 26.01185177 |
| 42 | 1.377 | 1.315 | 5.687321095 | 28.43660548 |
| 43 | 1.291 | 1.229 | 5.140393018 | 25.70196509 |
| 44 | 1.291 | 1.229 | 5.140393018 | 25.70196509 |
| 45 | 1.163 | 1.101 | 4.38471097 | 21.92355485 |
| 46 | 1.344 | 1.282 | 5.473727625 | 27.36863812 |
| 47 | 1.380 | 1.318 | 5.706968745 | 28.53484372 |
| 48 | 1.340 | 1.278 | 5.448152777 | 27.24076388 |
| 49 | 1.166 | 1.104 | 4.401623443 | 22.00811722 |
| 50 | 1.243 | 1.181 | 4.848832282 | 24.24416141 |
| 51 | 1.456 | 1.394 | 6.217498927 | 31.08749464 |
| 52 | 1.362 | 1.3 | 5.589658 | 27.94829 |
| 53 | 1.374 | 1.312 | 5.667711789 | 28.33855894 |
| 54 | 1.260 | 1.198 | 4.950970761 | 24.7548538 |
| 55 | 1.210 | 1.148 | 4.654078301 | 23.2703915 |
| 56 | 1.166 | 1.104 | 4.401623443 | 22.00811722 |
| 57 | 1.260 | 1.198 | 4.950970761 | 24.7548538 |
| 58 | 1.314 | 1.252 | 5.283577821 | 26.4178891 |
| 59 | 1.403 | 1.341 | 5.858874586 | 29.29437293 |
| 60 | 1.230 | 1.168 | 4.771557165 | 23.85778582 |

| | | | | | |
|---|---|---|---|---|---|
| 0.065 | 0.362 | 0.563 | 1.062 | 1.718 | 2.521 |
| 0 | 0.297 | 0.498 | 0.997 | 1.653 | 2.456 |
| 0 | 1 | 2 | 4 | 8 | 16 |

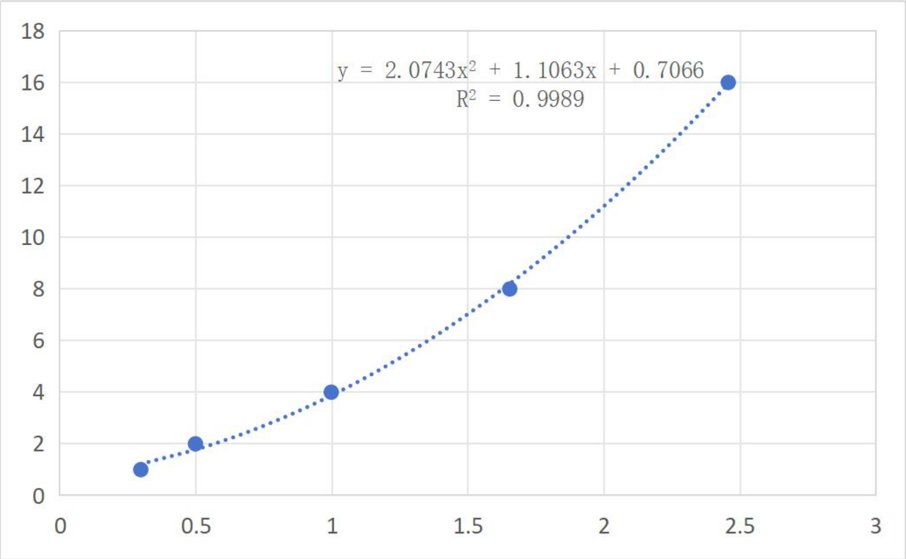


| Sample I | IOD （450r | Blank Co | Dilution Factc | Concentration |
|---|---|---|---|---|
| 7 | 1.191 | 1.126 | 4.582248987 | 22.91124493 |
| 8 | 1.51 | 1.445 | 6.636393758 | 33.18196879 |
| 9 | 1.45 | 1.385 | 6.217799618 | 31.08899809 |
| 10 | 1.326 | 1.261 | 5.40003229 | 27.00016145 |
| 11 | 1.422 | 1.357 | 6.027566761 | 30.1378338 |
| 12 | 1.188 | 1.123 | 4.564934785 | 22.82467392 |

| | | | | |
|---|---|---|---|---|
| 19 | 1.311 | 1.246 | 5.305433739 | 26.52716869 |
| 20 | 1.200 | 1.135 | 4.634415618 | 23.17207809 |
| 21 | 1.315 | 1.25 | 5.33056875 | 26.65284375 |
| 22 | 1.207 | 1.142 | 4.675221985 | 23.37610993 |
| 23 | 1.176 | 1.111 | 4.49605135 | 22.48025675 |
| 24 | 1.184 | 1.119 | 4.541907262 | 22.70953631 |
| 25 | 1.120 | 1.055 | 4.182494258 | 20.91247129 |
| 26 | 1.312 | 1.247 | 5.311711269 | 26.55855634 |
| 27 | 1.162 | 1.097 | 4.416442389 | 22.08221194 |
| 28 | 1.250 | 1.185 | 4.930349418 | 24.65174709 |
| 29 | 1.420 | 1.355 | 6.014103158 | 30.07051579 |
| 30 | 1.355 | 1.29 | 5.58556963 | 27.92784815 |
| 31 | 1.408 | 1.343 | 5.933670021 | 29.6683501 |
| 32 | 1.098 | 1.033 | 4.062870613 | 20.31435306 |
| 33 | 1.366 | 1.301 | 5.656858554 | 28.28429277 |
| 34 | 1.320 | 1.255 | 5.362080858 | 26.81040429 |
| 35 | 1.361 | 1.296 | 5.624392269 | 28.12196134 |
| 36 | 1.292 | 1.227 | 5.186948905 | 25.93474452 |
| 37 | 1.326 | 1.261 | 5.40003229 | 27.00016145 |
| 38 | 1.402 | 1.337 | 5.893677477 | 29.46838738 |
| 39 | 1.150 | 1.085 | 4.348853318 | 21.74426659 |
| 40 | 1.152 | 1.087 | 4.360076677 | 21.80038338 |
| 41 | 1.301 | 1.236 | 5.242886613 | 26.21443306 |
| 42 | 1.377 | 1.312 | 5.728649459 | 28.6432473 |
| 43 | 1.291 | 1.226 | 5.180754347 | 25.90377173 |
| 44 | 1.291 | 1.226 | 5.180754347 | 25.90377173 |
| 45 | 1.163 | 1.098 | 4.422101777 | 22.11050889 |
| 46 | 1.344 | 1.279 | 5.514782686 | 27.57391343 |
| 47 | 1.380 | 1.315 | 5.748315918 | 28.74157959 |
| 48 | 1.340 | 1.275 | 5.489166438 | 27.44583219 |
| 49 | 1.166 | 1.101 | 4.439104834 | 22.19552417 |
| 50 | 1.243 | 1.178 | 4.888294321 | 24.44147161 |
| 51 | 1.456 | 1.391 | 6.258986958 | 31.29493479 |
| 52 | 1.362 | 1.297 | 5.630877229 | 28.15438614 |
| 53 | 1.374 | 1.309 | 5.709020338 | 28.54510169 |
| 54 | 1.260 | 1.195 | 4.990780758 | 24.95390379 |
| 55 | 1.210 | 1.145 | 4.692772658 | 23.46386329 |
| 56 | 1.166 | 1.101 | 4.439104834 | 22.19552417 |
| 57 | 1.260 | 1.195 | 4.990780758 | 24.95390379 |
| 58 | 1.314 | 1.249 | 5.324278774 | 26.62139387 |
| 59 | 1.403 | 1.338 | 5.900332529 | 29.50166265 |
| 60 | 1.230 | 1.165 | 4.810731318 | 24.05365659 |

| | | | | | |
|---|---|---|---|---|---|
| 0.061 | 0.366 | 0.56 | 1.063 | 1.72 | 2.52 |
| 0 | 0.305 | 0.499 | 1.002 | 1.659 | 2.459 |
| 0 | 1 | 2 | 4 | 8 | 16 |

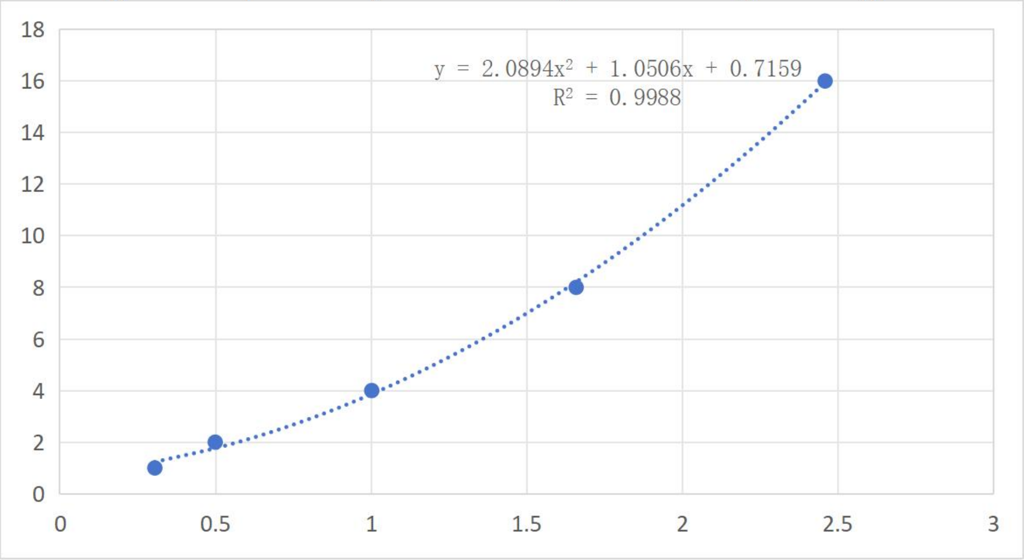


| Sample II | OD （450r | Blank Co | Dilution Fact | Concentration |
|---|---|---|---|---|
| 7 | 1.201 | 1.14 | 4.62896824 | 23.1448412 |
| 8 | 1.486 | 1.425 | 6.455792875 | 32.27896438 |
| 9 | 1.453 | 1.392 | 6.226890362 | 31.13445181 |
| 10 | 1.332 | 1.271 | 5.426515025 | 27.13257513 |
| 11 | 1.409 | 1.348 | 5.928765898 | 29.64382949 |
| 12 | 1.176 | 1.115 | 4.484913315 | 22.42456658 |
| 19 | 1.293 | 1.232 | 5.181580666 | 25.90790333 |
| 20 | 1.218 | 1.157 | 4.728417421 | 23.6420871 |
| 21 | 1.306 | 1.245 | 5.262519235 | 26.31259618 |
| 22 | 1.215 | 1.154 | 4.71077981 | 23.55389905 |
| 23 | 1.166 | 1.105 | 4.428022635 | 22.14011318 |
| 24 | 1.187 | 1.126 | 4.547975714 | 22.73987857 |
| 25 | 1.130 | 1.069 | 4.226676233 | 21.13338117 |
| 26 | 1.318 | 1.257 | 5.337858581 | 26.6892929 |
| 27 | 1.177 | 1.116 | 4.490625366 | 22.45312683 |
| 28 | 1.238 | 1.177 | 4.846962613 | 24.23481306 |
| 29 | 1.395 | 1.334 | 5.835604706 | 29.17802353 |
| 30 | 1.342 | 1.281 | 5.490342513 | 27.45171257 |
| 31 | 1.417 | 1.356 | 5.982368598 | 29.91184299 |
| 32 | 1.115 | 1.054 | 4.14438029 | 20.72190145 |
| 33 | 1.344 | 1.283 | 5.503158157 | 27.51579078 |
| 34 | 1.326 | 1.265 | 5.388419115 | 26.94209558 |
| 35 | 1.352 | 1.291 | 5.554587881 | 27.77293941 |
| 36 | 1.280 | 1.219 | 5.101348313 | 25.50674157 |
| 37 | 1.339 | 1.278 | 5.47115039 | 27.35575195 |
| 38 | 1.396 | 1.335 | 5.842231915 | 29.21115958 |
| 39 | 1.166 | 1.105 | 4.428022635 | 22.14011318 |
| 40 | 1.201 | 1.14 | 4.62896824 | 23.1448412 |
| 41 | 1.287 | 1.226 | 5.144462594 | 25.72231297 |

| | | | | |
|---|---|---|---|---|
| 42 | 1.364 | 1.303 | 5.632233925 | 28.16116962 |
| 43 | 1.300 | 1.239 | 5.225075217 | 26.12537609 |
| 44 | 1.296 | 1.235 | 5.200196115 | 26.00098058 |
| 45 | 1.152 | 1.091 | 4.349077721 | 21.74538861 |
| 46 | 1.356 | 1.295 | 5.580403035 | 27.90201518 |
| 47 | 1.368 | 1.307 | 5.658249661 | 28.2912483 |
| 48 | 1.332 | 1.271 | 5.426515025 | 27.13257513 |
| 49 | 1.183 | 1.122 | 4.52498543 | 22.62492715 |
| 50 | 1.240 | 1.179 | 4.858909065 | 24.29454533 |
| 51 | 1.447 | 1.386 | 6.185760642 | 30.92880321 |
| 52 | 1.354 | 1.293 | 5.567487101 | 27.8374355 |
| 53 | 1.362 | 1.301 | 5.619251129 | 28.09625565 |
| 54 | 1.254 | 1.193 | 4.943002261 | 24.7150113 |
| 55 | 1.220 | 1.159 | 4.740196721 | 23.70098361 |
| 56 | 1.162 | 1.101 | 4.405383369 | 22.02691685 |
| 57 | 1.257 | 1.196 | 4.96112879 | 24.80564395 |
| 58 | 1.322 | 1.261 | 5.363105417 | 26.81552709 |
| 59 | 1.396 | 1.335 | 5.842231915 | 29.21115958 |
| 60 | 1.223 | 1.162 | 4.757897014 | 23.78948507 |

| | | | | | |
|---|---|---|---|---|---|
| 0.063 | 0.354 | 0.57 | 1.058 | 1.723 | 2.53 |
| 0 | 0.291 | 0.507 | 0.995 | 1.66 | 2.467 |
| 0 | 1 | 2 | 4 | 8 | 16 |

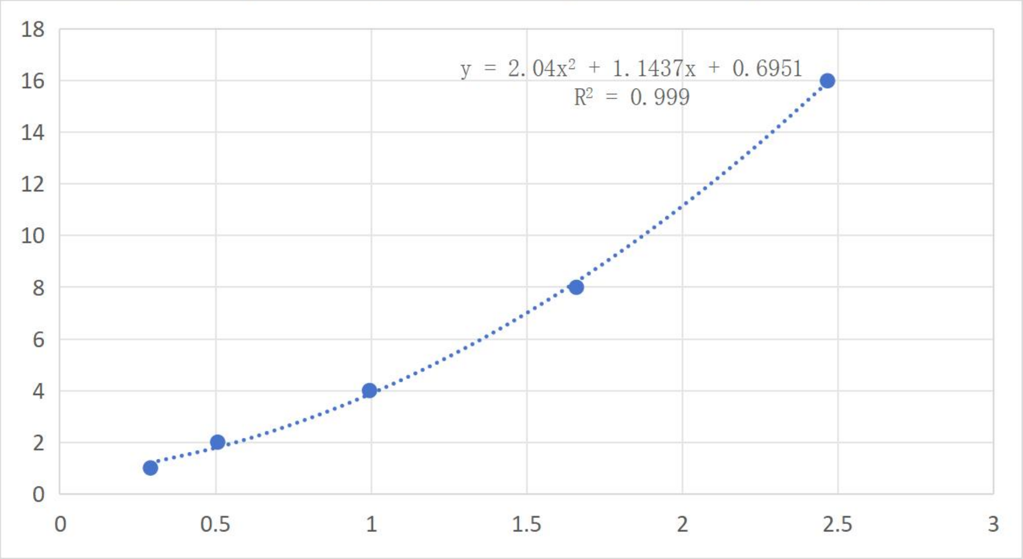


| Sample | IOD （450r | Blank Co | Dilution Fact | Concentration |
|---|---|---|---|---|
| 7 | 1.201 | 1.138 | 4.63852036 | 23.1926018 |
| 8 | 1.486 | 1.423 | 6.45344026 | 32.2672013 |
| 9 | 1.453 | 1.39 | 6.226327 | 31.131635 |
| 10 | 1.332 | 1.269 | 5.43159174 | 27.1579587 |
| 11 | 1.409 | 1.346 | 5.93042084 | 29.6521042 |
| 12 | 1.176 | 1.113 | 4.49512686 | 22.4756343 |

| | | | | |
|---|---|---|---|---|
| 19 | 1.293 | 1.23 | 5.188167 | 25.940835 |
| 20 | 1.218 | 1.155 | 4.7374845 | 23.6874225 |
| 21 | 1.306 | 1.243 | 5.26861906 | 26.3430953 |
| 22 | 1.215 | 1.152 | 4.71993456 | 23.5996728 |
| 23 | 1.166 | 1.103 | 4.43848346 | 22.1924173 |
| 24 | 1.187 | 1.124 | 4.55790584 | 22.7895292 |
| 25 | 1.130 | 1.067 | 4.23794546 | 21.1897273 |
| 26 | 1.318 | 1.255 | 5.3434945 | 26.7174725 |
| 27 | 1.177 | 1.114 | 4.50081364 | 22.5040682 |
| 28 | 1.238 | 1.175 | 4.8554225 | 24.2771125 |
| 29 | 1.395 | 1.332 | 5.83792536 | 29.1896268 |
| 30 | 1.342 | 1.279 | 5.49500794 | 27.4750397 |
| 31 | 1.417 | 1.354 | 5.98363444 | 29.9181722 |
| 32 | 1.115 | 1.052 | 4.15594856 | 20.7797428 |
| 33 | 1.344 | 1.281 | 5.50774014 | 27.5387007 |
| 34 | 1.326 | 1.263 | 5.39373786 | 26.9686893 |
| 35 | 1.352 | 1.289 | 5.55883214 | 27.7941607 |
| 36 | 1.280 | 1.217 | 5.10840446 | 25.5420223 |
| 37 | 1.339 | 1.276 | 5.47594024 | 27.3797012 |
| 38 | 1.396 | 1.333 | 5.84450566 | 29.2225283 |
| 39 | 1.166 | 1.103 | 4.43848346 | 22.1924173 |
| 40 | 1.201 | 1.138 | 4.63852036 | 23.1926018 |
| 41 | 1.287 | 1.224 | 5.15126784 | 25.7563392 |
| 42 | 1.364 | 1.301 | 5.63595974 | 28.1797987 |
| 43 | 1.300 | 1.237 | 5.23140166 | 26.1570083 |
| 44 | 1.296 | 1.233 | 5.20667166 | 26.0333583 |
| 45 | 1.152 | 1.089 | 4.35986814 | 21.7993407 |
| 46 | 1.356 | 1.293 | 5.58447606 | 27.9223803 |
| 47 | 1.368 | 1.305 | 5.6617995 | 28.3089975 |
| 48 | 1.332 | 1.269 | 5.43159174 | 27.1579587 |
| 49 | 1.183 | 1.12 | 4.53502 | 22.6751 |
| 50 | 1.240 | 1.177 | 4.86730606 | 24.3365303 |
| 51 | 1.447 | 1.384 | 6.18551104 | 30.9275552 |
| 52 | 1.354 | 1.291 | 5.57164594 | 27.8582297 |
| 53 | 1.362 | 1.299 | 5.62306434 | 28.1153217 |
| 54 | 1.254 | 1.191 | 4.95094794 | 24.7547397 |
| 55 | 1.220 | 1.157 | 4.74920486 | 23.7460243 |
| 56 | 1.162 | 1.099 | 4.41594034 | 22.0797017 |
| 57 | 1.257 | 1.194 | 4.96897524 | 24.8448762 |
| 58 | 1.322 | 1.259 | 5.36858354 | 26.8429177 |
| 59 | 1.396 | 1.333 | 5.84450566 | 29.2225283 |
| 60 | 1.223 | 1.16 | 4.766816 | 23.83408 |

| | |
|---|---|
| 23.16872 | 22.82383936 |
| 32.27308 | 32.82353029 |
| 31.13304 | 30.97668847 |
| 27.14527 | 26.98762116 |
| 29.64797 | 29.91519391 |
| 22.4501 | 22.5555105 |
| 25.92437 | 26.19223481 |
| 23.66475 | 23.89124797 |
| 26.32785 | 26.48360162 |
| 23.57679 | 23.3257939 |
| 22.16627 | 22.25145642 |
| 22.7647 | 22.60305096 |
| 21.16155 | 20.6706347 |
| 26.70338 | 26.44153545 |
| 22.4786 | 22.09154209 |
| 24.25596 | 24.35967528 |
| 29.18383 | 29.7042998 |
| 27.46338 | 27.59412991 |
| 29.91501 | 29.73550975 |
| 20.75082 | 20.2995014 |
| 27.52725 | 27.88469697 |
| 26.95539 | 26.82944776 |
| 27.78355 | 27.97015638 |
| 25.52438 | 25.62393822 |
| 27.36773 | 26.96689105 |
| 29.21684 | 29.18072811 |
| 22.16627 | 21.86574133 |
| 23.16872 | 22.13468977 |
| 25.73933 | 25.88127538 |
| 28.17048 | 28.35973228 |
| 26.14119 | 25.85002505 |
| 26.01717 | 25.84984299 |
| 21.77236 | 21.87489822 |
| 27.9122 | 27.63655249 |
| 28.30012 | 28.4139308 |
| 27.14527 | 27.24214822 |
| 22.65001 | 22.13033956 |
| 24.31554 | 24.25984329 |
| 30.92818 | 31.1260558 |
| 27.84783 | 27.89435941 |
| 28.10579 | 28.27281489 |
| 24.73488 | 24.8508755 |
| 23.7235 | 23.41357563 |
| 22.05331 | 21.96875038 |
| 24.82526 | 24.76000604 |
| 26.82922 | 26.65075492 |
| 29.21684 | 29.16978428 |
| 23.81178 | 23.93314588 |

Report
FT3

| Group | Mean | Std. Deviation |
|---|---|---|
| 1 | 25.90248078 | 3.654765219 |
| 2 | 25.7552292 | 3.116636913 |
| 3 | 25.84987241 | 2.659929241 |
| Total | 25.8275333 | 3.033528679 |

Tests of Normality

| | Group | Kolmogorov-Smirnov(K)a | | | Shapiro-Wilk | |
|---|---|---|---|---|---|---|
| | | Statistic | df | Sig. | Statisticdf | |
| FT3 | 1 | 0.209 | 12 | 0.155 | 0.873 | 12 |
| | 2 | 0.155 | 18 | .200* | 0.91 | 18 |
| | 3 | 0.091 | 18 | .200* | 0.966 | 18 |

* This is a lower bound of the true significance.
a Lilliefors Significance Correction

ANOVA
FT3

| | Sum of Squaresdf | | Mean Square | F | Sig. |
|---|---|---|---|---|---|
| Between G | 0.17 | 2 | 0.085 | 0.009 | 0.991 |
| Within Gr | 432.337 | 45 | 9.607 | | |
| Total | 432.508 | 47 | | | |

Multiple Comparisons
Dependent Variable: FT3

| | (I) Group | (J) Group | Mean Differenc | Std. Error | Sig. | 95% Confidence |
|---|---|---|---|---|---|---|
| | | | | | | Lower Bound |
| LSD(L) | 1 | 2 | 0.147251579 | 1.155151416 | 0.899 | -2.179342803 |
| | | 3 | 0.052608373 | 1.155151416 | 0.964 | -2.27398601 |
| | 2 | 1 | -0.147251579 | 1.155151416 | 0.899 | -2.473845962 |
| | | 3 | -0.094643207 | 1.033198837 | 0.927 | -2.175612485 |
| | 3 | 1 | -0.052608373 | 1.155151416 | 0.964 | -2.379202755 |
| | | 2 | 0.094643207 | 1.033198837 | 0.927 | -1.986326071 |
| Tamhane | 1 | 2 | 0.147251579 | 1.285590847 | 0.999 | -3.186362707 |
| | | 3 | 0.052608373 | 1.227264048 | 1 | -3.165315525 |
| | 2 | 1 | -0.147251579 | 1.285590847 | 0.999 | -3.480865866 |
| | | 3 | -0.094643207 | 0.965765362 | 1 | -2.522883249 |
| | 3 | 1 | -0.052608373 | 1.227264048 | 1 | -3.27053227 |
| | | 2 | 0.094643207 | 0.965765362 | 1 | -2.333596836 |
| Dunnett T | 1 | 2 | 0.147251579 | 1.285590847 | 0.999 | -3.173680451 |
| | | 3 | 0.052608373 | 1.227264048 | 1 | -3.150915942 |
| | 2 | 1 | -0.147251579 | 1.285590847 | 0.999 | -3.468183609 |
| | | 3 | -0.094643207 | 0.965765362 | 1 | -2.517691447 |

| | | | | | | |
|---|---|---|---|---|---|---|
| | 3 | 1 | -0.052608373 | 1.227264048 | 1 | -3.256132687 |
| | | 2 | 0.094643207 | 0.965765362 | 1 | -2.328405034 |
| Dunnett t | 1 | 3 | 0.052608373 | 1.155151416 | 0.999 | -2.592292557 |
| | 2 | 3 | -0.094643207 | 1.033198837 | 0.994 | -2.460314515 |

Dunnett t-test treats one group as a control and compares all other groups against it

| Sig. |
| --- |
| 0.072 |
| 0.087 |
| 0.726 |

| Interval |
| --- |
| Upper Bound |
| 2.473845962 |
| 2.379202755 |
| 2.179342803 |
| 1.986326071 |
| 2.27398601 |
| 2.175612485 |
| 3.480865866 |
| 3.27053227 |
| 3.186362707 |
| 2.333596836 |
| 3.165315525 |
| 2.522883249 |
| 3.468183609 |
| 3.256132687 |
| 3.173680451 |
| 2.328405034 |

3.150915942
2.517691447
2.697509302
2.271028102
[illegible].

| | | | | | |
|---|---|---|---|---|---|
| 0.07 | 0.524 | 0.936 | 1.455 | 1.832 | 2.694 |
| 0 | 0.454 | 0.866 | 1.385 | 1.762 | 2.624 |
| 0 | 7.5 | 15 | 30 | 60 | 120 |

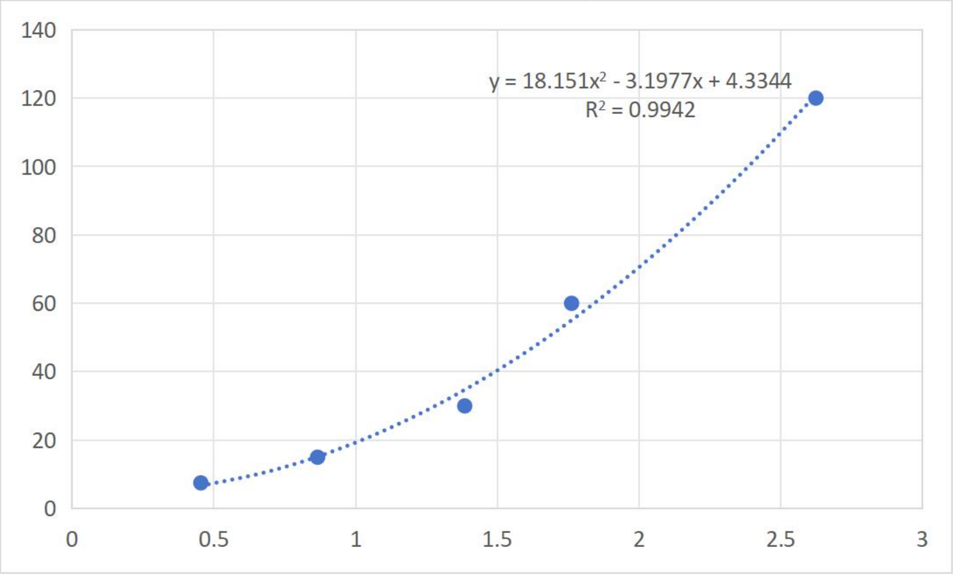


| Sample ID | OD (450nm) | Blank Corrected | Dilution Factor | Concentration |
|---|---|---|---|---|
| 7 | 0.792 | 0.722 | 11.48748648 | 57.43743242 |
| 8 | 0.763 | 0.693 | 10.8353935 | 54.1769675 |
| 9 | 0.756 | 0.686 | 10.6825658 | 53.41282898 |
| 10 | 0.785 | 0.715 | 11.32728948 | 56.63644738 |
| 11 | 0.782 | 0.712 | 11.25917814 | 56.29589072 |
| 12 | 0.823 | 0.753 | 12.21831226 | 61.0915613 |
| 19 | 0.791 | 0.721 | 11.46449229 | 57.32246146 |
| 20 | 0.767 | 0.697 | 10.92352226 | 54.6176113 |
| 21 | 0.881 | 0.811 | 13.67935917 | 68.39679586 |
| 22 | 0.764 | 0.694 | 10.85737124 | 54.28685618 |
| 23 | 0.789 | 0.719 | 11.41861281 | 57.09306406 |
| 24 | 0.774 | 0.704 | 11.07914522 | 55.39572608 |
| 25 | 0.803 | 0.733 | 11.74281854 | 58.7140927 |
| 26 | 0.762 | 0.692 | 10.81345206 | 54.06726032 |
| 27 | 0.852 | 0.782 | 12.93357072 | 64.66785362 |
| 28 | 0.838 | 0.768 | 12.58446182 | 62.92230912 |
| 29 | 0.872 | 0.802 | 13.4446404 | 67.22320202 |
| 30 | 0.862 | 0.792 | 13.18729046 | 65.93645232 |
| 31 | 0.744 | 0.674 | 10.42471388 | 52.12356938 |
| 32 | 0.815 | 0.745 | 12.02637228 | 60.13186138 |
| 33 | 0.842 | 0.772 | 12.68348118 | 63.41740592 |
| 34 | 0.805 | 0.735 | 11.78971448 | 58.94857238 |
| 35 | 0.892 | 0.822 | 13.97023088 | 69.85115442 |
| 36 | 0.859 | 0.789 | 13.11079337 | 65.55396686 |
| 37 | 0.831 | 0.761 | 12.41257557 | 62.06287786 |
| 38 | 0.753 | 0.683 | 10.61761274 | 53.0880637 |
| 39 | 0.87 | 0.800 | 13.39288 | 66.9644 |
| 40 | 0.849 | 0.779 | 12.85816269 | 64.29081346 |
| 41 | 0.767 | 0.697 | 10.92352226 | 54.6176113 |
| 42 | 0.834 | 0.764 | 12.4860233 | 62.43011648 |
| 43 | 0.901 | 0.831 | 14.21148401 | 71.05742006 |
| 44 | 0.822 | 0.752 | 12.1941927 | 60.97096352 |
| 45 | 0.81 | 0.740 | 11.9075896 | 59.537948 |
| 46 | 0.769 | 0.699 | 10.96780445 | 54.83902226 |

| | | | | |
|---|---|---|---|---|
| 47 | 0.876 | 0.806 | 13.54859684 | 67.74298418 |
| 48 | 0.869 | 0.799 | 13.36705425 | 66.83527126 |
| 49 | 0.818 | 0.748 | 12.0980775 | 60.49038752 |
| 50 | 0.761 | 0.691 | 10.79154693 | 53.95773466 |
| 51 | 0.809 | 0.739 | 11.88394197 | 59.41970986 |
| 52 | 0.914 | 0.844 | 14.56515194 | 72.82575968 |
| 53 | 0.876 | 0.806 | 13.54859684 | 67.74298418 |
| 54 | 0.901 | 0.831 | 14.21148401 | 71.05742006 |
| 55 | 0.737 | 0.667 | 10.27671434 | 51.3835717 |
| 56 | 0.793 | 0.723 | 11.51051698 | 57.5525849 |
| 57 | 0.784 | 0.714 | 11.3045494 | 56.52274698 |
| 58 | 0.786 | 0.716 | 11.35006586 | 56.75032928 |
| 59 | 0.795 | 0.725 | 11.55668688 | 57.78343438 |
| 60 | 0.77 | 0.700 | 10.99 | 54.95 |
| | | | | |
| 7 | 0.792 | 0.72 | 10.9835816 | 54.917908 |
| 8 | 0.763 | 0.691 | 10.31065156 | 51.55325782 |
| 9 | 0.756 | 0.684 | 10.15271686 | 50.76358432 |
| 10 | 0.785 | 0.713 | 10.81840224 | 54.09201118 |
| 11 | 0.782 | 0.71 | 10.7481464 | 53.740732 |
| 12 | 0.823 | 0.751 | 11.73611044 | 58.68055222 |
| 19 | 0.791 | 0.719 | 10.95987748 | 54.79938742 |
| 20 | 0.767 | 0.695 | 10.4016851 | 52.0084255 |
| 21 | 0.881 | 0.809 | 13.23617836 | 66.18089182 |
| 22 | 0.764 | 0.692 | 10.33335642 | 51.66678208 |
| 23 | 0.789 | 0.717 | 10.91257632 | 54.56288158 |
| 24 | 0.774 | 0.702 | 10.56236778 | 52.81183888 |
| 25 | 0.803 | 0.731 | 11.24668228 | 56.23341142 |
| 26 | 0.762 | 0.69 | 10.2879824 | 51.439912 |
| 27 | 0.852 | 0.78 | 12.4711376 | 62.355688 |
| 28 | 0.838 | 0.766 | 12.11254966 | 60.56274832 |
| 29 | 0.872 | 0.8 | 12.99554 | 64.9777 |
| 30 | 0.862 | 0.79 | 12.7315544 | 63.657772 |
| 31 | 0.744 | 0.672 | 9.886040096 | 49.43020048 |
| 32 | 0.815 | 0.743 | 11.53862616 | 57.69313078 |
| 33 | 0.842 | 0.77 | 12.2142896 | 61.071448 |
| 34 | 0.805 | 0.733 | 11.29498272 | 56.47491358 |
| 35 | 0.892 | 0.82 | 13.5342176 | 67.671088 |
| 36 | 0.859 | 0.787 | 12.65305464 | 63.26527318 |
| 37 | 0.831 | 0.759 | 11.93587876 | 59.67939382 |
| 38 | 0.753 | 0.681 | 10.08556588 | 50.42782942 |
| 39 | 0.87 | 0.798 | 12.94245738 | 64.71228688 |
| 40 | 0.849 | 0.777 | 12.39370848 | 61.96854238 |
| 41 | 0.767 | 0.695 | 10.4016851 | 52.0084255 |
| 42 | 0.834 | 0.762 | 12.01138074 | 60.05690368 |
| 43 | 0.901 | 0.829 | 13.7812798 | 68.90639902 |
| 44 | 0.822 | 0.75 | 11.7113 | 58.5565 |
| 45 | 0.81 | 0.738 | 11.41635834 | 57.08179168 |
| 46 | 0.769 | 0.697 | 10.447416 | 52.23707998 |
| 47 | 0.876 | 0.804 | 13.1021335 | 65.51066752 |
| 48 | 0.869 | 0.797 | 12.9159696 | 64.57984798 |
| 49 | 0.818 | 0.746 | 11.6124151 | 58.06207552 |
| 50 | 0.761 | 0.689 | 10.26534892 | 51.32674462 |
| 51 | 0.809 | 0.737 | 11.39201184 | 56.96005918 |

| | | | | |
|---|---|---|---|---|
| 52 | 0.914 | 0.842 | 14.14325082 | 70.71625408 |
| 53 | 0.876 | 0.804 | 13.1021335 | 65.51066752 |
| 54 | 0.901 | 0.829 | 13.7812798 | 68.90639902 |
| 55 | 0.737 | 0.665 | 9.7328519 | 48.6642595 |
| 56 | 0.793 | 0.721 | 11.0073214 | 55.03660702 |
| 57 | 0.784 | 0.712 | 10.79494794 | 53.97473968 |
| 58 | 0.786 | 0.714 | 10.84189222 | 54.20946112 |
| 59 | 0.795 | 0.723 | 11.05490808 | 55.27454038 |
| 60 | 0.77 | 0.698 | 10.47033498 | 52.35167488 |

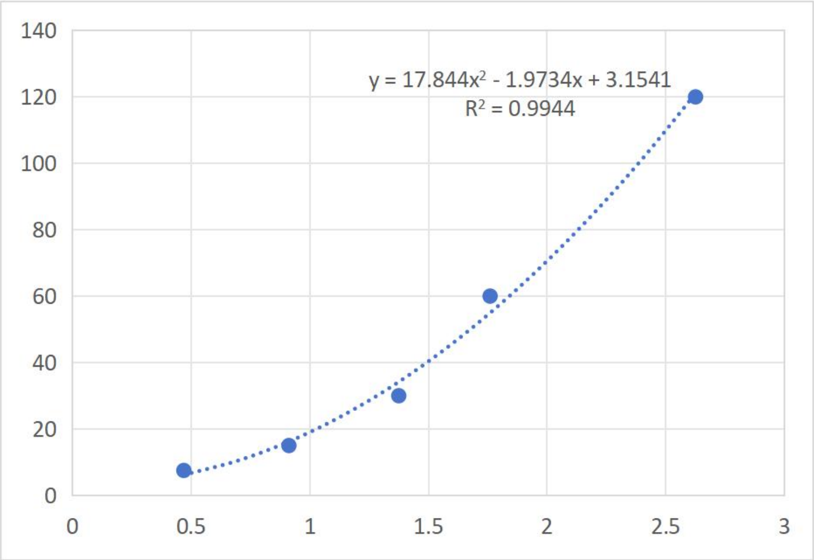

y = 17.844x² - 1.9734x + 3.1541
R² = 0.9944
140
120
100
80
60
40
20
0
0
0.5
1
1.5
2
2.5
3

| | | | | | |
|---|---|---|---|---|---|
| 0.066 | 0.528 | 0.974 | 1.458 | 1.836 | 2.685 |
| 0 | 0.462 | 0.908 | 1.392 | 1.77 | 2.619 |
| 0 | 7.5 | 15 | 30 | 60 | 120 |

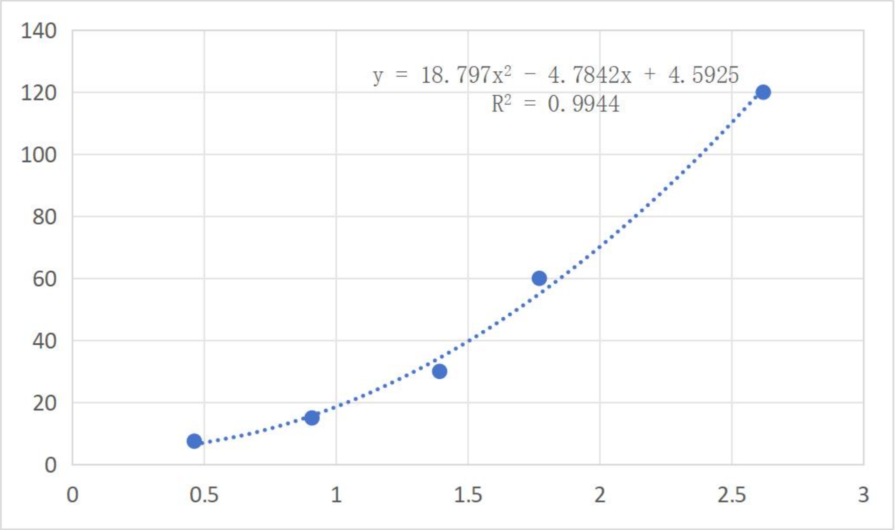


| Sample ID | OD (450nm) | Blank Corrected | Dilution Factor | Concentration |
|---|---|---|---|---|
| 7 | 0.811 | 0.745 | 11.46107593 | 57.30537963 |
| 8 | 0.776 | 0.71 | 10.6712857 | 53.3564285 |
| 9 | 0.752 | 0.686 | 10.15633181 | 50.78165906 |
| 10 | 0.77 | 0.704 | 10.54051715 | 52.70258576 |
| 11 | 0.784 | 0.718 | 10.84774903 | 54.23874514 |
| 12 | 0.826 | 0.76 | 11.8136552 | 59.068276 |
| 19 | 0.796 | 0.73 | 11.1169553 | 55.5847765 |
| 20 | 0.785 | 0.719 | 10.86997612 | 54.34988059 |
| 21 | 0.903 | 0.837 | 13.75672009 | 68.78360047 |
| 22 | 0.772 | 0.706 | 10.58395629 | 52.91978146 |
| 23 | 0.798 | 0.732 | 11.16234933 | 55.81174664 |
| 24 | 0.762 | 0.696 | 10.36826435 | 51.84132176 |
| 25 | 0.803 | 0.737 | 11.27649229 | 56.38246147 |
| 26 | 0.760 | 0.694 | 10.32557709 | 51.62788546 |
| 27 | 0.852 | 0.786 | 12.44483021 | 62.22415106 |
| 28 | 0.832 | 0.766 | 11.95705533 | 59.78527666 |
| 29 | 0.879 | 0.813 | 13.12717969 | 65.63589847 |
| 30 | 0.850 | 0.784 | 12.39537603 | 61.97688016 |
| 31 | 0.746 | 0.68 | 10.0309768 | 50.154884 |
| 32 | 0.818 | 0.752 | 11.62456029 | 58.12280144 |
| 33 | 0.844 | 0.778 | 12.24791575 | 61.23957874 |
| 34 | 0.814 | 0.748 | 11.53091509 | 57.65457544 |
| 35 | 0.870 | 0.804 | 12.89668475 | 64.48342376 |
| 36 | 0.826 | 0.76 | 11.8136552 | 59.068276 |
| 37 | 0.835 | 0.769 | 12.02926292 | 60.14631459 |
| 38 | 0.753 | 0.687 | 10.17735589 | 50.88677947 |
| 39 | 0.860 | 0.794 | 12.64415069 | 63.22075346 |
| 40 | 0.844 | 0.778 | 12.24791575 | 61.23957874 |
| 41 | 0.777 | 0.711 | 10.69321204 | 53.46606019 |
| 42 | 0.836 | 0.77 | 12.0534073 | 60.2670365 |
| 43 | 0.879 | 0.813 | 13.12717969 | 65.63589847 |
| 44 | 0.802 | 0.736 | 11.25358851 | 56.26794256 |

| | | | | |
|---|---|---|---|---|
| 45 | 0.816 | 0.75 | 11.5776625 | 57.8883125 |
| 46 | 0.774 | 0.708 | 10.62754581 | 53.13772904 |
| 47 | 0.862 | 0.796 | 12.69435675 | 63.47178376 |
| 48 | 0.856 | 0.79 | 12.5441897 | 62.7209485 |
| 49 | 0.796 | 0.73 | 11.1169553 | 55.5847765 |
| 50 | 0.770 | 0.704 | 10.54051715 | 52.70258576 |
| 51 | 0.806 | 0.74 | 11.3454292 | 56.727146 |
| 52 | 0.896 | 0.83 | 13.5708673 | 67.8543365 |
| 53 | 0.887 | 0.821 | 13.33462048 | 66.67310239 |
| 54 | 0.912 | 0.846 | 13.99838045 | 69.99190226 |
| 55 | 0.774 | 0.708 | 10.62754581 | 53.13772904 |
| 56 | 0.764 | 0.698 | 10.41110199 | 52.05550994 |
| 57 | 0.783 | 0.717 | 10.82555953 | 54.12779767 |
| 58 | 0.732 | 0.666 | 9.743744932 | 48.71872466 |
| 59 | 0.856 | 0.79 | 12.5441897 | 62.7209485 |
| 60 | 0.784 | 0.718 | 10.84774903 | 54.23874514 |

| | | | | | |
|---|---|---|---|---|---|
| 0.06 | 0.527 | 0.97 | 1.456 | 1.842 | 2.68 |
| 0 | 0.467 | 0.91 | 1.396 | 1.782 | 2.62 |
| 0 | 7.5 | 15 | 30 | 60 | 120 |

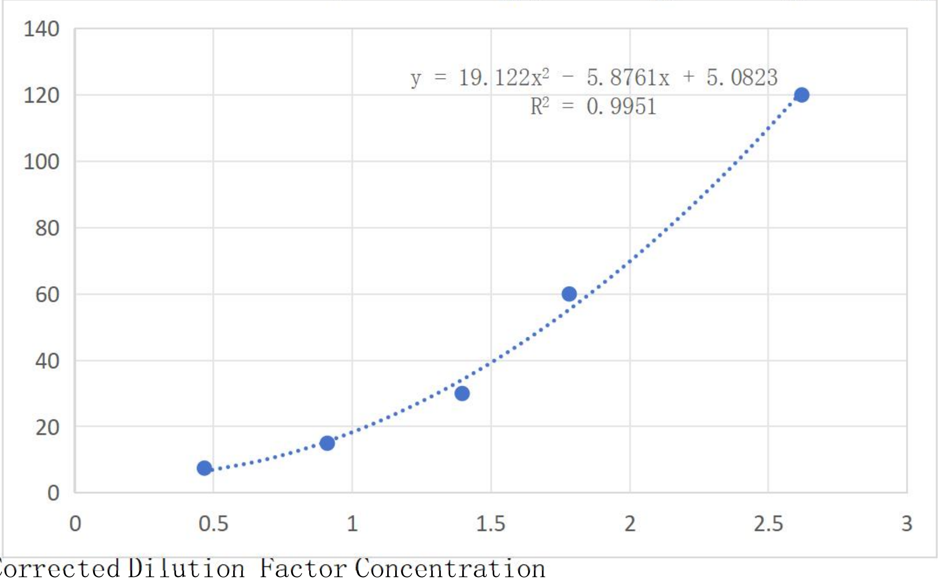


| Blank Corrected | Dilution Factor | Concentration |
|---|---|---|
| 0.751 | 11.45417602 | 57.27088011 |
| 0.716 | 10.67802043 | 53.39010216 |
| 0.692 | 10.17287621 | 50.86438104 |
| 0.71 | 10.5496692 | 52.748346 |
| 0.724 | 10.85129707 | 54.25648536 |
| 0.766 | 11.80115563 | 59.00577816 |
| 0.736 | 11.11580131 | 55.57900656 |
| 0.725 | 10.87312875 | 54.36564375 |
| 0.843 | 13.71777788 | 68.58888939 |
| 0.712 | 10.59229997 | 52.96149984 |
| 0.738 | 11.16042077 | 55.80210384 |
| 0.702 | 10.38067589 | 51.90337944 |
| 0.743 | 11.27263868 | 56.36319339 |
| 0.7 | 10.33881 | 51.69405 |
| 0.792 | 12.42297101 | 62.11485504 |
| 0.772 | 11.94235685 | 59.71178424 |
| 0.819 | 13.09606594 | 65.48032971 |
| 0.79 | 12.3742212 | 61.871106 |
| 0.686 | 10.05003211 | 50.25016056 |
| 0.758 | 11.61502901 | 58.07514504 |
| 0.784 | 12.22888963 | 61.14444816 |
| 0.754 | 11.52288355 | 57.61441776 |
| 0.81 | 12.8686032 | 64.343016 |
| 0.766 | 11.80115563 | 59.00577816 |
| 0.775 | 12.01347375 | 60.06736875 |
| 0.693 | 10.19348408 | 50.96742039 |
| 0.8 | 12.6195 | 63.0975 |
| 0.784 | 12.22888963 | 61.14444816 |
| 0.717 | 10.69954616 | 53.49773079 |
| 0.776 | 12.03725587 | 60.18627936 |
| 0.819 | 13.09606594 | 65.48032971 |
| 0.742 | 11.25011861 | 56.25059304 |

| | | |
|---|---|---|
| 0.756 | 11.56887979 | 57.84439896 |
| 0.714 | 10.63508371 | 53.17541856 |
| 0.802 | 12.66901469 | 63.34507344 |
| 0.796 | 12.52092955 | 62.60464776 |
| 0.736 | 11.11580131 | 55.57900656 |
| 0.71 | 10.5496692 | 52.748346 |
| 0.746 | 11.34042835 | 56.70214176 |
| 0.836 | 13.53416971 | 67.67084856 |
| 0.827 | 13.30085564 | 66.50427819 |
| 0.852 | 13.95659909 | 69.78299544 |
| 0.714 | 10.63508371 | 53.17541856 |
| 0.704 | 10.42269475 | 52.11347376 |
| 0.723 | 10.82950364 | 54.14751819 |
| 0.672 | 9.768750048 | 48.84375024 |
| 0.796 | 12.52092955 | 62.60464776 |
| 0.724 | 10.85129707 | 54.25648536 |

| | | | | | |
|---|---|---|---|---|---|
| 0.068 | 0.522 | 0.962 | 1.453 | 1.828 | 2.689 |
| 0 | 0.454 | 0.894 | 1.385 | 1.76 | 2.621 |
| 0 | 7.5 | 15 | 30 | 60 | 120 |

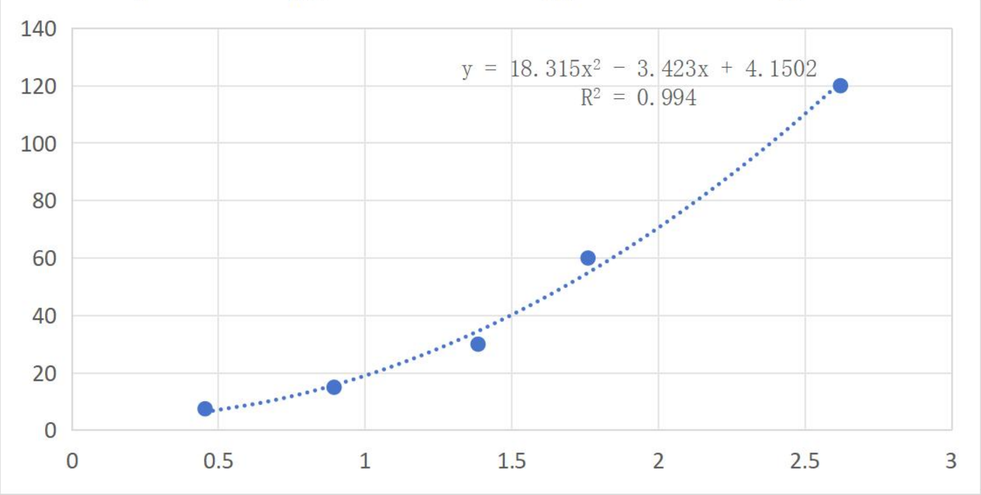


| Sample ID | OD (450nm) | Blank Corrected | Dilution Factor | Concentration |
|---|---|---|---|---|
| 7 | 0.802 | 0.734 | 11.50503414 | 57.5251707 |
| 8 | 0.771 | 0.703 | 10.79526884 | 53.97634418 |
| 9 | 0.764 | 0.696 | 10.63987104 | 53.1993552 |
| 10 | 0.774 | 0.706 | 10.86241734 | 54.3120867 |
| 11 | 0.786 | 0.718 | 11.13430806 | 55.6715403 |
| 12 | 0.830 | 0.762 | 12.17636886 | 60.8818443 |
| 19 | 0.804 | 0.736 | 11.55203424 | 57.7601712 |
| 20 | 0.772 | 0.704 | 10.81761504 | 54.0880752 |
| 21 | 0.892 | 0.824 | 13.76509344 | 68.8254672 |
| 22 | 0.764 | 0.696 | 10.63987104 | 53.1993552 |
| 23 | 0.792 | 0.724 | 11.27223144 | 56.3611572 |
| 24 | 0.755 | 0.687 | 10.44271124 | 52.21355618 |
| 25 | 0.811 | 0.743 | 11.71768844 | 58.58844218 |
| 26 | 0.764 | 0.696 | 10.63987104 | 53.1993552 |
| 27 | 0.860 | 0.792 | 12.92752416 | 64.6376208 |
| 28 | 0.844 | 0.776 | 12.52280544 | 62.6140272 |
| 29 | 0.886 | 0.818 | 13.60519206 | 68.0259603 |
| 30 | 0.853 | 0.785 | 12.74930588 | 63.74652938 |
| 31 | 0.750 | 0.682 | 10.33446006 | 51.6723003 |
| 32 | 0.822 | 0.754 | 11.98162854 | 59.9081427 |
| 33 | 0.851 | 0.783 | 12.69871604 | 63.49358018 |
| 34 | 0.820 | 0.752 | 11.93330976 | 59.6665488 |
| 35 | 0.888 | 0.82 | 13.658346 | 68.29173 |
| 36 | 0.844 | 0.776 | 12.52280544 | 62.6140272 |
| 37 | 0.840 | 0.772 | 12.42309096 | 62.1154548 |
| 38 | 0.746 | 0.678 | 10.24851846 | 51.2425923 |
| 39 | 0.866 | 0.798 | 13.08171126 | 65.4085563 |
| 40 | 0.852 | 0.784 | 12.72399264 | 63.6199632 |
| 41 | 0.784 | 0.716 | 11.08862664 | 55.4431332 |
| 42 | 0.841 | 0.773 | 12.44796464 | 62.23982318 |
| 43 | 0.882 | 0.814 | 13.49932374 | 67.4966187 |
| 44 | 0.810 | 0.742 | 11.69391366 | 58.4695683 |
| 45 | 0.829 | 0.761 | 12.15189812 | 60.75949058 |

| | | | | |
|---|---|---|---|---|
| 46 | 0.772 | 0.704 | 10.81761504 | 54.0880752 |
| 47 | 0.866 | 0.798 | 13.08171126 | 65.4085563 |
| 48 | 0.862 | 0.794 | 12.97877334 | 64.8938667 |
| 49 | 0.801 | 0.733 | 11.48158904 | 57.40794518 |
| 50 | 0.772 | 0.704 | 10.81761504 | 54.0880752 |
| 51 | 0.820 | 0.752 | 11.93330976 | 59.6665488 |
| 52 | 0.911 | 0.843 | 14.28014744 | 71.40073718 |
| 53 | 0.882 | 0.814 | 13.49932374 | 67.4966187 |
| 54 | 0.929 | 0.861 | 14.78029112 | 73.90145558 |
| 55 | 0.745 | 0.677 | 10.22712464 | 51.13562318 |
| 56 | 0.760 | 0.692 | 10.55187816 | 52.7593908 |
| 57 | 0.799 | 0.731 | 11.43480872 | 57.17404358 |
| 58 | 0.729 | 0.661 | 9.889805115 | 49.44902558 |
| 59 | 0.877 | 0.809 | 13.36781252 | 66.83906258 |
| 60 | 0.782 | 0.714 | 11.04309174 | 55.2154587 |

| | | | | | |
|---|---|---|---|---|---|
| 0.071 | 0.531 | 0.978 | 1.452 | 1.84 | 2.67 |
| 0 | 0.46 | 0.907 | 1.381 | 1.769 | 2.599 |
| 0 | 7.5 | 15 | 30 | 60 | 120 |

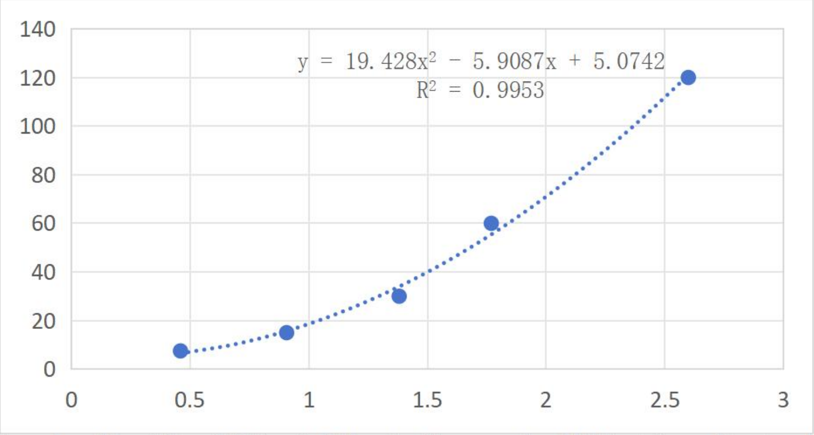


| Sample ID | OD (450nm) | Blank Corrected | Dilution Facto | Concentration |
|---|---|---|---|---|
| 7 | 0.802 | 0.731 | 11.13650581 | 55.68252904 |
| 8 | 0.771 | 0.7 | 10.45783 | 52.28915 |
| 9 | 0.764 | 0.693 | 10.30974847 | 51.54874236 |
| 10 | 0.774 | 0.703 | 10.52187635 | 52.60938176 |
| 11 | 0.786 | 0.715 | 10.7815588 | 53.907794 |
| 12 | 0.830 | 0.759 | 11.78159837 | 58.90799184 |
| 19 | 0.804 | 0.733 | 11.18157359 | 55.90786796 |
| 20 | 0.772 | 0.701 | 10.47913993 | 52.39569964 |
| 21 | 0.892 | 0.821 | 13.31842585 | 66.59212924 |
| 22 | 0.764 | 0.693 | 10.30974847 | 51.54874236 |
| 23 | 0.792 | 0.721 | 10.91349825 | 54.56749124 |
| 24 | 0.755 | 0.684 | 10.12215557 | 50.61077784 |
| 25 | 0.811 | 0.74 | 11.3405348 | 56.702674 |
| 26 | 0.764 | 0.693 | 10.30974847 | 51.54874236 |
| 27 | 0.860 | 0.789 | 12.50657369 | 62.53286844 |
| 28 | 0.844 | 0.773 | 12.11556831 | 60.57784156 |
| 29 | 0.886 | 0.815 | 13.1631728 | 65.815864 |
| 30 | 0.853 | 0.782 | 12.33428487 | 61.67142436 |

| | | | | |
|---|---|---|---|---|
| 31 | 0.750 | 0.679 | 10.01929725 | 50.09648624 |
| 32 | 0.822 | 0.751 | 11.59417773 | 57.97088864 |
| 33 | 0.851 | 0.78 | 12.2854092 | 61.427046 |
| 34 | 0.820 | 0.749 | 11.54771113 | 57.73855564 |
| 35 | 0.888 | 0.817 | 13.21476839 | 66.07384196 |
| 36 | 0.844 | 0.773 | 12.11556831 | 60.57784156 |
| 37 | 0.840 | 0.769 | 12.01937121 | 60.09685604 |
| 38 | 0.746 | 0.675 | 9.93771 | 49.68855 |
| 39 | 0.866 | 0.795 | 12.6557652 | 63.278826 |
| 40 | 0.852 | 0.781 | 12.30982761 | 61.54913804 |
| 41 | 0.784 | 0.713 | 10.73788983 | 53.68944916 |
| 42 | 0.841 | 0.77 | 12.0433622 | 60.216811 |
| 43 | 0.882 | 0.811 | 13.06044789 | 65.30223944 |
| 44 | 0.810 | 0.739 | 11.31770949 | 56.58854744 |
| 45 | 0.829 | 0.758 | 11.75803479 | 58.79017396 |
| 46 | 0.772 | 0.701 | 10.47913993 | 52.39569964 |
| 47 | 0.866 | 0.795 | 12.6557652 | 63.278826 |
| 48 | 0.862 | 0.791 | 12.55614877 | 62.78074384 |
| 49 | 0.801 | 0.73 | 11.1140302 | 55.570151 |
| 50 | 0.772 | 0.701 | 10.47913993 | 52.39569964 |
| 51 | 0.820 | 0.749 | 11.54771113 | 57.73855564 |
| 52 | 0.911 | 0.84 | 13.8192888 | 69.096444 |
| 53 | 0.882 | 0.811 | 13.06044789 | 65.30223944 |
| 54 | 0.929 | 0.858 | 14.30672959 | 71.53364796 |
| 55 | 0.745 | 0.674 | 9.917410328 | 49.58705164 |
| 56 | 0.760 | 0.689 | 10.22598529 | 51.12992644 |
| 57 | 0.799 | 0.728 | 11.06919555 | 55.34597776 |
| 58 | 0.729 | 0.658 | 9.597899992 | 47.98949996 |
| 59 | 0.877 | 0.806 | 12.93291601 | 64.66458004 |
| 60 | 0.782 | 0.711 | 10.69437629 | 53.47188144 |

| | | |
|---|---|---|
| 57.53 | 55.68 | 56.605 |
| 53.98 | 52.29 | 53.135 |
| 53.2 | 51.55 | 52.375 |
| 54.31 | 52.61 | 53.46 |
| 55.67 | 53.91 | 54.79 |
| 60.88 | 58.91 | 59.895 |
| 57.76 | 55.91 | 56.835 |
| 54.09 | 52.4 | 53.245 |
| 68.83 | 66.59 | 67.71 |
| 53.2 | 51.55 | 52.375 |
| 56.36 | 54.57 | 55.465 |
| 52.21 | 50.61 | 51.41 |
| 58.59 | 56.7 | 57.645 |
| 53.2 | 51.55 | 52.375 |
| 64.64 | 62.53 | 63.585 |
| 62.61 | 60.58 | 61.595 |
| 68.03 | 65.82 | 66.925 |
| 63.75 | 61.67 | 62.71 |
| 51.67 | 50.1 | 50.885 |
| 59.91 | 57.97 | 58.94 |
| 63.49 | 61.43 | 62.46 |
| 59.67 | 57.74 | 58.705 |
| 68.29 | 66.07 | 67.18 |
| 62.61 | 60.58 | 61.595 |
| 62.12 | 60.1 | 61.11 |
| 51.24 | 49.69 | 50.465 |
| 65.41 | 63.28 | 64.345 |
| 63.62 | 61.55 | 62.585 |
| 55.44 | 53.69 | 54.565 |
| 62.24 | 60.22 | 61.23 |
| 67.5 | 65.3 | 66.4 |
| 58.47 | 56.59 | 57.53 |
| 60.76 | 58.79 | 59.775 |
| 54.09 | 52.4 | 53.245 |
| 65.41 | 63.28 | 64.345 |
| 64.89 | 62.78 | 63.835 |
| 57.41 | 55.57 | 56.49 |
| 54.09 | 52.4 | 53.245 |
| 59.67 | 57.74 | 58.705 |
| 71.4 | 69.1 | 70.25 |
| 67.5 | 65.3 | 66.4 |
| 73.9 | 71.53 | 72.715 |
| 51.14 | 49.59 | 50.365 |
| 64.64 | 62.53 | 63.585 |
| 69.77 | 67.51 | 68.64 |
| 60.76 | 58.79 | 59.775 |
| 66.84 | 64.66 | 65.75 |
| 67.5 | 65.3 | 66.4 |

| | | | | | |
|---|---|---|---|---|---|
| 57.4374324 | 54.917908 | 56.1776702 | 56.6038499 | 57.5251707 | 55.682529 |
| 54.1769675 | 51.5532578 | 52.86511265 | 53.1327471 | 53.9763442 | 52.28915 |
| 53.412829 | 50.7635843 | 52.08820665 | 52.3740488 | 53.1993552 | 51.5487424 |
| 54.3969264 | 51.7804848 | 53.0887056 | 53.4607343 | 54.3120867 | 52.6093818 |
| 56.2958907 | 53.740732 | 55.01831135 | 54.7896672 | 55.6715403 | 53.907794 |
| 61.0915613 | 58.6805522 | 59.88605675 | 59.8949181 | 60.8818443 | 58.9079918 |
| 57.3224615 | 54.7993874 | 56.06092445 | 56.8340196 | 57.7601712 | 55.907868 |
| 54.6176113 | 52.0084255 | 53.3130184 | 53.2418874 | 54.0880752 | 52.3956996 |
| 68.3967959 | 66.1808918 | 67.28884385 | 67.7087982 | 68.8254672 | 66.5921292 |
| 54.2868562 | 51.6667821 | 52.97681915 | 52.3740488 | 53.1993552 | 51.5487424 |
| 57.0930641 | 54.5628816 | 55.82797285 | 55.4643242 | 56.3611572 | 54.5674912 |
| 55.3957261 | 52.8118389 | 54.1037825 | 51.412167 | 52.2135562 | 50.6107778 |
| 58.7140927 | 56.2334114 | 57.47375205 | 57.6455581 | 58.5884422 | 56.702674 |
| 54.0672603 | 51.439912 | 52.75358615 | 52.3740488 | 53.1993552 | 51.5487424 |
| 64.6678536 | 62.355688 | 63.5117708 | 63.5852446 | 64.6376208 | 62.5328684 |
| 62.9223091 | 60.5627483 | 61.7425287 | 61.5959344 | 62.6140272 | 60.5778416 |
| 67.223202 | 64.9777 | 66.100451 | 66.9209122 | 68.0259603 | 65.815864 |
| 65.9364523 | 63.657772 | 64.79711215 | 62.7089769 | 63.7465294 | 61.6714244 |
| 52.1235694 | 49.4302005 | 50.77688495 | 50.8843933 | 51.6723003 | 50.0964862 |
| 60.1318614 | 57.6931308 | 58.9124961 | 58.9395157 | 59.9081427 | 57.9708886 |
| 63.4174059 | 61.071448 | 62.24442695 | 62.4603131 | 63.4935802 | 61.427046 |
| 58.9485724 | 56.4749136 | 57.711743 | 58.7025522 | 59.6665488 | 57.7385556 |
| 69.8511544 | 67.671088 | 68.7611212 | 67.182786 | 68.29173 | 66.073842 |
| 65.5539669 | 63.2652732 | 64.40962005 | 61.5959344 | 62.6140272 | 60.5778416 |
| 62.0628779 | 59.6793938 | 60.87113585 | 61.1061554 | 62.1154548 | 60.096856 |
| 53.0880637 | 50.4278294 | 51.75794655 | 50.4655712 | 51.2425923 | 49.68855 |
| 66.9644 | 64.7122869 | 65.83834345 | 64.3436912 | 65.4085563 | 63.278826 |
| 64.2908135 | 61.9685424 | 63.12967795 | 62.5845506 | 63.6199632 | 61.549138 |
| 54.6176113 | 52.0084255 | 53.3130184 | 54.5662912 | 55.4431332 | 53.6894492 |
| 62.4301165 | 60.0569037 | 61.2435101 | 61.2283171 | 62.2398232 | 60.216811 |
| 71.0574201 | 68.906399 | 69.98190955 | 66.3994291 | 67.4966187 | 65.3022394 |
| 60.9709635 | 58.5565 | 59.76373175 | 57.5290579 | 58.4695683 | 56.5885474 |
| 59.537948 | 57.0817917 | 58.30986985 | 59.7748323 | 60.7594906 | 58.790174 |
| 54.8390223 | 52.23708 | 53.53805115 | 53.2418874 | 54.0880752 | 52.3956996 |
| 67.7429842 | 65.5106675 | 66.62682585 | 64.3436912 | 65.4085563 | 63.278826 |
| 66.8352713 | 64.579848 | 65.70755965 | 63.8373053 | 64.8938667 | 62.7807438 |
| 60.4903875 | 58.0620755 | 59.2762315 | 56.4890481 | 57.4079452 | 55.570151 |
| 53.9577347 | 51.3267446 | 52.64223965 | 53.2418874 | 54.0880752 | 52.3956996 |
| 59.4197099 | 56.9600592 | 58.18988455 | 58.7025522 | 59.6665488 | 57.7385556 |
| 72.8257597 | 70.7162541 | 71.7710069 | 70.2485906 | 71.4007372 | 69.096444 |
| 67.7429842 | 65.5106675 | 66.62682585 | 66.3994291 | 67.4966187 | 65.3022394 |
| 71.0574201 | 68.906399 | 69.98190955 | 72.7175518 | 73.9014556 | 71.533648 |
| 51.3835717 | 48.6642595 | 50.0239156 | 50.3613374 | 51.1356232 | 49.5870516 |
| 66.0643105 | 63.7889618 | 64.92663615 | 63.5852446 | 64.6376208 | 62.5328684 |
| 70.1179429 | 67.9443525 | 69.0311477 | 68.6365852 | 69.7665586 | 67.5066118 |
| 62.6758498 | 60.3094691 | 61.49265945 | 59.7748323 | 60.7594906 | 58.790174 |
| 66.3205714 | 64.0518768 | 65.1862241 | 65.7518213 | 66.8390626 | 64.66458 |

| | | | | | |
|---|---|---|---|---|---|
| 64.416312 | 62.0974125 | 63.25686225 | 66.3994291 | 67.4966187 | 65.3022394 |

Report
FT4 (14)

| Group | | Mean | Std. Deviation |
|---|---|---|---|
| | 1 | 53.84083867587 | 7.2370747034125 |
| | 2 | 35.92516072773 | 3.6524107274908 |
| | 3 | 38.52778410373 | 3.5558749771251 |
| Total | | 41.50407614938 | 8.8454534735736 |

Tests of Normality

| | Group | Kolmogorov-Smirnova | | | Shapiro-Wilk |
|---|---|---|---|---|---|
| | | Statistic | df | Sig. | Statistic |
| FT4 (14) | 1 | 0.183 | 12 | .200* | 0.914 |
| | 2 | 0.184 | 18 | 0.108 | 0.916 |
| | 3 | 0.178 | 16 | 0.189 | 0.886 |

* This is a lower bound of the true significance.
a Lilliefors Significance Correction

Hypothesis Test Summary

| | Null Hypothesis | Test | Sig. | Decision |
|---|---|---|---|---|
| 1 | The distribution of FT4 (14) is the same across categories of Group. | Independent-Samples Kruskal-Wallis Test | .000 | Reject the null hypothesis. |

Asymptotic significances are displayed. The significance level is .05.

ANOVA
FT4 (14)

| | Sum of Squares | df | Mean Square | F | Sig. |
|---|---|---|---|---|---|
| Between Groups | 2528.319 | 2 | 1264.159 | 54.766 | 0 |
| Within Groups | 992.573 | 43 | 23.083 | | |
| Total | 3520.892 | 45 | | | |

Multiple Comparisons
Dependent Variable: FT4 (14)
Bonferroni

| (I) Group | (J) Group | Mean Difference | Std. Error | Sig. | 95% Confidence |
|---|---|---|---|---|---|
| | | | | | Lower Bound |
| 1 | 2 | 17.91567794805 | 1.7905267022 | 0 | 13.45500867604 |

| | | | | | |
|---|---|---|---|---|---|
| | 3 | 15.31305457208 | 1.83474389626 | 0 | 10.74222873072 |
| 2 | 1 | -17.91567794801 | 1.79052670221 | 0 | -22.37634722 |
| | 3 | -2.602623376 | 1.65078405330 | 0.367 | -6.715157242 |
| 3 | 1 | -15.31305457201 | 1.83474389626 | 0 | -19.88388041 |
| | 2 | 2.60262337597 | 21.65078405330 | 0.367 | -1.50991049 |

* The mean difference is significant at the 0.05 level.

| | |
|---|---|
| 56.60384985 | 56.39076005 |
| 53.1327471 | 52.99892988 |
| 52.3740488 | 52.23112773 |
| 53.46073425 | 53.27471995 |
| 54.78966715 | 54.90398928 |
| 59.89491805 | 59.89048743 |
| 56.8340196 | 56.44747203 |
| 53.2418874 | 53.2774529 |
| 67.7087982 | 67.49882103 |
| 52.3740488 | 52.67543398 |
| 55.4643242 | 55.64614853 |
| 51.412167 | 52.75797475 |
| 57.6455581 | 57.55965508 |
| 52.3740488 | 52.56381748 |
| 63.5852446 | 63.5485077 |
| 61.5959344 | 61.66923155 |
| 66.92091215 | 66.5106816 |
| 62.7089769 | 63.75304453 |
| 50.88439325 | 50.83063913 |
| 58.93951565 | 58.9260059 |
| 62.4603131 | 62.35237003 |
| 58.7025522 | 58.2071476 |
| 67.182786 | 67.9719536 |
| 61.5959344 | 63.00277723 |
| 61.1061554 | 60.98864563 |
| 50.46557115 | 51.11175888 |
| 64.34369115 | 65.09101733 |
| 62.5845506 | 62.85711428 |
| 54.5662912 | 53.9396548 |
| 61.2283171 | 61.2359136 |
| 66.39942905 | 68.19066933 |
| 57.52905785 | 58.64639483 |
| 59.7748323 | 59.04235108 |
| 53.2418874 | 53.38996928 |
| 64.34369115 | 65.48525853 |
| 63.83730525 | 64.77243248 |
| 56.4890481 | 57.8826398 |
| 53.2418874 | 52.94206353 |
| 58.7025522 | 58.44621838 |
| 70.2485906 | 71.00979875 |
| 66.39942905 | 66.51312748 |
| 72.7175518 | 71.34973068 |
| 50.3613374 | 50.1926265 |
| 63.5852446 | 64.25594038 |
| 68.6365852 | 68.83386645 |
| 59.7748323 | 60.63374588 |
| 65.7518213 | 65.4690227 |

66.39942905 64.82814568

| df | Sig. |
| --- | --- |
| 12 | 0.238 |
| 18 | 0.11 |
| 16 | 0.049 |

Interval
Upper Bound
22.376347220068

19.883880413438
-13.45500868
1.50991049
-10.74222873
6.715157242

| | | | | | |
|---|---|---|---|---|---|
| 0.068 | 0.42 | 0.964 | 1.56 | 2.012 | 2.604 |
| 0 | 0.352 | 0.896 | 1.492 | 1.944 | 2.536 |
| 0 | 1.25 | 2.5 | 5 | 10 | 20 |

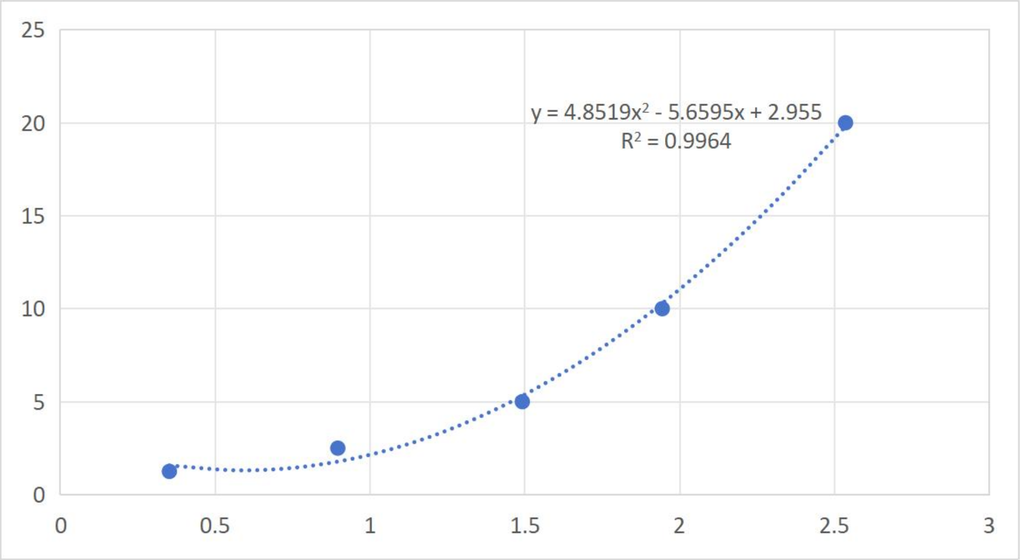


| | | x | | |
|---|---|---|---|---|
| Sample ID | OD (450nm) | Blank Corrected | Dilution Factor | Concentration |
| 7 | 0.88 | 0.812 | 1.558557154 | 7.792785768 |
| 8 | 0.862 | 0.794 | 1.520169428 | 7.600847142 |
| 9 | 0.823 | 0.755 | 1.447781798 | 7.238908988 |
| 10 | 0.95 | 0.882 | 1.737730456 | 8.688652278 |
| 11 | 0.952 | 0.884 | 1.743548366 | 8.717741832 |
| 12 | 0.791 | 0.723 | 1.399410335 | 6.997051676 |
| 19 | 0.97 | 0.902 | 1.797656248 | 8.988281238 |
| 20 | 0.854 | 0.786 | 1.504117412 | 7.520587062 |
| 21 | 0.904 | 0.836 | 1.614631502 | 8.073157512 |
| 22 | 0.98 | 0.912 | 1.829074714 | 9.145373568 |
| 23 | 0.893 | 0.825 | 1.588236938 | 7.941184688 |
| 24 | 0.862 | 0.794 | 1.520169428 | 7.600847142 |
| 25 | 0.923 | 0.855 | 1.662987698 | 8.314938488 |
| 26 | 0.962 | 0.894 | 1.773220148 | 8.866100742 |
| 27 | 0.832 | 0.764 | 1.463176622 | 7.315883112 |
| 28 | 0.9 | 0.832 | 1.604897626 | 8.024488128 |
| 29 | 0.973 | 0.905 | 1.806979898 | 9.034899488 |
| 30 | 0.955 | 0.887 | 1.752348011 | 8.761740056 |
| 31 | 0.941 | 0.873 | 1.712030195 | 8.560150976 |
| 32 | 0.862 | 0.794 | 1.520169428 | 7.600847142 |
| 33 | 0.95 | 0.882 | 1.737730456 | 8.688652278 |
| 34 | 0.932 | 0.864 | 1.687115942 | 8.435579712 |
| 35 | 0.976 | 0.908 | 1.816390882 | 9.081954408 |
| 36 | 0.914 | 0.846 | 1.63964546 | 8.198227302 |
| 37 | 0.967 | 0.899 | 1.788419932 | 8.94209966 |
| 38 | 0.832 | 0.764 | 1.463176622 | 7.315883112 |
| 39 | 0.866 | 0.798 | 1.528428328 | 7.642141638 |
| 40 | 0.944 | 0.876 | 1.720509614 | 8.602548072 |
| 41 | 0.975 | 0.907 | 1.813244183 | 9.066220916 |
| 42 | 0.86 | 0.792 | 1.516098202 | 7.580491008 |
| 43 | 0.834 | 0.766 | 1.466704436 | 7.333522182 |
| 44 | 0.925 | 0.857 | 1.668281603 | 8.341408016 |

| | | | | |
|---|---|---|---|---|
| 45 | 0.929 | 0.861 | 1.67898586 | 8.3949293 |
| 46 | 0.864 | 0.796 | 1.52427947 | 7.621397352 |
| 47 | 0.912 | 0.844 | 1.634565038 | 8.172825192 |
| 48 | 0.941 | 0.873 | 1.712030195 | 8.560150976 |
| 49 | 0.988 | 0.920 | 1.85490816 | 9.2745408 |
| 50 | 0.979 | 0.911 | 1.8258892 | 9.129446 |
| 51 | 0.852 | 0.784 | 1.500201446 | 7.501007232 |
| 52 | 0.948 | 0.880 | 1.73195136 | 8.6597568 |
| 53 | 0.942 | 0.874 | 1.714846964 | 8.574234822 |
| 54 | 0.934 | 0.866 | 1.692584516 | 8.462922582 |
| 55 | 0.858 | 0.790 | 1.51206579 | 7.56032895 |
| 56 | 0.96 | 0.892 | 1.767208162 | 8.836040808 |
| 57 | 0.833 | 0.765 | 1.464935678 | 7.324678388 |
| 58 | 0.884 | 0.816 | 1.567514726 | 7.837573632 |
| 59 | 0.938 | 0.870 | 1.70363811 | 8.51819055 |
| 60 | 0.924 | 0.856 | 1.665629798 | 8.328148992 |

| | | | | | |
|---|---|---|---|---|---|
| 0.066 | 0.423 | 0.932 | 1.542 | 2.013 | 2.598 |
| 0 | 0.357 | 0.866 | 1.476 | 1.947 | 2.532 |
| 0 | 1.25 | 2.5 | 5 | 10 | 20 |

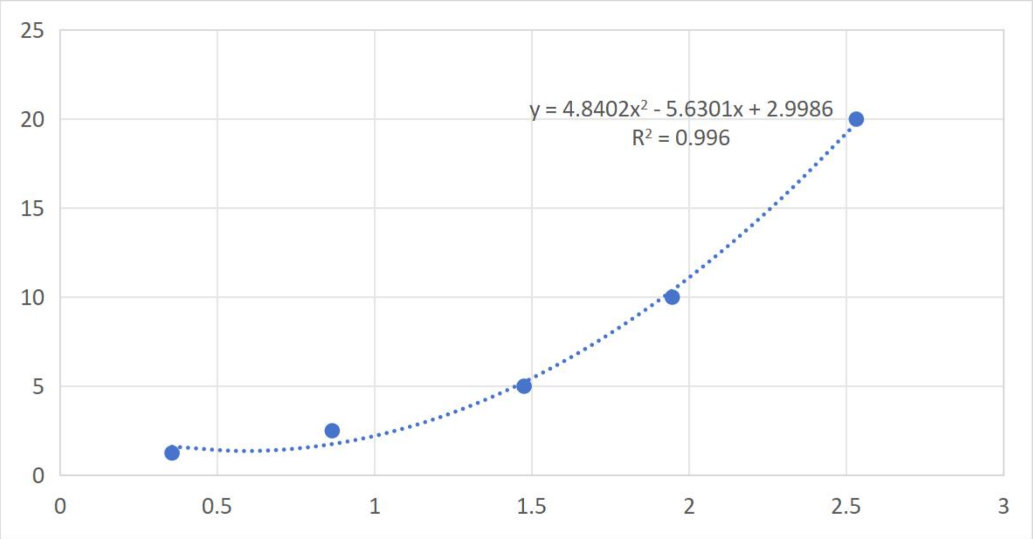


| Sample ID | OD (450nm) | Blank Corrected | Dilution Factor | Concentration |
|---|---|---|---|---|
| 7 | 0.88 | 0.814 | 1.622795759 | 8.113978796 |
| 8 | 0.862 | 0.796 | 1.583868563 | 7.919342816 |
| 9 | 0.823 | 0.757 | 1.51028607 | 7.551430349 |
| 10 | 0.95 | 0.884 | 1.803994931 | 9.019974656 |
| 11 | 0.952 | 0.886 | 1.809869039 | 9.049345196 |
| 12 | 0.791 | 0.725 | 1.460907625 | 7.304538125 |
| 19 | 0.97 | 0.904 | 1.864478483 | 9.322392416 |
| 20 | 0.854 | 0.788 | 1.567574349 | 7.837871744 |
| 21 | 0.904 | 0.838 | 1.679577609 | 8.397888044 |
| 22 | 0.98 | 0.914 | 1.896172319 | 9.480861596 |
| 23 | 0.893 | 0.827 | 1.652860446 | 8.264302229 |
| 24 | 0.862 | 0.796 | 1.583868563 | 7.919342816 |
| 25 | 0.923 | 0.857 | 1.72848435 | 8.642421749 |
| 26 | 0.962 | 0.896 | 1.839820403 | 9.199102016 |

| | | | | |
|---|---|---|---|---|
| 27 | 0.832 | 0.766 | 1.525959791 | 7.629798956 |
| 28 | 0.9 | 0.834 | 1.669726751 | 8.348633756 |
| 29 | 0.973 | 0.907 | 1.87388499 | 9.369424949 |
| 30 | 0.955 | 0.889 | 1.818752804 | 9.093764021 |
| 31 | 0.941 | 0.875 | 1.778040625 | 8.890203125 |
| 32 | 0.862 | 0.796 | 1.583868563 | 7.919342816 |
| 33 | 0.95 | 0.884 | 1.803994931 | 9.019974656 |
| 34 | 0.932 | 0.866 | 1.752870431 | 8.764352156 |
| 35 | 0.976 | 0.91 | 1.88337862 | 9.4168931 |
| 36 | 0.914 | 0.848 | 1.704882381 | 8.524411904 |
| 37 | 0.967 | 0.901 | 1.8551591 | 9.275795501 |
| 38 | 0.832 | 0.766 | 1.525959791 | 7.629798956 |
| 39 | 0.866 | 0.8 | 1.592248 | 7.96124 |
| 40 | 0.944 | 0.878 | 1.786604937 | 8.933024684 |
| 41 | 0.975 | 0.909 | 1.880204396 | 9.401021981 |
| 42 | 0.86 | 0.794 | 1.579736927 | 7.898684636 |
| 43 | 0.834 | 0.768 | 1.529549325 | 7.647746624 |
| 44 | 0.925 | 0.859 | 1.733835716 | 8.669178581 |
| 45 | 0.929 | 0.863 | 1.744654614 | 8.723273069 |
| 46 | 0.864 | 0.798 | 1.588038921 | 7.940194604 |
| 47 | 0.912 | 0.846 | 1.699743983 | 8.498719916 |
| 48 | 0.941 | 0.875 | 1.778040625 | 8.890203125 |
| 49 | 0.988 | 0.922 | 1.922224377 | 9.611121884 |
| 50 | 0.979 | 0.913 | 1.892959374 | 9.464796869 |
| 51 | 0.852 | 0.786 | 1.563597599 | 7.817987996 |
| 52 | 0.948 | 0.882 | 1.798159545 | 8.990797724 |
| 53 | 0.942 | 0.876 | 1.780885715 | 8.904428576 |
| 54 | 0.934 | 0.868 | 1.758396045 | 8.791980224 |
| 55 | 0.858 | 0.792 | 1.575644013 | 7.878220064 |
| 56 | 0.96 | 0.894 | 1.833752687 | 9.168763436 |
| 57 | 0.833 | 0.767 | 1.527749718 | 7.638748589 |
| 58 | 0.884 | 0.818 | 1.631872185 | 8.159360924 |
| 59 | 0.938 | 0.872 | 1.769563437 | 8.847817184 |
| 60 | 0.924 | 0.858 | 1.731155193 | 8.655775964 |

| | | | | | |
|---|---|---|---|---|---|
| 0.07 | 0.424 | 0.942 | 1.566 | 2.016 | 2.608 |
| 0 | 0.354 | 0.872 | 1.496 | 1.946 | 2.538 |
| 0 | 1.25 | 2.5 | 5 | 10 | 20 |

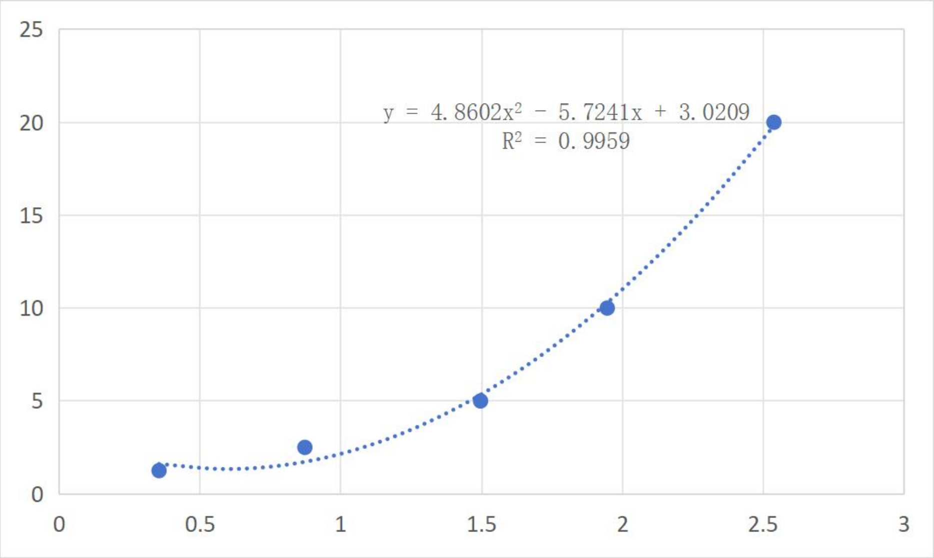


| Sample ID | OD (450nm) | Blank Corrected | Dilution Factor | Concentration |
|---|---|---|---|---|
| 7 | 0.872 | 0.802 | 1.556271881 | 7.781359404 |
| 8 | 0.86 | 0.79 | 1.53211182 | 7.6605591 |
| 9 | 0.822 | 0.752 | 1.464839341 | 7.324196704 |
| 10 | 0.931 | 0.861 | 1.695418224 | 8.477091121 |
| 11 | 0.944 | 0.874 | 1.730626735 | 8.653133676 |
| 12 | 0.801 | 0.731 | 1.433684232 | 7.168421161 |
| 19 | 0.966 | 0.896 | 1.793952723 | 8.969763616 |
| 20 | 0.832 | 0.762 | 1.481181769 | 7.405908844 |
| 21 | 0.912 | 0.842 | 1.646914633 | 8.234573164 |
| 22 | 0.982 | 0.912 | 1.842962989 | 9.214814944 |
| 23 | 0.906 | 0.836 | 1.632326739 | 8.161633696 |
| 24 | 0.881 | 0.811 | 1.575310504 | 7.876552521 |
| 25 | 0.927 | 0.857 | 1.68491533 | 8.424576649 |
| 26 | 0.918 | 0.848 | 1.661852461 | 8.309262304 |
| 27 | 0.820 | 0.75 | 1.4616875 | 7.3084375 |
| 28 | 0.947 | 0.877 | 1.738985066 | 8.694925329 |
| 29 | 0.966 | 0.896 | 1.793952723 | 8.969763616 |
| 30 | 0.942 | 0.872 | 1.725103117 | 8.625515584 |
| 31 | 0.902 | 0.832 | 1.622795885 | 8.113979424 |
| 32 | 0.856 | 0.786 | 1.524369519 | 7.621847596 |
| 33 | 0.933 | 0.863 | 1.700727994 | 8.503639969 |
| 34 | 0.905 | 0.835 | 1.629929445 | 8.149647225 |
| 35 | 0.964 | 0.894 | 1.788001407 | 8.940007036 |
| 36 | 0.920 | 0.85 | 1.6669095 | 8.3345475 |
| 37 | 0.951 | 0.881 | 1.750265592 | 8.751327961 |
| 38 | 0.823 | 0.753 | 1.466429842 | 7.332149209 |
| 39 | 0.851 | 0.781 | 1.514910352 | 7.574551761 |
| 40 | 0.930 | 0.86 | 1.69277792 | 8.4638896 |
| 41 | 0.960 | 0.89 | 1.77621542 | 8.8810771 |
| 42 | 0.872 | 0.802 | 1.556271881 | 7.781359404 |

| | | | | |
|---|---|---|---|---|
| 43 | 0.822 | 0.752 | 1.464839341 | 7.324196704 |
| 44 | 0.917 | 0.847 | 1.659338522 | 8.296692609 |
| 45 | 0.920 | 0.85 | 1.6669095 | 8.3345475 |
| 46 | 0.856 | 0.786 | 1.524369519 | 7.621847596 |
| 47 | 0.923 | 0.853 | 1.674567962 | 8.372839809 |
| 48 | 0.935 | 0.865 | 1.706076645 | 8.530383225 |
| 49 | 0.968 | 0.898 | 1.799942921 | 8.999714604 |
| 50 | 0.957 | 0.887 | 1.767477994 | 8.837389969 |
| 51 | 0.881 | 0.811 | 1.575310504 | 7.876552521 |
| 52 | 0.944 | 0.874 | 1.730626735 | 8.653133676 |
| 53 | 0.938 | 0.868 | 1.714172525 | 8.570862624 |
| 54 | 0.936 | 0.866 | 1.708765551 | 8.543827756 |
| 55 | 0.860 | 0.79 | 1.53211182 | 7.6605591 |
| 56 | 0.953 | 0.883 | 1.755964178 | 8.779820889 |
| 57 | 0.846 | 0.776 | 1.505694195 | 7.528470976 |
| 58 | 0.877 | 0.807 | 1.56675169 | 7.833758449 |
| 59 | 0.922 | 0.852 | 1.672005421 | 8.360027104 |
| 60 | 0.931 | 0.861 | 1.695418224 | 8.477091121 |

| | | | | | |
|---|---|---|---|---|---|
| 0.068 | 0.428 | 0.936 | 1.562 | 2.015 | 2.604 |
| 0 | 0.36 | 0.868 | 1.494 | 1.947 | 2.536 |
| 0 | 1.25 | 2.5 | 5 | 10 | 20 |

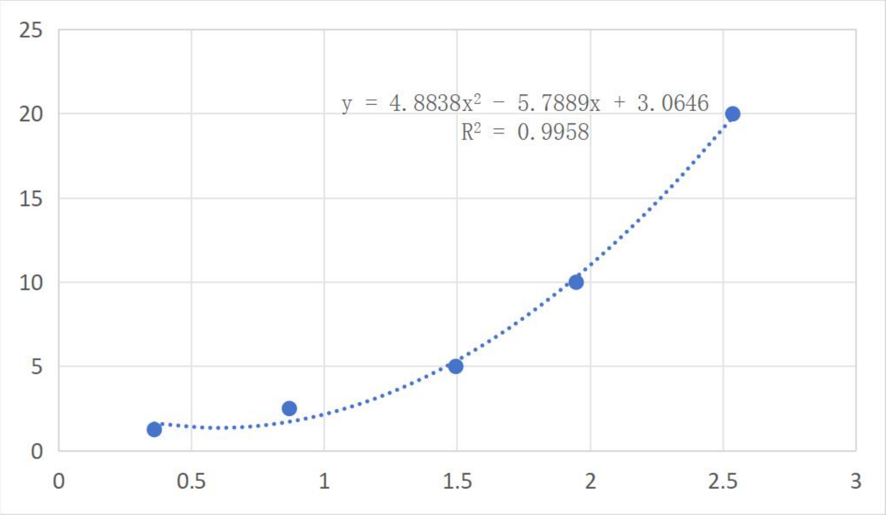


| Sample ID | OD (450nm) | Blank Corrected | Dilution Factor | Concentration |
|---|---|---|---|---|
| 7 | 0.872 | 0.804 | 1.567290861 | 7.836454304 |
| 8 | 0.86 | 0.792 | 1.543223123 | 7.716115616 |
| 9 | 0.822 | 0.754 | 1.476287841 | 7.381439204 |
| 10 | 0.931 | 0.863 | 1.706082142 | 8.530410711 |
| 11 | 0.944 | 0.876 | 1.741234509 | 8.706172544 |
| 12 | 0.801 | 0.733 | 1.445348318 | 7.226741591 |
| 19 | 0.966 | 0.898 | 1.804483655 | 9.022418276 |
| 20 | 0.832 | 0.764 | 1.492534925 | 7.462674624 |
| 21 | 0.912 | 0.844 | 1.657674957 | 8.288374784 |
| 22 | 0.982 | 0.914 | 1.853452385 | 9.267261924 |

| | | | | |
|---|---|---|---|---|
| 23 | 0.906 | 0.838 | 1.643121047 | 8.215605236 |
| 24 | 0.881 | 0.813 | 1.586264702 | 7.931323511 |
| 25 | 0.927 | 0.859 | 1.695598128 | 8.477990639 |
| 26 | 0.918 | 0.85 | 1.6725805 | 8.3629025 |
| 27 | 0.820 | 0.752 | 1.473155635 | 7.365778176 |
| 28 | 0.947 | 0.879 | 1.749581016 | 8.747905079 |
| 29 | 0.966 | 0.898 | 1.804483655 | 9.022418276 |
| 30 | 0.942 | 0.874 | 1.735719009 | 8.678595044 |
| 31 | 0.902 | 0.834 | 1.633613793 | 8.168068964 |
| 32 | 0.856 | 0.788 | 1.535513107 | 7.677565536 |
| 33 | 0.933 | 0.865 | 1.711382755 | 8.556913775 |
| 34 | 0.905 | 0.837 | 1.640729582 | 8.203647911 |
| 35 | 0.964 | 0.896 | 1.798538381 | 8.992691904 |
| 36 | 0.920 | 0.852 | 1.677627155 | 8.388135776 |
| 37 | 0.951 | 0.883 | 1.760846438 | 8.804232191 |
| 38 | 0.823 | 0.755 | 1.477868595 | 7.389342975 |
| 39 | 0.851 | 0.783 | 1.526095358 | 7.630476791 |
| 40 | 0.930 | 0.862 | 1.703446487 | 8.517232436 |
| 41 | 0.960 | 0.892 | 1.786765043 | 8.933825216 |
| 42 | 0.872 | 0.804 | 1.567290861 | 7.836454304 |
| 43 | 0.822 | 0.754 | 1.476287841 | 7.381439204 |
| 44 | 0.917 | 0.849 | 1.670071824 | 8.350359119 |
| 45 | 0.920 | 0.852 | 1.677627155 | 8.388135776 |
| 46 | 0.856 | 0.788 | 1.535513107 | 7.677565536 |
| 47 | 0.923 | 0.855 | 1.685270395 | 8.426351975 |
| 48 | 0.935 | 0.867 | 1.716722438 | 8.583612191 |
| 49 | 0.968 | 0.9 | 1.810468 | 9.05234 |
| 50 | 0.957 | 0.889 | 1.7780376 | 8.890187999 |
| 51 | 0.881 | 0.813 | 1.586264702 | 7.931323511 |
| 52 | 0.944 | 0.876 | 1.741234509 | 8.706172544 |
| 53 | 0.938 | 0.87 | 1.72480522 | 8.6240261 |
| 54 | 0.936 | 0.868 | 1.719406931 | 8.597034656 |
| 55 | 0.860 | 0.792 | 1.543223123 | 7.716115616 |
| 56 | 0.953 | 0.885 | 1.766537755 | 8.832688775 |
| 57 | 0.846 | 0.778 | 1.516921799 | 7.584608996 |
| 58 | 0.877 | 0.809 | 1.577734208 | 7.888671039 |
| 59 | 0.922 | 0.854 | 1.682712881 | 8.413564404 |
| 60 | 0.931 | 0.863 | 1.706082142 | 8.530410711 |

| | | | | | |
|---|---|---|---|---|---|
| 0.072 | 0.425 | 0.956 | 1.57 | 2.02 | 2.611 |
| 0 | 0.353 | 0.884 | 1.498 | 1.948 | 2.539 |
| 0 | 1.25 | 2.5 | 5 | 10 | 20 |

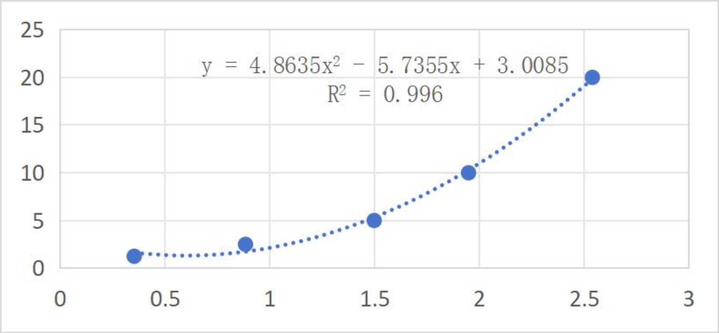


| Sample ID | OD (450nm) | Blank Corrected | Dilution Factor | Concentration |
|---|---|---|---|---|
| 7 | 0.877 | 0.805 | 1.543092088 | 7.715460438 |
| 8 | 0.872 | 0.8 | 1.53274 | 7.6637 |
| 9 | 0.82 | 0.748 | 1.439493704 | 7.19746852 |
| 10 | 0.927 | 0.855 | 1.659987588 | 8.299937938 |
| 11 | 0.933 | 0.861 | 1.675649184 | 8.378245918 |
| 12 | 0.794 | 0.722 | 1.402733734 | 7.01366867 |
| 19 | 0.958 | 0.886 | 1.744675046 | 8.72337523 |
| 20 | 0.840 | 0.768 | 1.472245024 | 7.36122512 |
| 21 | 0.906 | 0.834 | 1.607929606 | 8.03964803 |
| 22 | 0.970 | 0.898 | 1.779966854 | 8.89983427 |
| 23 | 0.911 | 0.839 | 1.619935284 | 8.099676418 |
| 24 | 0.886 | 0.814 | 1.562338646 | 7.81169323 |
| 25 | 0.933 | 0.861 | 1.675649184 | 8.378245918 |
| 26 | 0.909 | 0.837 | 1.615103832 | 8.075519158 |
| 27 | 0.822 | 0.75 | 1.44259375 | 7.21296875 |
| 28 | 0.938 | 0.866 | 1.688968006 | 8.44484003 |
| 29 | 0.961 | 0.889 | 1.753366684 | 8.766833418 |
| 30 | 0.948 | 0.876 | 1.716335176 | 8.58167588 |
| 31 | 0.900 | 0.828 | 1.593843784 | 7.96921892 |
| 32 | 0.866 | 0.794 | 1.520638486 | 7.60319243 |
| 33 | 0.928 | 0.856 | 1.662573536 | 8.31286768 |
| 34 | 0.914 | 0.842 | 1.627255414 | 8.13627707 |
| 35 | 0.957 | 0.885 | 1.741797288 | 8.708986438 |
| 36 | 0.926 | 0.854 | 1.657411366 | 8.28705683 |
| 37 | 0.955 | 0.883 | 1.736070952 | 8.680354758 |
| 38 | 0.831 | 0.759 | 1.457025444 | 7.285127218 |
| 39 | 0.844 | 0.772 | 1.479262184 | 7.39631092 |
| 40 | 0.922 | 0.85 | 1.64720375 | 8.23601875 |
| 41 | 0.955 | 0.883 | 1.736070952 | 8.680354758 |
| 42 | 0.877 | 0.805 | 1.543092088 | 7.715460438 |
| 43 | 0.813 | 0.741 | 1.428949944 | 7.144749718 |
| 44 | 0.899 | 0.827 | 1.591530192 | 7.957650958 |
| 45 | 0.925 | 0.853 | 1.654844872 | 8.274224358 |
| 46 | 0.863 | 0.791 | 1.514719044 | 7.573595218 |
| 47 | 0.931 | 0.859 | 1.670389744 | 8.351948718 |
| 48 | 0.940 | 0.868 | 1.694363624 | 8.47181812 |

| | | | | |
|---|---|---|---|---|
| 49 | 0.950 | 0.878 | 1.721925334 | 8.60962667 |
| 50 | 0.963 | 0.891 | 1.759209744 | 8.796048718 |
| 51 | 0.884 | 0.812 | 1.557993544 | 7.78996772 |
| 52 | 0.935 | 0.863 | 1.680947532 | 8.404737658 |
| 53 | 0.933 | 0.861 | 1.675649184 | 8.378245918 |
| 54 | 0.942 | 0.87 | 1.69979815 | 8.49899075 |
| 55 | 0.859 | 0.787 | 1.506962632 | 7.534813158 |
| 56 | 0.966 | 0.894 | 1.768047286 | 8.84023643 |
| 57 | 0.851 | 0.779 | 1.491916704 | 7.459583518 |
| 58 | 0.874 | 0.802 | 1.536851654 | 7.68425827 |
| 59 | 0.923 | 0.851 | 1.649741064 | 8.248705318 |
| 60 | 0.920 | 0.848 | 1.642158304 | 8.21079152 |

| | | | | | |
|---|---|---|---|---|---|
| 0.069 | 0.419 | 0.952 | 1.569 | 2.017 | 2.614 |
| 0 | 0.35 | 0.883 | 1.5 | 1.948 | 2.545 |
| 0 | 1.25 | 2.5 | 5 | 10 | 20 |

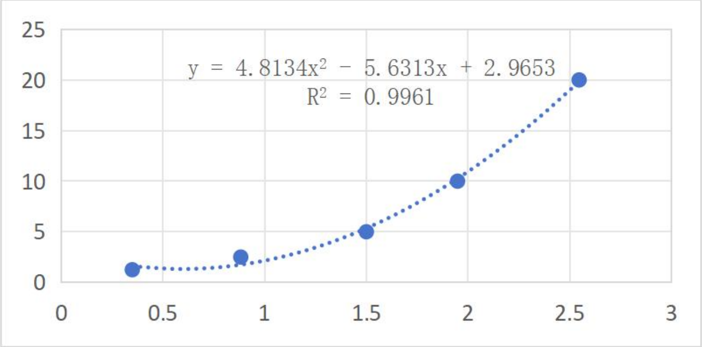


| Blank Corrected | Dilution Factor | Concentration |
|---|---|---|
| 0.808 | 1.557705178 | 7.788525888 |
| 0.803 | 1.547089741 | 7.735448703 |
| 0.751 | 1.450956113 | 7.254780567 |
| 0.858 | 1.677096398 | 8.385481988 |
| 0.864 | 1.693040646 | 8.465203232 |
| 0.725 | 1.412650875 | 7.063254375 |
| 0.889 | 1.763205401 | 8.816027007 |
| 0.771 | 1.484850009 | 7.424250047 |
| 0.837 | 1.624020725 | 8.120103623 |
| 0.901 | 1.799021633 | 8.995108167 |
| 0.842 | 1.636272718 | 8.181363588 |
| 0.817 | 1.577419453 | 7.887097263 |
| 0.864 | 1.693040646 | 8.465203232 |
| 0.84 | 1.63134304 | 8.1567152 |
| 0.753 | 1.454172221 | 7.270861103 |
| 0.869 | 1.706592257 | 8.532961287 |
| 0.892 | 1.772029498 | 8.860147488 |
| 0.879 | 1.734417489 | 8.672087447 |
| 0.831 | 1.609636017 | 8.048180087 |
| 0.797 | 1.534668901 | 7.673344503 |
| 0.859 | 1.679729705 | 8.398648527 |
| 0.845 | 1.643739435 | 8.218697175 |
| 0.888 | 1.76028329 | 8.801416448 |
| 0.857 | 1.674472717 | 8.372363583 |
| 0.886 | 1.754467946 | 8.772339732 |
| 0.762 | 1.46912123 | 7.345606148 |
| 0.775 | 1.492090875 | 7.460454375 |
| 0.853 | 1.664074261 | 8.320371303 |
| 0.886 | 1.754467946 | 8.772339732 |
| 0.808 | 1.557705178 | 7.788525888 |
| 0.744 | 1.440002982 | 7.200014912 |
| 0.83 | 1.60727226 | 8.0363613 |
| 0.856 | 1.671858662 | 8.359293312 |
| 0.794 | 1.528588442 | 7.642942212 |
| 0.862 | 1.68768739 | 8.438436948 |
| 0.871 | 1.712080289 | 8.560401447 |

| | | |
|---|---|---|
| 0.881 | 1.740098057 | 8.700490287 |
| 0.894 | 1.777960362 | 8.889801812 |
| 0.815 | 1.572971115 | 7.864855575 |
| 0.866 | 1.69843241 | 8.492162052 |
| 0.864 | 1.693040646 | 8.465203232 |
| 0.873 | 1.717606829 | 8.588034143 |
| 0.79 | 1.52061594 | 7.6030797 |
| 0.897 | 1.786928861 | 8.934644303 |
| 0.782 | 1.505133022 | 7.525665108 |
| 0.805 | 1.551307035 | 7.756535175 |
| 0.854 | 1.666659434 | 8.333297172 |
| 0.851 | 1.658932793 | 8.294663967 |

Report

TSH

| Group | Mean | Std. Deviation |
|---|---|---|
| 1 | 8.087781 | .6488272388394 |
| 2 | 8.323028 | .5426198511602 |
| 3 | 8.285393 | .5210950233504 |
| Total | 8.250103 | .5590010525052 |

Tests of Normality

| | Group | Kolmogorov-Smirnova | | | Shapiro-Wilk | | |
|---|---|---|---|---|---|---|---|
| | | Statistic | df | Sig. | Statistic | df | Sig. |
| TSH | 1 | 0.15 | 12 | .200* | 0.965 | 12 | 0.846 |
| | 2 | 0.199 | 18 | 0.057 | 0.903 | 18 | 0.066 |
| | 3 | 0.181 | 18 | 0.124 | 0.932 | 18 | 0.209 |

* This is a lower bound of the true significance.

a Lilliefors Significance Correction

ANOVA

TSH

| | Sum of Sq | df | Mean Squa | F | Sig. |
|---|---|---|---|---|---|
| Between ( | 0.434 | 2 | 0.217 | 0.686 | 0.509 |
| Within Gr | 14.252 | 45 | 0.317 | | |
| Total | 14.687 | 47 | | | |

Multiple Comparisons

Dependent Variable: TSH

Tukey HSD

| (I) Group | (J) Group | Mean Diff | Std. Erro | Sig. | 95% Confidence Interval | |
|---|---|---|---|---|---|---|
| | | | | | Lower Bou | Upper Bound |
| 1 | 2 | -0.23525 | .2097348 | 0.506 | -0.74356 | 0.27307 |
| | 3 | -0.19761 | .2097348 | 0.617 | -0.70593 | 0.310705 |
| 2 | 1 | .2352462 | .2097348 | 0.506 | -0.27307 | 0.743562 |
| | 3 | .0376349 | .1875925 | 0.978 | -0.41702 | 0.492287 |
| 3 | 1 | .1976112 | .2097348 | 0.617 | -0.3107 | 0.705928 |
| | 2 | -0.03763 | .1875925 | 0.978 | -0.49229 | 0.417017 |

| | | | | | | |
|---|---|---|---|---|---|---|
| 0.051 | 0.308 | 0.552 | 1.053 | 1.698 | 2.504 | 0.062 |
| | 0.257 | 0.501 | 1.002 | 1.647 | 2.453 | 0 |
| 0 | 1 | 2 | 4 | 8 | 16 | |

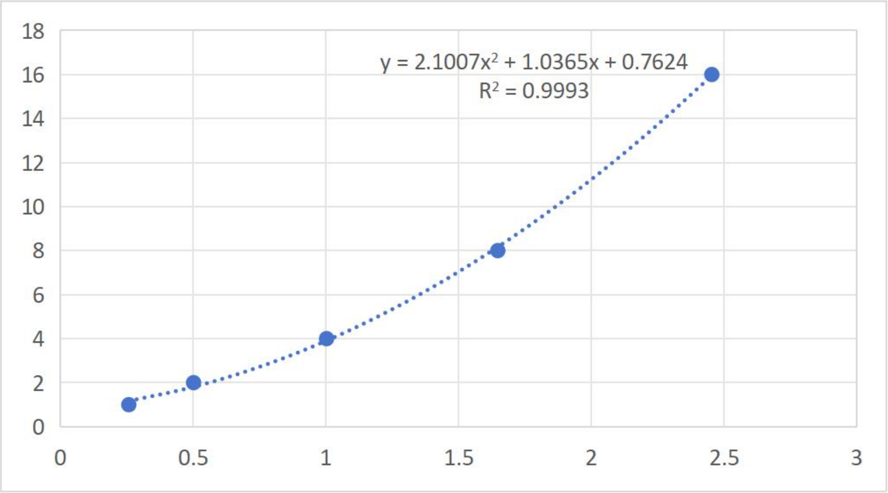


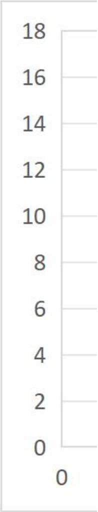


| Sample I | IOD （450nm） | Blank Correct | Dilution Fact | Concentration | | Blank Correct |
|---|---|---|---|---|---|---|
| 7 | 1.123 | 1.072 | 4.287618829 | 21.43809414 | | 1.061 |
| 8 | 1.283 | 1.232 | 5.227860877 | 26.13930438 | | 1.221 |
| 9 | 1.322 | 1.271 | 5.473348409 | 27.36674204 | | 1.26 |
| 10 | 1.258 | 1.207 | 5.073858194 | 25.36929097 | | 1.196 |
| 11 | 1.338 | 1.287 | 5.575909858 | 27.87954929 | | 1.276 |
| 12 | 1.274 | 1.223 | 5.17211741 | 25.86058705 | | 1.212 |
| 19 | 1.177 | 1.126 | 4.592926113 | 22.96463057 | | 1.115 |
| 20 | 1.23 | 1.179 | 4.904492629 | 24.52246314 | | 1.168 |
| 21 | 1.261 | 1.21 | 5.09219987 | 25.46099935 | | 1.199 |
| 22 | 1.134 | 1.083 | 4.348817422 | 21.74408711 | | 1.072 |
| 23 | 1.266 | 1.215 | 5.122853358 | 25.61426679 | | 1.204 |
| 24 | 1.145 | 1.094 | 4.410524385 | 22.05262193 | | 1.083 |
| 25 | 0.928 | 0.877 | 3.28711979 | 16.43559895 | | 0.866 |
| 26 | 0.829 | 0.778 | 2.840317099 | 14.20158549 | | 0.767 |
| 27 | 0.814 | 0.763 | 2.776211918 | 13.88105959 | | 0.752 |
| 28 | 0.896 | 0.845 | 3.138194818 | 15.69097409 | | 0.834 |
| 29 | 0.857 | 0.806 | 2.962509345 | 14.81254673 | | 0.795 |
| 30 | 0.902 | 0.851 | 3.165790541 | 15.8289527 | | 0.84 |
| 31 | 0.929 | 0.878 | 3.291843019 | 16.45921509 | | 0.867 |
| 32 | 0.877 | 0.826 | 3.051806193 | 15.25903097 | | 0.815 |
| 33 | 0.915 | 0.864 | 3.226100147 | 16.13050074 | | 0.853 |
| 34 | 0.839 | 0.788 | 2.883579061 | 14.4178953 | | 0.777 |
| 35 | 0.923 | 0.872 | 3.263566669 | 16.31783334 | | 0.861 |
| 36 | 0.998 | 0.947 | 3.627892166 | 18.13946083 | | 0.936 |
| 37 | 0.788 | 0.737 | 2.667335618 | 13.33667809 | | 0.726 |
| 38 | 0.808 | 0.757 | 2.750834534 | 13.75417267 | | 0.746 |
| 39 | 0.871 | 0.82 | 3.02484068 | 15.1242034 | | 0.809 |

| | | | | | |
|---|---|---|---|---|---|
| 40 | 0.861 | 0.81 | 2.98023427 | 14.90117135 | 0.799 |
| 41 | 0.811 | 0.76 | 2.76350432 | 13.8175216 | 0.749 |
| 42 | 0.897 | 0.846 | 3.142783601 | 15.71391801 | 0.835 |
| 43 | 0.919 | 0.868 | 3.244799797 | 16.22399898 | 0.857 |
| 44 | 1.006 | 0.955 | 3.668148418 | 18.34074209 | 0.944 |
| 45 | 0.989 | 0.938 | 3.582925291 | 17.91462645 | 0.927 |
| 46 | 0.997 | 0.946 | 3.622879041 | 18.11439521 | 0.935 |
| 47 | 0.959 | 0.908 | 3.435493525 | 17.17746762 | 0.897 |
| 48 | 1.017 | 0.966 | 3.723939809 | 18.61969905 | 0.955 |
| 49 | 0.942 | 0.891 | 3.353627317 | 16.76813658 | 0.88 |
| 50 | 0.961 | 0.91 | 3.44520467 | 17.22602335 | 0.899 |
| 51 | 0.934 | 0.883 | 3.315522182 | 16.57761091 | 0.872 |
| 52 | 1.004 | 0.953 | 3.658059146 | 18.29029573 | 0.942 |
| 53 | 0.923 | 0.872 | 3.263566669 | 16.31783334 | 0.861 |
| 54 | 1.088 | 1.037 | 4.096278158 | 20.48139079 | 1.026 |
| 55 | 1.019 | 0.968 | 3.734138317 | 18.67069158 | 0.957 |
| 56 | 1.041 | 0.99 | 3.84743107 | 19.23715535 | 0.979 |
| 57 | 1.053 | 1.002 | 3.910084203 | 19.55042101 | 0.991 |
| 58 | 1.008 | 0.957 | 3.678254494 | 18.39127247 | 0.946 |

| | | | | |
|---|---|---|---|---|
| 0.353 | 0.566 | 1.064 | 1.732 | 2.512 |
| 0.291 | 0.504 | 1.002 | 1.67 | 2.45 |
| 1 | 2 | 4 | 8 | 16 |

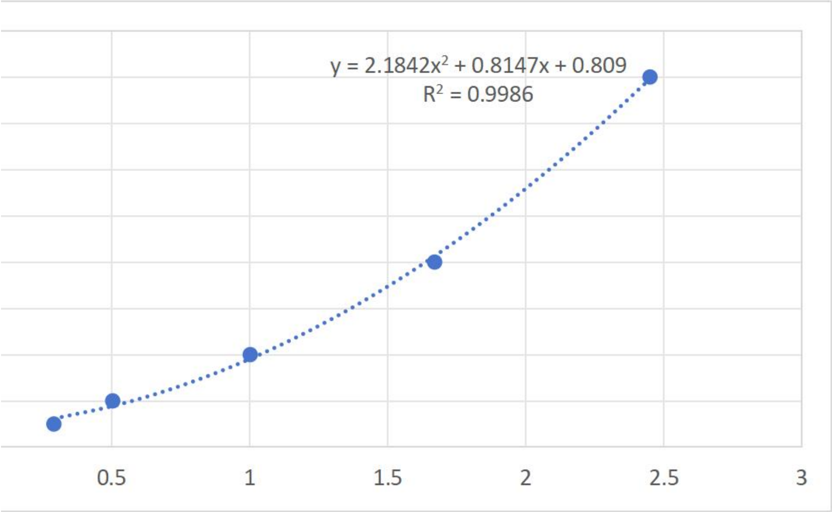


| Dilution Fact | Concentration |
|---|---|
| 4.132196508 | 20.66098254 |
| 5.060043612 | 25.30021806 |
| 5.30315792 | 26.5157896 |
| 4.907695827 | 24.53847914 |
| 5.404819219 | 27.0240961 |
| 5.004883885 | 25.02441942 |
| 4.432842545 | 22.16421273 |
| 4.740307661 | 23.7015383 |
| 4.925833404 | 24.62916702 |
| 4.192406093 | 20.96203046 |
| 4.956150067 | 24.78075034 |
| 4.253144254 | 21.26572127 |
| 3.152584095 | 15.76292048 |
| 2.718815734 | 13.59407867 |
| 2.656828237 | 13.28414118 |
| 3.007693215 | 15.03846608 |
| 2.837155505 | 14.18577753 |
| 3.03451952 | 15.1725976 |
| 3.157184014 | 15.78592007 |
| 2.923780745 | 14.61890373 |
| 3.093182678 | 15.46591339 |
| 2.760686782 | 13.80343391 |
| 3.129650028 | 15.64825014 |
| 3.485128083 | 17.42564042 |
| 2.551711599 | 12.758558 |
| 2.632308447 | 13.16154224 |
| 2.8976097 | 14.4880485 |

| | |
|---|---|
| 2.854340764 | 14.27170382 |
| 2.644548684 | 13.22274342 |
| 3.012153345 | 15.06076673 |
| 3.111381406 | 15.55690703 |
| 3.524496051 | 17.62248026 |
| 3.441173302 | 17.20586651 |
| 3.480226745 | 17.40113373 |
| 3.297212878 | 16.48606439 |
| 3.579083505 | 17.89541753 |
| 3.21738048 | 16.0869024 |
| 3.306687924 | 16.53343962 |
| 3.180249133 | 15.90124566 |
| 3.514627849 | 17.57313924 |
| 3.129650028 | 15.64825014 |
| 3.944137119 | 19.7206856 |
| 3.589065286 | 17.94532643 |
| 3.700018132 | 18.50009066 |
| 3.76142902 | 18.8071451 |
| 3.534381727 | 17.67190864 |

| | | | | | | | | |
|---|---|---|---|---|---|---|---|---|
| 0.057 | 0.346 | 0.551 | 1.062 | 1.724 | 2.511 | | 0.061 | 0.355 |
| 0 | 0.289 | 0.494 | 1.005 | 1.667 | 2.454 | | 0 | 0.294 |
| | 1 | 2 | 4 | 8 | 16 | | | 1 |

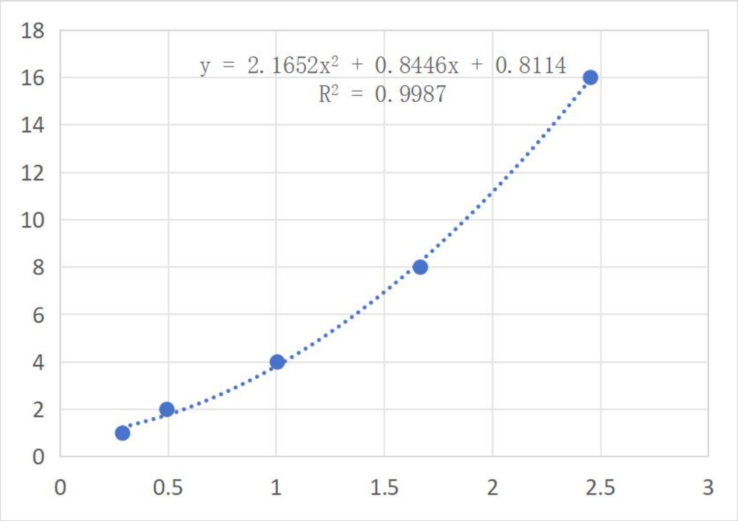


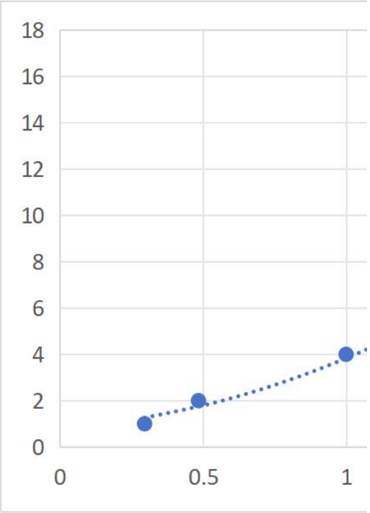


| Sample II | OD （450r | Blank Co | Dilution | Concentration | Blank Co | Dilution Factc | Concentratic |
|---|---|---|---|---|---|---|---|
| 7 | 1.044 | 0.987 | 3.75429 | 18.7715 | 0.983 | 3.759264201 | 18.796321 |
| 8 | 1.476 | 1.419 | 6.36965 | 31.8482 | 1.415 | 6.366273523 | 31.8313676 |
| 9 | 1.32 | 1.263 | 5.33199 | 26.6599 | 1.259 | 5.332364896 | 26.6618245 |
| 10 | 1.248 | 1.191 | 4.88861 | 24.4431 | 1.187 | 4.890439509 | 24.4521975 |
| 11 | 1.339 | 1.282 | 5.45274 | 27.2637 | 1.278 | 5.45269816 | 27.2634908 |
| 12 | 1.081 | 1.024 | 3.94665 | 19.7332 | 1.02 | 3.95115524 | 19.7557762 |
| 19 | 1.172 | 1.115 | 4.44496 | 22.2248 | 1.111 | 4.44812454 | 22.2406227 |
| 20 | 1.130 | 1.073 | 4.21051 | 21.0526 | 1.069 | 4.214333304 | 21.0716665 |
| 21 | 1.242 | 1.185 | 4.85268 | 24.2634 | 1.181 | 4.854617704 | 24.2730885 |
| 22 | 1.128 | 1.071 | 4.19954 | 20.9977 | 1.067 | 4.203389421 | 21.0169471 |
| 23 | 1.072 | 1.015 | 3.89931 | 19.4966 | 1.011 | 3.90393772 | 19.5196886 |
| 24 | 1.120 | 1.063 | 4.15582 | 20.7791 | 1.059 | 4.159785736 | 20.7989287 |
| 25 | 0.921 | 0.864 | 3.15745 | 15.7872 | 0.86 | 3.16363076 | 15.8181538 |
| 26 | 0.844 | 0.787 | 2.81716 | 14.0858 | 0.783 | 2.823835281 | 14.1191764 |
| 27 | 0.822 | 0.765 | 2.72465 | 13.6232 | 0.761 | 2.73142942 | 13.6571471 |
| 28 | 0.901 | 0.844 | 3.06659 | 15.333 | 0.84 | 3.07292336 | 15.3646168 |
| 29 | 0.863 | 0.806 | 2.89874 | 14.4937 | 0.802 | 2.905313712 | 14.5265686 |
| 30 | 0.900 | 0.843 | 3.0621 | 15.3105 | 0.839 | 3.0684331 | 15.3421655 |
| 31 | 0.916 | 0.859 | 3.13457 | 15.6729 | 0.855 | 3.140792803 | 15.703964 |
| 32 | 0.868 | 0.811 | 2.92047 | 14.6023 | 0.807 | 2.927013177 | 14.6350659 |
| 33 | 0.911 | 0.854 | 3.1118 | 15.559 | 0.85 | 3.11806225 | 15.5903113 |
| 34 | 0.840 | 0.783 | 2.80018 | 14.0009 | 0.779 | 2.806879552 | 14.0343978 |

| | | | | | | | |
|---|---|---|---|---|---|---|---|
| 35 | 0.919 | 0.862 | 3.14828 | 15.7414 | 0.858 | 3.154482688 | 15.7724134 |
| 36 | 1.002 | 0.945 | 3.54312 | 17.7156 | 0.941 | 3.548569336 | 17.7428467 |
| 37 | 0.798 | 0.741 | 2.62612 | 13.1306 | 0.737 | 2.632994529 | 13.1649726 |
| 38 | 0.812 | 0.755 | 2.68329 | 13.4165 | 0.751 | 2.690114148 | 13.4505707 |
| 39 | 0.874 | 0.817 | 2.94669 | 14.7334 | 0.813 | 2.953194309 | 14.7659715 |
| 40 | 0.877 | 0.82 | 2.95985 | 14.7993 | 0.816 | 2.966342874 | 14.8317144 |
| 41 | 0.802 | 0.745 | 2.64237 | 13.2118 | 0.741 | 2.649228496 | 13.2461425 |
| 42 | 0.902 | 0.845 | 3.07109 | 15.3555 | 0.841 | 3.077417916 | 15.3870896 |
| 43 | 0.916 | 0.859 | 3.13457 | 15.6729 | 0.855 | 3.140792803 | 15.703964 |
| 44 | 1.011 | 0.954 | 3.58773 | 17.9387 | 0.95 | 3.59308025 | 17.9654013 |
| 45 | 0.993 | 0.936 | 3.49887 | 17.4943 | 0.932 | 3.504406414 | 17.5220321 |
| 46 | 0.997 | 0.94 | 3.51849 | 17.5925 | 0.936 | 3.523991418 | 17.6199571 |
| 47 | 0.963 | 0.906 | 3.35388 | 16.7694 | 0.902 | 3.359709952 | 16.7985498 |
| 48 | 1.014 | 0.957 | 3.60268 | 18.0134 | 0.953 | 3.607994553 | 18.0399728 |
| 49 | 0.951 | 0.894 | 3.29698 | 16.4849 | 0.89 | 3.30291401 | 16.5145701 |
| 50 | 0.966 | 0.909 | 3.36821 | 16.841 | 0.905 | 3.374005603 | 16.870028 |
| 51 | 0.942 | 0.885 | 3.25471 | 16.2735 | 0.881 | 3.260723044 | 16.3036152 |
| 52 | 1.010 | 0.953 | 3.58276 | 17.9138 | 0.949 | 3.588117408 | 17.940587 |
| 53 | 0.932 | 0.875 | 3.20816 | 16.0408 | 0.871 | 3.214252332 | 16.0712617 |
| 54 | 1.068 | 1.011 | 3.87839 | 19.3919 | 1.007 | 3.883063857 | 19.4153193 |
| 55 | 1.012 | 0.955 | 3.59271 | 17.9635 | 0.951 | 3.598047388 | 17.9902369 |
| 56 | 1.042 | 0.985 | 3.74406 | 18.7203 | 0.981 | 3.749059264 | 18.7452963 |
| 57 | 0.972 | 0.915 | 3.39697 | 16.9848 | 0.911 | 3.4027129 | 17.0135645 |
| 58 | 1.109 | 1.052 | 4.09615 | 20.4808 | 1.048 | 4.100279622 | 20.5013981 |

| | | | |
|---|---|---|---|
| 0.543 | 1.058 | 1.727 | 2.515 |
| 0.482 | 0.997 | 1.666 | 2.454 |
| 2 | 4 | 8 | 16 |

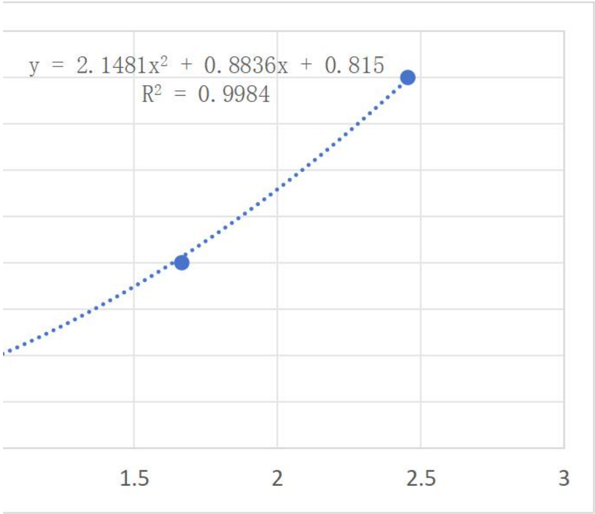


on

| | | | | | |
|---|---|---|---|---|---|
| 0.055 | 0.336 | 0.544 | 1.05 | 1.713 | 2.5 |
| 0 | 0.281 | 0.489 | 0.995 | 1.658 | 2.445 |
| 0 | 1 | 2 | 4 | 8 | 16 |

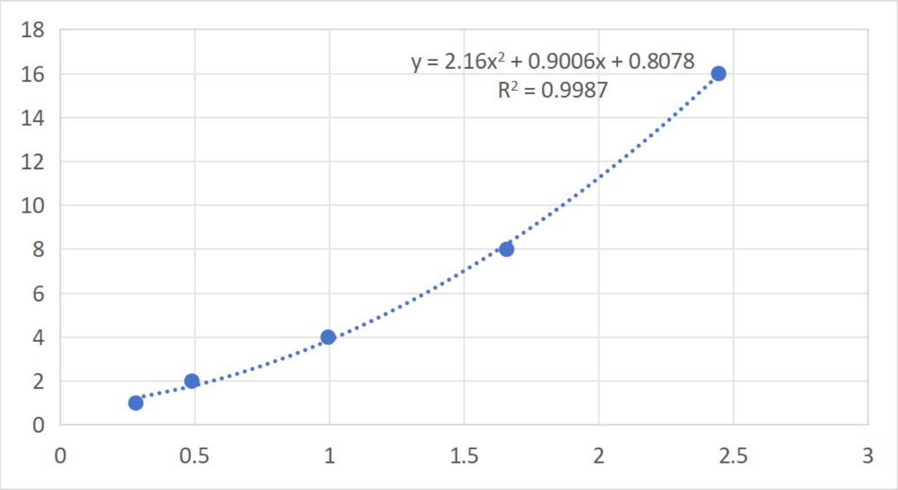


| Sample ID | OD (450nm) | Blank Corrected | Dilution Factor | Concentration |
|---|---|---|---|---|
| 7 | 1.034 | 0.979 | 3.75971996 | 18.7985998 |
| 8 | 1.479 | 1.424 | 6.47025056 | 32.3512528 |
| 9 | 1.222 | 1.167 | 4.80048044 | 24.0024022 |
| 10 | 1.253 | 1.198 | 4.98675944 | 24.9337972 |
| 11 | 1.328 | 1.273 | 5.45460644 | 27.2730322 |
| 12 | 1.077 | 1.022 | 3.98429864 | 19.9214932 |
| 19 | 1.180 | 1.125 | 4.554725 | 22.773625 |
| 20 | 1.237 | 1.182 | 4.89009704 | 24.4504852 |
| 21 | 1.254 | 1.199 | 4.99283756 | 24.9641878 |
| 22 | 1.137 | 1.082 | 4.31101304 | 21.5550652 |
| 23 | 1.076 | 1.021 | 3.97898516 | 19.8949258 |
| 24 | 1.125 | 1.07 | 4.244426 | 21.22213 |
| 25 | 0.931 | 0.876 | 3.25425776 | 16.2712888 |
| 26 | 0.835 | 0.78 | 2.824412 | 14.12206 |
| 27 | 0.829 | 0.774 | 2.79886856 | 13.9943428 |
| 28 | 0.889 | 0.834 | 3.06130136 | 15.3065068 |
| 29 | 0.850 | 0.795 | 2.888951 | 14.444755 |
| 30 | 0.905 | 0.85 | 3.13391 | 15.66955 |
| 31 | 0.924 | 0.869 | 3.22156916 | 16.1078458 |
| 32 | 0.863 | 0.808 | 2.94567104 | 14.7283552 |
| 33 | 0.909 | 0.854 | 3.15223496 | 15.7611748 |
| 34 | 0.845 | 0.79 | 2.86733 | 14.33665 |
| 35 | 0.932 | 0.877 | 3.25894484 | 16.2947242 |
| 36 | 0.991 | 0.936 | 3.54312896 | 17.7156448 |
| 37 | 0.785 | 0.73 | 2.616302 | 13.08151 |
| 38 | 0.810 | 0.755 | 2.719007 | 13.595035 |
| 39 | 0.882 | 0.827 | 3.02988284 | 15.1494142 |
| 40 | 0.873 | 0.818 | 2.98979864 | 14.9489932 |
| 41 | 0.805 | 0.75 | 2.69825 | 13.49125 |
| 42 | 0.884 | 0.829 | 3.03883796 | 15.1941898 |
| 43 | 0.925 | 0.87 | 3.226226 | 16.13113 |

| | | | | |
|---|---|---|---|---|
| 44 | 1.001 | 0.946 | 3.59278616 | 17.9639308 |
| 45 | 0.991 | 0.936 | 3.54312896 | 17.7156448 |
| 46 | 0.989 | 0.934 | 3.53324936 | 17.6662468 |
| 47 | 0.969 | 0.914 | 3.43540376 | 17.1770188 |
| 48 | 1.027 | 0.972 | 3.72391664 | 18.6195832 |
| 49 | 0.947 | 0.892 | 3.32976944 | 16.6488472 |
| 50 | 0.961 | 0.906 | 3.39674936 | 16.9837468 |
| 51 | 0.940 | 0.885 | 3.296597 | 16.482985 |
| 52 | 1.012 | 0.957 | 3.64790804 | 18.2395402 |
| 53 | 0.920 | 0.865 | 3.202985 | 16.014925 |
| 54 | 1.028 | 0.973 | 3.72901844 | 18.6450922 |
| 55 | 1.008 | 0.953 | 3.62780324 | 18.1390162 |
| 56 | 1.030 | 0.975 | 3.739235 | 18.696175 |
| 57 | 0.966 | 0.911 | 3.42087596 | 17.1043798 |
| 58 | 1.017 | 0.962 | 3.67313624 | 18.3656812 |

| | | | | | |
|---|---|---|---|---|---|
| 0.06 | 0.357 | 0.558 | 1.066 | 1.72 | 2.509 |
| 0 | 0.297 | 0.498 | 1.006 | 1.66 | 2.449 |
| 0 | 1 | 2 | 4 | 8 | 16 |

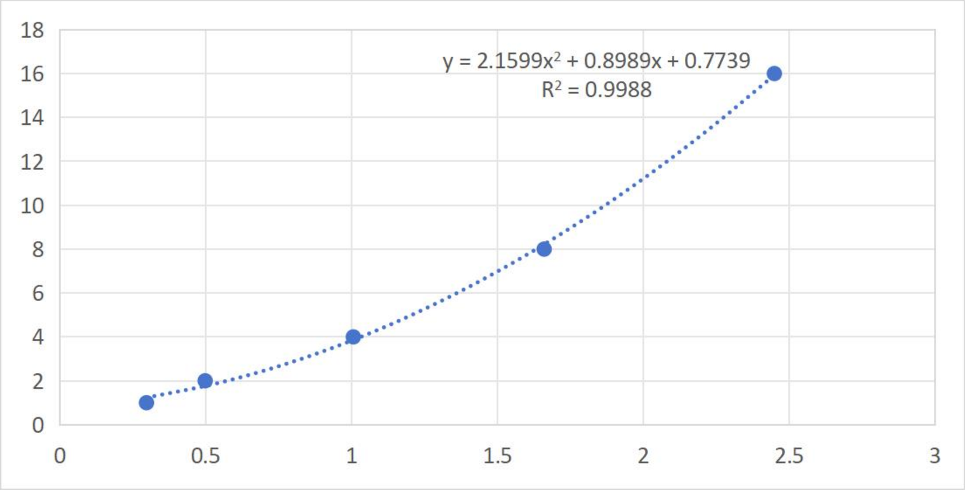


| Blank Corrected | Dilution Factor | Concentration |
|---|---|---|
| 0.974 | 3.698473892 | 18.49236946 |
| 1.419 | 6.398529504 | 31.99264752 |
| 1.162 | 4.734813816 | 23.67406908 |
| 1.193 | 4.920363215 | 24.60181608 |
| 1.268 | 5.386444258 | 26.93222129 |
| 1.017 | 3.922042111 | 19.61021056 |
| 1.12 | 4.49004656 | 22.4502328 |
| 1.177 | 4.824077407 | 24.12038704 |
| 1.194 | 4.926417796 | 24.63208898 |
| 1.077 | 4.247345947 | 21.23672974 |
| 1.016 | 3.916752134 | 19.58376067 |
| 1.065 | 4.181041078 | 20.90520539 |
| 0.871 | 3.195430596 | 15.97715298 |
| 0.775 | 2.767837438 | 13.83918719 |
| 0.769 | 2.742434724 | 13.71217362 |
| 0.829 | 3.003459936 | 15.01729968 |
| 0.79 | 2.83202459 | 14.16012295 |
| 0.845 | 3.075693098 | 15.37846549 |
| 0.864 | 3.16290631 | 15.81453155 |
| 0.803 | 2.888439659 | 14.4421983 |
| 0.849 | 3.09392418 | 15.4696209 |
| 0.785 | 2.810520878 | 14.05260439 |
| 0.872 | 3.200094202 | 16.00047101 |
| 0.931 | 3.482892984 | 17.41446492 |
| 0.725 | 2.560899938 | 12.80449969 |
| 0.75 | 2.66301875 | 13.31509375 |
| 0.822 | 2.972205672 | 14.86102836 |
| 0.813 | 2.932332643 | 14.66166322 |
| 0.745 | 2.642378998 | 13.21189499 |
| 0.824 | 2.981113862 | 14.90556931 |
| 0.865 | 3.167539678 | 15.83769839 |

| | | |
|---|---|---|
| 0.941 | 3.532315312 | 17.66157656 |
| 0.931 | 3.482892984 | 17.41446492 |
| 0.929 | 3.473060356 | 17.36530178 |
| 0.909 | 3.375684432 | 16.87842216 |
| 0.967 | 3.662835031 | 18.31417516 |
| 0.887 | 3.270566663 | 16.35283332 |
| 0.901 | 3.33721788 | 16.6860894 |
| 0.88 | 3.23755856 | 16.1877928 |
| 0.952 | 3.58717881 | 17.93589405 |
| 0.86 | 3.14441604 | 15.7220802 |
| 0.968 | 3.667913338 | 18.33956669 |
| 0.948 | 3.56716797 | 17.83583985 |
| 0.97 | 3.67808291 | 18.39041455 |
| 0.906 | 3.361227076 | 16.80613538 |
| 0.957 | 3.612289555 | 18.06144778 |

| | | | | |
|---|---|---|---|---|
| 21.43809414 | 20.66098254 | 21.04953834 | 18.77145459 | 18.796321 |
| 32.77206918 | 31.88557446 | 32.32882182 | 31.84824839 | 31.8313676 |
| 27.36674204 | 26.5157896 | 26.94126582 | 26.65994859 | 26.6618245 |
| 25.36929097 | 24.53847914 | 24.95388506 | 24.44306831 | 24.4521975 |
| 27.87954929 | 27.0240961 | 27.4518227 | 27.26367682 | 27.2634908 |
| 25.86058705 | 25.02441942 | 25.44250324 | 19.73323578 | 19.7557762 |
| 22.96463057 | 22.16421273 | 22.56442165 | 22.22479885 | 22.2406227 |
| 24.52246314 | 23.7015383 | 24.11200072 | 21.05256675 | 21.0716665 |
| 25.46099935 | 24.62916702 | 25.04508319 | 24.26339485 | 24.2730885 |
| 21.74408711 | 20.96203046 | 21.35305879 | 20.99769887 | 21.0169471 |
| 25.61426679 | 24.78075034 | 25.19750857 | 19.49656085 | 19.5196886 |
| 22.05262193 | 21.26572127 | 21.6591716 | 20.77909339 | 20.7989287 |
| 16.43559895 | 15.76292048 | 16.09925972 | 15.7872377 | 15.8181538 |
| 14.20158549 | 13.59407867 | 13.89783208 | 14.08578979 | 14.1191764 |
| 13.88105959 | 13.28414118 | 13.58260039 | 13.62324085 | 13.6571471 |
| 15.69097409 | 15.03846608 | 15.36472009 | 15.33296154 | 15.3646168 |
| 14.81254673 | 14.18577753 | 14.49916213 | 14.49369734 | 14.5265686 |
| 15.8289527 | 15.1725976 | 15.50077515 | 15.31047507 | 15.3421655 |
| 16.45921509 | 15.78592007 | 16.12256758 | 15.67285671 | 15.703964 |
| 15.25903097 | 14.61890373 | 14.93896735 | 14.60234055 | 14.6350659 |
| 16.13050074 | 15.46591339 | 15.79820707 | 15.55901702 | 15.5903113 |
| 14.4178953 | 13.80343391 | 14.11066461 | 14.00091051 | 14.0343978 |
| 16.31783334 | 15.64825014 | 15.98304174 | 15.74142034 | 15.7724134 |
| 18.13946083 | 17.42564042 | 17.78255063 | 17.71562365 | 17.7428467 |
| 13.33667809 | 12.758558 | 13.04761805 | 13.13059391 | 13.1649726 |
| 13.75417267 | 13.16154224 | 13.45785746 | 13.41645565 | 13.4505707 |
| 15.1242034 | 14.4880485 | 14.80612595 | 14.73342691 | 14.7659715 |
| 14.90117135 | 14.27170382 | 14.58643759 | 14.7992624 | 14.8317144 |
| 13.8175216 | 13.22274342 | 13.52013251 | 13.21183565 | 13.2461425 |
| 15.71391801 | 15.06076673 | 15.38734237 | 15.35546965 | 15.3870896 |
| 16.22399898 | 15.55690703 | 15.89045301 | 15.67285671 | 15.703964 |
| 18.34074209 | 17.62248026 | 17.98161118 | 17.93865782 | 17.9654013 |
| 17.91462645 | 17.20586651 | 17.56024648 | 17.4943433 | 17.5220321 |
| 18.11439521 | 17.40113373 | 17.75776447 | 17.5924736 | 17.6199571 |
| 17.17746762 | 16.48606439 | 16.83176601 | 16.76940854 | 16.7985498 |
| 18.61969905 | 17.89541753 | 18.25755829 | 18.01339227 | 18.0399728 |
| 16.76813658 | 16.0869024 | 16.42751949 | 16.48489094 | 16.5145701 |
| 17.22602335 | 16.53343962 | 16.87973149 | 16.84102511 | 16.870028 |
| 16.57761091 | 15.90124566 | 16.23942829 | 16.27354885 | 16.3036152 |
| 18.29029573 | 17.57313924 | 17.93171749 | 17.91378963 | 17.940587 |
| 16.31783334 | 15.64825014 | 15.98304174 | 16.04078125 | 16.0712617 |
| 20.48139079 | 19.7206856 | 20.1010382 | 19.39193495 | 19.4153193 |
| 18.67069158 | 17.94532643 | 18.30800901 | 17.96354765 | 17.9902369 |
| 19.23715535 | 18.50009066 | 18.86862301 | 18.72031085 | 18.7452963 |
| 17.03230461 | 16.3444629 | 16.68838376 | 16.98484285 | 17.0135645 |
| 18.39127247 | 17.67190864 | 18.03159056 | 20.4807735 | 20.5013981 |

| | | | |
|---|---|---|---|
| 18.7838878 | 18.7985998 | 18.49236946 | 18.64548463 |
| 31.839808 | 32.3512528 | 31.99264752 | 32.17195016 |
| 26.66088654 | 24.0024022 | 23.67406908 | 23.83823564 |
| 24.44763293 | 24.9337972 | 24.60181608 | 24.76780664 |
| 27.26358381 | 27.2730322 | 26.93222129 | 27.10262675 |
| 19.74450599 | 19.9214932 | 19.61021056 | 19.76585188 |
| 22.23271078 | 22.773625 | 22.4502328 | 22.6119289 |
| 21.06211664 | 24.4504852 | 24.12038704 | 24.28543612 |
| 24.26824169 | 24.9641878 | 24.63208898 | 24.79813839 |
| 21.00732299 | 21.5550652 | 21.23672974 | 21.39589747 |
| 19.50812473 | 19.8949258 | 19.58376067 | 19.73934324 |
| 20.78901104 | 21.22213 | 20.90520539 | 21.0636677 |
| 15.80269575 | 16.2712888 | 15.97715298 | 16.12422089 |
| 14.1024831 | 14.12206 | 13.83918719 | 13.9806236 |
| 13.64019398 | 13.9943428 | 13.71217362 | 13.85325821 |
| 15.34878917 | 15.3065068 | 15.01729968 | 15.16190324 |
| 14.51013295 | 14.444755 | 14.16012295 | 14.30243898 |
| 15.32632029 | 15.66955 | 15.37846549 | 15.52400775 |
| 15.68841036 | 16.1078458 | 15.81453155 | 15.96118868 |
| 14.61870322 | 14.7283552 | 14.4421983 | 14.58527675 |
| 15.57466414 | 15.7611748 | 15.4696209 | 15.61539785 |
| 14.01765414 | 14.33665 | 14.05260439 | 14.1946272 |
| 15.75691689 | 16.2947242 | 16.00047101 | 16.14759761 |
| 17.72923517 | 17.7156448 | 17.41446492 | 17.56505486 |
| 13.14778328 | 13.08151 | 12.80449969 | 12.94300485 |
| 13.4335132 | 13.595035 | 13.31509375 | 13.45506438 |
| 14.74969923 | 15.1494142 | 14.86102836 | 15.00522128 |
| 14.81548839 | 14.9489932 | 14.66166322 | 14.80532821 |
| 13.22898907 | 13.49125 | 13.21189499 | 13.3515725 |
| 15.37127962 | 15.1941898 | 14.90556931 | 15.04987956 |
| 15.68841036 | 16.13113 | 15.83769839 | 15.9844142 |
| 17.95202954 | 17.9639308 | 17.66157656 | 17.81275368 |
| 17.50818769 | 17.7156448 | 17.41446492 | 17.56505486 |
| 17.60621535 | 17.6662468 | 17.36530178 | 17.51577429 |
| 16.78397915 | 17.1770188 | 16.87842216 | 17.02772048 |
| 18.02668252 | 18.6195832 | 18.31417516 | 18.46687918 |
| 16.4997305 | 16.6488472 | 16.35283332 | 16.50084026 |
| 16.85552656 | 16.9837468 | 16.6860894 | 16.8349181 |
| 16.28858204 | 16.482985 | 16.1877928 | 16.3353889 |
| 17.92718834 | 18.2395402 | 17.93589405 | 18.08771713 |
| 16.05602146 | 16.014925 | 15.7220802 | 15.8685026 |
| 19.40362712 | 18.6450922 | 18.33956669 | 18.49232945 |
| 17.9768923 | 18.1390162 | 17.83583985 | 17.98742803 |
| 18.73280359 | 18.696175 | 18.39041455 | 18.54329478 |
| 16.99920368 | 17.1043798 | 16.80613538 | 16.95525759 |
| 20.49108581 | 18.3656812 | 18.06144778 | 18.21356449 |

| | | | |
|---|---|---|---|
| 21.04953834 | 18.7838878 | 18.64548463 | 19.49297026 |
| 32.32882182 | 31.839808 | 32.17195016 | 32.11352666 |
| 26.94126582 | 26.66088654 | 23.83823564 | 25.81346267 |
| 24.95388506 | 24.44763293 | 24.76780664 | 24.72310821 |
| 27.4518227 | 27.26358381 | 27.10262675 | 27.27267775 |
| 25.44250324 | 19.74450599 | 19.76585188 | 21.6509537 |
| 22.56442165 | 22.23271078 | 22.6119289 | 22.46968711 |
| 24.11200072 | 21.06211664 | 24.28543612 | 23.15318449 |
| 25.04508319 | 24.26824169 | 24.79813839 | 24.70382109 |
| 21.35305879 | 21.00732299 | 21.39589747 | 21.25209308 |
| 25.19750857 | 19.50812473 | 19.73934324 | 21.48165885 |
| 21.6591716 | 20.78901104 | 21.0636677 | 21.17061678 |
| 16.09925972 | 15.80269575 | 16.12422089 | 16.00872545 |
| 13.89783208 | 14.1024831 | 13.9806236 | 13.99364626 |
| 13.58260039 | 13.64019398 | 13.85325821 | 13.69201753 |
| 15.36472009 | 15.34878917 | 15.16190324 | 15.29180417 |
| 14.49916213 | 14.51013295 | 14.30243898 | 14.43724469 |
| 15.50077515 | 15.32632029 | 15.52400775 | 15.45036773 |
| 16.12256758 | 15.68841036 | 15.96118868 | 15.92405554 |
| 14.93896735 | 14.61870322 | 14.58527675 | 14.71431577 |
| 15.79820707 | 15.57466414 | 15.61539785 | 15.66275635 |
| 14.11066461 | 14.01765414 | 14.1946272 | 14.10764865 |
| 15.98304174 | 15.75691689 | 16.14759761 | 15.96251875 |
| 17.78255063 | 17.72923517 | 17.56505486 | 17.69228022 |
| 13.04761805 | 13.14778328 | 12.94300485 | 13.04613539 |
| 13.45785746 | 13.4335132 | 13.45506438 | 13.44881168 |
| 14.80612595 | 14.74969923 | 15.00522128 | 14.85368215 |
| 14.58643759 | 14.81548839 | 14.80532821 | 14.7357514 |
| 13.52013251 | 13.22898907 | 13.3515725 | 13.36689803 |
| 15.38734237 | 15.37127962 | 15.04987956 | 15.26950052 |
| 15.89045301 | 15.68841036 | 15.9844142 | 15.85442586 |
| 17.98161118 | 17.95202954 | 17.81275368 | 17.9154648 |
| 17.56024648 | 17.50818769 | 17.56505486 | 17.54449634 |
| 17.75776447 | 17.60621535 | 17.51577429 | 17.6265847 |
| 16.83176601 | 16.78397915 | 17.02772048 | 16.88115521 |
| 18.25755829 | 18.02668252 | 18.46687918 | 18.25037333 |
| 16.42751949 | 16.4997305 | 16.50084026 | 16.47603008 |
| 16.87973149 | 16.85552656 | 16.8349181 | 16.85672538 |
| 16.23942829 | 16.28858204 | 16.3353889 | 16.28779974 |
| 17.93171749 | 17.92718834 | 18.08771713 | 17.98220765 |
| 15.98304174 | 16.05602146 | 15.8685026 | 15.9691886 |
| 20.1010382 | 19.40362712 | 18.49232945 | 19.33233159 |
| 18.30800901 | 17.9768923 | 17.98742803 | 18.09077645 |
| 18.86862301 | 18.73280359 | 18.54329478 | 18.71490713 |
| 16.68838376 | 16.99920368 | 16.95525759 | 16.88094834 |
| 18.03159056 | 20.49108581 | 18.21356449 | 18.91208029 |

Report
FT3 (14)

| Group | Mean | Std. Deviation |
|---|---|---|
| 1 | 23.774813 | 3.4513087965229 |
| 2 | 14.869897 | 1.1742731394459 |
| 3 | 17.473468 | 1.0614643936836 |
| Total | 18.098509 | 4.0876393262666 |

Tests of Normality

| | Group | Kolmogorov-Smirnova | | | Shapiro-Wilk | | |
|---|---|---|---|---|---|---|---|
| | | Statistic | df | Sig. | Statistic | df | Sig. |
| FT3 (14) | 1 | 0.155 | 12 | .200* | 0.894 | 12 | 0.135 |
| | 2 | 0.111 | 18 | .200* | 0.962 | 18 | 0.648 |
| | 3 | 0.149 | 16 | .200* | 0.963 | 16 | 0.722 |

* This is a lower bound of the true significance.
a Lilliefors Significance Correction

ANOVA
FT3 (14)

| | Sum of Sq | df | Mean Squa | F | Sig. |
|---|---|---|---|---|---|
| Between G | 580.527 | 2 | 290.263 | 72.833 | 0 |
| Within Gr | 171.369 | 43 | 3.985 | | |
| Total | 751.896 | 45 | | | |

Robust Tests of Equality of Means
FT3 (14)

| | Statistic | df1 | df2 | Sig. |
|---|---|---|---|---|
| Welch | 49.275 | 2 | 22.584 | 0 |

a Asymptotically F distributed.

Multiple Comparisons
Dependent Variable: FT3 (14)
Games-Howell

| (I) Group | (J) Group | Mean Diff | Std. Errc | Sig. | 95% Confidence Interval | |
|---|---|---|---|---|---|---|
| | | | | | Lower Bou | Upper Bound |
| 1 | 2 | 8.904915 | 1.0340378 | 0 | 6.167004 | 11.642827326283 |
| | 3 | 6.301344 | 1.0310416 | 0 | 3.567383 | 9.035307 |
| 2 | 1 | -8.90491 | 1.0340378 | 0 | -11.6428 | -6.167 |
| | 3 | -2.60357 | 0.38343928 | 0 | -3.54584 | -1.6613 |
| 3 | 1 | -6.30134 | 1.0310416 | 0 | -9.03531 | -3.56738 |
| | 2 | 2.603570 | 0.38343928 | 0 | 1.661299 | 3.545842 |

* The mean difference is significant at the 0.05 level.

| | | | | | |
|---|---|---|---|---|---|
| 0.073 | 0.536 | 0.962 | 1.501 | 1.857 | 2.812 |
| 0 | 0.463 | 0.889 | 1.428 | 1.784 | 2.739 |
| 0 | 7.5 | 15 | 30 | 60 | 120 |

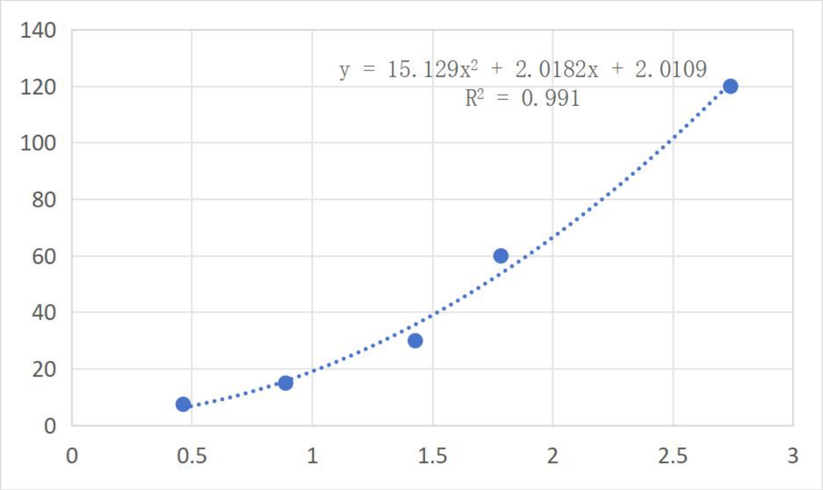


| Sample ID | OD (450nm) | Blank Corrected | Dilution Factor | Concentration |
|---|---|---|---|---|
| 7 | 0.836 | 0.763 | 12.4064904 | 62.03245201 |
| 8 | 0.849 | 0.776 | 12.7362319 | 63.68115952 |
| 9 | 0.813 | 0.74 | 11.8356284 | 59.178142 |
| 10 | 0.882 | 0.809 | 13.59623385 | 67.98116925 |
| 11 | 0.702 | 0.629 | 9.305627489 | 46.52813745 |
| 12 | 0.711 | 0.638 | 9.496874276 | 47.48437138 |
| 19 | 0.762 | 0.689 | 10.62690081 | 53.13450405 |
| 20 | 0.808 | 0.735 | 11.71364603 | 58.56823013 |
| 21 | 0.682 | 0.609 | 8.889409449 | 44.44704725 |
| 22 | 0.678 | 0.605 | 8.807618225 | 44.03809113 |
| 23 | 0.727 | 0.654 | 9.842920164 | 49.21460082 |
| 24 | 0.716 | 0.643 | 9.604181521 | 48.02090761 |
| 25 | 0.524 | 0.451 | 6.026774929 | 30.13387465 |
| 26 | 0.512 | 0.439 | 5.840222809 | 29.20111405 |
| 27 | 0.622 | 0.549 | 7.713374529 | 38.56687265 |
| 28 | 0.503 | 0.43 | 5.7031681 | 28.5158405 |
| 29 | 0.613 | 0.54 | 7.5463644 | 37.731822 |
| 30 | 0.541 | 0.468 | 6.298515696 | 31.49257848 |
| 31 | 0.644 | 0.571 | 8.131939489 | 40.65969745 |
| 32 | 0.578 | 0.505 | 6.920179225 | 34.60089613 |
| 33 | 0.549 | 0.476 | 6.429419504 | 32.14709752 |
| 34 | 0.542 | 0.469 | 6.314772769 | 31.57386385 |
| 35 | 0.622 | 0.549 | 7.713374529 | 38.56687265 |
| 36 | 0.650 | 0.577 | 8.248635241 | 41.24317621 |
| 37 | 0.573 | 0.5 | 6.83375 | 34.16875 |
| 38 | 0.521 | 0.448 | 5.979728416 | 29.89864208 |
| 39 | 0.564 | 0.491 | 6.680083649 | 33.40041825 |

| | | | | |
|---|---|---|---|---|
| 40 | 0.580 | 0.507 | 6.954962721 | 34.77481361 |
| 41 | 0.552 | 0.479 | 6.479007689 | 32.39503845 |
| 42 | 0.533 | 0.46 | 6.1695484 | 30.847742 |
| 43 | 0.652 | 0.579 | 8.287775889 | 41.43887945 |
| 44 | 0.567 | 0.494 | 6.731033444 | 33.65516722 |
| 45 | 0.644 | 0.571 | 8.131939489 | 40.65969745 |
| 46 | 0.603 | 0.53 | 7.3636721 | 36.8183605 |
| 47 | 0.612 | 0.539 | 7.527959009 | 37.63979505 |
| 48 | 0.615 | 0.542 | 7.583265956 | 37.91632978 |
| 49 | 0.647 | 0.574 | 8.190151204 | 40.95075602 |
| 50 | 0.652 | 0.579 | 8.287775889 | 41.43887945 |
| 51 | 0.563 | 0.49 | 6.6631609 | 33.3158045 |
| 52 | 0.568 | 0.495 | 6.748077225 | 33.74038613 |
| 53 | 0.537 | 0.464 | 6.233789984 | 31.16894992 |
| 54 | 0.633 | 0.56 | 7.9208264 | 39.604132 |
| 55 | 0.609 | 0.536 | 7.472924384 | 37.36462192 |
| 56 | 0.655 | 0.582 | 8.346713796 | 41.73356898 |
| 57 | 0.521 | 0.448 | 5.979728416 | 29.89864208 |
| 58 | 0.662 | 0.589 | 8.485294609 | 42.42647305 |

| | | | | | |
|---|---|---|---|---|---|
| 0.07 | 0.542 | 0.977 | 1.513 | 1.868 | 2.846 |
| | 0.472 | 0.907 | 1.443 | 1.798 | 2.776 |
| | 7.5 | 15 | 30 | 60 | 120 |

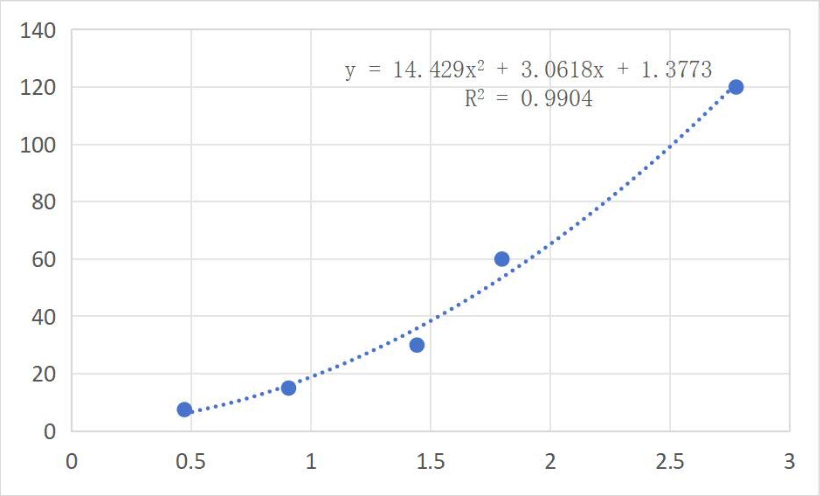


| Sample ID | OD (450nm) | Blank Corrected | Dilution Factor | Concentration |
|---|---|---|---|---|
| 7 | 0.836 | 0.766 | 12.18894112 | 60.94470562 |
| 8 | 0.849 | 0.779 | 12.51855099 | 62.59275495 |
| 9 | 0.813 | 0.743 | 11.61773242 | 58.08866211 |
| 10 | 0.882 | 0.812 | 13.37715618 | 66.88578088 |
| 11 | 0.702 | 0.632 | 9.075646496 | 45.37823248 |
| 12 | 0.711 | 0.641 | 9.268515749 | 46.34257875 |
| 19 | 0.762 | 0.692 | 10.40559426 | 52.02797128 |

| | | | | |
|---|---|---|---|---|
| 20 | 0.808 | 0.738 | 11.49557668 | 57.47788338 |
| 21 | 0.682 | 0.612 | 8.655416976 | 43.27708488 |
| 22 | 0.678 | 0.608 | 8.572756256 | 42.86378128 |
| 23 | 0.727 | 0.657 | 9.617166021 | 48.08583011 |
| 24 | 0.716 | 0.646 | 9.376675364 | 46.88337682 |
| 25 | 0.524 | 0.454 | 5.741404964 | 28.70702482 |
| 26 | 0.512 | 0.442 | 5.549522756 | 27.74761378 |
| 27 | 0.622 | 0.552 | 7.463987616 | 37.31993808 |
| 28 | 0.503 | 0.433 | 5.408338181 | 27.04169091 |
| 29 | 0.613 | 0.543 | 7.294233621 | 36.47116811 |
| 30 | 0.541 | 0.471 | 6.020351589 | 30.10175795 |
| 31 | 0.644 | 0.574 | 7.888782404 | 39.44391202 |
| 32 | 0.578 | 0.508 | 6.656299856 | 33.28149928 |
| 33 | 0.549 | 0.479 | 6.154506389 | 30.77253195 |
| 34 | 0.542 | 0.472 | 6.037019936 | 30.18509968 |
| 35 | 0.622 | 0.552 | 7.463987616 | 37.31993808 |
| 36 | 0.650 | 0.58 | 8.0070596 | 40.035298 |
| 37 | 0.573 | 0.503 | 6.568052261 | 32.84026131 |
| 38 | 0.521 | 0.451 | 5.693044829 | 28.46522415 |
| 39 | 0.564 | 0.494 | 6.411024644 | 32.05512322 |
| 40 | 0.580 | 0.51 | 6.6918009 | 33.4590045 |
| 41 | 0.552 | 0.482 | 6.205290596 | 31.02645298 |
| 42 | 0.533 | 0.463 | 5.888043701 | 29.44021851 |
| 43 | 0.652 | 0.582 | 8.046716196 | 40.23358098 |
| 44 | 0.567 | 0.497 | 6.463107461 | 32.31553731 |
| 45 | 0.644 | 0.574 | 7.888782404 | 39.44391202 |
| 46 | 0.603 | 0.533 | 7.108359581 | 35.54179791 |
| 47 | 0.612 | 0.542 | 7.275516356 | 36.37758178 |
| 48 | 0.615 | 0.545 | 7.331754725 | 36.65877363 |
| 49 | 0.647 | 0.577 | 7.947791141 | 39.73895571 |
| 50 | 0.652 | 0.582 | 8.046716196 | 40.23358098 |
| 51 | 0.563 | 0.493 | 6.393721421 | 31.96860711 |
| 52 | 0.568 | 0.498 | 6.480526116 | 32.40263058 |
| 53 | 0.537 | 0.467 | 5.953966781 | 29.76983391 |
| 54 | 0.633 | 0.563 | 7.674639101 | 38.37319551 |
| 55 | 0.609 | 0.539 | 7.219537709 | 36.09768855 |
| 56 | 0.655 | 0.585 | 8.106417525 | 40.53208763 |
| 57 | 0.521 | 0.451 | 5.693044829 | 28.46522415 |
| 58 | 0.662 | 0.592 | 8.246730656 | 41.23365328 |

| | | | | | | | |
|---|---|---|---|---|---|---|---|
| 0.075 | 0.546 | 0.973 | 1.508 | 1.866 | 2.709 | | 0.068 |
| 0 | 0.471 | 0.898 | 1.433 | 1.791 | 2.634 | | 0 |
| 0 | 7.5 | 15 | 30 | 60 | 120 | | 0 |

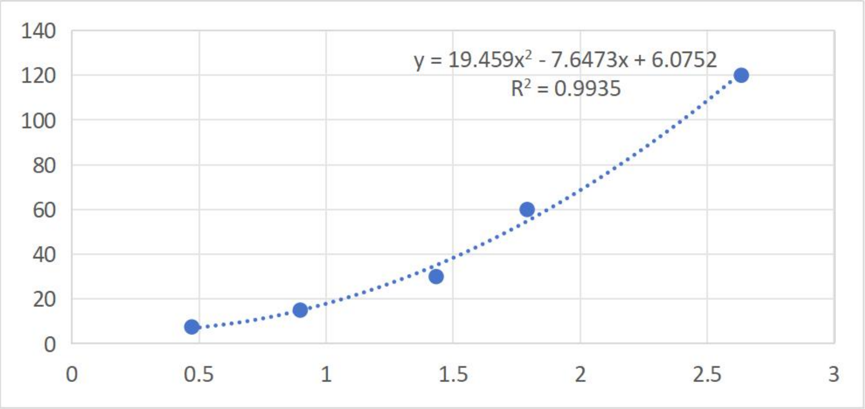


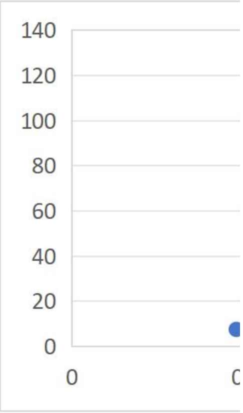


| Sample I | IOD （450r | Blank Cor | Dilution Fact | Concentration | | Blank Cor |
|---|---|---|---|---|---|---|
| | 0.839 | 0.764 | 11.59080326 | 57.95401632 | | 0.771 |
| | 0.756 | 0.681 | 9.891713999 | 49.45857 | | 0.688 |
| | 0.787 | 0.712 | 10.4949457 | 52.47472848 | | 0.719 |
| | 0.878 | 0.803 | 12.48175643 | 62.40878216 | | 0.81 |
| | 0.712 | 0.637 | 9.099728871 | 45.49864436 | | 0.644 |
| | 0.726 | 0.651 | 9.343551359 | 46.7177568 | | 0.658 |
| | 0.78 | 0.705 | 10.35546298 | 51.77731488 | | 0.712 |
| | 0.824 | 0.749 | 11.26389076 | 56.3194538 | | 0.756 |
| | 0.692 | 0.617 | 8.764643151 | 43.82321576 | | 0.624 |
| | 0.678 | 0.603 | 8.539345631 | 42.69672816 | | 0.61 |
| | 0.737 | 0.662 | 9.540477396 | 47.70238698 | | 0.669 |
| | 0.733 | 0.658 | 9.468323076 | 47.34161538 | | 0.665 |
| | 0.512 | 0.437 | 6.449395671 | 32.24697836 | | 0.444 |
| | 0.524 | 0.449 | 6.564516159 | 32.8225808 | | 0.456 |
| | 0.562 | 0.487 | 6.966036471 | 34.83018236 | | 0.494 |
| | 0.613 | 0.538 | 7.593243396 | 37.96621698 | | 0.545 |
| | 0.622 | 0.547 | 7.714434831 | 38.57217416 | | 0.554 |
| | 0.541 | 0.466 | 6.737196804 | 33.68598402 | | 0.473 |
| | 0.655 | 0.58 | 8.1857736 | 40.928868 | | 0.587 |
| | 0.587 | 0.512 | 7.260842496 | 36.30421248 | | 0.519 |
| | 0.554 | 0.479 | 6.876835719 | 34.3841786 | | 0.486 |
| | 0.55 | 0.475 | 6.833169375 | 34.16584688 | | 0.482 |
| | 0.627 | 0.552 | 7.783125536 | 38.91562768 | | 0.559 |
| | 0.67 | 0.595 | 8.414028975 | 42.07014488 | | 0.602 |
| | 0.573 | 0.498 | 7.092754436 | 35.46377218 | | 0.505 |
| | 0.521 | 0.446 | 6.535210644 | 32.67605322 | | 0.453 |
| | 0.58 | 0.505 | 7.175844975 | 35.87922488 | | 0.512 |
| | 0.564 | 0.489 | 6.988725839 | 34.9436292 | | 0.496 |
| | 0.652 | 0.577 | 8.141173311 | 40.70586656 | | 0.584 |

| | | | | |
|---|---|---|---|---|
| 0.533 | 0.458 | 6.654534276 | 33.27267138 | 0.465 |
| 0.578 | 0.503 | 7.151910231 | 35.75955116 | 0.51 |
| 0.528 | 0.453 | 6.604135031 | 33.02067516 | 0.46 |
| 0.557 | 0.482 | 6.909994116 | 34.54997058 | 0.489 |
| 0.554 | 0.479 | 6.876835719 | 34.3841786 | 0.486 |
| 0.652 | 0.577 | 8.141173311 | 40.70586656 | 0.584 |
| 0.526 | 0.451 | 6.584247759 | 32.9212388 | 0.458 |
| 0.647 | 0.572 | 8.067617856 | 40.33808928 | 0.579 |
| 0.652 | 0.577 | 8.141173311 | 40.70586656 | 0.584 |
| 0.563 | 0.488 | 6.977361696 | 34.88680848 | 0.495 |
| 0.56 | 0.485 | 6.943502775 | 34.71751388 | 0.492 |
| 0.437 | 0.362 | 5.856862596 | 29.28431298 | 0.369 |
| 0.633 | 0.558 | 7.866838676 | 39.33419338 | 0.565 |
| 0.613 | 0.538 | 7.593243396 | 37.96621698 | 0.545 |
| 0.651 | 0.576 | 8.126384384 | 40.63192192 | 0.583 |
| 0.663 | 0.588 | 8.306420096 | 41.53210048 | 0.595 |
| 0.531 | 0.456 | 6.634257824 | 33.17128912 | 0.463 |

| | | | | |
|---|---|---|---|---|
| 0.539 | 0.974 | 1.515 | 1.861 | 2.718 |
| 0.471 | 0.906 | 1.447 | 1.793 | 2.65 |
| 7.5 | 15 | 30 | 60 | 120 |

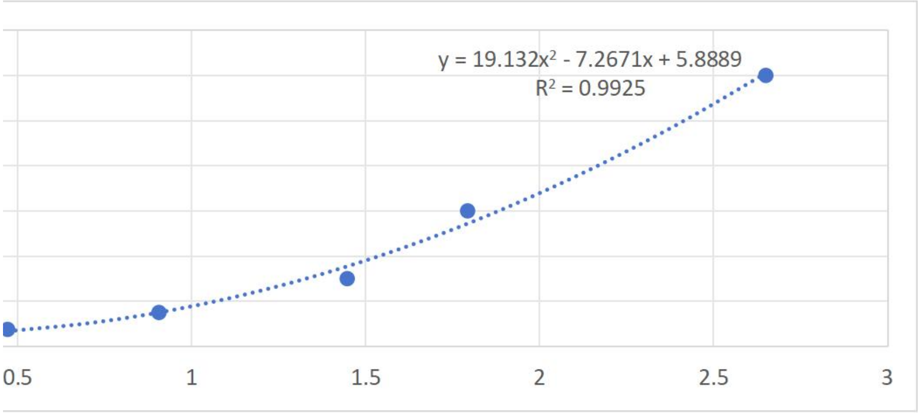


| Dilution Fact | Concentration |
|---|---|
| 11.65881111 | 58.29405556 |
| 9.945152608 | 49.72576304 |
| 10.55435295 | 52.77176476 |
| 12.5550542 | 62.775271 |
| 9.143616752 | 45.71808376 |
| 9.390615448 | 46.95307724 |
| 10.41357741 | 52.06788704 |
| 11.32959915 | 56.64799576 |
| 8.803771232 | 44.01885616 |
| 8.5749862 | 42.874931 |
| 9.589947152 | 47.94973576 |
| 9.5169272 | 47.584636 |
| 6.433913552 | 32.16956776 |
| 6.553333952 | 32.76666976 |
| 6.967849352 | 34.83924676 |
| 7.6110128 | 38.055064 |
| 7.734843512 | 38.67421756 |
| 6.731944928 | 33.65972464 |
| 8.215406408 | 41.07703204 |
| 7.270689752 | 36.35344876 |
| 6.875991272 | 34.37995636 |
| 6.830980568 | 34.15490284 |
| 7.804977592 | 39.02488796 |
| 8.447619128 | 42.23809564 |
| 7.0981528 | 35.490764 |
| 6.522962288 | 32.61481144 |
| 7.183483808 | 35.91741904 |
| 6.991196512 | 34.95598256 |
| 8.169996992 | 40.84998496 |

| | |
|---|---|
| 6.6465152 | 33.232576 |
| 7.1589122 | 35.794561 |
| 6.5943652 | 32.971826 |
| 6.910151072 | 34.55075536 |
| 6.875991272 | 34.37995636 |
| 8.169996992 | 40.84998496 |
| 6.573773048 | 32.86886524 |
| 8.095079912 | 40.47539956 |
| 8.169996992 | 40.84998496 |
| 6.9795038 | 34.897519 |
| 6.944655248 | 34.72327624 |
| 5.812372352 | 29.06186176 |
| 7.8904012 | 39.452006 |
| 7.6110128 | 38.055064 |
| 8.154937048 | 40.77468524 |
| 8.3381818 | 41.690909 |
| 6.625540408 | 33.12770204 |

| | | | | | |
|---|---|---|---|---|---|
| 0.066 | 0.458 | 0.854 | 1.362 | 1.763 | 2.605 |
| 0 | 0.392 | 0.788 | 1.296 | 1.697 | 2.539 |
| 0 | 7.5 | 15 | 30 | 60 | 120 |

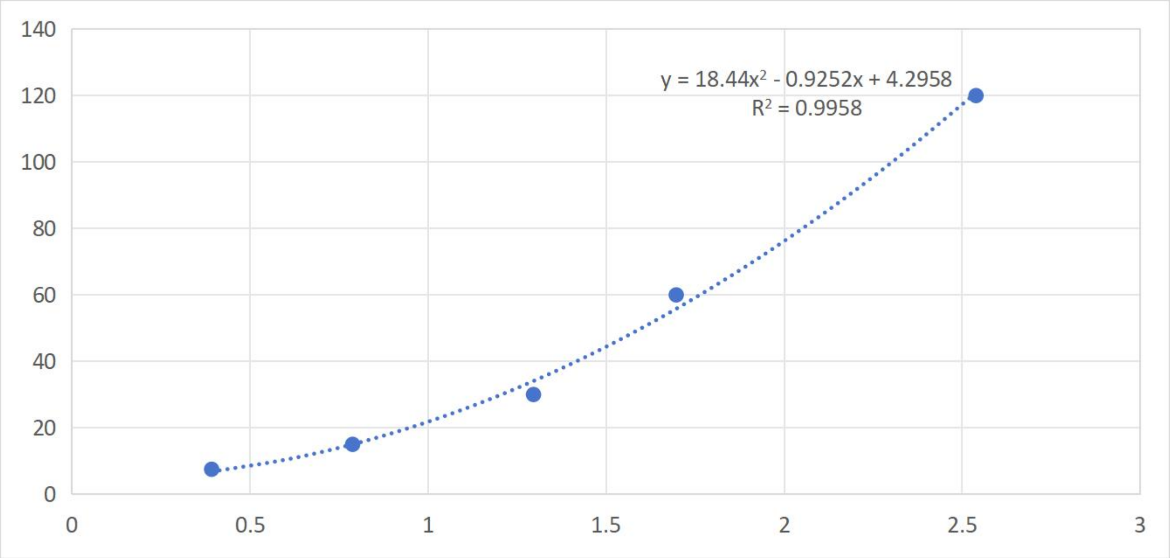


| Sample ID | OD (450nm) | Blank Corrected | Dilution Factor | Concentration |
|---|---|---|---|---|
| 7 | 0.816 | 0.75 | 13.9744 | 69.872 |
| 8 | 0.733 | 0.667 | 11.88244476 | 59.4122238 |
| 9 | 0.772 | 0.706 | 12.83376864 | 64.1688432 |
| 10 | 0.873 | 0.807 | 15.55819516 | 77.7909758 |
| 11 | 0.679 | 0.613 | 10.65783276 | 53.2891638 |
| 12 | 0.691 | 0.625 | 10.920675 | 54.603375 |
| 19 | 0.753 | 0.687 | 12.36329596 | 61.8164798 |
| 20 | 0.8 | 0.734 | 13.55136384 | 67.7568192 |
| 21 | 0.654 | 0.588 | 10.12730176 | 50.6365088 |
| 22 | 0.659 | 0.593 | 10.23156396 | 51.1578198 |
| 23 | 0.701 | 0.635 | 11.143767 | 55.718835 |
| 24 | 0.68 | 0.614 | 10.67953344 | 53.3976672 |
| 25 | 0.503 | 0.437 | 7.41295596 | 37.0647798 |
| 26 | 0.51 | 0.444 | 7.52019904 | 37.6009952 |
| 27 | 0.588 | 0.522 | 8.83745056 | 44.1872528 |
| 28 | 0.597 | 0.531 | 9.00387964 | 45.0193982 |
| 29 | 0.617 | 0.551 | 9.38441724 | 46.9220862 |
| 30 | 0.536 | 0.47 | 7.934352 | 39.67176 |
| 31 | 0.631 | 0.565 | 9.659571 | 48.297855 |
| 32 | 0.563 | 0.497 | 8.39082156 | 41.9541078 |
| 33 | 0.539 | 0.473 | 7.98374316 | 39.9187158 |
| 34 | 0.533 | 0.467 | 7.88529276 | 39.4264638 |
| 35 | 0.608 | 0.542 | 9.21134976 | 46.0567488 |
| 36 | 0.647 | 0.581 | 9.98288364 | 49.9144182 |
| 37 | 0.569 | 0.503 | 8.49591036 | 42.4795518 |
| 38 | 0.504 | 0.438 | 7.42816576 | 37.1408288 |
| 39 | 0.576 | 0.51 | 8.620192 | 43.10096 |
| 40 | 0.551 | 0.485 | 8.184627 | 40.923135 |
| 41 | 0.644 | 0.578 | 9.92154336 | 49.6077168 |
| 42 | 0.528 | 0.462 | 7.80426496 | 39.0213248 |

| | | | | |
|---|---|---|---|---|
| 43 | 0.646 | 0.58 | 9.9624 | 49.812 |
| 44 | 0.554 | 0.488 | 8.23567776 | 41.1783888 |
| 45 | 0.641 | 0.575 | 9.860535 | 49.302675 |
| 46 | 0.623 | 0.557 | 9.50145516 | 47.5072758 |
| 47 | 0.608 | 0.542 | 9.21134976 | 46.0567488 |
| 48 | 0.606 | 0.54 | 9.173296 | 45.86648 |
| 49 | 0.636 | 0.57 | 9.759592 | 48.79796 |
| 50 | 0.649 | 0.583 | 10.02396156 | 50.1198078 |
| 51 | 0.546 | 0.48 | 8.10028 | 40.5014 |
| 52 | 0.557 | 0.491 | 8.28706044 | 41.4353022 |
| 53 | 0.531 | 0.465 | 7.852771 | 39.263855 |
| 54 | 0.629 | 0.563 | 9.61982076 | 48.0991038 |
| 55 | 0.604 | 0.538 | 9.13538976 | 45.6769488 |
| 56 | 0.646 | 0.58 | 9.9624 | 49.812 |
| 57 | 0.652 | 0.586 | 10.08585504 | 50.4292752 |
| 58 | 0.524 | 0.458 | 7.74010656 | 38.7005328 |

| | | | | | |
|---|---|---|---|---|---|
| 0.078 | 0.555 | 0.994 | 1.463 | 1.854 | 2.711 |
| 0 | 0.477 | 0.916 | 1.385 | 1.776 | 2.633 |
| 0 | 7.5 | 15 | 30 | 60 | 120 |

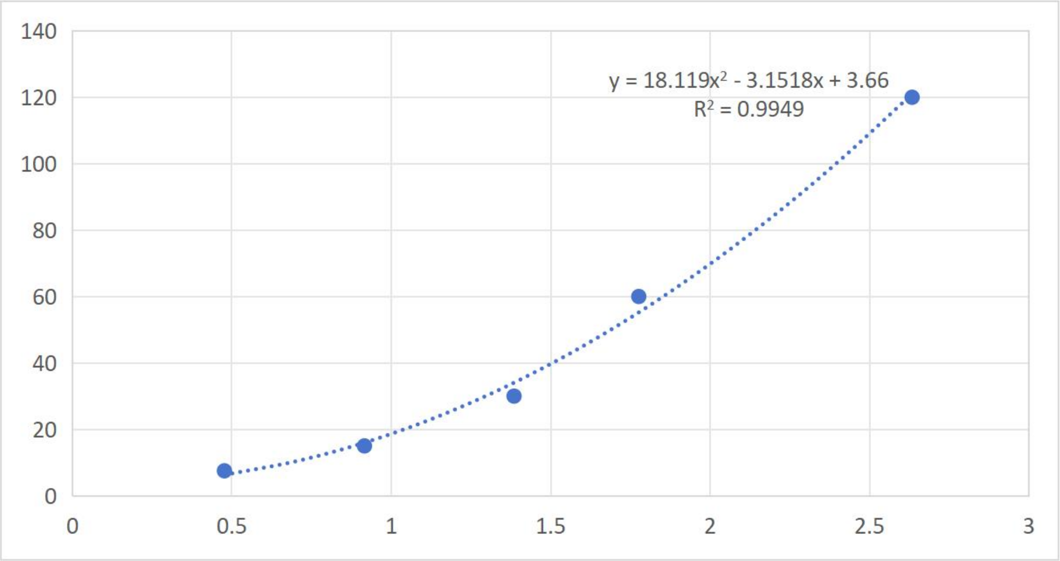


| Sample ID | OD (450nm) | Blank Corrected | Dilution Factor | Concentration |
|---|---|---|---|---|
| 7 | 0.816 | 0.738 | 13.65623776 | 68.2811888 |
| 8 | 0.733 | 0.655 | 11.601015 | 58.005075 |
| 9 | 0.772 | 0.694 | 12.53507904 | 62.6753952 |
| 10 | 0.873 | 0.795 | 15.214807 | 76.074035 |
| 11 | 0.679 | 0.601 | 10.40030124 | 52.0015062 |
| 12 | 0.691 | 0.613 | 10.65783276 | 53.2891638 |
| 19 | 0.753 | 0.675 | 12.073015 | 60.365075 |
| 20 | 0.8 | 0.722 | 13.24028256 | 66.2014128 |
| 21 | 0.654 | 0.576 | 9.88083424 | 49.4041712 |
| 22 | 0.659 | 0.581 | 9.98288364 | 49.9144182 |
| 23 | 0.701 | 0.623 | 10.87649916 | 54.3824958 |
| 24 | 0.68 | 0.602 | 10.42155936 | 52.1077968 |

| | | | | |
|---|---|---|---|---|
| 25 | 0.503 | 0.425 | 5.593229375 | 27.96614688 |
| 26 | 0.51 | 0.432 | 5.679862656 | 28.39931328 |
| 27 | 0.588 | 0.51 | 6.7653339 | 33.8266695 |
| 28 | 0.597 | 0.519 | 6.904767759 | 34.5238388 |
| 29 | 0.617 | 0.539 | 7.225129799 | 36.125649 |
| 30 | 0.536 | 0.458 | 6.017189516 | 30.08594758 |
| 31 | 0.631 | 0.553 | 7.458007871 | 37.29003936 |
| 32 | 0.563 | 0.485 | 6.393418775 | 31.96709388 |
| 33 | 0.539 | 0.461 | 6.057688199 | 30.288441 |
| 34 | 0.533 | 0.455 | 5.977016975 | 29.88508488 |
| 35 | 0.608 | 0.53 | 7.0791731 | 35.3958655 |
| 36 | 0.647 | 0.569 | 7.732851359 | 38.6642568 |
| 37 | 0.569 | 0.491 | 6.480612839 | 32.4030642 |
| 38 | 0.504 | 0.426 | 5.605496844 | 28.02748422 |
| 39 | 0.576 | 0.498 | 6.583988076 | 32.91994038 |
| 40 | 0.551 | 0.473 | 6.222944351 | 31.11472176 |
| 41 | 0.644 | 0.566 | 7.680611564 | 38.40305782 |
| 42 | 0.528 | 0.45 | 5.9107875 | 29.5539375 |
| 43 | 0.646 | 0.568 | 7.715401856 | 38.57700928 |
| 44 | 0.554 | 0.476 | 6.265073744 | 31.32536872 |
| 45 | 0.641 | 0.563 | 7.628697911 | 38.14348956 |
| 46 | 0.623 | 0.545 | 7.324064975 | 36.62032488 |
| 47 | 0.608 | 0.53 | 7.0791731 | 35.3958655 |
| 48 | 0.606 | 0.528 | 7.047136896 | 35.23568448 |
| 49 | 0.636 | 0.558 | 7.542899916 | 37.71449958 |
| 50 | 0.649 | 0.571 | 7.767859079 | 38.8392954 |
| 51 | 0.546 | 0.468 | 6.153453456 | 30.76726728 |
| 52 | 0.557 | 0.479 | 6.307529279 | 31.5376464 |
| 53 | 0.531 | 0.453 | 5.950416471 | 29.75208236 |
| 54 | 0.629 | 0.551 | 7.424304719 | 37.1215236 |
| 55 | 0.604 | 0.526 | 7.015245644 | 35.07622822 |
| 56 | 0.646 | 0.568 | 7.715401856 | 38.57700928 |
| 57 | 0.652 | 0.574 | 7.820642444 | 39.10321222 |
| 58 | 0.524 | 0.446 | 5.858456204 | 29.29228102 |

| | | |
|---|---|---|
| 61.48858 | 58.12404 | 62.89640305 |
| 63.13696 | 49.59217 | 57.14592439 |
| 58.6334 | 52.62325 | 58.22625596 |
| 67.43348 | 62.59203 | 68.98600235 |
| 45.95318 | 45.60836 | 48.06896134 |
| 46.91348 | 46.83542 | 49.2317205 |
| 52.58124 | 51.9226 | 55.19820534 |
| 58.02306 | 56.48372 | 60.49529918 |
| 43.86207 | 43.92104 | 45.93448068 |
| 43.45094 | 42.78583 | 45.5909616 |
| 48.65022 | 47.82606 | 50.50898075 |
| 47.45214 | 47.46313 | 49.22266664 |
| 29.42045 | 32.20827 | 31.38139538 |
| 28.47436 | 32.79463 | 31.42304781 |
| 37.94341 | 34.83471 | 37.26169369 |
| 27.77877 | 38.01064 | 35.18700823 |
| 37.1015 | 38.6232 | 39.08285284 |
| 30.79717 | 33.67285 | 33.11629211 |
| 40.0518 | 41.00295 | 41.28290065 |
| 33.9412 | 36.32883 | 35.74354306 |
| 31.45981 | 34.38207 | 33.64848687 |
| 30.87948 | 34.16037 | 33.23187699 |
| 37.94341 | 38.97026 | 39.21332345 |
| 40.63924 | 42.15412 | 42.36089829 |
| 33.50451 | 35.47727 | 35.47436058 |
| 29.18193 | 32.64543 | 31.47050732 |
| 32.72777 | 35.89832 | 35.5455143 |
| 34.11691 | 34.94981 | 35.02854777 |
| 31.71075 | 40.77793 | 38.83135293 |
| 30.14398 | 33.25262 | 32.5614117 |
| 40.83623 | 35.77706 | 40.26926365 |
| 32.98535 | 32.99625 | 34.0778272 |
| 40.0518 | 34.55036 | 39.44175 |
| 36.18008 | 34.38207 | 37.54198234 |
| 37.00869 | 40.77793 | 39.50430711 |
| 37.28755 | 32.89505 | 36.91122866 |
| 40.34486 | 40.40674 | 41.33594336 |
| 40.83623 | 40.77793 | 42.03123586 |
| 32.64221 | 34.89216 | 34.38956773 |
| 33.07151 | 34.7204 | 34.75945924 |
| 30.46939 | 29.17309 | 31.38348266 |
| 38.98866 | 39.3931 | 40.33069238 |
| 36.73116 | 38.01064 | 38.37279475 |
| 41.13283 | 40.7033 | 42.01021218 |
| 29.18193 | 41.6115 | 38.51989386 |
| 41.83006 | 33.1495 | 36.32532189 |

Report
FT4 (14)

| Group | Mean | Std. Deviation |
|---|---|---|
| 1 | 54.292155 | 7.4025367421174 |
| 2 | 35.658056 | 3.3779967116097 |
| 3 | 37.950310 | 3.1161089719024 |
| Total | 41.316431 | 9.0926645840535 |

Tests of Normality

| | Group | Kolmogorov-Smirnova | | | Shapiro-Wilk | | |
|---|---|---|---|---|---|---|---|
| | | Statistic | df | Sig. | Statistic | df | Sig. |
| FT4 (14) | 1 | 0.195 | 12 | .200* | 0.929 | 12 | 0.372 |
| | 2 | 0.157 | 18 | .200* | 0.935 | 18 | 0.234 |
| | 3 | 0.121 | 16 | .200* | 0.951 | 16 | 0.512 |

* This is a lower bound of the true significance.
a Lilliefors Significance Correction

ANOVA
FT4 (14)

| | Sum of Sq | df | Mean Squa | F | Sig. |
|---|---|---|---|---|---|
| Between G | 2778.035 | 2 | 1389.017 | 63.378 | 0 |
| Within Gr | 942.41 | 43 | 21.917 | | |
| Total | 3720.445 | 45 | | | |

Robust Tests of Equality of Means
FT4 (14)

| | Statistic | df1 | df2 | Sig. |
|---|---|---|---|---|
| Welch | 32.451 | 2 | 23.162 | 0 |

a Asymptotically F distributed.

Multiple Comparisons
Dependent Variable: FT4 (14)
Games-Howell

| (I) Group | (J) Group | Mean Diff | Std. Erro | Sig. | 95% Confidence Interval | |
|---|---|---|---|---|---|---|
| | | | | | Lower Bou | Upper Bound |
| 1 | 2 | 18.634098 | 2.2804383 | 0 | 12.669924 | 24.598273491827 |
| | 3 | 16.341844 | 2.2744990 | 0 | 10.385795 | 22.297894255616 |
| 2 | 1 | -18.63409 | 2.2804383 | 0 | -24.5983 | -12.6699 |
| | 3 | -2.29225 | 1.1139210 | 0.115 | -5.02979 | 0.445283 |
| 3 | 1 | -16.34184 | 2.2744990 | 0 | -22.2979 | -10.3858 |
| | 2 | 2.2922538 | 1.1139210 | 0.115 | -0.44528 | 5.029791 |

* The mean difference is significant at the 0.05 level.

| | | | | | |
|---|---|---|---|---|---|
| 0.067 | 0.385 | 0.814 | 1.534 | 2.015 | 2.639 |
| 0 | 0.318 | 0.747 | 1.467 | 1.948 | 2.572 |
| | 1.25 | 2.5 | 5 | 10 | 20 |

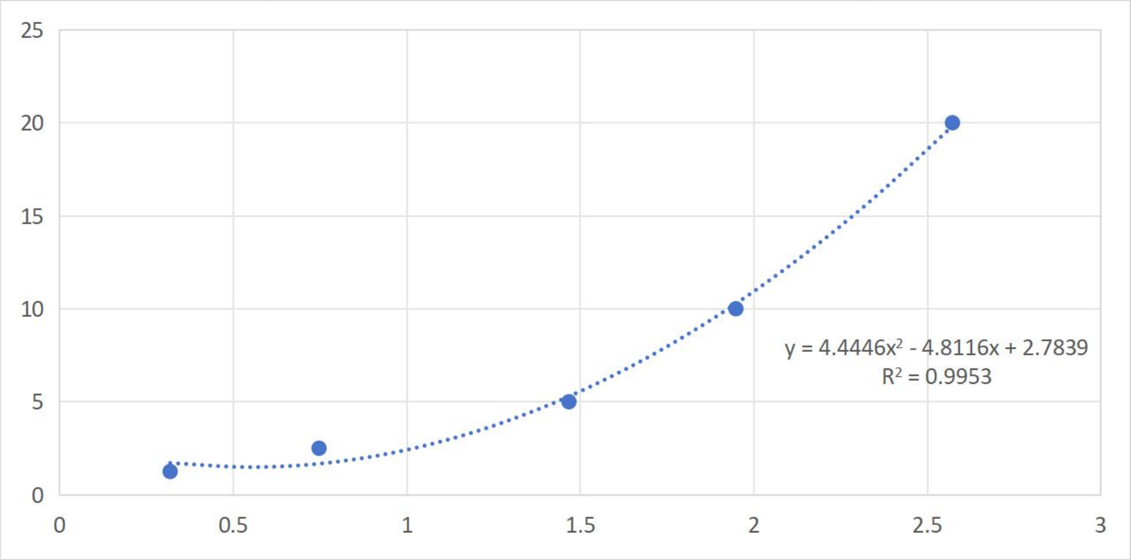


| Sample | IIOD （450nm） | Blank Correct | Dilution Fact | Concentration |
|---|---|---|---|---|
| 7 | 0.878 | 0.811 | 1.804999157 | 9.024995783 |
| 8 | 0.905 | 0.838 | 1.872972882 | 9.364864412 |
| 9 | 0.884 | 0.817 | 1.819544409 | 9.097722047 |
| 10 | 0.783 | 0.716 | 1.617345258 | 8.086726288 |
| 11 | 0.921 | 0.854 | 1.916311494 | 9.581557468 |
| 12 | 0.832 | 0.765 | 1.704117035 | 8.520585175 |
| 19 | 0.859 | 0.792 | 1.761050374 | 8.805251872 |
| 20 | 0.763 | 0.696 | 1.588061754 | 7.940308768 |
| 21 | 0.891 | 0.824 | 1.83691833 | 9.184591648 |
| 22 | 0.916 | 0.849 | 1.902523725 | 9.512618623 |
| 23 | 0.822 | 0.755 | 1.684675115 | 8.423375575 |
| 24 | 0.933 | 0.866 | 1.950308838 | 9.751544188 |
| 25 | 0.998 | 0.931 | 2.156706341 | 10.7835317 |
| 26 | 1.011 | 0.944 | 2.202492666 | 11.01246333 |
| 27 | 0.982 | 0.915 | 2.102416235 | 10.51208118 |
| 28 | 1.035 | 0.968 | 2.29096807 | 11.45484035 |
| 29 | 1.088 | 1.021 | 2.504489669 | 12.52244834 |
| 30 | 0.951 | 0.884 | 2.003704938 | 10.01852469 |
| 31 | 1.054 | 0.987 | 2.364642337 | 11.82321169 |
| 32 | 0.935 | 0.868 | 1.95609951 | 9.780497552 |
| 33 | 1.001 | 0.934 | 2.167139078 | 10.83569539 |
| 34 | 1.163 | 1.096 | 2.849311034 | 14.24655517 |
| 35 | 1.027 | 0.96 | 2.26090736 | 11.3045368 |
| 36 | 1.022 | 0.955 | 2.242408315 | 11.21204158 |
| 37 | 0.965 | 0.898 | 2.047226418 | 10.23613209 |
| 38 | 1.015 | 0.948 | 2.216882998 | 11.08441499 |
| 39 | 0.990 | 0.923 | 2.129276833 | 10.64638417 |
| 40 | 1.065 | 0.998 | 2.408762578 | 12.04381289 |
| 41 | 1.070 | 1.003 | 2.429172801 | 12.14586401 |
| 42 | 1.055 | 0.988 | 2.368608822 | 11.84304411 |
| 43 | 1.088 | 1.021 | 2.504489669 | 12.52244834 |

| | | | | |
|---|---|---|---|---|
| 44 | 0.969 | 0.902 | 2.059981138 | 10.29990569 |
| 45 | 0.941 | 0.874 | 1.97368487 | 9.868424348 |
| 46 | 1.132 | 1.065 | 2.700722435 | 13.50361218 |
| 47 | 0.980 | 0.913 | 2.095789977 | 10.47894989 |
| 48 | 0.996 | 0.929 | 2.149795629 | 10.74897814 |
| 49 | 1.023 | 0.956 | 2.246090346 | 11.23045173 |
| 50 | 0.963 | 0.896 | 2.040902394 | 10.20451197 |
| 51 | 1.055 | 0.988 | 2.368608822 | 11.84304411 |
| 52 | 0.979 | 0.912 | 2.092490182 | 10.46245091 |
| 53 | 0.943 | 0.876 | 1.97961777 | 9.898088848 |
| 54 | 0.986 | 0.919 | 2.115775421 | 10.5788771 |
| 55 | 0.964 | 0.897 | 2.044059961 | 10.22029981 |
| 56 | 0.984 | 0.917 | 2.109078049 | 10.54539025 |
| 57 | 1.082 | 1.015 | 2.479064035 | 12.39532018 |
| 58 | 1.012 | 0.945 | 2.206076915 | 11.03038458 |

| | | | | | |
|---|---|---|---|---|---|
| 0.063 | 0.416 | 0.998 | 1.544 | 2.001 | 2.58 |
| 0 | 0.353 | 0.935 | 1.481 | 1.938 | 2.517 |
| | 1.25 | 2.5 | 5 | 10 | 20 |

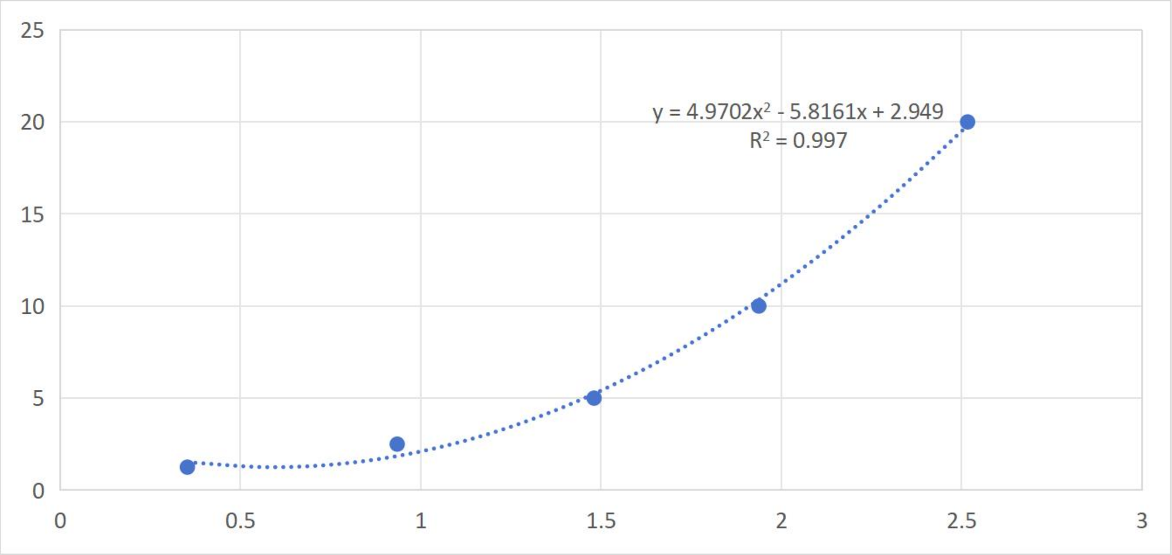


| Sample | IIOD （450nm） | Blank Correct | Dilution Fact | Concentration |
|---|---|---|---|---|
| 7 | 0.878 | 0.815 | 1.510209595 | 7.551047975 |
| 8 | 0.905 | 0.842 | 1.575536673 | 7.877683364 |
| 9 | 0.884 | 0.821 | 1.524100478 | 7.620502391 |
| 10 | 0.783 | 0.72 | 1.33795968 | 6.6897984 |
| 11 | 0.921 | 0.858 | 1.617668513 | 8.088342564 |
| 12 | 0.832 | 0.769 | 1.415601542 | 7.078007711 |
| 19 | 0.859 | 0.796 | 1.468582643 | 7.342913216 |
| 20 | 0.763 | 0.7 | 1.313128 | 6.56564 |
| 21 | 0.891 | 0.828 | 1.540758797 | 7.703793984 |
| 22 | 0.916 | 0.853 | 1.604228952 | 8.021144759 |
| 23 | 0.822 | 0.759 | 1.397817886 | 6.989089431 |
| 24 | 0.933 | 0.87 | 1.65093738 | 8.2546869 |

| | | | | |
|---|---|---|---|---|
| 25 | 1.128 | 1.065 | 2.392178595 | 11.96089298 |
| 26 | 1.131 | 1.068 | 2.406534605 | 12.03267302 |
| 27 | 1.082 | 1.019 | 2.183255942 | 10.91627971 |
| 28 | 1.095 | 1.032 | 2.240167085 | 11.20083542 |
| 29 | 1.088 | 1.025 | 2.209313875 | 11.04656938 |
| 30 | 1.051 | 0.988 | 2.054324109 | 10.27162054 |
| 31 | 1.054 | 0.991 | 2.066383886 | 10.33191943 |
| 32 | 0.935 | 0.872 | 1.656621357 | 8.283106784 |
| 33 | 1.001 | 0.938 | 1.866498849 | 9.332494244 |
| 34 | 1.163 | 1.1 | 2.565232 | 12.82616 |
| 35 | 1.027 | 0.964 | 1.961066579 | 9.805332896 |
| 36 | 1.022 | 0.959 | 1.942358606 | 9.711793031 |
| 37 | 1.065 | 1.002 | 2.111368481 | 10.5568424 |
| 38 | 1.015 | 0.952 | 1.916584941 | 9.582924704 |
| 39 | 1.099 | 1.036 | 2.258016179 | 11.2900809 |
| 40 | 1.065 | 1.002 | 2.111368481 | 10.5568424 |
| 41 | 1.070 | 1.007 | 2.13221364 | 10.6610682 |
| 42 | 1.055 | 0.992 | 2.070423693 | 10.35211846 |
| 43 | 1.088 | 1.025 | 2.209313875 | 11.04656938 |
| 44 | 0.969 | 0.906 | 1.759332487 | 8.796662436 |
| 45 | 0.941 | 0.878 | 1.673911857 | 8.369559284 |
| 46 | 1.132 | 1.069 | 2.411339822 | 12.05669911 |
| 47 | 0.980 | 0.917 | 1.795022808 | 8.975114039 |
| 48 | 0.996 | 0.933 | 1.849083128 | 9.245415639 |
| 49 | 1.023 | 0.96 | 1.94608032 | 9.7304016 |
| 50 | 1.019 | 0.956 | 1.931253107 | 9.656265536 |
| 51 | 1.055 | 0.992 | 2.070423693 | 10.35211846 |
| 52 | 1.027 | 0.964 | 1.961066579 | 9.805332896 |
| 53 | 1.043 | 0.98 | 2.02260208 | 10.1130104 |
| 54 | 1.026 | 0.963 | 1.957305104 | 9.786525519 |
| 55 | 0.964 | 0.901 | 1.74350723 | 8.717536151 |
| 56 | 0.984 | 0.921 | 1.808299318 | 9.041496591 |
| 57 | 1.082 | 1.019 | 2.183255942 | 10.91627971 |
| 58 | 1.012 | 0.949 | 1.90568819 | 9.528440951 |

| | | | | | |
|---|---|---|---|---|---|
| 0.066 | 0.403 | 0.946 | 1.54 | 2.011 | 2.622 |
| 0 | 0.337 | 0.88 | 1.474 | 1.945 | 2.556 |
| 0 | 1.25 | 2.5 | 5 | 10 | 20 |

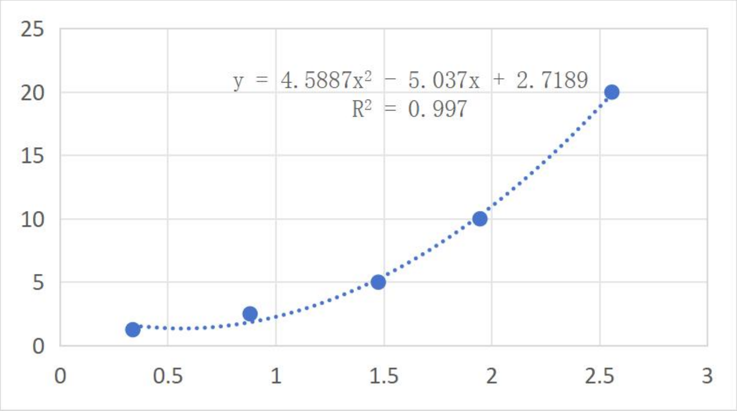


| Sample ID | OD (450nm) | Blank Corrected | Dilution Factor | Concentration |
|---|---|---|---|---|
| 7 | 0.869 | 0.803 | 1.633024058 | 8.165120292 |
| 8 | 0.898 | 0.832 | 1.704524269 | 8.522621344 |
| 9 | 0.882 | 0.816 | 1.664121427 | 8.320607136 |
| 10 | 0.788 | 0.722 | 1.474201891 | 7.371009454 |
| 11 | 0.917 | 0.851 | 1.755554129 | 8.777770644 |
| 12 | 0.836 | 0.77 | 1.56105023 | 7.80525115 |
| 19 | 0.862 | 0.796 | 1.616921739 | 8.084608696 |
| 20 | 0.772 | 0.706 | 1.449951273 | 7.249756366 |
| 21 | 0.886 | 0.82 | 1.67400188 | 8.3700094 |
| 22 | 0.913 | 0.847 | 1.744535678 | 8.722678392 |
| 23 | 0.811 | 0.745 | 1.513178218 | 7.565891088 |
| 24 | 0.928 | 0.862 | 1.786612003 | 8.933060014 |
| 25 | 1.077 | 1.011 | 2.316699633 | 11.58349816 |
| 26 | 1.083 | 1.017 | 2.342312934 | 11.71156467 |
| 27 | 1.086 | 1.02 | 2.35524348 | 11.7762174 |
| 28 | 1.042 | 0.976 | 2.173873491 | 10.86936746 |
| 29 | 1.066 | 1 | 2.2706 | 11.353 |
| 30 | 1.098 | 1.032 | 2.407791629 | 12.03895814 |
| 31 | 1.041 | 0.975 | 2.169957938 | 10.84978969 |
| 32 | 0.938 | 0.872 | 1.815810061 | 9.079050304 |
| 33 | 1.007 | 0.941 | 2.042289665 | 10.21144832 |
| 34 | 1.155 | 1.089 | 2.675442693 | 13.37721346 |
| 35 | 1.032 | 0.966 | 2.135130937 | 10.67565469 |
| 36 | 1.031 | 0.965 | 2.131307158 | 10.65653579 |
| 37 | 1.061 | 0.995 | 2.250012718 | 11.25006359 |
| 38 | 1.132 | 1.066 | 2.563854777 | 12.81927389 |
| 39 | 1.094 | 1.028 | 2.390128741 | 11.9506437 |
| 40 | 1.052 | 0.986 | 2.213533785 | 11.06766893 |
| 41 | 1.065 | 0.999 | 2.266464189 | 11.33232094 |
| 42 | 1.049 | 0.983 | 2.201539334 | 11.00769667 |
| 43 | 1.074 | 1.008 | 2.304016877 | 11.52008438 |
| 44 | 0.972 | 0.906 | 1.921948153 | 9.609740766 |
| 45 | 0.933 | 0.867 | 1.801096314 | 9.005481572 |

| | | | | |
|---|---|---|---|---|
| 46 | 1.129 | 1.063 | 2.54965775 | 12.74828875 |
| 47 | 0.986 | 0.92 | 1.96873568 | 9.8436784 |
| 48 | 1.002 | 0.936 | 2.024409715 | 10.12204858 |
| 49 | 1.017 | 0.951 | 2.078737869 | 10.39368934 |
| 50 | 1.072 | 1.006 | 2.295607593 | 11.47803797 |
| 51 | 1.044 | 0.978 | 2.181732131 | 10.90866065 |
| 52 | 1.062 | 0.996 | 2.254111819 | 11.2705591 |
| 53 | 1.051 | 0.985 | 2.209526458 | 11.04763229 |
| 54 | 0.998 | 0.932 | 2.010270949 | 10.05135474 |
| 55 | 0.969 | 0.903 | 1.912156278 | 9.560781392 |
| 56 | 0.992 | 0.926 | 1.989338121 | 9.946690606 |
| 57 | 1.074 | 1.008 | 2.304016877 | 11.52008438 |
| 58 | 1.022 | 0.956 | 2.097306123 | 10.48653062 |

| | | | | | |
|---|---|---|---|---|---|
| 0.07 | 0.402 | 0.973 | 1.526 | 2.009 | 2.617 |
| 0 | 0.332 | 0.903 | 1.456 | 1.939 | 2.547 |
| 0 | 1.25 | 2.5 | 5 | 10 | 20 |

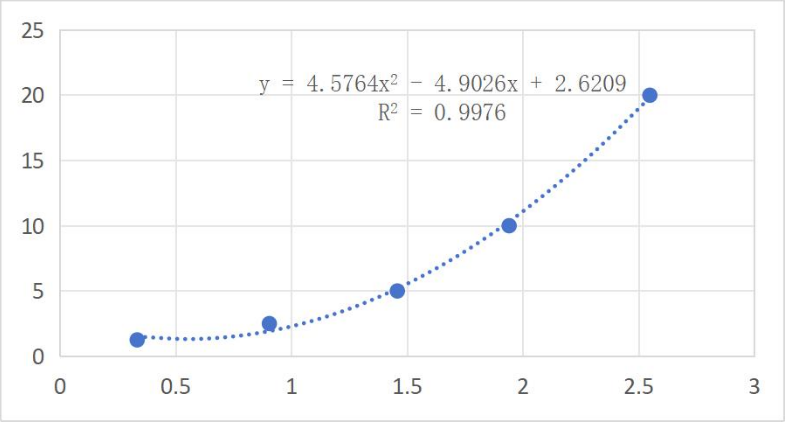


| Sample ID | OD (450nm) | Blank Corrected | Dilution Factor | Concentration |
|---|---|---|---|---|
| 7 | 0.869 | 0.799 | 1.625300936 | 8.126504682 |
| 8 | 0.898 | 0.828 | 1.699053818 | 8.495269088 |
| 9 | 0.882 | 0.812 | 1.657410682 | 8.287053408 |
| 10 | 0.788 | 0.718 | 1.460077234 | 7.300386168 |
| 11 | 0.917 | 0.847 | 1.751548348 | 8.757741738 |
| 12 | 0.836 | 0.766 | 1.550738558 | 7.753692792 |
| 19 | 0.862 | 0.792 | 1.60865177 | 8.043258848 |
| 20 | 0.772 | 0.702 | 1.434543026 | 7.172715128 |
| 21 | 0.886 | 0.816 | 1.667601798 | 8.338008992 |
| 22 | 0.913 | 0.843 | 1.740222284 | 8.701111418 |
| 23 | 0.811 | 0.741 | 1.500887688 | 7.504438442 |
| 24 | 0.928 | 0.858 | 1.78345013 | 8.917250648 |
| 25 | 1.077 | 1.007 | 2.324675644 | 11.62337822 |
| 26 | 1.083 | 1.013 | 2.350726012 | 11.75363006 |
| 27 | 1.086 | 1.016 | 2.363874758 | 11.81937379 |
| 28 | 1.042 | 0.972 | 2.179282298 | 10.89641149 |

| | | | | |
|---|---|---|---|---|
| 29 | 1.066 | 0.996 | 2.277772422 | 11.38886211 |
| 30 | 1.098 | 1.028 | 2.417293498 | 12.08646749 |
| 31 | 1.041 | 0.971 | 2.175292952 | 10.87646476 |
| 32 | 0.938 | 0.868 | 1.813412794 | 9.067063968 |
| 33 | 1.007 | 0.937 | 2.045101132 | 10.22550566 |
| 34 | 1.155 | 1.085 | 2.68903149 | 13.44515745 |
| 35 | 1.032 | 0.962 | 2.139800722 | 10.69900361 |
| 36 | 1.031 | 0.961 | 2.135902904 | 10.67951452 |
| 37 | 1.061 | 0.991 | 2.256818888 | 11.28409444 |
| 38 | 1.132 | 1.062 | 2.575804082 | 12.87902041 |
| 39 | 1.094 | 1.024 | 2.399340806 | 11.99670403 |
| 40 | 1.052 | 0.982 | 2.219679154 | 11.09839577 |
| 41 | 1.065 | 0.995 | 2.27356341 | 11.36781705 |
| 42 | 1.049 | 0.979 | 2.207463992 | 11.03731996 |
| 43 | 1.074 | 1.004 | 2.311774022 | 11.55887011 |
| 44 | 0.972 | 0.902 | 1.922132146 | 9.610660728 |
| 45 | 0.933 | 0.863 | 1.798317052 | 8.991585258 |
| 46 | 1.129 | 1.059 | 2.561392248 | 12.80696124 |
| 47 | 0.986 | 0.916 | 1.969974278 | 9.849871392 |
| 48 | 1.002 | 0.932 | 2.026847674 | 10.13423837 |
| 49 | 1.017 | 0.947 | 2.082294508 | 10.41147254 |
| 50 | 1.072 | 1.002 | 2.303218706 | 11.51609353 |
| 51 | 1.044 | 0.974 | 2.187288446 | 10.93644223 |
| 52 | 1.062 | 0.992 | 2.26099129 | 11.30495645 |
| 53 | 1.051 | 0.981 | 2.21559828 | 11.0779914 |
| 54 | 0.998 | 0.928 | 2.012409658 | 10.06204829 |
| 55 | 0.969 | 0.899 | 1.912113656 | 9.560568282 |
| 56 | 0.992 | 0.922 | 1.991027218 | 9.955136088 |
| 57 | 1.074 | 1.004 | 2.311774022 | 11.55887011 |
| 58 | 1.022 | 0.952 | 2.101234426 | 10.50617213 |

| | | | | | |
|---|---|---|---|---|---|
| 0.061 | 0.412 | 0.983 | 1.538 | 2.011 | 2.62 |
| 0 | 0.351 | 0.922 | 1.477 | 1.95 | 2.559 |
| | 1.25 | 2.5 | 5 | 10 | 20 |

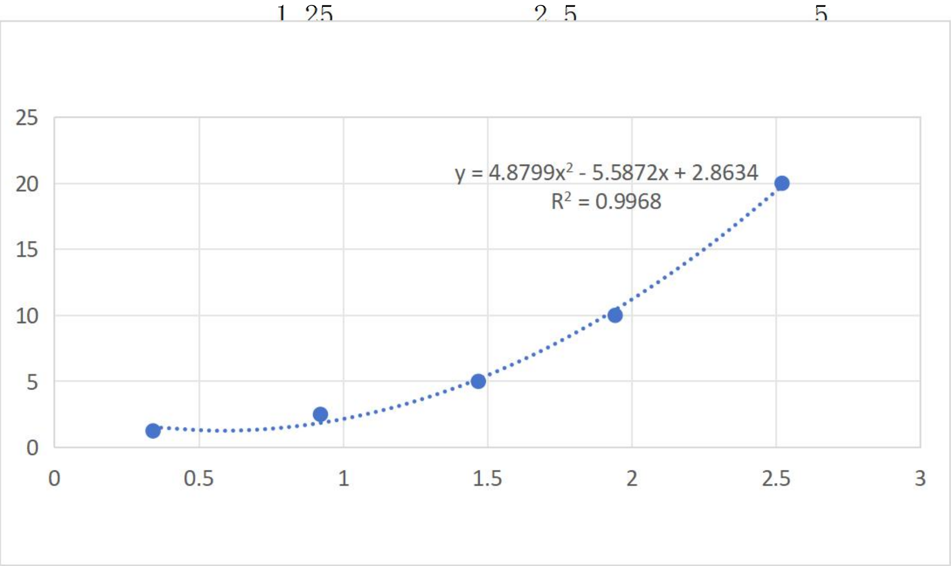


| Sample ID | OD (450nm) | Blank Corrected | Dilution Factor | Concentration |
|---|---|---|---|---|
| 7 | 0.872 | 0.811 | 1.602020834 | 8.010104169 |
| 8 | 0.886 | 0.825 | 1.635950125 | 8.179750625 |
| 9 | 0.89 | 0.829 | 1.64597669 | 8.229883449 |
| 10 | 0.785 | 0.724 | 1.431751133 | 7.158755664 |
| 11 | 0.92 | 0.859 | 1.725886082 | 8.629430409 |
| 12 | 0.824 | 0.763 | 1.499434408 | 7.497172041 |
| 19 | 0.853 | 0.792 | 1.558869299 | 7.794346496 |
| 20 | 0.778 | 0.717 | 1.421089784 | 7.105448921 |
| 21 | 0.879 | 0.818 | 1.618759207 | 8.093796036 |
| 22 | 0.910 | 0.849 | 1.698326058 | 8.491630289 |
| 23 | 0.802 | 0.741 | 1.459527042 | 7.297635209 |
| 24 | 0.926 | 0.865 | 1.742865405 | 8.714327025 |
| 25 | 1.097 | 1.036 | 2.366543069 | 11.83271534 |
| 26 | 1.089 | 1.028 | 2.331343555 | 11.65671778 |
| 27 | 1.081 | 1.02 | 2.29673512 | 11.4836756 |
| 28 | 1.065 | 1.004 | 2.229291485 | 11.14645742 |
| 29 | 1.072 | 1.011 | 2.258507154 | 11.29253577 |
| 30 | 1.151 | 1.09 | 2.61960018 | 13.0980009 |
| 31 | 1.033 | 0.972 | 2.101497155 | 10.50748578 |
| 32 | 0.944 | 0.883 | 1.795798264 | 8.978991321 |
| 33 | 1.001 | 0.94 | 1.98316008 | 9.9158004 |
| 34 | 1.139 | 1.078 | 2.561037895 | 12.80518948 |
| 35 | 1.026 | 0.965 | 2.074802805 | 10.37401403 |
| 36 | 1.040 | 0.979 | 2.12864405 | 10.64322025 |
| 37 | 1.103 | 1.042 | 2.393330599 | 11.966653 |
| 38 | 1.098 | 1.037 | 2.370984568 | 11.85492284 |
| 39 | 1.112 | 1.051 | 2.434135298 | 12.17067649 |
| 40 | 1.037 | 0.976 | 2.116954253 | 10.58477126 |
| 41 | 1.085 | 1.024 | 2.313965453 | 11.56982726 |
| 42 | 1.090 | 1.029 | 2.33571117 | 11.67855585 |

| | | | | |
|---|---|---|---|---|
| 43 | 1.057 | 0.996 | 2.196456285 | 10.98228142 |
| 44 | 0.968 | 0.907 | 1.871030152 | 9.355150761 |
| 45 | 0.937 | 0.876 | 1.774857693 | 8.874288464 |
| 46 | 1.114 | 1.053 | 2.4433046 | 12.216523 |
| 47 | 0.975 | 0.914 | 1.893974849 | 9.469874244 |
| 48 | 0.994 | 0.933 | 1.958534504 | 9.792672521 |
| 49 | 1.012 | 0.951 | 2.022771738 | 10.11385869 |
| 50 | 1.098 | 1.037 | 2.370984568 | 11.85492284 |
| 51 | 1.036 | 0.975 | 2.113076125 | 10.56538063 |
| 52 | 1.091 | 1.03 | 2.34008802 | 11.7004401 |
| 53 | 1.052 | 0.991 | 2.176234442 | 10.88117221 |
| 54 | 1.003 | 0.942 | 1.990279079 | 9.951395396 |
| 55 | 0.957 | 0.896 | 1.835888525 | 9.179442624 |
| 56 | 0.981 | 0.92 | 1.91400192 | 9.5700096 |
| 57 | 1.066 | 1.005 | 2.233437445 | 11.16718723 |
| 58 | 1.037 | 0.976 | 2.116954253 | 10.58477126 |

| | | | |
|---|---|---|---|
| 0.068 | 0.409 | 0.989 | 1.536 |
| 0 | 0.341 | 0.921 | 1.468 |
| | 1.25 | 2.5 | 5 |

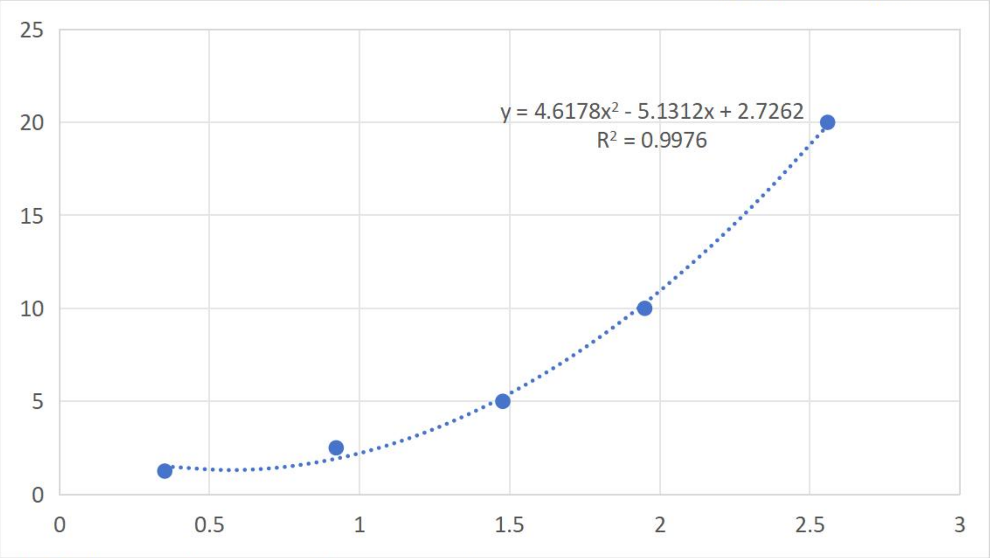


| Blank Corrected | Dilution Factor | Concentration |
|---|---|---|
| 0.804 | 1.525736638 | 7.628683192 |
| 0.818 | 1.558328608 | 7.791643038 |
| 0.822 | 1.567991952 | 7.839959758 |
| 0.717 | 1.366080511 | 6.830402556 |
| 0.852 | 1.64544453 | 8.227222648 |
| 0.756 | 1.428515326 | 7.142576632 |
| 0.785 | 1.484564378 | 7.422821888 |
| 0.71 | 1.35644559 | 6.78222795 |
| 0.811 | 1.541793508 | 7.70896754 |
| 0.842 | 1.618651024 | 8.093255118 |
| 0.734 | 1.391470604 | 6.957353022 |
| 0.858 | 1.661989104 | 8.309945518 |
| 1.029 | 2.281209396 | 11.40604698 |
| 1.021 | 2.245876636 | 11.22938318 |
| 1.013 | 2.211168503 | 11.05584252 |
| 0.997 | 2.143626119 | 10.7181306 |
| 1.004 | 2.172868478 | 10.86434239 |
| 1.083 | 2.536043431 | 12.68021716 |
| 0.965 | 2.016036878 | 10.08018439 |
| 0.876 | 1.713730942 | 8.568654712 |
| 0.933 | 1.898441671 | 9.492208356 |
| 1.071 | 2.476954176 | 12.38477088 |
| 0.958 | 1.989458944 | 9.947294718 |
| 0.972 | 2.043093042 | 10.21546521 |
| 1.035 | 2.308118878 | 11.54059439 |
| 1.03 | 2.28566991 | 11.42834955 |
| 1.044 | 2.349141886 | 11.74570943 |
| 0.969 | 2.031438984 | 10.15719492 |
| 1.017 | 2.228444491 | 11.14222246 |
| 1.022 | 2.250259072 | 11.25129536 |

| | | |
|---|---|---|
| 0.989 | 2.110791868 | 10.55395934 |
| 0.9 | 1.787639 | 8.938195 |
| 0.869 | 1.693233364 | 8.46616682 |
| 1.046 | 2.358365468 | 11.79182734 |
| 0.907 | 1.810254455 | 9.051272276 |
| 0.926 | 1.874049932 | 9.370249662 |
| 0.944 | 1.937737766 | 9.688688832 |
| 1.03 | 2.28566991 | 11.42834955 |
| 0.968 | 2.027573818 | 10.13786909 |
| 1.023 | 2.254651267 | 11.27325634 |
| 0.984 | 2.090587654 | 10.45293827 |
| 0.935 | 1.905498578 | 9.527492888 |
| 0.889 | 1.753066648 | 8.76533324 |
| 0.913 | 1.830019763 | 9.150098816 |
| 0.998 | 2.14777432 | 10.7388716 |
| 0.969 | 2.031438984 | 10.15719492 |

| | |
|---|---|
| 2.01 | 2.588 |
| 1.942 | 2.52 |
| 10 | 20 |

| | | | | |
|---|---|---|---|---|
| 8.16512029 | 8.12650468 | 8.145812485 | 8.01010417 | 7.62868319 |
| 8.52262134 | 8.49526909 | 8.508945215 | 8.17975063 | 7.79164304 |
| 8.32060714 | 8.28705341 | 8.303830275 | 8.22988345 | 7.83995976 |
| 7.37100945 | 7.30038617 | 7.33569781 | 7.15875566 | 6.83040256 |
| 8.77777064 | 8.75774174 | 8.76775619 | 8.62943041 | 8.22722265 |
| 7.80525115 | 7.75369279 | 7.77947197 | 7.49717204 | 7.14257663 |
| 8.0846087 | 8.04325885 | 8.063933775 | 7.7943465 | 7.42282189 |
| 7.24975637 | 7.17271513 | 7.21123575 | 7.10544892 | 6.78222795 |
| 8.3700094 | 8.33800899 | 8.354009195 | 8.09379604 | 7.70896754 |
| 8.72267839 | 8.70111142 | 8.711894905 | 8.49163029 | 8.09325512 |
| 7.56589109 | 7.50443844 | 7.535164765 | 7.29763521 | 6.95735302 |
| 8.93306001 | 8.91725065 | 8.92515533 | 8.71432703 | 8.30994552 |
| 11.58349816 | 11.62337822 | 11.60343819 | 11.83271534 | 11.40604698 |
| 11.71156467 | 11.75363006 | 11.73259737 | 11.65671778 | 11.22938318 |
| 11.7762174 | 11.81937379 | 11.7977956 | 11.4836756 | 11.05584252 |
| 10.86936746 | 10.89641149 | 10.88288948 | 11.14645742 | 10.7181306 |
| 11.353 | 11.38886211 | 11.37093106 | 11.29253577 | 10.86434239 |
| 12.03895814 | 12.08646749 | 12.06271282 | 13.0980009 | 12.68021716 |
| 10.84978969 | 10.87646476 | 10.86312723 | 10.50748578 | 10.08018439 |
| 9.0790503 | 9.06706397 | 9.073057135 | 8.97899132 | 8.56865471 |
| 10.21144832 | 10.22550566 | 10.21847699 | 9.9158004 | 9.49220836 |
| 13.37721346 | 13.44515745 | 13.41118546 | 12.80518948 | 12.38477088 |
| 10.67565469 | 10.69900361 | 10.68732915 | 10.37401403 | 9.94729472 |
| 10.65653579 | 10.67951452 | 10.66802516 | 10.64322025 | 10.21546521 |
| 11.25006359 | 11.28409444 | 11.26707902 | 11.966653 | 11.54059439 |
| 12.81927389 | 12.87902041 | 12.84914715 | 11.85492284 | 11.42834955 |
| 11.9506437 | 11.99670403 | 11.97367387 | 12.17067649 | 11.74570943 |
| 11.06766893 | 11.09839577 | 11.08303235 | 10.58477126 | 10.15719492 |
| 11.33232094 | 11.36781705 | 11.350069 | 11.56982726 | 11.14222246 |
| 11.00769667 | 11.03731996 | 11.02250832 | 11.67855585 | 11.25129536 |
| 11.52008438 | 11.55887011 | 11.53947725 | 10.98228142 | 10.55395934 |
| 9.60974077 | 9.61066073 | 9.61020075 | 9.35515076 | 8.938195 |
| 9.00548157 | 8.99158526 | 8.998533415 | 8.87428846 | 8.46616682 |
| 12.74828875 | 12.80696124 | 12.777625 | 12.216523 | 11.79182734 |
| 9.8436784 | 9.84987139 | 9.846774895 | 9.46987424 | 9.05127228 |
| 10.12204858 | 10.13423837 | 10.12814348 | 9.79267252 | 9.37024966 |
| 10.39368934 | 10.41147254 | 10.40258094 | 10.11385869 | 9.68868883 |
| 11.47803797 | 11.51609353 | 11.49706575 | 11.85492284 | 11.42834955 |
| 10.90866065 | 10.93644223 | 10.92255144 | 10.56538063 | 10.13786909 |
| 11.2705591 | 11.30495645 | 11.28775778 | 11.7004401 | 11.27325634 |
| 11.04763229 | 11.0779914 | 11.06281185 | 10.88117221 | 10.45293827 |
| 10.05135474 | 10.06204829 | 10.05670152 | 9.9513954 | 9.52749289 |
| 9.56078139 | 9.56056828 | 9.560674835 | 9.17944262 | 8.76533324 |
| 9.94669061 | 9.95513609 | 9.95091335 | 9.5700096 | 9.15009882 |
| 11.52008438 | 11.55887011 | 11.53947725 | 11.16718723 | 10.7388716 |
| 10.48653062 | 10.50617213 | 10.49635138 | 10.58477126 | 10.15719492 |

Report
TSH

| Group | Mean | Standard Deviation |
|---|---|---|
| 1 | 8.031943713 | 0.561873758 |
| 2 | 11.12525836 | 0.857996217 |
| 3 | 10.38619774 | 0.933077434 |
| Total | 10.06124215 | 1.494667681 |

Tests of Normality

| | Group | Kolmogorov-Smirnov(K)a | df | Sig. | Shapiro-Wilk Statistic |
|---|---|---|---|---|---|
| | | Statistic | | | |
| TSH | 1 | 0.14 | 12 | .200* | 0.947 |
| | 2 | 0.196 | 18 | 0.065 | 0.912 |
| | 3 | 0.101 | 16 | .200* | 0.97 |

* The mean difference is significant at the 0.05 level.
a Lilliefors Significance Correction

Test of Homogeneity of Variances
TSH

| Levene's Stat | df1 | df2 | Sig. |
|---|---|---|---|
| 1.011 | 2 | 43 | 0.373 |

ANOVA
TSH

| | Sum of Square | df | Mean Square | F | Sig. |
|---|---|---|---|---|---|
| Between Group | 71.485 | 2 | 35.742 | 52.912 | 0 |
| Within Groups | 29.047 | 43 | 0.676 | | |
| Total | 100.531 | 45 | | | |

Multiple Comparisons
Dependent Variable

| | (I) Group | (J) Group | Mean Differen | Std. Error | Sig. |
|---|---|---|---|---|---|
| LSD(L) | 1 | 2 | -3.0933146513 | 0.306301726 | 0 |
| | | 3 | -2.3542540230 | 0.313865871 | 0 |
| | 2 | 1 | 3.0933146513 | 0.306301726 | 0 |
| | | 3 | .73906062828* | 0.282396238 | 0.012 |
| | 3 | 1 | 2.35425402308 | 0.313865871 | 0 |
| | | 2 | -.73906062828 | 0.282396238 | 0.012 |
| Tamhane | 1 | 2 | -3.0933146513 | 0.259241489 | 0 |
| | | 3 | -2.3542540230 | 0.284118116 | 0 |
| | 2 | 1 | 3.0933146513 | 0.259241489 | 0 |

| | | | | |
|---|---|---|---|---|
| | 3 | 0.739060628 | 0.308726794 | 0.067 |
| 3 | 1 | 2.35425402308 | 0.284118116 | 0 |
| | 2 | -0.739060628 | 0.308726794 | 0.067 |

* The mean difference is significant at the 0.05 level.

| | | | | |
|---|---|---|---|---|
| 7.81939368 | 7.55104798 | 9.02499578 | 8.28802188 | 8.28802188 |
| 7.985696835 | 7.87768336 | 9.36486441 | 8.621273885 | 8.62127389 |
| 8.034921605 | 7.62050239 | 9.09772205 | 8.35911222 | 8.35911222 |
| 6.99457911 | 6.6897984 | 8.08672629 | 7.388262345 | 7.38826235 |
| 8.42832653 | 8.08834256 | 9.58155747 | 8.834950015 | 8.83495002 |
| 7.319874335 | 7.07800771 | 8.52058518 | 7.799296445 | 7.79929645 |
| 7.608584195 | 7.34291322 | 8.80525187 | 8.074082545 | 8.07408255 |
| 6.943838435 | 6.56564 | 7.94030877 | 7.252974385 | 7.25297439 |
| 7.90138179 | 7.70379398 | 9.18459165 | 8.444192815 | 8.44419282 |
| 8.292442705 | 8.02114476 | 9.51261862 | 8.76688169 | 8.76688169 |
| 7.127494115 | 6.98908943 | 8.42337558 | 7.706232505 | 7.70623251 |
| 8.512136275 | 8.2546869 | 9.75154419 | 9.003115545 | 9.00311555 |
| 11.61938116 | 11.96089298 | 10.7835317 | 11.37221234 | 11.37221234 |
| 11.44305048 | 12.03267302 | 11.01246333 | 11.52256818 | 11.52256818 |
| 11.26975906 | 10.91627971 | 10.51208118 | 10.71418045 | 10.71418045 |
| 10.93229401 | 11.20083542 | 11.45484035 | 11.32783789 | 11.32783789 |
| 11.07843908 | 11.04656938 | 12.52244834 | 11.78450886 | 11.78450886 |
| 12.88910903 | 10.27162054 | 10.01852469 | 10.14507262 | 10.14507262 |
| 10.29383509 | 10.33191943 | 11.82321169 | 11.07756556 | 11.07756556 |
| 8.773823015 | 8.28310678 | 9.78049755 | 9.031802165 | 9.03180217 |
| 9.70400438 | 9.33249424 | 10.83569539 | 10.08409482 | 10.08409482 |
| 12.59498018 | 12.82616 | 14.24655517 | 13.53635759 | 13.53635759 |
| 10.16065438 | 9.8053329 | 11.3045368 | 10.55493485 | 10.55493485 |
| 10.42934273 | 9.71179303 | 11.21204158 | 10.46191731 | 10.46191731 |
| 11.7536237 | 10.5568424 | 10.23613209 | 10.39648725 | 10.39648725 |
| 11.6416362 | 9.5829247 | 11.08441499 | 10.33366985 | 10.33366985 |
| 11.95819296 | 11.2900809 | 10.64638417 | 10.96823254 | 10.96823254 |
| 10.37098309 | 10.5568424 | 12.04381289 | 11.30032765 | 11.30032765 |
| 11.35602486 | 10.6610682 | 12.14586401 | 11.40346611 | 11.40346611 |
| 11.46492561 | 10.35211846 | 11.84304411 | 11.09758129 | 11.09758129 |
| 10.76812038 | 11.04656938 | 12.52244834 | 11.78450886 | 11.78450886 |
| 9.14667288 | 8.79666244 | 10.29990569 | 9.548284065 | 9.54828407 |
| 8.67022764 | 8.36955928 | 9.86842435 | 9.118991815 | 9.11899182 |
| 12.00417517 | 12.05669911 | 13.50361218 | 12.78015565 | 12.78015565 |
| 9.26057326 | 8.97511404 | 10.47894989 | 9.727031965 | 9.72703197 |
| 9.58146109 | 9.24541564 | 10.74897814 | 9.99719689 | 9.99719689 |
| 9.90127376 | 9.7304016 | 11.23045173 | 10.48042667 | 10.48042667 |
| 11.6416362 | 9.65626554 | 10.20451197 | 9.930388755 | 9.93038876 |
| 10.35162486 | 10.35211846 | 11.84304411 | 11.09758129 | 11.09758129 |
| 11.48684822 | 9.8053329 | 10.46245091 | 10.13389191 | 10.13389191 |
| 10.66705524 | 10.1130104 | 9.89808885 | 10.00554963 | 10.00554963 |
| 9.739444145 | 9.78652552 | 10.5788771 | 10.18270131 | 10.18270131 |
| 8.97238793 | 8.71753615 | 10.22029981 | 9.46891798 | 9.46891798 |
| 9.36005421 | 9.04149659 | 10.54539025 | 9.79344342 | 9.79344342 |
| 10.95302942 | 10.91627971 | 12.39532018 | 11.65579995 | 11.65579995 |
| 10.37098309 | 9.52844095 | 11.03038458 | 10.27941277 | 10.27941277 |

| df | Sig. |
|---|---|
| 12 | 0.596 |
| 18 | 0.095 |
| 16 | 0.843 |

| 95% Confidence Interval | |
|---|---|
| Lower Bound | Upper Bound |
| -3.711030954 | -2.475598349 |
| -2.987224877 | -1.72128317 |
| 2.475598349 | 3.711030954 |
| 0.169554337 | 1.308566919 |
| 1.72128317 | 2.987224877 |
| -1.308566919 | -0.169554337 |
| -3.751559068 | -2.435070235 |
| -3.081099522 | -1.627408524 |
| 2.435070235 | 3.751559068 |

| | |
|---|---|
| -0.040484167 | 1.518605423 |
| 1.627408524 | 3.081099522 |
| -1.518605423 | 0.040484167 |

| | | |
|---|---|---|
| 7.81939368 | 8.14581249 | 8.08440935 |
| 7.98569684 | 8.50894522 | 8.371971983 |
| 8.03492161 | 8.30383028 | 8.23262137 |
| 6.99457911 | 7.33569781 | 7.23951309 |
| 8.42832653 | 8.76775619 | 8.677010913 |
| 7.31987434 | 7.77947197 | 7.63288092 |
| 7.6085842 | 8.06393378 | 7.91553351 |
| 6.94383844 | 7.21123575 | 7.136016193 |
| 7.90138179 | 8.3540092 | 8.233194603 |
| 8.29244271 | 8.71189491 | 8.590406437 |
| 7.12749412 | 7.53516477 | 7.456297133 |
| 8.51213628 | 8.92515533 | 8.813469053 |
| 11.61938116 | 11.60343819 | 11.53167723 |
| 11.44305048 | 11.73259737 | 11.56607201 |
| 11.26975906 | 11.7977956 | 11.26057837 |
| 10.93229401 | 10.88288948 | 11.04767379 |
| 11.07843908 | 11.37093106 | 11.411293 |
| 12.88910903 | 12.06271282 | 11.69896482 |
| 10.29383509 | 10.86312723 | 10.74484263 |
| 8.77382302 | 9.07305714 | 8.959560777 |
| 9.70400438 | 10.21847699 | 10.00219206 |
| 12.59498018 | 13.41118546 | 13.18084108 |
| 10.16065438 | 10.68732915 | 10.46763946 |
| 10.42934273 | 10.66802516 | 10.51976173 |
| 11.7536237 | 11.26707902 | 11.13906332 |
| 11.6416362 | 12.84914715 | 11.60815107 |
| 11.95819296 | 11.97367387 | 11.63336646 |
| 10.37098309 | 11.08303235 | 10.91811436 |
| 11.35602486 | 11.350069 | 11.36985332 |
| 11.46492561 | 11.02250832 | 11.19500507 |
| 10.76812038 | 11.53947725 | 11.3640355 |
| 9.14667288 | 9.61020075 | 9.435052567 |
| 8.67022764 | 8.99853342 | 8.92925096 |
| 12.00417517 | 12.777625 | 12.52065194 |
| 9.26057326 | 9.8467749 | 9.611460043 |
| 9.58146109 | 10.12814348 | 9.902267153 |
| 9.90127376 | 10.40258094 | 10.26142712 |
| 11.6416362 | 11.49706575 | 11.02303024 |
| 10.35162486 | 10.92255144 | 10.79058586 |
| 11.48684822 | 11.28775778 | 10.9694993 |
| 10.66705524 | 11.06281185 | 10.57847224 |
| 9.73944415 | 10.05670152 | 9.992948993 |
| 8.97238793 | 9.56067484 | 9.333993583 |
| 9.36005421 | 9.95091335 | 9.701470327 |
| 10.95302942 | 11.53947725 | 11.38276887 |
| 10.37098309 | 10.49635138 | 10.38224908 |

| | | | | | |
|---|---|---|---|---|---|
| 0.036 | 0.302 | 0.503 | 0.988 | 1.532 | 2.433 |
| 0 | 0.266 | 0.467 | 0.952 | 1.496 | 2.397 |
| | 1 | 2 | 4 | 8 | 16 |

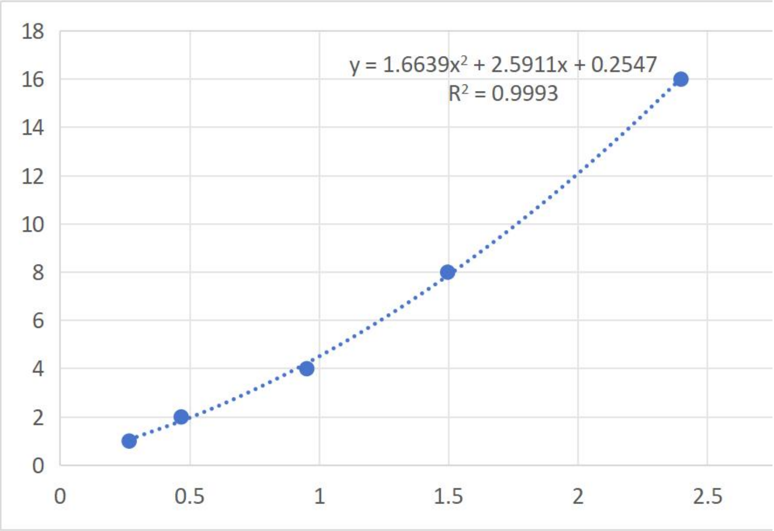


| Sample ID | OD (450nm) | Blank Corrected | Dilution Factor | Concentration |
|---|---|---|---|---|
| 1 | 1.038 | 1.002 | 4.521544456 | 22.60772228 |
| 2 | 1.052 | 1.016 | 4.604828358 | 23.02414179 |
| 3 | 1.047 | 1.011 | 4.575009232 | 22.87504616 |
| 4 | 1.345 | 1.309 | 6.497510936 | 32.48755468 |
| 5 | 1.298 | 1.262 | 6.174668552 | 30.87334276 |
| 6 | 1.376 | 1.34 | 6.71447284 | 33.5723642 |
| 7 | 1.093 | 1.057 | 4.852483311 | 24.26241656 |
| 8 | 1.089 | 1.053 | 4.828075595 | 24.14037798 |
| 9 | 1.099 | 1.063 | 4.889194719 | 24.4459736 |
| 10 | 1.142 | 1.106 | 5.15579898 | 25.7789949 |
| 11 | 1.167 | 1.131 | 5.313630088 | 26.56815044 |
| 12 | 1.149 | 1.113 | 5.199782039 | 25.9989102 |
| 13 | 1.058 | 1.022 | 4.640721128 | 23.20360564 |
| 14 | 1.073 | 1.037 | 4.730977179 | 23.6548859 |
| 15 | 1.084 | 1.048 | 4.797640826 | 23.98820413 |
| 16 | 1.231 | 1.195 | 5.727155298 | 28.63577649 |
| 17 | 1.228 | 1.192 | 5.70746681 | 28.53733405 |
| 18 | 1.225 | 1.189 | 5.687808272 | 28.43904136 |
| 19 | 1.136 | 1.1 | 5.118229 | 25.591145 |
| 20 | 1.152 | 1.116 | 5.218681838 | 26.09340919 |
| 21 | 1.164 | 1.128 | 5.294580538 | 26.47290269 |
| 22 | 1.097 | 1.061 | 4.876944272 | 24.38472136 |
| 23 | 1.078 | 1.042 | 4.76122892 | 23.8061446 |
| 24 | 1.083 | 1.047 | 4.791563855 | 23.95781928 |
| 1 | 0.742 | 0.706 | 2.91336426 | 14.5668213 |
| 2 | 0.735 | 0.699 | 2.878862104 | 14.39431052 |
| 3 | 0.723 | 0.687 | 2.820094919 | 14.1004746 |
| 4 | 0.737 | 0.701 | 2.888703224 | 14.44351612 |
| 5 | 0.731 | 0.695 | 2.859219798 | 14.29609899 |
| 6 | 0.754 | 0.718 | 2.972890184 | 14.86445092 |
| 7 | 0.759 | 0.723 | 2.997834083 | 14.98917042 |
| 8 | 0.742 | 0.706 | 2.91336426 | 14.5668213 |

| | | | | |
|---|---|---|---|---|
| 9 | 0.755 | 0.719 | 2.977872308 | 14.88936154 |
| 10 | 0.705 | 0.669 | 2.732842648 | 13.66421324 |
| 11 | 0.710 | 0.674 | 2.756971236 | 13.78485618 |
| 12 | 0.713 | 0.677 | 2.771488323 | 13.85744162 |
| 13 | 0.723 | 0.687 | 2.820094919 | 14.1004746 |
| 14 | 0.728 | 0.692 | 2.84452301 | 14.22261505 |
| 15 | 0.719 | 0.683 | 2.800612347 | 14.00306174 |
| 16 | 0.757 | 0.721 | 2.98784654 | 14.9392327 |
| 17 | 0.765 | 0.729 | 3.02787658 | 15.1393829 |
| 18 | 0.744 | 0.708 | 2.92325197 | 14.61625985 |
| 19 | 0.762 | 0.726 | 3.012840356 | 15.06420178 |
| 20 | 0.763 | 0.727 | 3.017849103 | 15.08924552 |
| 21 | 0.756 | 0.72 | 2.98285776 | 14.9142888 |
| 22 | 0.761 | 0.725 | 3.007834938 | 15.03917469 |
| 23 | 0.737 | 0.701 | 2.888703224 | 14.44351612 |
| 24 | 0.725 | 0.689 | 2.829856172 | 14.14928086 |
| 25 | 0.695 | 0.659 | 2.684835056 | 13.42417528 |
| 26 | 0.683 | 0.647 | 2.627665215 | 13.13832608 |
| 27 | 0.689 | 0.653 | 2.656190235 | 13.28095118 |
| 28 | 0.757 | 0.721 | 2.98784654 | 14.9392327 |
| 29 | 0.741 | 0.705 | 2.908425398 | 14.54212699 |
| 30 | 0.777 | 0.741 | 3.088320976 | 15.44160488 |
| 1 | 0.742 | 0.706 | 2.91336426 | 14.5668213 |
| 2 | 0.739 | 0.703 | 2.898557655 | 14.49278828 |
| 3 | 0.735 | 0.699 | 2.878862104 | 14.39431052 |
| 4 | 0.801 | 0.765 | 3.210647378 | 16.05323689 |
| 5 | 0.796 | 0.76 | 3.18500464 | 15.9250232 |
| 6 | 0.792 | 0.756 | 3.16455035 | 15.82275175 |
| 7 | 0.748 | 0.712 | 2.943067322 | 14.71533661 |
| 8 | 0.767 | 0.731 | 3.037917368 | 15.18958684 |
| 9 | 0.773 | 0.737 | 3.068119599 | 15.340598 |
| 10 | 0.744 | 0.708 | 2.92325197 | 14.61625985 |
| 11 | 0.739 | 0.703 | 2.898557655 | 14.49278828 |
| 12 | 0.756 | 0.72 | 2.98285776 | 14.9142888 |
| 13 | 0.771 | 0.735 | 3.058038878 | 15.29019439 |
| 14 | 0.777 | 0.741 | 3.088320976 | 15.44160488 |
| 15 | 0.763 | 0.727 | 3.017849103 | 15.08924552 |
| 16 | 0.738 | 0.702 | 2.893628776 | 14.46814388 |
| 17 | 0.745 | 0.709 | 2.928200816 | 14.64100408 |
| 18 | 0.733 | 0.697 | 2.869034295 | 14.34517148 |
| 19 | 0.755 | 0.719 | 2.977872308 | 14.88936154 |
| 20 | 0.745 | 0.709 | 2.928200816 | 14.64100408 |
| 21 | 0.76 | 0.724 | 3.002832846 | 15.01416423 |
| 22 | 0.793 | 0.757 | 3.169658931 | 15.84829466 |
| 23 | 0.801 | 0.765 | 3.210647378 | 16.05323689 |
| 24 | 0.807 | 0.771 | 3.24152848 | 16.2076424 |
| 25 | 0.749 | 0.713 | 2.948029479 | 14.7401474 |
| 26 | 0.737 | 0.701 | 2.888703224 | 14.44351612 |
| 27 | 0.74 | 0.704 | 2.903489862 | 14.51744931 |
| 28 | 0.763 | 0.727 | 3.017849103 | 15.08924552 |
| 29 | 0.754 | 0.718 | 2.972890184 | 14.86445092 |
| 30 | 0.75 | 0.714 | 2.952994964 | 14.76497482 |

| | | | | |
|---|---|---|---|---|
| 0.042 | 0.342 | 0.513 | 0.994 | 1.548 |
| 0 | 0.3 | 0.471 | 0.952 | 1.506 |
| | 1 | 2 | 4 | 8 |

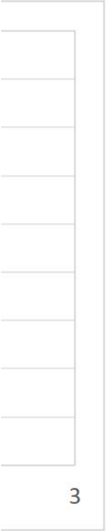


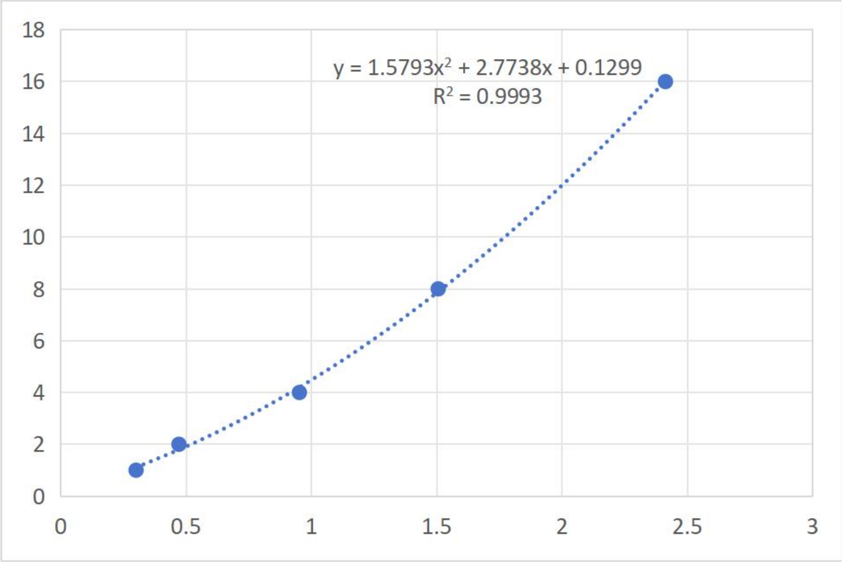


| Blank Corrected | Dilution Factor | Concentration | |
|---|---|---|---|
| 0.996 | 4.459295669 | 22.29647834 | 22.60772 |
| 1.01 | 4.54248193 | 22.71240965 | 23.02414 |
| 1.005 | 4.512701483 | 22.56350741 | 22.87505 |
| 1.303 | 6.425511154 | 32.12755577 | 32.48755 |
| 1.256 | 6.105195405 | 30.52597702 | 30.87334 |
| 1.334 | 6.640601991 | 33.20300995 | 33.57236 |
| 1.051 | 4.789660159 | 23.9483008 | 24.26242 |
| 1.047 | 4.765311474 | 23.82655737 | 24.14038 |
| 1.057 | 4.826277946 | 24.13138973 | 24.44597 |
| 1.1 | 5.092033 | 25.460165 | 25.77899 |
| 1.125 | 5.249226563 | 26.24613281 | 26.56815 |
| 1.107 | 5.135848206 | 25.67924103 | 25.99891 |
| 1.016 | 4.578322701 | 22.8916135 | 23.20361 |
| 1.031 | 4.668422107 | 23.34211054 | 23.65489 |
| 1.042 | 4.734946685 | 23.67473343 | 23.9882 |
| 1.189 | 5.660637775 | 28.30318888 | 28.63578 |
| 1.186 | 5.641063863 | 28.20531931 | 28.53733 |
| 1.183 | 5.621518378 | 28.10759189 | 28.43904 |
| 1.094 | 5.054600295 | 25.27300147 | 25.59115 |
| 1.11 | 5.15467353 | 25.77336765 | 26.09341 |
| 1.122 | 5.230259101 | 26.15129551 | 26.4729 |
| 1.055 | 4.814059383 | 24.07029691 | 24.38472 |
| 1.036 | 4.698613173 | 23.49306586 | 23.80614 |
| 1.041 | 4.728883203 | 23.64441602 | 23.95782 |
| 0.7 | 2.845417 | 14.227085 | 14.56682 |
| 0.693 | 2.810600646 | 14.05300323 | 14.39431 |
| 0.681 | 2.751275547 | 13.75637774 | 14.10047 |
| 0.695 | 2.820532383 | 14.10266191 | 14.44352 |
| 0.689 | 2.790775075 | 13.95387538 | 14.2961 |
| 0.712 | 2.905462259 | 14.5273113 | 14.86445 |
| 0.717 | 2.930615358 | 14.65307679 | 14.98917 |
| 0.7 | 2.845417 | 14.227085 | 14.56682 |

| | | | |
|---|---|---|---|
| 0.713 | 2.910486562 | 14.55243281 | 14.88936 |
| 0.663 | 2.663140722 | 13.31570361 | 13.66421 |
| 0.668 | 2.687519963 | 13.43759982 | 13.78486 |
| 0.671 | 2.702185411 | 13.51092706 | 13.85744 |
| 0.681 | 2.751275547 | 13.75637774 | 14.10047 |
| 0.686 | 2.775939063 | 13.87969531 | 14.22262 |
| 0.677 | 2.73160159 | 13.65800795 | 14.00306 |
| 0.715 | 2.920544643 | 14.60272321 | 14.93923 |
| 0.723 | 2.96090331 | 14.80451655 | 15.13938 |
| 0.702 | 2.855392957 | 14.27696479 | 14.61626 |
| 0.72 | 2.94574512 | 14.7287256 | 15.0642 |
| 0.721 | 2.950794691 | 14.75397346 | 15.08925 |
| 0.714 | 2.915514023 | 14.57757011 | 14.91429 |
| 0.719 | 2.940698707 | 14.70349354 | 15.03917 |
| 0.695 | 2.820532383 | 14.10266191 | 14.44352 |
| 0.683 | 2.761131478 | 13.80565739 | 14.14928 |
| 0.653 | 2.614619134 | 13.07309567 | 13.42418 |
| 0.641 | 2.556810163 | 12.78405082 | 13.13833 |
| 0.647 | 2.585657794 | 12.92828897 | 13.28095 |
| 0.715 | 2.920544643 | 14.60272321 | 14.93923 |
| 0.699 | 2.840433759 | 14.2021688 | 14.54213 |
| 0.735 | 3.021820343 | 15.10910171 | 15.4416 |
| 0.7 | 2.845417 | 14.227085 | 14.56682 |
| 0.697 | 2.830476754 | 14.15238377 | 14.49279 |
| 0.693 | 2.810600646 | 14.05300323 | 14.39431 |
| 0.759 | 3.145018923 | 15.72509462 | 16.05324 |
| 0.754 | 3.119202519 | 15.59601259 | 15.92502 |
| 0.75 | 3.09860625 | 15.49303125 | 15.82275 |
| 0.706 | 2.875382775 | 14.37691387 | 14.71534 |
| 0.725 | 2.971024563 | 14.85512281 | 15.18959 |
| 0.731 | 3.001464127 | 15.00732064 | 15.3406 |
| 0.702 | 2.855392957 | 14.27696479 | 14.61626 |
| 0.697 | 2.830476754 | 14.15238377 | 14.49279 |
| 0.714 | 2.915514023 | 14.57757011 | 14.91429 |
| 0.729 | 2.991304971 | 14.95652486 | 15.29019 |
| 0.735 | 3.021820343 | 15.10910171 | 15.4416 |
| 0.721 | 2.950794691 | 14.75397346 | 15.08925 |
| 0.696 | 2.825502989 | 14.12751494 | 14.46814 |
| 0.703 | 2.860385674 | 14.30192837 | 14.641 |
| 0.691 | 2.800681543 | 14.00340772 | 14.34517 |
| 0.713 | 2.910486562 | 14.55243281 | 14.88936 |
| 0.703 | 2.860385674 | 14.30192837 | 14.641 |
| 0.718 | 2.935655453 | 14.67827727 | 15.01416 |
| 0.751 | 3.103750579 | 15.5187529 | 15.84829 |
| 0.759 | 3.145018923 | 15.72509462 | 16.05324 |
| 0.765 | 3.176102843 | 15.88051421 | 16.20764 |
| 0.707 | 2.880388126 | 14.40194063 | 14.74015 |
| 0.695 | 2.820532383 | 14.10266191 | 14.44352 |
| 0.698 | 2.835453677 | 14.17726839 | 14.51745 |
| 0.721 | 2.950794691 | 14.75397346 | 15.08925 |
| 0.712 | 2.905462259 | 14.5273113 | 14.86445 |
| 0.708 | 2.885396635 | 14.42698318 | 14.76497 |

2.455
2.413
16

| | | | | |
|---|---|---|---|---|
| 22.29648 | 22.45210031 | 1 | 22.4521 | 22.45210031 |
| 22.71241 | 22.86827572 | | 22.86828 | 32.30755523 |
| 22.56351 | 22.71927679 | | 22.71928 | 24.10535868 |
| 32.12756 | 32.30755523 | 2 | 32.30756 | 25.61957995 |
| 30.52598 | 30.69965989 | | 30.69966 | 23.04760957 |
| 33.20301 | 33.38768708 | | 33.38769 | 28.46948269 |
| 23.9483 | 24.10535868 | 3 | 24.10536 | 25.43207324 |
| 23.82656 | 23.98346768 | | 23.98347 | 24.22750914 |
| 24.13139 | 24.28868167 | | 24.28868 | 14.39695315 |
| 25.46017 | 25.61957995 | 4 | 25.61958 | 14.27308902 |
| 26.24613 | 26.40714163 | | 26.40714 | 14.82112361 |
| 25.67924 | 25.83907562 | | 25.83908 | 13.48995843 |
| 22.89161 | 23.04760957 | 5 | 23.04761 | 13.92842617 |
| 23.34211 | 23.49849822 | | 23.4985 | 14.77097796 |
| 23.67473 | 23.83146878 | | 23.83147 | 14.89646369 |
| 28.30319 | 28.46948269 | 6 | 28.46948 | 14.87133412 |
| 28.20532 | 28.37132668 | | 28.37133 | 13.24863548 |
| 28.10759 | 28.27331663 | | 28.27332 | 14.77097796 |
| 25.273 | 25.43207324 | 7 | 25.43207 | 14.39695315 |
| 25.77337 | 25.93338842 | | 25.93339 | 15.88916576 |
| 26.1513 | 26.3120991 | | 26.3121 | 14.54612524 |
| 24.0703 | 24.22750914 | 8 | 24.22751 | 14.44661232 |
| 23.49307 | 23.64960523 | | 23.64961 | 15.12335963 |
| 23.64442 | 23.80111765 | | 23.80112 | 14.29782941 |
| 14.22709 | 14.39695315 | 9 | 14.39695 | 14.72089718 |
| 14.053 | 14.22365688 | | 14.22366 | 15.68352378 |
| 13.75638 | 13.92842617 | | 13.92843 | 14.57104402 |
| 14.10266 | 14.27308902 | 10 | 14.27309 | 14.92160949 |
| 13.95388 | 14.12498719 | | 14.12499 | |
| 14.52731 | 14.69588111 | | 14.69588 | |
| 14.65308 | 14.82112361 | 11 | 14.82112 | |
| 14.22709 | 14.39695315 | | 14.39695 | |

| | | | |
|---|---|---|---|
| 14.55243 | 14.72089718 | | 14.7209 |
| 13.3157 | 13.48995843 | 12 | 13.48996 |
| 13.4376 | 13.611228 | | 13.61123 |
| 13.51093 | 13.68418434 | | 13.68418 |
| 13.75638 | 13.92842617 | 13 | 13.92843 |
| 13.8797 | 14.05115518 | | 14.05116 |
| 13.65801 | 13.83053485 | | 13.83053 |
| 14.60272 | 14.77097796 | 14 | 14.77098 |
| 14.80452 | 14.97194973 | | 14.97195 |
| 14.27696 | 14.44661232 | | 14.44661 |
| 14.72873 | 14.89646369 | 15 | 14.89646 |
| 14.75397 | 14.92160949 | | 14.92161 |
| 14.57757 | 14.74592946 | | 14.74593 |
| 14.70349 | 14.87133412 | 16 | 14.87133 |
| 14.10266 | 14.27308902 | | 14.27309 |
| 13.80566 | 13.97746913 | | 13.97747 |
| 13.0731 | 13.24863548 | 17 | 13.24864 |
| 12.78405 | 12.96118845 | | 12.96119 |
| 12.92829 | 13.10462008 | | 13.10462 |
| 14.60272 | 14.77097796 | 18 | 14.77098 |
| 14.20217 | 14.3721479 | | 14.37215 |
| 15.1091 | 15.2753533 | | 15.27535 |
| 14.22709 | 14.39695315 | 19 | 14.39695 |
| 14.15238 | 14.32258603 | | 14.32259 |
| 14.053 | 14.22365688 | | 14.22366 |
| 15.72509 | 15.88916576 | 20 | 15.88917 |
| 15.59601 | 15.7605179 | | 15.76052 |
| 15.49303 | 15.6578915 | | 15.65789 |
| 14.37691 | 14.54612524 | 21 | 14.54613 |
| 14.85512 | 15.02235483 | | 15.02235 |
| 15.00732 | 15.17395932 | | 15.17396 |
| 14.27696 | 14.44661232 | 22 | 14.44661 |
| 14.15238 | 14.32258603 | | 14.32259 |
| 14.57757 | 14.74592946 | | 14.74593 |
| 14.95652 | 15.12335963 | 23 | 15.12336 |
| 15.1091 | 15.2753533 | | 15.27535 |
| 14.75397 | 14.92160949 | | 14.92161 |
| 14.12751 | 14.29782941 | 24 | 14.29783 |
| 14.30193 | 14.47146623 | | 14.47147 |
| 14.00341 | 14.1742896 | | 14.17429 |
| 14.55243 | 14.72089718 | 25 | 14.7209 |
| 14.30193 | 14.47146623 | | 14.47147 |
| 14.67828 | 14.84622075 | | 14.84622 |
| 15.51875 | 15.68352378 | 26 | 15.68352 |
| 15.72509 | 15.88916576 | | 15.88917 |
| 15.88051 | 16.04407831 | | 16.04408 |
| 14.40194 | 14.57104402 | 27 | 14.57104 |
| 14.10266 | 14.27308902 | | 14.27309 |
| 14.17727 | 14.34735885 | | 14.34736 |
| 14.75397 | 14.92160949 | 28 | 14.92161 |
| 14.52731 | 14.69588111 | | 14.69588 |
| 14.42698 | 14.595979 | | 14.59598 |

| | | |
|---|---|---|
| 22.86827572 | 22.71927679 | 22.67988427 |
| 30.69965989 | 33.38768708 | 32.13163407 |
| 23.98346768 | 24.28868167 | 24.12583601 |
| 26.40714163 | 25.83907562 | 25.95526573 |
| 23.49849822 | 23.83146878 | 23.45919219 |
| 28.37132668 | 28.27331663 | 28.37137533 |
| 25.93338842 | 26.3120991 | 25.89252025 |
| 23.64960523 | 23.80111765 | 23.89274401 |
| 14.22365688 | 13.92842617 | 14.18301207 |
| 14.12498719 | 14.69588111 | 14.36465244 |
| 14.39695315 | 14.72089718 | 14.64632465 |
| 13.611228 | 13.68418434 | 13.59512359 |
| 14.05115518 | 13.83053485 | 13.9367054 |
| 14.97194973 | 14.44661232 | 14.72984667 |
| 14.92160949 | 14.74592946 | 14.85466755 |
| 14.27308902 | 13.97746913 | 14.37396409 |
| 12.96118845 | 13.10462008 | 13.10481467 |
| 14.3721479 | 15.2753533 | 14.80615972 |
| 14.32258603 | 14.22365688 | 14.31439869 |
| 15.7605179 | 15.6578915 | 15.76919172 |
| 15.02235483 | 15.17395932 | 14.91414646 |
| 14.32258603 | 14.74592946 | 14.5050426 |
| 15.2753533 | 14.92160949 | 15.10677414 |
| 14.47146623 | 14.1742896 | 14.31452841 |
| 14.47146623 | 14.84622075 | 14.67952805 |
| 15.88916576 | 16.04407831 | 15.87225595 |
| 14.27308902 | 14.34735885 | 14.39716396 |
| 14.69588111 | 14.595979 | 14.7378232 |

Report
FT3 (28)

| Group | Mean | Std. Deviation |
|---|---|---|
| 1 | 25.81355€ | 3.1256476391919 |
| 2 | 14.25952 | 7.5708493283189 |
| 3 | 14.86108 | 5.5674542100060 |
| Total | 17.77552C | 5.4421542989522 |

Tests of Normality

| | Group | Kolmogorov-Smirnova | | | Shapiro-Wilk | | |
|---|---|---|---|---|---|---|---|
| | | Statistic | df | Sig. | Statistic | df | Sig. |
| FT3 (28) | 1 | 0.232 | 8 | .200* | 0.875 | 8 | 0.167 |
| | 2 | 0.173 | 10 | .200* | 0.905 | 10 | 0.251 |
| | 3 | 0.186 | 10 | .200* | 0.858 | 10 | 0.073 |

* This is a lower bound of the true significance.
a Lilliefors Significance Correction

ANOVA
FT3 (28)

| | Sum of Sq | df | Mean Square |
|---|---|---|---|
| Between G | 725.442 | 2 | 362.721 |

Robust Tests of Equality of Means
FT3 (28)

| | Statistic | df1 | df2 | Sig. |
|---|---|---|---|---|
| Welch | 51.424 | 2 | 13.594 | 0 |

a Asymptotically F distributed.

Multiple Comparisons
Dependent Variable: FT3 (28)
Games-Howell

| (I) Group | (J) Group | Mean Diff | Std. Errc | Sig. | 95% Confidence Interval | |
|---|---|---|---|---|---|---|
| | | | | | Lower Bou | Upper Bound |
| 1 | 2 | 11.55402\ | 1.1197303 | 0 | 8.296752 | 14.811306494078 |
| | 3 | 10.95247 | 1.1195577 | 0 | 7.695245 | 14.209697822711 |
| 2 | 1 | -11.5540 | 1.1197303 | 0 | -14.8113 | -8.29675 |
| | 3 | -0.60156 | .25453354 | 0.072 | -1.25117 | 0.048055 |
| 3 | 1 | -10.9524 | 1.1195577 | 0 | -14.2097 | -7.69524 |
| | 2 | .6015582 | .25453354 | 0.072 | -0.04805 | 1.251171 |

* The mean difference is significant at the 0.05 level.

| | | | | | |
|---|---|---|---|---|---|
| 0.068 | 0.547 | 0.98 | 1.455 | 1.796 | 2.644 |
| 0 | 0.479 | 0.912 | 1.387 | 1.728 | 2.576 |
| 0 | 7.5 | 15 | 30 | 60 | 120 |

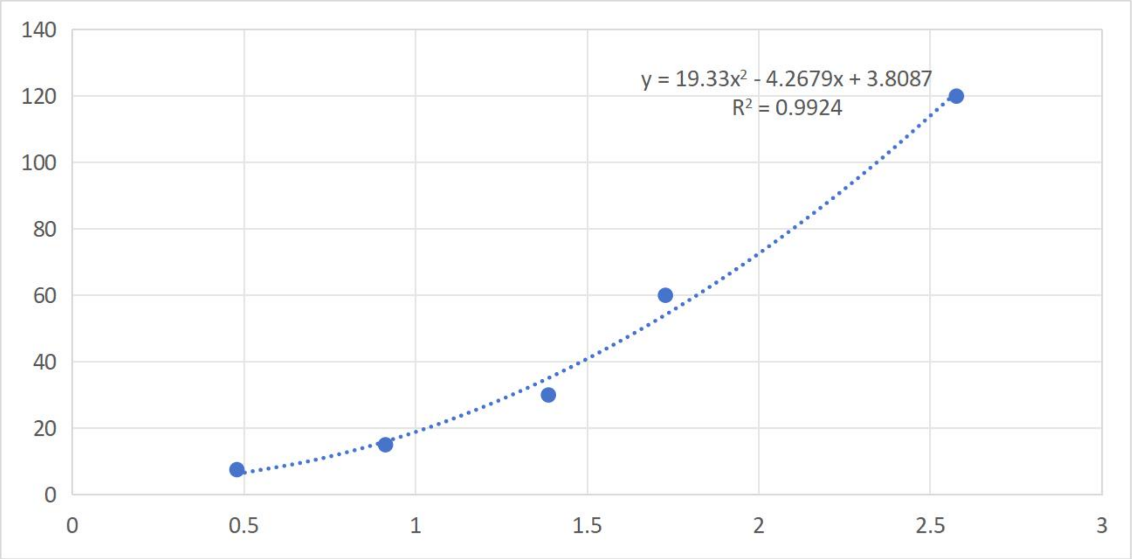


| Sample ID | OD (450r | Blank Cor | Dilution Fact | Concentration | Blank Corrected |
|---|---|---|---|---|---|
| 1 | 0.795 | 0.727 | 10.92240227 | 54.61201135 | 0.731 |
| 2 | 0.796 | 0.728 | 10.94625952 | 54.7312976 | 0.732 |
| 3 | 0.799 | 0.731 | 11.01806323 | 55.09031615 | 0.735 |
| 4 | 0.812 | 0.744 | 11.33323328 | 56.6661664 | 0.748 |
| 5 | 0.815 | 0.747 | 11.40689267 | 57.03446335 | 0.751 |
| 6 | 0.813 | 0.745 | 11.35774775 | 56.78873875 | 0.749 |
| 7 | 0.817 | 0.749 | 11.45619223 | 57.28096115 | 0.753 |
| 8 | 0.820 | 0.752 | 11.53043152 | 57.6521576 | 0.756 |
| 9 | 0.809 | 0.741 | 11.25992183 | 56.29960915 | 0.745 |
| 10 | 0.785 | 0.717 | 10.68595607 | 53.42978035 | 0.721 |
| 11 | 0.784 | 0.716 | 10.66252408 | 53.3126204 | 0.72 |
| 12 | 0.782 | 0.714 | 10.61577608 | 53.0788804 | 0.718 |
| 13 | 0.806 | 0.738 | 11.18695832 | 55.9347916 | 0.742 |
| 14 | 0.801 | 0.733 | 11.06612567 | 55.33062835 | 0.737 |
| 15 | 0.804 | 0.736 | 11.13850928 | 55.6925464 | 0.74 |
| 16 | 0.819 | 0.751 | 11.50564643 | 57.52823215 | 0.755 |
| 17 | 0.813 | 0.745 | 11.35774775 | 56.78873875 | 0.749 |
| 18 | 0.817 | 0.749 | 11.45619223 | 57.28096115 | 0.753 |
| 19 | 0.828 | 0.76 | 11.730104 | 58.65052 | 0.764 |
| 20 | 0.823 | 0.755 | 11.60501875 | 58.02509375 | 0.759 |
| 21 | 0.829 | 0.761 | 11.75523703 | 58.77618515 | 0.765 |
| 22 | 0.778 | 0.71 | 10.522744 | 52.61372 | 0.714 |
| 23 | 0.773 | 0.705 | 10.40732375 | 52.03661875 | 0.709 |
| 24 | 0.770 | 0.702 | 10.33853552 | 51.6926776 | 0.706 |
| 1 | 0.462 | 0.394 | 5.12785928 | 25.6392964 | 0.398 |
| 2 | 0.475 | 0.407 | 5.27365987 | 26.36829935 | 0.411 |
| 3 | 0.489 | 0.421 | 5.43798263 | 27.18991315 | 0.425 |
| 4 | 0.405 | 0.337 | 4.56570647 | 22.82853235 | 0.341 |
| 5 | 0.397 | 0.329 | 4.49685943 | 22.48429715 | 0.333 |
| 6 | 0.413 | 0.345 | 4.63702775 | 23.18513875 | 0.349 |
| 7 | 0.476 | 0.408 | 5.28514592 | 26.4257296 | 0.412 |

| | | | | | |
|---|---|---|---|---|---|
| 8 | 0.471 | 0.403 | 5.22810227 | 26.14051135 | 0.407 |
| 9 | 0.469 | 0.401 | 5.20555543 | 26.02777715 | 0.405 |
| 10 | 0.439 | 0.371 | 4.88590963 | 24.42954815 | 0.375 |
| 11 | 0.434 | 0.366 | 4.83601808 | 24.1800904 | 0.37 |
| 12 | 0.428 | 0.36 | 4.777424 | 23.88712 | 0.364 |
| 13 | 0.425 | 0.357 | 4.74864887 | 23.74324435 | 0.361 |
| 14 | 0.428 | 0.36 | 4.777424 | 23.88712 | 0.364 |
| 15 | 0.431 | 0.363 | 4.80654707 | 24.03273535 | 0.367 |
| 16 | 0.448 | 0.38 | 4.97815 | 24.89075 | 0.384 |
| 17 | 0.464 | 0.396 | 5.14986488 | 25.7493244 | 0.4 |
| 18 | 0.459 | 0.391 | 5.09514083 | 25.47570415 | 0.395 |
| 19 | 0.483 | 0.415 | 5.36663075 | 26.83315375 | 0.419 |
| 20 | 0.494 | 0.426 | 5.49850568 | 27.4925284 | 0.43 |
| 21 | 0.497 | 0.429 | 5.53528343 | 27.67641715 | 0.433 |
| 22 | 0.479 | 0.411 | 5.31983603 | 26.59918015 | 0.415 |
| 23 | 0.472 | 0.404 | 5.23943368 | 26.1971684 | 0.408 |
| 24 | 0.475 | 0.407 | 5.27365987 | 26.36829935 | 0.411 |
| 25 | 0.455 | 0.387 | 5.05205747 | 25.26028735 | 0.391 |
| 26 | 0.448 | 0.38 | 4.97815 | 24.89075 | 0.384 |
| 27 | 0.431 | 0.363 | 4.80654707 | 24.03273535 | 0.367 |
| 28 | 0.504 | 0.436 | 5.62245128 | 28.1122564 | 0.44 |
| 29 | 0.512 | 0.444 | 5.72439128 | 28.6219564 | 0.448 |
| 30 | 0.509 | 0.441 | 5.68587383 | 28.42936915 | 0.445 |
| 1 | 0.552 | 0.484 | 6.27120488 | 31.3560244 | 0.488 |
| 2 | 0.556 | 0.488 | 6.32928832 | 31.6464416 | 0.492 |
| 3 | 0.544 | 0.476 | 6.15689368 | 30.7844684 | 0.48 |
| 4 | 0.558 | 0.49 | 6.358562 | 31.79281 | 0.494 |
| 5 | 0.549 | 0.481 | 6.22804823 | 31.14024115 | 0.485 |
| 6 | 0.541 | 0.473 | 6.11466487 | 30.57332435 | 0.477 |
| 7 | 0.599 | 0.531 | 6.99275123 | 34.96375615 | 0.535 |
| 8 | 0.581 | 0.513 | 6.70632407 | 33.53162035 | 0.517 |
| 9 | 0.589 | 0.521 | 6.83207863 | 34.16039315 | 0.525 |
| 10 | 0.584 | 0.516 | 6.75319208 | 33.7659604 | 0.52 |
| 11 | 0.580 | 0.512 | 6.69077872 | 33.4538936 | 0.516 |
| 12 | 0.591 | 0.523 | 6.86390387 | 34.31951935 | 0.527 |
| 13 | 0.574 | 0.506 | 6.59831848 | 32.9915924 | 0.51 |
| 14 | 0.570 | 0.502 | 6.53745152 | 32.6872576 | 0.506 |
| 15 | 0.579 | 0.511 | 6.67527203 | 33.37636015 | 0.515 |
| 16 | 0.588 | 0.52 | 6.816224 | 34.08112 | 0.524 |
| 17 | 0.581 | 0.513 | 6.70632407 | 33.53162035 | 0.517 |
| 18 | 0.577 | 0.509 | 6.64437463 | 33.22187315 | 0.513 |
| 19 | 0.542 | 0.474 | 6.12870248 | 30.6435124 | 0.478 |
| 20 | 0.539 | 0.471 | 6.08670563 | 30.43352815 | 0.475 |
| 21 | 0.534 | 0.466 | 6.01748408 | 30.0874204 | 0.47 |
| 22 | 0.605 | 0.537 | 7.09101047 | 35.45505235 | 0.541 |
| 23 | 0.600 | 0.532 | 7.00903112 | 35.0451556 | 0.536 |
| 24 | 0.612 | 0.544 | 7.20740528 | 36.0370264 | 0.548 |
| 25 | 0.583 | 0.515 | 6.73753075 | 33.68765375 | 0.519 |
| 26 | 0.577 | 0.509 | 6.64437463 | 33.22187315 | 0.513 |
| 27 | 0.585 | 0.517 | 6.76889207 | 33.84446035 | 0.521 |
| 28 | 0.548 | 0.48 | 6.21374 | 31.0687 | 0.484 |
| 29 | 0.543 | 0.475 | 6.14277875 | 30.71389375 | 0.479 |
| 30 | 0.535 | 0.467 | 6.03125107 | 30.15625535 | 0.471 |

| | | | | | |
|---|---|---|---|---|---|
| 0.064 | 0.539 | 0.977 | 1.505 | 1.825 | 2.704 |
| 0 | 0.475 | 0.913 | 1.441 | 1.761 | 2.64 |
| 0 | 7.5 | 15 | 30 | 60 | 120 |

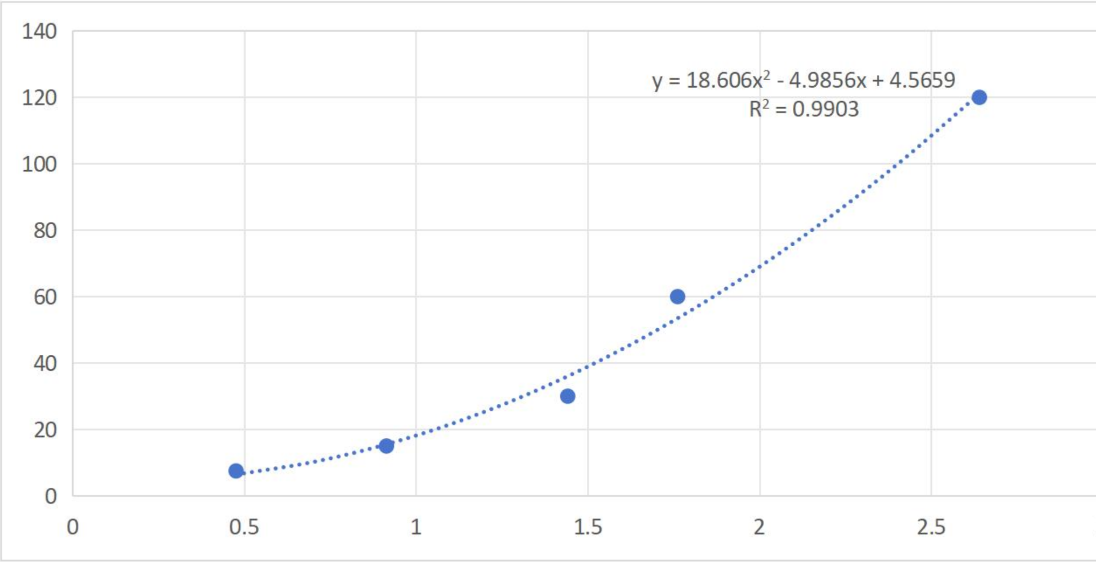


| Dilution Factor | Concentration | | | |
|---|---|---|---|---|
| 10.86374717 | 54.31873583 | 54.61201 | 54.31874 | 54.46537359 |
| 10.88598214 | 54.42991072 | 54.7313 | 54.42991 | 54.58060416 |
| 10.95291035 | 54.76455175 | 55.09032 | 54.76455 | 54.92743395 |
| 11.24680262 | 56.23401312 | 56.66617 | 56.23401 | 56.45008976 |
| 11.31551701 | 56.57758503 | 57.03446 | 56.57759 | 56.80602419 |
| 11.26967021 | 56.34835103 | 56.78874 | 56.34835 | 56.56854489 |
| 11.36151265 | 56.80756327 | 57.28096 | 56.80756 | 57.04426221 |
| 11.43078522 | 57.15392608 | 57.65216 | 57.15393 | 57.40304184 |
| 11.17842315 | 55.89211575 | 56.29961 | 55.89212 | 56.09586245 |
| 10.64344405 | 53.21722023 | 53.42978 | 53.21722 | 53.32350029 |
| 10.6216184 | 53.108092 | 53.31262 | 53.10809 | 53.2103562 |
| 10.57807874 | 52.89039372 | 53.07888 | 52.89039 | 52.98463706 |
| 11.11037858 | 55.55189292 | 55.93479 | 55.55189 | 55.74334226 |
| 10.99771521 | 54.98857607 | 55.33063 | 54.98858 | 55.15960221 |
| 11.0652016 | 55.326008 | 55.69255 | 55.32601 | 55.5092772 |
| 11.40765715 | 57.03828575 | 57.52823 | 57.03829 | 57.28325895 |
| 11.26967021 | 56.34835103 | 56.78874 | 56.34835 | 56.56854489 |
| 11.36151265 | 56.80756327 | 57.28096 | 56.80756 | 57.04426221 |
| 11.61714938 | 58.08574688 | 58.65052 | 58.08575 | 58.36813344 |
| 11.50039269 | 57.50196343 | 58.02509 | 57.50196 | 57.76352859 |
| 11.64061235 | 58.20306175 | 58.77619 | 58.20306 | 58.48962345 |
| 10.49144598 | 52.45722988 | 52.61372 | 52.45723 | 52.53547494 |
| 10.38399229 | 51.91996143 | 52.03662 | 51.91996 | 51.97829009 |
| 10.31996662 | 51.59983308 | 51.69268 | 51.59983 | 51.64625534 |
| 5.528896024 | 27.64448012 | 25.6393 | 27.64448 | 26.64188826 |
| 5.659762526 | 28.29881263 | 26.3683 | 28.29881 | 27.33355599 |
| 5.80772875 | 29.03864375 | 27.18991 | 29.03864 | 28.11427845 |
| 5.029334686 | 25.14667343 | 22.82853 | 25.14667 | 23.98760289 |
| 4.968895934 | 24.84447967 | 22.4843 | 24.84448 | 23.66438841 |
| 5.092155006 | 25.46077503 | 23.18514 | 25.46078 | 24.32295689 |
| 5.670089664 | 28.35044832 | 26.42573 | 28.35045 | 27.38808896 |

| | | | | |
|---|---|---|---|---|
| 5.618826094 | 28.09413047 | 26.14051 | 28.09413 | 27.11732091 |
| 5.59858115 | 27.99290575 | 26.02778 | 27.99291 | 27.01034145 |
| 5.31276875 | 26.56384375 | 24.42955 | 26.56384 | 25.49669595 |
| 5.2683894 | 26.341947 | 24.18009 | 26.34195 | 25.2610187 |
| 5.216362176 | 26.08181088 | 23.88712 | 26.08181 | 24.98446544 |
| 5.190850926 | 25.95425463 | 23.74324 | 25.95425 | 24.84874949 |
| 5.216362176 | 26.08181088 | 23.88712 | 26.08181 | 24.98446544 |
| 5.242208334 | 26.21104167 | 24.03274 | 26.21104 | 25.12188851 |
| 5.394995936 | 26.97497968 | 24.89075 | 26.97498 | 25.93286484 |
| 5.54862 | 27.7431 | 25.74932 | 27.7431 | 26.7462122 |
| 5.49958915 | 27.49794575 | 25.4757 | 27.49795 | 26.48682495 |
| 5.743421566 | 28.71710783 | 26.83315 | 28.71711 | 27.77513079 |
| 5.8623414 | 29.311707 | 27.49253 | 29.31171 | 28.4021177 |
| 5.895555534 | 29.47777767 | 27.67642 | 29.47778 | 28.57709741 |
| 5.70129435 | 28.50647175 | 26.59918 | 28.50647 | 27.55282595 |
| 5.629004384 | 28.14502192 | 26.19717 | 28.14502 | 27.17109516 |
| 5.659762526 | 28.29881263 | 26.3683 | 28.29881 | 27.33355599 |
| 5.461034286 | 27.30517143 | 25.26029 | 27.30517 | 26.28272939 |
| 5.394995936 | 26.97497968 | 24.89075 | 26.97498 | 25.93286484 |
| 5.242208334 | 26.21104167 | 24.03274 | 26.21104 | 25.12188851 |
| 5.9743576 | 29.871788 | 28.11226 | 29.87179 | 28.9920222 |
| 6.066649824 | 30.33324912 | 28.62196 | 30.33325 | 29.47760276 |
| 6.03176115 | 30.15880575 | 28.42937 | 30.15881 | 29.29408745 |
| 6.563834464 | 32.81917232 | 31.35602 | 32.81917 | 32.08759836 |
| 6.616827584 | 33.08413792 | 31.64644 | 33.08414 | 32.36528976 |
| 6.4596344 | 32.298172 | 30.78447 | 32.29817 | 31.5413202 |
| 6.643547416 | 33.21773708 | 31.79281 | 33.21774 | 32.50527354 |
| 6.52448035 | 32.62240175 | 31.14024 | 32.6224 | 31.88132145 |
| 6.421173374 | 32.10586687 | 30.57332 | 32.10587 | 31.33959561 |
| 7.22410635 | 36.12053175 | 34.96376 | 36.12053 | 35.54214395 |
| 6.961523934 | 34.80761967 | 33.53162 | 34.80762 | 34.16962001 |
| 7.07673875 | 35.38369375 | 34.16039 | 35.38369 | 34.77204345 |
| 7.0044504 | 35.022252 | 33.76596 | 35.02225 | 34.3941062 |
| 6.947289536 | 34.73644768 | 33.45389 | 34.73645 | 34.09517064 |
| 7.105914574 | 35.52957287 | 34.31952 | 35.52957 | 34.92454611 |
| 6.8626646 | 34.313323 | 32.99159 | 34.31332 | 33.6524577 |
| 6.806992216 | 34.03496108 | 32.68726 | 34.03496 | 33.36110934 |
| 6.93309235 | 34.66546175 | 33.37636 | 34.66546 | 34.02091095 |
| 7.062206656 | 35.31103328 | 34.08112 | 35.31103 | 34.69607664 |
| 6.961523934 | 34.80761967 | 33.53162 | 34.80762 | 34.16962001 |
| 6.904809614 | 34.52404807 | 33.22187 | 34.52405 | 33.87296061 |
| 6.433956504 | 32.16978252 | 30.64351 | 32.16978 | 31.40664746 |
| 6.39571875 | 31.97859375 | 30.43353 | 31.97859 | 31.20606095 |
| 6.3327334 | 31.663667 | 30.08742 | 31.66367 | 30.8755437 |
| 7.314313086 | 36.57156543 | 35.45505 | 36.57157 | 36.01330889 |
| 7.239047776 | 36.19523888 | 35.04516 | 36.19524 | 35.62019724 |
| 7.421247424 | 37.10623712 | 36.03703 | 37.10624 | 36.57163176 |
| 6.990104366 | 34.95052183 | 33.68765 | 34.95052 | 34.31908779 |
| 6.904809614 | 34.52404807 | 33.22187 | 34.52405 | 33.87296061 |
| 7.018833646 | 35.09416823 | 33.84446 | 35.09417 | 34.46931429 |
| 6.511436736 | 32.55718368 | 31.0687 | 32.55718 | 31.81294184 |
| 6.446776846 | 32.23388423 | 30.71389 | 32.23388 | 31.47388899 |
| 6.345256046 | 31.72628023 | 30.15626 | 31.72628 | 30.94126779 |

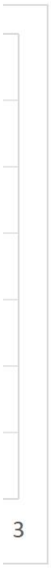


| | | | | |
|---|---|---|---|---|
| 1 | 54.46537 | 54.46537359 | 54.58060416 | 54.92743395 |
| | 54.5806 | 56.45008976 | 56.80602419 | 56.56854489 |
| | 54.92743 | 57.04426221 | 57.40304184 | 56.09586245 |
| 2 | 56.45009 | 53.32350029 | 53.2103562 | 52.98463706 |
| | 56.80602 | 55.74334226 | 55.15960221 | 55.5092772 |
| | 56.56854 | 57.28325895 | 56.56854489 | 57.04426221 |
| 3 | 57.04426 | 58.36813344 | 57.76352859 | 58.48962345 |
| | 57.40304 | 52.53547494 | 51.97829009 | 51.64625534 |
| | 56.09586 | 26.64188826 | 27.33355599 | 28.11427845 |
| 4 | 53.3235 | 23.98760289 | 23.66438841 | 24.32295689 |
| | 53.21036 | 27.38808896 | 27.11732091 | 27.01034145 |
| | 52.98464 | 25.49669595 | 25.2610187 | 24.98446544 |
| 5 | 55.74334 | 24.84874949 | 24.98446544 | 25.12188851 |
| | 55.1596 | 25.93286484 | 26.7462122 | 26.48682495 |
| | 55.50928 | 27.77513079 | 28.4021177 | 28.57709741 |
| 6 | 57.28326 | 27.55282595 | 27.17109516 | 27.33355599 |
| | 56.56854 | 26.28272939 | 25.93286484 | 25.12188851 |
| | 57.04426 | 28.9920222 | 29.47760276 | 29.29408745 |
| 7 | 58.36813 | 32.08759836 | 32.36528976 | 31.5413202 |
| | 57.76353 | 32.50527354 | 31.88132145 | 31.33959561 |
| | 58.48962 | 35.54214395 | 34.16962001 | 34.77204345 |
| 8 | 52.53547 | 34.3941062 | 34.09517064 | 34.92454611 |
| | 51.97829 | 33.6524577 | 33.36110934 | 34.02091095 |
| | 51.64626 | 34.69607664 | 34.16962001 | 33.87296061 |
| 9 | 26.64189 | 31.40664746 | 31.20606095 | 30.8755437 |
| | 27.33356 | 36.01330889 | 35.62019724 | 36.57163176 |
| | 28.11428 | 34.31908779 | 33.87296061 | 34.46931429 |
| 10 | 23.9876 | 31.81294184 | 31.47388899 | 30.94126779 |
| | 23.66439 | | | |
| | 24.32296 | | | |
| 11 | 27.38809 | | | |

| | |
|---|---|
| | 27.11732 |
| | 27.01034 |
| 12 | 25.4967 |
| | 25.26102 |
| | 24.98447 |
| 13 | 24.84875 |
| | 24.98447 |
| | 25.12189 |
| 14 | 25.93286 |
| | 26.74621 |
| | 26.48682 |
| 15 | 27.77513 |
| | 28.40212 |
| | 28.5771 |
| 16 | 27.55283 |
| | 27.1711 |
| | 27.33356 |
| 17 | 26.28273 |
| | 25.93286 |
| | 25.12189 |
| 18 | 28.99202 |
| | 29.4776 |
| | 29.29409 |
| 19 | 32.0876 |
| | 32.36529 |
| | 31.54132 |
| 20 | 32.50527 |
| | 31.88132 |
| | 31.3396 |
| 21 | 35.54214 |
| | 34.16962 |
| | 34.77204 |
| 22 | 34.39411 |
| | 34.09517 |
| | 34.92455 |
| 23 | 33.65246 |
| | 33.36111 |
| | 34.02091 |
| 24 | 34.69608 |
| | 34.16962 |
| | 33.87296 |
| 25 | 31.40665 |
| | 31.20606 |
| | 30.87554 |
| 26 | 36.01331 |
| | 35.6202 |
| | 36.57163 |
| 27 | 34.31909 |
| | 33.87296 |
| | 34.46931 |
| 28 | 31.81294 |
| | 31.47389 |
| | 30.94127 |

| |
|---|
| 54.6578039 |
| 56.60821961 |
| 56.84772217 |
| 53.17283118 |
| 55.47074056 |
| 56.96535535 |
| 58.20709516 |
| 52.05334012 |
| 27.3632409 |
| 23.9916494 |
| 27.17191711 |
| 25.24739336 |
| 24.98503448 |
| 26.388634 |
| 28.25144863 |
| 27.35249237 |
| 25.77916091 |
| 29.2545708 |
| 31.99806944 |
| 31.9087302 |
| 34.8279358 |
| 34.47127432 |
| 33.67815933 |
| 34.24621909 |
| 31.1627507 |
| 36.0683793 |
| 34.22045423 |
| 31.40936621 |

Report

FT4 (28)

| Group | Mean | Std. Deviation |
|---|---|---|
| 1 | 55.497888 | 2.0882470441629 |
| 2 | 26.578554 | 1.6057156143398 |
| 3 | 33.399133 | 1.6643542837555 |
| Total | 37.277142 | 12.2165842312046 |

Tests of Normality

| | Group | Kolmogorov-Smirnova | | | Shapiro-Wilk | | |
|---|---|---|---|---|---|---|---|
| | | Statistic | df | Sig. | Statistic | df | Sig. |
| FT4 (28) | 1 | 0.203 | 8 | .200* | 0.945 | 8 | 0.66 |
| | 2 | 0.144 | 10 | .200* | 0.981 | 10 | 0.972 |
| | 3 | 0.2 | 10 | .200* | 0.919 | 10 | 0.347 |

* This is a lower bound of the true significance.

a Lilliefors Significance Correction

ANOVA

FT4 (28)

| | Sum of Sq | df | Mean Squa | F | Sig. |
|---|---|---|---|---|---|
| Between G | 3950.952 | 2 | 1975.476 | 627.845 | 0 |
| Within Gr | 78.661 | 25 | 3.146 | | |
| Total | 4029.613 | 27 | | | |

Multiple Comparisons

Dependent Variable: FT4 (28)

Tukey HSD

| (I) Group | (J) Group | Mean Diff | Std. Erro | Sig. | 95% Confidence Interval | |
|---|---|---|---|---|---|---|
| | | | | | Lower Bou | Upper Bound |
| 1 | 2 | 28.91933 | 4.8413971 | 0 | 26.823557 | 31.015111571338 |
| | 3 | 22.09875 | 4.8413971 | 0 | 20.002977 | 24.194531905338 |
| 2 | 1 | -28.9193 | 3.8413971 | 0 | -31.0151 | -26.8236 |
| | 3 | -6.82057 | 9.7932768 | 0 | -8.7965 | -4.84466 |
| 3 | 1 | -22.0987 | 5.8413971 | 0 | -24.1945 | -20.003 |
| | 2 | 6.820579 | 6.7932768 | 0 | 4.844662 | 8.796497 |

* The mean difference is significant at the 0.05 level.

| | | | | | |
|---|---|---|---|---|---|
| 0.069 | 0.477 | 0.954 | 1.501 | 1.925 | 2.649 |
| 0 | 0.408 | 0.885 | 1.432 | 1.856 | 2.58 |
| 0 | 1.25 | 2.5 | 5 | 10 | 20 |

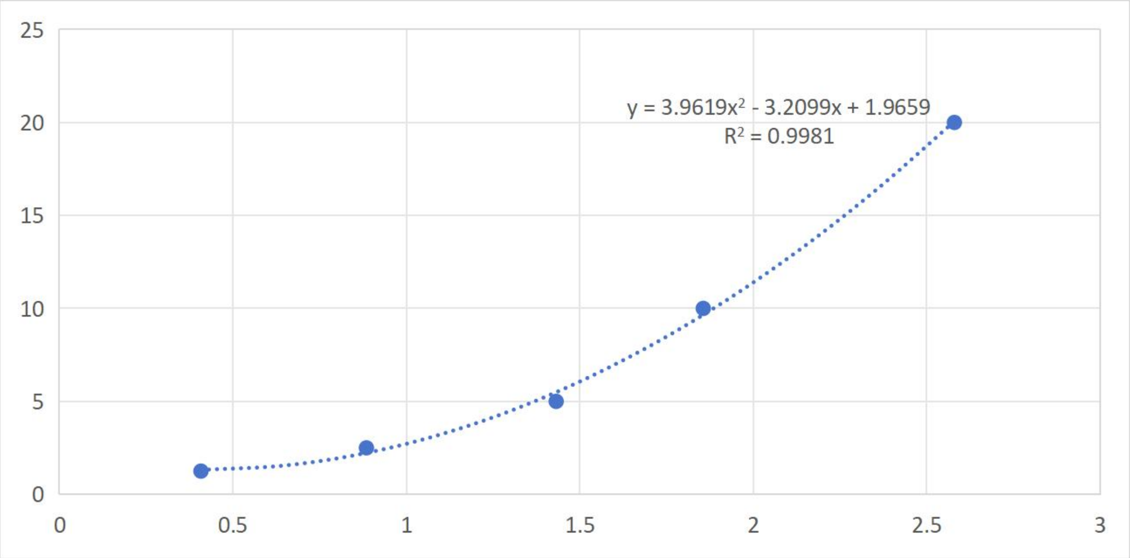


| Sample ID | OD (450nm) | Blank Corrected | Dilution Factor | Concentration |
|---|---|---|---|---|
| 1 | 0.745 | 0.676 | 1.606500814 | 8.032504072 |
| 2 | 0.756 | 0.687 | 1.630592681 | 8.152963406 |
| 3 | 0.747 | 0.678 | 1.61080984 | 8.054049198 |
| 4 | 0.754 | 0.685 | 1.626141028 | 8.130705138 |
| 5 | 0.778 | 0.709 | 1.681652754 | 8.40826377 |
| 6 | 0.767 | 0.698 | 1.655643328 | 8.278216638 |
| 7 | 0.782 | 0.713 | 1.691348441 | 8.456742206 |
| 8 | 0.781 | 0.712 | 1.688912634 | 8.444563168 |
| 9 | 0.787 | 0.718 | 1.703646336 | 8.518231678 |
| 10 | 0.734 | 0.665 | 1.583367728 | 7.916838638 |
| 11 | 0.789 | 0.72 | 1.70862096 | 8.5431048 |
| 12 | 0.746 | 0.677 | 1.608651365 | 8.043256826 |
| 13 | 0.803 | 0.734 | 1.744330796 | 8.721653982 |
| 14 | 0.809 | 0.74 | 1.76011044 | 8.8005522 |
| 15 | 0.800 | 0.731 | 1.736547946 | 8.68273973 |
| 16 | 0.763 | 0.694 | 1.646423068 | 8.232115342 |
| 17 | 0.793 | 0.724 | 1.718665294 | 8.593326472 |
| 18 | 0.780 | 0.711 | 1.68648475 | 8.43242375 |
| 19 | 0.772 | 0.703 | 1.667346937 | 8.336734686 |
| 20 | 0.759 | 0.69 | 1.63732959 | 8.18664795 |
| 21 | 0.767 | 0.698 | 1.655643328 | 8.278216638 |
| 22 | 0.781 | 0.712 | 1.688912634 | 8.444563168 |
| 23 | 0.779 | 0.71 | 1.68406479 | 8.42032395 |
| 24 | 0.765 | 0.696 | 1.65101735 | 8.255086752 |
| 1 | 1.096 | 1.027 | 2.848063525 | 14.24031763 |
| 2 | 1.102 | 1.033 | 2.877773209 | 14.38886605 |
| 3 | 1.098 | 1.029 | 2.857935058 | 14.28967529 |
| 4 | 1.096 | 1.027 | 2.848063525 | 14.24031763 |

| | | | | |
|---|---|---|---|---|
| 5 | 1.112 | 1.043 | 2.927923253 | 14.63961627 |
| 6 | 1.129 | 1.06 | 3.01499684 | 15.0749842 |
| 7 | 1.161 | 1.092 | 3.185112322 | 15.92556161 |
| 8 | 1.192 | 1.123 | 3.357649285 | 16.78824643 |
| 9 | 1.196 | 1.127 | 3.380466785 | 16.90233393 |
| 10 | 1.094 | 1.025 | 2.838223688 | 14.19111844 |
| 11 | 1.198 | 1.129 | 3.391923078 | 16.95961539 |
| 12 | 1.101 | 1.032 | 2.872801786 | 14.36400893 |
| 13 | 1.159 | 1.09 | 3.17424239 | 15.87121195 |
| 14 | 1.153 | 1.084 | 3.141822766 | 15.70911383 |
| 15 | 1.165 | 1.096 | 3.20694727 | 16.03473635 |
| 16 | 1.119 | 1.05 | 2.96349975 | 14.81749875 |
| 17 | 1.173 | 1.104 | 3.25099751 | 16.25498755 |
| 18 | 1.162 | 1.093 | 3.190559173 | 15.95279587 |
| 19 | 1.187 | 1.118 | 3.329305696 | 16.64652848 |
| 20 | 1.198 | 1.129 | 3.391923078 | 16.95961539 |
| 21 | 1.185 | 1.116 | 3.318023726 | 16.59011863 |
| 22 | 1.171 | 1.102 | 3.239937408 | 16.19968704 |
| 23 | 1.169 | 1.1 | 3.228909 | 16.144545 |
| 24 | 1.175 | 1.106 | 3.262089308 | 16.31044654 |
| 25 | 1.102 | 1.033 | 2.877773209 | 14.38886605 |
| 26 | 1.109 | 1.04 | 2.91279504 | 14.5639752 |
| 27 | 1.093 | 1.024 | 2.833315654 | 14.16657827 |
| 28 | 1.117 | 1.048 | 2.953295418 | 14.76647709 |
| 29 | 1.076 | 1.007 | 2.751091433 | 13.75545717 |
| 30 | 1.089 | 1.02 | 2.81376276 | 14.0688138 |
| 1 | 1.036 | 0.967 | 2.566655809 | 12.83327905 |
| 2 | 1.048 | 0.979 | 2.620655298 | 13.10327649 |
| 3 | 1.042 | 0.973 | 2.593512925 | 12.96756463 |
| 4 | 1.025 | 0.956 | 2.518158638 | 12.59079319 |
| 5 | 1.022 | 0.953 | 2.505098537 | 12.52549269 |
| 6 | 1.029 | 0.973 | 2.593512925 | 12.96756463 |
| 7 | 1.089 | 1.02 | 2.81376276 | 14.0688138 |
| 8 | 1.097 | 1.028 | 2.85299533 | 14.26497665 |
| 9 | 1.09 | 1.021 | 2.818639098 | 14.09319549 |
| 10 | 1.022 | 0.953 | 2.505098537 | 12.52549269 |
| 11 | 1.019 | 0.95 | 2.49210975 | 12.46054875 |
| 12 | 1.013 | 0.944 | 2.466346118 | 12.33173059 |
| 13 | 1.045 | 0.976 | 2.607048454 | 13.03524227 |
| 14 | 1.047 | 0.978 | 2.61611176 | 13.0805588 |
| 15 | 1.046 | 0.977 | 2.611576145 | 13.05788073 |
| 16 | 1.078 | 1.009 | 2.760646014 | 13.80323007 |
| 17 | 1.085 | 1.016 | 2.794336646 | 13.97168323 |
| 18 | 1.073 | 1.004 | 2.73681899 | 13.68409495 |
| 19 | 1.012 | 0.943 | 2.462079913 | 12.31039957 |
| 20 | 1.003 | 0.934 | 2.424040636 | 12.12020318 |
| 21 | 1.021 | 0.952 | 2.500761018 | 12.50380509 |
| 22 | 1.038 | 0.969 | 2.575576486 | 12.87788243 |
| 23 | 1.033 | 0.964 | 2.553334222 | 12.76667111 |
| 24 | 1.03 | 0.961 | 2.54008395 | 12.70041975 |
| 25 | 1.047 | 0.978 | 2.61611176 | 13.0805588 |
| 26 | 1.052 | 0.983 | 2.638908689 | 13.19454345 |
| 27 | 1.068 | 0.999 | 2.713190062 | 13.56595031 |
| 28 | 1.094 | 1.025 | 2.838223688 | 14.19111844 |

| | | | | |
|---|---|---|---|---|
| 29 | 1.1 | 1.031 | 2.867838286 | 14.33919143 |
| 30 | 1.098 | 1.029 | 2.857935058 | 14.28967529 |

| | | | | |
|---|---|---|---|---|
| 0.068 | 0.456 | 0.949 | 1.523 | 1.955 |
| 0 | 0.388 | 0.881 | 1.455 | 1.887 |
| 0 | 1.25 | 2.5 | 5 | 10 |

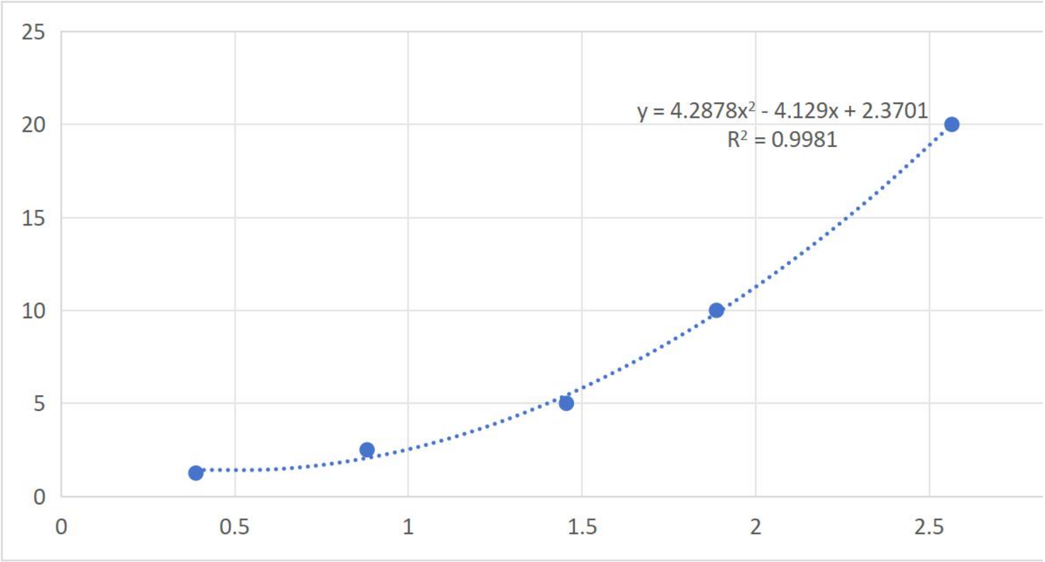


| Blank Corrected | Dilution Factor | Concentration | | |
|---|---|---|---|---|
| 0.677 | 1.539990086 | 7.699950431 | 8.03250407 | 7.69995043 |
| 0.688 | 1.558952403 | 7.794762016 | 8.15296341 | 7.79476202 |
| 0.679 | 1.5433606 | 7.716802999 | 8.0540492 | 7.716803 |
| 0.686 | 1.555427529 | 7.777137644 | 8.13070514 | 7.77713764 |
| 0.71 | 1.59998998 | 7.9999499 | 8.40826377 | 7.9999499 |
| 0.699 | 1.578952368 | 7.894761839 | 8.27821664 | 7.89476184 |
| 0.714 | 1.607897289 | 8.039486444 | 8.45674221 | 8.03948644 |
| 0.713 | 1.605907598 | 8.029537991 | 8.44456317 | 8.02953799 |
| 0.719 | 1.617974376 | 8.089871879 | 8.51823168 | 8.08987188 |
| 0.666 | 1.522065417 | 7.610327084 | 7.91683864 | 7.61032708 |
| 0.721 | 1.62206524 | 8.110326199 | 8.5431048 | 8.1103262 |
| 0.678 | 1.541671055 | 7.708355276 | 8.04325683 | 7.70835528 |
| 0.735 | 1.651661755 | 8.258308775 | 8.72165398 | 8.25830878 |
| 0.741 | 1.664860512 | 8.324302559 | 8.8005522 | 8.32430256 |
| 0.732 | 1.645178147 | 8.225890736 | 8.68273973 | 8.22589074 |
| 0.695 | 1.571559595 | 7.857797975 | 8.23211534 | 7.85779798 |
| 0.725 | 1.630349875 | 8.151749375 | 8.59332647 | 8.15174938 |
| 0.712 | 1.603926483 | 8.019632416 | 8.43242375 | 8.01963242 |
| 0.704 | 1.588386285 | 7.941931424 | 8.33673469 | 7.94193142 |
| 0.691 | 1.564304032 | 7.821520159 | 8.18664795 | 7.82152016 |
| 0.699 | 1.578952368 | 7.894761839 | 8.27821664 | 7.89476184 |
| 0.713 | 1.605907598 | 8.029537991 | 8.44456317 | 8.02953799 |
| 0.711 | 1.601953944 | 8.009769719 | 8.42032395 | 8.00976972 |
| 0.697 | 1.57523883 | 7.876194151 | 8.25508675 | 7.87619415 |
| 1.028 | 2.656766435 | 13.28383218 | 14.24031763 | 13.28383218 |
| 1.034 | 2.685041097 | 13.42520548 | 14.38886605 | 13.42520548 |
| 1.03 | 2.66615702 | 13.3307851 | 14.28967529 | 13.3307851 |
| 1.028 | 2.656766435 | 13.28383218 | 14.24031763 | 13.28383218 |

| | | | | |
|---|---|---|---|---|
| 1.044 | 2.732851581 | 13.6642579 | 14.63961627 | 13.6642579 |
| 1.061 | 2.816097504 | 14.08048752 | 15.0749842 | 14.08048752 |
| 1.093 | 2.979518982 | 14.89759491 | 15.92556161 | 14.89759491 |
| 1.124 | 3.146207613 | 15.73103806 | 16.78824643 | 15.73103806 |
| 1.128 | 3.168316115 | 15.84158058 | 16.90233393 | 15.84158058 |
| 1.026 | 2.647410153 | 13.23705076 | 14.19111844 | 13.23705076 |
| 1.13 | 3.17942182 | 15.8971091 | 16.95961539 | 15.8971091 |
| 1.033 | 2.680307214 | 13.40153607 | 14.36400893 | 13.40153607 |
| 1.091 | 2.969047872 | 14.84523936 | 15.87121195 | 14.84523936 |
| 1.085 | 2.937840355 | 14.68920178 | 15.70911383 | 14.68920178 |
| 1.097 | 3.00056411 | 15.00282055 | 16.03473635 | 15.00282055 |
| 1.051 | 2.766829168 | 13.83414584 | 14.81749875 | 13.83414584 |
| 1.105 | 3.043065995 | 15.21532998 | 16.25498755 | 15.21532998 |
| 1.094 | 2.984767401 | 14.923837 | 15.95279587 | 14.923837 |
| 1.119 | 3.118764936 | 15.59382468 | 16.64652848 | 15.59382468 |
| 1.13 | 3.17942182 | 15.8971091 | 16.95961539 | 15.8971091 |
| 1.117 | 3.107847894 | 15.53923947 | 16.59011863 | 15.53923947 |
| 1.103 | 3.03238907 | 15.16194535 | 16.19968704 | 15.16194535 |
| 1.101 | 3.021746448 | 15.10873224 | 16.144545 | 15.10873224 |
| 1.107 | 3.053777222 | 15.26888611 | 16.31044654 | 15.26888611 |
| 1.034 | 2.685041097 | 13.42520548 | 14.38886605 | 13.42520548 |
| 1.041 | 2.718418392 | 13.59209196 | 14.5639752 | 13.59209196 |
| 1.025 | 2.642744875 | 13.21372438 | 14.16657827 | 13.21372438 |
| 1.049 | 2.757078408 | 13.78539204 | 14.76647709 | 13.78539204 |
| 1.008 | 2.564747219 | 12.8237361 | 13.75545717 | 12.8237361 |
| 1.021 | 2.62416952 | 13.1208476 | 14.0688138 | 13.1208476 |
| 0.968 | 2.390999507 | 11.95499754 | 12.83327905 | 11.95499754 |
| 0.98 | 2.44168312 | 12.2084156 | 13.10327649 | 12.2084156 |
| 0.974 | 2.416186953 | 12.08093476 | 12.96756463 | 12.08093476 |
| 0.957 | 2.345624342 | 11.72812171 | 12.59079319 | 11.72812171 |
| 0.954 | 2.333429385 | 11.66714692 | 12.52549269 | 11.66714692 |
| 0.961 | 2.362004344 | 11.81002172 | 12.96756463 | 11.81002172 |
| 1.021 | 2.62416952 | 13.1208476 | 14.0688138 | 13.1208476 |
| 1.029 | 2.66145744 | 13.3072872 | 14.26497665 | 13.3072872 |
| 1.022 | 2.628800495 | 13.14400248 | 14.09319549 | 13.14400248 |
| 0.954 | 2.333429385 | 11.66714692 | 12.52549269 | 11.66714692 |
| 0.951 | 2.321311608 | 11.60655804 | 12.46054875 | 11.60655804 |
| 0.945 | 2.297307595 | 11.48653798 | 12.33173059 | 11.48653798 |
| 0.977 | 2.428896446 | 12.14448223 | 13.03524227 | 12.14448223 |
| 0.979 | 2.43741232 | 12.1870616 | 13.0805588 | 12.1870616 |
| 0.978 | 2.433150095 | 12.16575048 | 13.05788073 | 12.16575048 |
| 1.01 | 2.57379478 | 12.8689739 | 13.80323007 | 12.8689739 |
| 1.017 | 2.605731374 | 13.02865687 | 13.97168323 | 13.02865687 |
| 1.005 | 2.551240195 | 12.75620098 | 13.68409495 | 12.75620098 |
| 0.944 | 2.293336941 | 11.4666847 | 12.31039957 | 11.4666847 |
| 0.935 | 2.257986955 | 11.28993478 | 12.12020318 | 11.28993478 |
| 0.953 | 2.32938155 | 11.64690775 | 12.50380509 | 11.64690775 |
| 0.97 | 2.39936102 | 11.9968051 | 12.87788243 | 11.9968051 |
| 0.965 | 2.378521555 | 11.89260778 | 12.76667111 | 11.89260778 |
| 0.962 | 2.366120783 | 11.83060392 | 12.70041975 | 11.83060392 |
| 0.979 | 2.43741232 | 12.1870616 | 13.0805588 | 12.1870616 |
| 0.984 | 2.458852077 | 12.29426038 | 13.19454345 | 12.29426038 |
| 1 | 2.5289 | 12.6445 | 13.56595031 | 12.6445 |
| 1.026 | 2.647410153 | 13.23705076 | 14.19111844 | 13.23705076 |

| | | | | | |
|---|---|---|---|---|---|
| 1.032 | 2.675581907 | 13.37790954 | | 14.33919143 | 13.37790954 |
| 1.03 | 2.66615702 | 13.3307851 | | 14.28967529 | 13.3307851 |

2.633
2.565
20

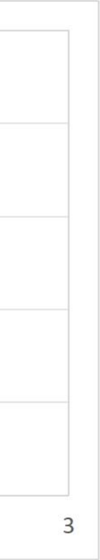


| | | | | |
|---|---|---|---|---|
| 7.86622725 | 1 | 7.86622725 | 7.86622725 | 7.97386272 |
| 7.973862715 | | 7.97386272 | 7.95392139 | 8.20410684 |
| 7.8854261 | | 7.8854261 | 8.24811433 | 8.23705058 |
| 7.95392139 | 2 | 7.95392139 | 7.76358286 | 8.3267155 |
| 8.204106835 | | 8.20410684 | 8.48998138 | 8.56242738 |
| 8.08648924 | | 8.08648924 | 8.04495666 | 8.37253793 |
| 8.248114325 | 3 | 8.24811433 | 8.13933306 | 8.00408406 |
| 8.23705058 | | 8.23705058 | 8.23705058 | 8.21504684 |
| 8.30405178 | | 8.30405178 | 13.76207491 | 13.90703577 |
| 7.76358286 | 4 | 7.76358286 | 13.76207491 | 14.15193709 |
| 8.3267155 | | 8.3267155 | 15.41157826 | 16.25964225 |
| 7.875806055 | | 7.87580606 | 13.7140846 | 16.42836225 |
| 8.48998138 | 5 | 8.48998138 | 15.35822566 | 15.19915781 |
| 8.56242738 | | 8.56242738 | 14.3258223 | 15.73515877 |
| 8.454315235 | | 8.45431524 | 16.12017658 | 16.42836225 |
| 8.04495666 | 6 | 8.04495666 | 15.6808162 | 15.62663862 |
| 8.372537925 | | 8.37253793 | 13.90703577 | 14.07803358 |
| 8.226028085 | | 8.22602809 | 14.27593457 | 13.28959664 |
| 8.139333055 | 7 | 8.13933306 | 12.3941383 | 12.65584605 |
| 8.004084055 | | 8.00408406 | 12.15945745 | 12.09631981 |
| 8.08648924 | | 8.08648924 | 13.5948307 | 13.78613193 |
| 8.23705058 | 8 | 8.23705058 | 12.09631981 | 12.0335534 |
| 8.215046835 | | 8.21504684 | 12.58986225 | 12.6338102 |
| 8.06564045 | | 8.06564045 | 13.33610199 | 13.50017005 |
| 13.76207491 | 9 | 13.76207491 | 11.88854214 | 11.70506898 |
| 13.90703577 | | 13.90703577 | 12.43734377 | 12.32963945 |
| 13.8102302 | | 13.8102302 | 12.6338102 | 12.74440192 |
| 13.76207491 | 10 | 13.76207491 | 13.7140846 | 13.85855049 |

| | | |
|---|---|---|
| 14.15193709 | | 14.15193709 |
| 14.57773586 | | 14.57773586 |
| 15.41157826 | 11 | 15.41157826 |
| 16.25964225 | | 16.25964225 |
| 16.37195726 | | 16.37195726 |
| 13.7140846 | 12 | 13.7140846 |
| 16.42836225 | | 16.42836225 |
| 13.8827725 | | 13.8827725 |
| 15.35822566 | 13 | 15.35822566 |
| 15.19915781 | | 15.19915781 |
| 15.51877845 | | 15.51877845 |
| 14.3258223 | 14 | 14.3258223 |
| 15.73515877 | | 15.73515877 |
| 15.43831644 | | 15.43831644 |
| 16.12017658 | 15 | 16.12017658 |
| 16.42836225 | | 16.42836225 |
| 16.06467905 | | 16.06467905 |
| 15.6808162 | 16 | 15.6808162 |
| 15.62663862 | | 15.62663862 |
| 15.78966633 | | 15.78966633 |
| 13.90703577 | 17 | 13.90703577 |
| 14.07803358 | | 14.07803358 |
| 13.69015133 | | 13.69015133 |
| 14.27593457 | 18 | 14.27593457 |
| 13.28959664 | | 13.28959664 |
| 13.5948307 | | 13.5948307 |
| 12.3941383 | 19 | 12.3941383 |
| 12.65584605 | | 12.65584605 |
| 12.5242497 | | 12.5242497 |
| 12.15945745 | 20 | 12.15945745 |
| 12.09631981 | | 12.09631981 |
| 12.38879318 | | 12.38879318 |
| 13.5948307 | 21 | 13.5948307 |
| 13.78613193 | | 13.78613193 |
| 13.61859899 | | 13.61859899 |
| 12.09631981 | 22 | 12.09631981 |
| 12.0335534 | | 12.0335534 |
| 11.90913429 | | 11.90913429 |
| 12.58986225 | 23 | 12.58986225 |
| 12.6338102 | | 12.6338102 |
| 12.61181561 | | 12.61181561 |
| 13.33610199 | 24 | 13.33610199 |
| 13.50017005 | | 13.50017005 |
| 13.22014797 | | 13.22014797 |
| 11.88854214 | 25 | 11.88854214 |
| 11.70506898 | | 11.70506898 |
| 12.07535642 | | 12.07535642 |
| 12.43734377 | 26 | 12.43734377 |
| 12.32963945 | | 12.32963945 |
| 12.26551184 | | 12.26551184 |
| 12.6338102 | 27 | 12.6338102 |
| 12.74440192 | | 12.74440192 |
| 13.10522516 | | 13.10522516 |
| 13.7140846 | 28 | 13.7140846 |

| | |
|---|---|
| 13.85855049 | 13.85855049 |
| 13.8102302 | 13.8102302 |

| | | |
|---|---|---|
| 7.8854261 | 7.908505357 | 1 |
| 8.08648924 | 8.081505823 | 2 |
| 8.30405178 | 8.26307223 | 3 |
| 7.87580606 | 7.988701473 | 4 |
| 8.45431524 | 8.502241333 | 5 |
| 8.22602809 | 8.21450756 | 6 |
| 8.08648924 | 8.076635453 | 7 |
| 8.06564045 | 8.17257929 | 8 |
| 13.8102302 | 13.82644696 | 9 |
| 14.57773586 | 14.16391595 | 10 |
| 16.37195726 | 16.01439259 | 11 |
| 13.8827725 | 14.67507312 | 12 |
| 15.51877845 | 15.35872064 | 13 |
| 15.43831644 | 15.1664325 | 14 |
| 16.06467905 | 16.20440596 | 15 |
| 15.78966633 | 15.69904038 | 16 |
| 13.69015133 | 13.89174023 | 17 |
| 13.5948307 | 13.72012064 | 18 |
| 12.5242497 | 12.52474468 | 19 |
| 12.38879318 | 12.21485681 | 20 |
| 13.61859899 | 13.66652054 | 21 |
| 11.90913429 | 12.0130025 | 22 |
| 12.61181561 | 12.61182935 | 23 |
| 13.22014797 | 13.35214 | 24 |
| 12.07535642 | 11.88965585 | 25 |
| 12.26551184 | 12.34416502 | 26 |
| 13.10522516 | 12.82781243 | 27 |
| 13.8102302 | 13.79428843 | 28 |

Report
TSH（28）

| Group | Mean | Std. Deviation |
|---|---|---|
| 1 | 8.150969 | .1835886383860 |
| 2 | 14.872028 | .9433409532229 |
| 3 | 12.723901 | .6746713618005 |
| Total | 12.184537 | 2.8391069197567 |

Tests of Normality

| | Group | Kolmogorov-Smirnova | | | Shapiro-Wilk | | |
|---|---|---|---|---|---|---|---|
| | | Statistic | df | Sig. | Statistic | df | Sig. |
| TSH（28） | 1 | 0.147 | 8 | .200* | 0.958 | 8 | 0.787 |
| | 2 | 0.174 | 10 | .200* | 0.909 | 10 | 0.277 |
| | 3 | 0.166 | 10 | .200* | 0.922 | 10 | 0.373 |

* This is a lower bound of the true significance.
a Lilliefors Significance Correction

ANOVA
TSH（28）

| | Sum of Sq | df | Mean Squa | F | Sig. |
|---|---|---|---|---|---|
| Between G | 205.293 | 2 | 102.646 | 207.928 | 0 |
| Within Gr | 12.342 | 25 | 0.494 | | |
| Total | 217.634 | 27 | | | |

Robust Tests of Equality of Means
TSH（28）

| | Statistic | df1 | df2 | Sig. |
|---|---|---|---|---|
| Welch | 406.171 | 2 | 13.466 | 0 |

a Asymptotically F distributed.

Multiple Comparisons
Dependent Variable: TSH（28）
Games-Howell

| (I) Group | (J) Group | Mean Diff | Std. Erro | Sig. | 95% Confidence Interval | |
|---|---|---|---|---|---|---|
| | | | | | Lower Bou | Upper Bound |
| 1 | 2 | -6.72106 | 0.30529054 | 0 | -7.56013 | -5.88199 |
| | 3 | -4.57293 | 2.22300502 | 0 | -5.17839 | -3.96748 |
| 2 | 1 | 6.721060 | 3.30529054 | 0 | 5.881987 | 7.560133 |
| | 3 | 2.148127 | 3.36675245 | 0 | 1.203495 | 3.092759 |
| 3 | 1 | 4.572932 | 9.22300502 | 0 | 3.967479 | 5.178387 |
| | 2 | -2.148127 | .36675245 | 0 | -3.09276 | -1.2035 |

* The mean difference is significant at the 0.05 level.

| | | | | | |
|---|---|---|---|---|---|
| 0.326 | 0.416 | 0.742 | 1.392 | 2.49 | 0.164 |
| 0.162 | 0.252 | 0.578 | 1.228 | 2.326 | 0 |
| 1.25 | 2.5 | 5 | 10 | 20 | |

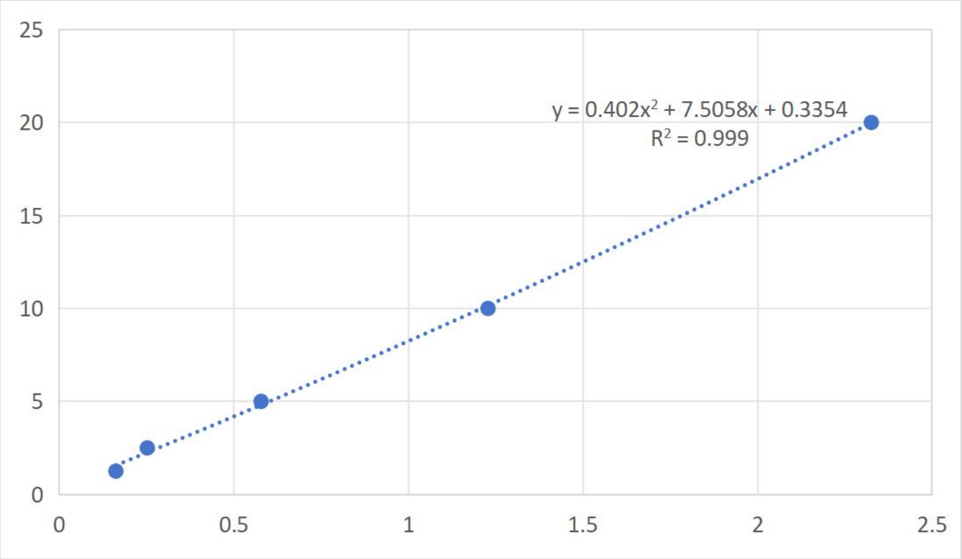


| Sample ID | OD (450nm) | Blank Corrected | Dilution Factor | Concentration |
|---|---|---|---|---|
| 1 | 0.591 | 0.427 | 3.613672858 | 18.06836429 |
| 2 | 0.597 | 0.433 | 3.660781978 | 18.30390989 |
| 3 | 0.594 | 0.43 | 3.6372238 | 18.186119 |
| 4 | 0.558 | 0.394 | 3.355090072 | 16.77545036 |
| 5 | 0.547 | 0.383 | 3.269090378 | 16.34545189 |
| 6 | 0.558 | 0.394 | 3.355090072 | 16.77545036 |
| 7 | 0.74 | 0.576 | 4.792114752 | 23.96057376 |
| 8 | 0.651 | 0.487 | 4.086066538 | 20.43033269 |
| 9 | 0.565 | 0.401 | 3.409867802 | 17.04933901 |
| 10 | 0.661 | 0.497 | 4.165080218 | 20.82540109 |
| 11 | 0.6 | 0.436 | 3.684347392 | 18.42173696 |
| 12 | 0.564 | 0.4 | 3.40204 | 17.0102 |
| 13 | 0.541 | 0.377 | 3.222222458 | 16.11111229 |
| 14 | 0.671 | 0.507 | 4.244174298 | 21.22087149 |
| 15 | 0.61 | 0.446 | 3.762951032 | 18.81475516 |
| 16 | 0.552 | 0.388 | 3.308169088 | 16.54084544 |
| 17 | 0.568 | 0.404 | 3.433356032 | 17.16678016 |
| 18 | 0.606 | 0.442 | 3.731499928 | 18.65749964 |
| 19 | 0.539 | 0.375 | 3.20660625 | 16.03303125 |
| 20 | 0.543 | 0.379 | 3.237841882 | 16.18920941 |
| 21 | 0.549 | 0.385 | 3.28471945 | 16.42359725 |
| 22 | 0.568 | 0.404 | 3.433356032 | 17.16678016 |
| 23 | 0.573 | 0.409 | 3.472519162 | 17.36259581 |
| 24 | 0.582 | 0.418 | 3.543063448 | 17.71531724 |
| 1 | 0.242 | 0.078 | 0.923298168 | 4.61649084 |
| 2 | 0.244 | 0.08 | 0.9384368 | 4.692184 |
| 3 | 0.24 | 0.076 | 0.908162752 | 4.54081376 |
| 4 | 0.237 | 0.073 | 0.885465658 | 4.42732829 |
| 5 | 0.23 | 0.066 | 0.832533912 | 4.16266956 |
| 6 | 0.235 | 0.071 | 0.870338282 | 4.35169141 |
| 7 | 0.239 | 0.075 | 0.90059625 | 4.50298125 |
| 8 | 0.238 | 0.074 | 0.893030552 | 4.46515276 |
| 9 | 0.23 | 0.066 | 0.832533912 | 4.16266956 |

| | | | | |
|---|---|---|---|---|
| 10 | 0.285 | 0.121 | 1.249487482 | 6.24743741 |
| 11 | 0.273 | 0.109 | 1.158308362 | 5.79154181 |
| 12 | 0.28 | 0.116 | 1.211482112 | 6.05741056 |
| 13 | 0.254 | 0.09 | 1.0141782 | 5.070891 |
| 14 | 0.25 | 0.086 | 0.983871992 | 4.91935996 |
| 15 | 0.257 | 0.093 | 1.036916298 | 5.18458149 |
| 16 | 0.272 | 0.108 | 1.150715328 | 5.75357664 |
| 17 | 0.273 | 0.109 | 1.158308362 | 5.79154181 |
| 18 | 0.271 | 0.107 | 1.143123098 | 5.71561549 |
| 19 | 0.248 | 0.084 | 0.968723712 | 4.84361856 |
| 20 | 0.250 | 0.086 | 0.983871992 | 4.91935996 |
| 21 | 0.253 | 0.089 | 1.006600442 | 5.03300221 |
| 22 | 0.263 | 0.099 | 1.082414202 | 5.41207101 |
| 23 | 0.268 | 0.104 | 1.120351232 | 5.60175616 |
| 24 | 0.264 | 0.1 | 1.09 | 5.45 |
| 25 | 0.237 | 0.073 | 0.885465658 | 4.42732829 |
| 26 | 0.230 | 0.066 | 0.832533912 | 4.16266956 |
| 27 | 0.241 | 0.077 | 0.915730058 | 4.57865029 |
| 28 | 0.251 | 0.087 | 0.991447338 | 4.95723669 |
| 29 | 0.242 | 0.078 | 0.923298168 | 4.61649084 |
| 30 | 0.253 | 0.089 | 1.006600442 | 5.03300221 |
| 1 | 0.297 | 0.133 | 1.340782378 | 6.70391189 |
| 2 | 0.295 | 0.131 | 1.325558522 | 6.62779261 |
| 3 | 0.300 | 0.136 | 1.363624192 | 6.81812096 |
| 4 | 0.314 | 0.15 | 1.470315 | 7.351575 |
| 5 | 0.316 | 0.152 | 1.485569408 | 7.42784704 |
| 6 | 0.311 | 0.147 | 1.447439418 | 7.23719709 |
| 7 | 0.332 | 0.168 | 1.607720448 | 8.03860224 |
| 8 | 0.325 | 0.161 | 1.554254042 | 7.77127021 |
| 9 | 0.33 | 0.166 | 1.592440312 | 7.96220156 |
| 10 | 0.348 | 0.184 | 1.730077312 | 8.65038656 |
| 11 | 0.34 | 0.176 | 1.668873152 | 8.34436576 |
| 12 | 0.35 | 0.186 | 1.745386392 | 8.72693196 |
| 13 | 0.303 | 0.139 | 1.386473242 | 6.93236621 |
| 14 | 0.305 | 0.141 | 1.401709962 | 7.00854981 |
| 15 | 0.301 | 0.137 | 1.371239738 | 6.85619869 |
| 16 | 0.284 | 0.12 | 1.2418848 | 6.209424 |
| 17 | 0.289 | 0.125 | 1.27990625 | 6.39953125 |
| 18 | 0.280 | 0.116 | 1.211482112 | 6.05741056 |
| 19 | 0.275 | 0.111 | 1.173496842 | 5.86748421 |
| 20 | 0.278 | 0.114 | 1.196285592 | 5.98142796 |
| 21 | 0.269 | 0.105 | 1.12794105 | 5.63970525 |
| 22 | 0.324 | 0.16 | 1.5466192 | 7.733096 |
| 23 | 0.320 | 0.156 | 1.516087872 | 7.58043936 |
| 24 | 0.334 | 0.17 | 1.6230038 | 8.115019 |
| 25 | 0.363 | 0.199 | 1.844973802 | 9.22486901 |
| 26 | 0.367 | 0.203 | 1.875643418 | 9.37821709 |
| 27 | 0.372 | 0.208 | 1.913998528 | 9.56999264 |
| 28 | 0.380 | 0.216 | 1.975408512 | 9.87704256 |
| 29 | 0.374 | 0.21 | 1.9293462 | 9.646731 |
| 30 | 0.362 | 0.198 | 1.837308408 | 9.18654204 |

| | | | | | |
|---|---|---|---|---|---|
| 18.0683643 | 17.6639843 | 17.86617428 | 1 | 17.86617428 | 17.86617 |
| 18.3039099 | 17.8966986 | 18.10030423 | | 18.10030423 | 16.58116 |
| 18.186119 | 17.7803218 | 17.9832204 | | 17.9832204 | 23.72503 |
| 16.7754504 | 16.3868607 | 16.58115551 | 2 | 16.58115551 | 20.60707 |
| 16.3454519 | 15.9622077 | 16.15382978 | | 16.15382978 | 15.92096 |
| 16.7754504 | 16.3868607 | 16.58115551 | | 16.58115551 | 16.34801 |
| 23.9605738 | 23.4894946 | 23.72503416 | 3 | 23.72503416 | 15.84337 |
| 20.4303327 | 19.9981888 | 20.21426075 | | 20.21426075 | 16.97007 |
| 17.049339 | 16.657369 | 16.853354 | | 16.853354 | 4.507145 |
| 20.8254011 | 20.3887485 | 20.60707482 | 4 | 20.60707482 | 4.319462 |
| 18.421737 | 18.0131146 | 18.21742576 | | 18.21742576 | 4.394523 |
| 17.0102 | 16.6187119 | 16.81445593 | | 16.81445593 | 6.125537 |
| 16.1111123 | 15.7308011 | 15.92095668 | 5 | 15.92095668 | 4.95801 |
| 21.2208715 | 20.7797442 | 21.00030784 | | 21.00030784 | 5.635439 |
| 18.8147552 | 18.4014512 | 18.60810318 | | 18.60810318 | 4.732502 |
| 16.5408454 | 16.1551664 | 16.3480059 | 6 | 16.3480059 | 5.296555 |
| 17.1667802 | 16.7733665 | 16.97007334 | | 16.97007334 | 4.319462 |
| 18.6574996 | 18.2460642 | 18.45178194 | | 18.45178194 | 4.845237 |
| 16.0330313 | 15.6537004 | 15.84336583 | 7 | 15.84336583 | 6.578564 |
| 16.1892094 | 15.8079192 | 15.99856429 | | 15.99856429 | 7.221385 |
| 16.4235973 | 16.0393781 | 16.23148766 | | 16.23148766 | 7.903338 |
| 17.1667802 | 16.7733665 | 16.97007334 | 8 | 16.97007334 | 8.510658 |
| 17.3625958 | 16.9667829 | 17.16468937 | | 17.16468937 | 6.805303 |
| 17.7153172 | 17.3152071 | 17.51526215 | | 17.51526215 | 6.087812 |
| 4.61649084 | 4.39779853 | 4.507144684 | 9 | 4.507144684 | 5.748475 |
| 4.692184 | 4.47230994 | 4.582246971 | | 4.582246971 | 7.60008 |
| 4.54081376 | 4.32330455 | 4.432059155 | | 4.432059155 | 9.080995 |
| 4.42732829 | 4.21159628 | 4.319462283 | 10 | 4.319462283 | 9.728517 |
| 4.16266956 | 3.9510962 | 4.05688288 | | 4.05688288 | |
| 4.35169141 | 4.13714589 | 4.244418649 | | 4.244418649 | |
| 4.50298125 | 4.2860641 | 4.394522675 | 11 | 4.394522675 | |
| 4.46515276 | 4.24882801 | 4.356990384 | | 4.356990384 | |
| 4.16266956 | 3.9510962 | 4.05688288 | | 4.05688288 | |
| 6.24743741 | 6.00363639 | 6.125536899 | 12 | 6.125536899 | |
| 5.79154181 | 5.55468482 | 5.673113313 | | 5.673113313 | |
| 6.05741056 | 5.81649695 | 5.936953755 | | 5.936953755 | |
| 5.070891 | 4.84512855 | 4.958009776 | 13 | 4.958009776 | |
| 4.91935996 | 4.6959488 | 4.80765438 | | 4.80765438 | |
| 5.18458149 | 4.95705914 | 5.070820313 | | 5.070820313 | |
| 5.75357664 | 5.51730052 | 5.635438579 | 14 | 5.635438579 | |
| 5.79154181 | 5.55468482 | 5.673113313 | | 5.673113313 | |
| 5.71561549 | 5.47992058 | 5.597768035 | | 5.597768035 | |
| 4.84361856 | 4.62138508 | 4.732501819 | 15 | 4.732501819 | |
| 4.91935996 | 4.6959488 | 4.80765438 | | 4.80765438 | |
| 5.03300221 | 4.80782708 | 4.920414643 | | 4.920414643 | |
| 5.41207101 | 5.181038 | 5.296554503 | 16 | 5.296554503 | |
| 5.60175616 | 5.36780692 | 5.484781539 | | 5.484781539 | |
| 5.45 | 5.21838306 | 5.334191531 | | 5.334191531 | |
| 4.42732829 | 4.21159628 | 4.319462283 | 17 | 4.319462283 | |
| 4.16266956 | 3.9510962 | 4.05688288 | | 4.05688288 | |
| 4.57865029 | 4.36054936 | 4.469599825 | | 4.469599825 | |
| 4.95723669 | 4.7332372 | 4.845236945 | 18 | 4.845236945 | |

| | | | | |
|---|---|---|---|---|
| 4.61649084 | 4.39779853 | 4.507144684 | | 4.507144684 |
| 5.03300221 | 4.80782708 | 4.920414643 | | 4.920414643 |
| 6.70391189 | 6.45321566 | 6.578563773 | 19 | 6.578563773 |
| 6.62779261 | 6.37824219 | 6.503017399 | | 6.503017399 |
| 6.81812096 | 6.56570855 | 6.691914755 | | 6.691914755 |
| 7.351575 | 7.09119411 | 7.221384556 | 20 | 7.221384556 |
| 7.42784704 | 7.16633322 | 7.297090131 | | 7.297090131 |
| 7.23719709 | 6.97851814 | 7.107857615 | | 7.107857615 |
| 8.03860224 | 7.7680738 | 7.903338019 | 21 | 7.903338019 |
| 7.77127021 | 7.50467499 | 7.637972599 | | 7.637972599 |
| 7.96220156 | 7.6927952 | 7.82749838 | | 7.82749838 |
| 8.65038656 | 8.37093028 | 8.510658419 | 22 | 8.510658419 |
| 8.34436576 | 8.06936255 | 8.206864155 | | 8.206864155 |
| 8.72693196 | 8.4463658 | 8.58664888 | | 8.58664888 |
| 6.93236621 | 6.67824068 | 6.805303443 | 23 | 6.805303443 |
| 7.00854981 | 6.75328389 | 6.880916849 | | 6.880916849 |
| 6.85619869 | 6.6032149 | 6.729706795 | | 6.729706795 |
| 6.209424 | 5.96619978 | 6.087811891 | 24 | 6.087811891 |
| 6.39953125 | 6.1534264 | 6.276478825 | | 6.276478825 |
| 6.05741056 | 5.81649695 | 5.936953755 | | 5.936953755 |
| 5.86748421 | 5.62946649 | 5.748475349 | 25 | 5.748475349 |
| 5.98142796 | 5.74167169 | 5.861549824 | | 5.861549824 |
| 5.63970525 | 5.40517378 | 5.522439515 | | 5.522439515 |
| 7.733096 | 7.46706402 | 7.600080011 | 26 | 7.600080011 |
| 7.58043936 | 7.31666375 | 7.448551555 | | 7.448551555 |
| 8.115019 | 7.84336983 | 7.979194416 | | 7.979194416 |
| 9.22486901 | 8.9371217 | 9.080995353 | 27 | 9.080995353 |
| 9.37821709 | 9.08827172 | 9.233244403 | | 9.233244403 |
| 9.56999264 | 9.27730732 | 9.423649979 | | 9.423649979 |
| 9.87704256 | 9.57999095 | 9.728516755 | 28 | 9.728516755 |
| 9.646731 | 9.35295207 | 9.499841536 | | 9.499841536 |
| 9.18654204 | 8.89934509 | 9.042943564 | | 9.042943564 |

| | | | | | |
|---|---|---|---|---|---|
| 0.332 | 0.423 | 0.758 | 1.406 | 2.505 | 0.17 |
| 0.162 | 0.253 | 0.588 | 1.236 | 2.335 | |
| 1.25 | 2.5 | 5 | 10 | 20 | |

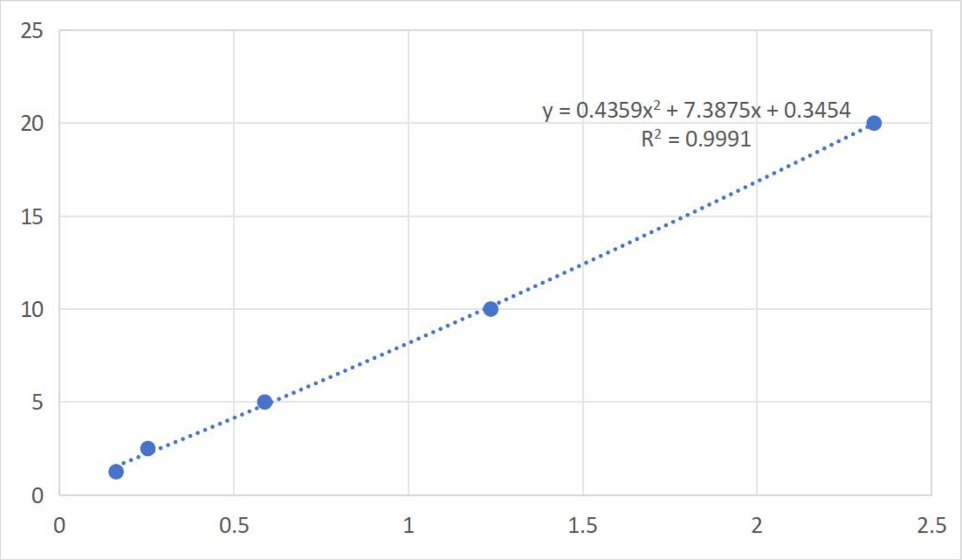


| Blank Corrected | Dilution Factor | Concentration |
|---|---|---|
| 0.421 | 3.532796852 | 17.66398426 |
| 0.427 | 3.579339711 | 17.89669856 |
| 0.424 | 3.556064358 | 17.78032179 |
| 0.388 | 3.27737213 | 16.38686065 |
| 0.377 | 3.192441531 | 15.96220766 |
| 0.388 | 3.27737213 | 16.38686065 |
| 0.57 | 4.69789891 | 23.48949455 |
| 0.481 | 3.99963776 | 19.9981888 |
| 0.395 | 3.331473798 | 16.65736899 |
| 0.491 | 4.077749708 | 20.38874854 |
| 0.43 | 3.60262291 | 18.01311455 |
| 0.394 | 3.323742372 | 16.61871186 |
| 0.371 | 3.146160212 | 15.73080106 |
| 0.501 | 4.155948836 | 20.77974418 |
| 0.44 | 3.68029024 | 18.4014512 |
| 0.382 | 3.231033272 | 16.15516636 |
| 0.398 | 3.354673304 | 16.77336652 |
| 0.436 | 3.649212846 | 18.24606423 |
| 0.369 | 3.13074008 | 15.6537004 |
| 0.373 | 3.161583831 | 15.80791916 |
| 0.379 | 3.207875612 | 16.03937806 |
| 0.398 | 3.354673304 | 16.77336652 |
| 0.403 | 3.393356583 | 16.96678292 |
| 0.412 | 3.46304141 | 17.31520705 |
| 0.072 | 0.879559706 | 4.397798528 |
| 0.074 | 0.894461988 | 4.472309942 |
| 0.07 | 0.86466091 | 4.32330455 |
| 0.067 | 0.842319255 | 4.211596276 |
| 0.06 | 0.79021924 | 3.9510962 |
| 0.065 | 0.827429178 | 4.137145888 |
| 0.069 | 0.85721282 | 4.2860641 |
| 0.068 | 0.849765602 | 4.248828008 |
| 0.06 | 0.79021924 | 3.9510962 |

| | | |
|---|---|---|
| 0.115 | 1.200727278 | 6.003636388 |
| 0.103 | 1.110936963 | 5.554684816 |
| 0.11 | 1.16329939 | 5.81649695 |
| 0.084 | 0.96902571 | 4.845128552 |
| 0.08 | 0.93918976 | 4.6959488 |
| 0.087 | 0.991411827 | 4.957059136 |
| 0.102 | 1.103460104 | 5.517300518 |
| 0.103 | 1.110936963 | 5.554684816 |
| 0.101 | 1.095984116 | 5.47992058 |
| 0.078 | 0.924277016 | 4.621385078 |
| 0.08 | 0.93918976 | 4.6959488 |
| 0.083 | 0.961565415 | 4.807827076 |
| 0.093 | 1.036207599 | 5.181037996 |
| 0.098 | 1.073561384 | 5.367806918 |
| 0.094 | 1.043676612 | 5.218383062 |
| 0.067 | 0.842319255 | 4.211596276 |
| 0.06 | 0.79021924 | 3.9510962 |
| 0.071 | 0.872109872 | 4.36054936 |
| 0.081 | 0.94664744 | 4.7332372 |
| 0.072 | 0.879559706 | 4.397798528 |
| 0.083 | 0.961565415 | 4.807827076 |
| 0.127 | 1.290643131 | 6.453215656 |
| 0.125 | 1.275648438 | 6.378242188 |
| 0.13 | 1.31314171 | 6.56570855 |
| 0.144 | 1.418238822 | 7.091194112 |
| 0.146 | 1.433266644 | 7.166333222 |
| 0.141 | 1.395703628 | 6.97851814 |
| 0.162 | 1.55361476 | 7.768073798 |
| 0.155 | 1.500934998 | 7.504674988 |
| 0.16 | 1.53855904 | 7.6927952 |
| 0.178 | 1.674186056 | 8.370930278 |
| 0.17 | 1.61387251 | 8.06936255 |
| 0.18 | 1.68927316 | 8.4463658 |
| 0.133 | 1.335648135 | 6.678240676 |
| 0.135 | 1.350656778 | 6.753283888 |
| 0.131 | 1.32064298 | 6.6032149 |
| 0.114 | 1.193239956 | 5.966199782 |
| 0.119 | 1.23068528 | 6.1534264 |
| 0.11 | 1.16329939 | 5.81649695 |
| 0.105 | 1.125893298 | 5.629466488 |
| 0.108 | 1.148334338 | 5.741671688 |
| 0.099 | 1.081034756 | 5.40517378 |
| 0.154 | 1.493412804 | 7.467064022 |
| 0.15 | 1.46333275 | 7.31666375 |
| 0.164 | 1.568673966 | 7.843369832 |
| 0.193 | 1.787424339 | 8.937121696 |
| 0.197 | 1.817654343 | 9.088271716 |
| 0.202 | 1.855461464 | 9.277307318 |
| 0.21 | 1.91599819 | 9.57999095 |
| 0.204 | 1.870590414 | 9.352952072 |
| 0.192 | 1.779869018 | 8.899345088 |

| | | | |
|---|---|---|---|
| 18.10030423 | 17.9832204 | 17.98323297 | 1 |
| 16.15382978 | 16.58115551 | 16.4387136 | 2 |
| 20.21426075 | 16.853354 | 20.2642163 | 3 |
| 18.21742576 | 16.81445593 | 18.54631884 | 4 |
| 21.00030784 | 18.60810318 | 18.50978923 | 5 |
| 16.97007334 | 18.45178194 | 17.25662039 | 6 |
| 15.99856429 | 16.23148766 | 16.02447259 | 7 |
| 17.16468937 | 17.51526215 | 17.21667495 | 8 |
| 4.582246971 | 4.432059155 | 4.50715027 | 9 |
| 4.05688288 | 4.244418649 | 4.206921271 | 10 |
| 4.356990384 | 4.05688288 | 4.269465313 | 11 |
| 5.673113313 | 5.936953755 | 5.911867989 | 12 |
| 4.80765438 | 5.070820313 | 4.945494823 | 13 |
| 5.673113313 | 5.597768035 | 5.635439976 | 14 |
| 4.80765438 | 4.920414643 | 4.820190281 | 15 |
| 5.484781539 | 5.334191531 | 5.371842524 | 16 |
| 4.05688288 | 4.469599825 | 4.281981663 | 17 |
| 4.507144684 | 4.920414643 | 4.757598757 | 18 |
| 6.503017399 | 6.691914755 | 6.591165309 | 19 |
| 7.297090131 | 7.107857615 | 7.208777434 | 20 |
| 7.637972599 | 7.82749838 | 7.789602999 | 21 |
| 8.206864155 | 8.58664888 | 8.434723818 | 22 |
| 6.880916849 | 6.729706795 | 6.805309029 | 23 |
| 6.276478825 | 5.936953755 | 6.100414824 | 24 |
| 5.861549824 | 5.522439515 | 5.710821563 | 25 |
| 7.448551555 | 7.979194416 | 7.675941994 | 26 |
| 9.233244403 | 9.423649979 | 9.245963245 | 27 |
| 9.499841536 | 9.042943564 | 9.423767285 | 28 |

Report
Tg

| Group | Mean | Std. Deviation |
|---|---|---|
| 1 | 17.780004 | 1.3524928398048 |
| 2 | 4.870795 | .5983107865421 |
| 3 | 7.498649 | 1.2601220764137 |
| Total | 9.49766 | 5.5548935069202 |

Tests of Normality

| | Group | Kolmogorov-Smirnova | | | Shapiro-Wilk | | |
|---|---|---|---|---|---|---|---|
| | | Statistic | df | Sig. | Statistic | df | Sig. |
| Tg | 1 | 0.16 | 8 | .200* | 0.956 | 8 | 0.775 |
| | 2 | 0.15 | 10 | .200* | 0.916 | 10 | 0.321 |
| | 3 | 0.117 | 10 | .200* | 0.958 | 10 | 0.76 |

* This is a lower bound of the true significance.
a Lilliefors Significance Correction

ANOVA
Tg

| | Sum of Sq | df | Mean Squa | F | Sig. |
|---|---|---|---|---|---|
| Between G | 802.817 | 2 | 401.409 | 331.003 | 0 |
| Within Gr | 30.318 | 25 | 1.213 | | |
| Total | 833.135 | 27 | | | |

Robust Tests of Equality of Means
Tg

| | Statistic | df1 | df2 | Sig. |
|---|---|---|---|---|
| Welch | 302.121 | 2 | 13.425 | 0 |

a Asymptotically F distributed.

Multiple Comparisons
Dependent Variable: Tg
Games-Howell

| (I) Group | (J) Group | Mean Diff | Std. Erro | Sig. | 95% Confidence Interval | |
|---|---|---|---|---|---|---|
| | | | | | Lower Bou | Upper Bound |
| 1 | 2 | 12.909209 | .51424915 | 0 | 11.478825 | 14.339593990300 |
| | 3 | 10.281356 | .62245110 | 0 | 8.660038 | 11.902673995852 |
| 2 | 1 | -12.9092 | 0.51424915 | 0 | -14.3396 | -11.4788 |
| | 3 | -2.627853 | .44112168 | 0 | -3.79416 | -1.46155 |
| 3 | 1 | -10.2813 | 5.62245110 | 0 | -11.9027 | -8.66004 |
| | 2 | 2.627853 | 4.44112168 | 0 | 1.461549 | 3.794158 |

* The mean difference is significant at the 0.05 level.

| | | | | | |
|---|---|---|---|---|---|
| 0.05 | 0.344 | 0.562 | 1.041 | 1.693 | 2.492 |
| 0 | 0.294 | 0.512 | 0.991 | 1.643 | 2.442 |
| 0 | 1 | 2 | 4 | 8 | 16 |

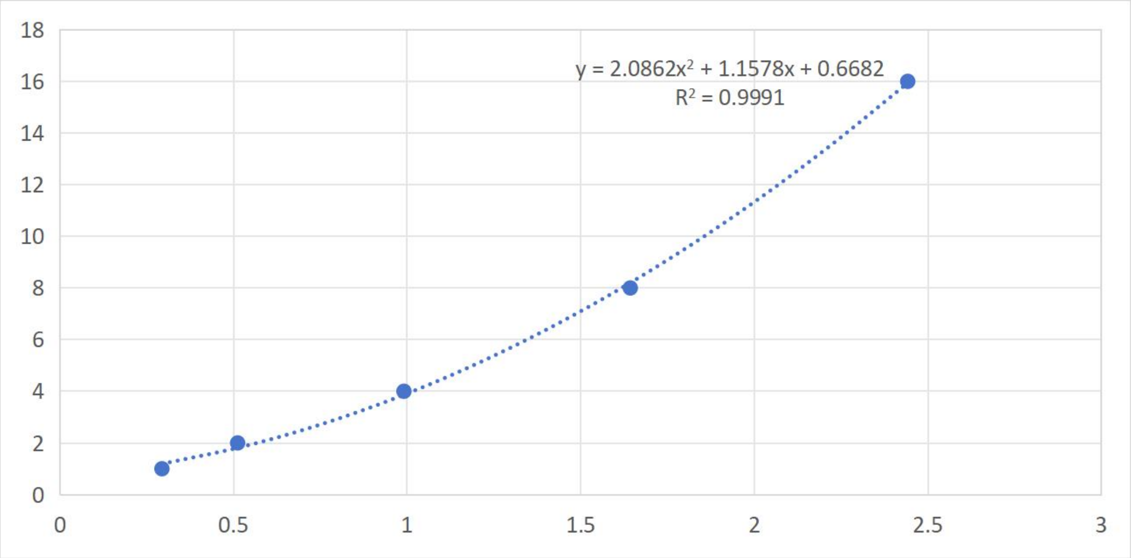


| Sample ID | OD （450n | Blank Cor | Dilution Fact | Concentration |
|---|---|---|---|---|
| 1 | 1.181 | 1.131 | 4.646257478 | 23.23128739 |
| 2 | 1.22 | 1.17 | 4.87862518 | 24.3931259 |
| 3 | 1.144 | 1.094 | 4.431672463 | 22.15836232 |
| 4 | 1.453 | 1.403 | 6.399088256 | 31.99544128 |
| 5 | 1.328 | 1.278 | 5.555225481 | 27.7761274 |
| 6 | 1.255 | 1.205 | 5.092563555 | 25.46281778 |
| 7 | 1.324 | 1.274 | 5.529298351 | 27.64649176 |
| 8 | 1.274 | 1.224 | 5.210841971 | 26.05420986 |
| 9 | 1.211 | 1.161 | 4.82443859 | 24.12219295 |
| 10 | 1.219 | 1.169 | 4.872587758 | 24.36293879 |
| 11 | 1.082 | 1.032 | 4.084902669 | 20.42451334 |
| 12 | 1.311 | 1.261 | 5.44549623 | 27.22748115 |
| 13 | 0.733 | 0.683 | 2.432166752 | 12.16083376 |
| 14 | 0.761 | 0.711 | 2.54601371 | 12.73006855 |
| 15 | 0.721 | 0.671 | 2.384376574 | 11.92188287 |
| 16 | 0.709 | 0.659 | 2.337187222 | 11.68593611 |
| 17 | 0.695 | 0.645 | 2.282892355 | 11.41446178 |
| 18 | 0.701 | 0.651 | 2.306061446 | 11.53030723 |
| 19 | 0.672 | 0.622 | 2.195469001 | 10.977345 |
| 20 | 0.683 | 0.633 | 2.237004792 | 11.18502396 |
| 21 | 0.671 | 0.621 | 2.191718054 | 10.95859027 |
| 22 | 0.643 | 0.593 | 2.088385544 | 10.44192772 |
| 23 | 0.692 | 0.642 | 2.271364137 | 11.35682068 |
| 24 | 0.727 | 0.677 | 2.40819656 | 12.0409828 |
| 25 | 0.699 | 0.649 | 2.298321726 | 11.49160863 |
| 26 | 0.679 | 0.629 | 2.221842454 | 11.10921227 |
| 27 | 0.705 | 0.655 | 2.321590955 | 11.60795478 |
| 28 | 0.662 | 0.612 | 2.158147293 | 10.79073646 |
| 29 | 0.687 | 0.637 | 2.252233888 | 11.26116944 |
| 30 | 0.717 | 0.667 | 2.368580032 | 11.84290016 |

| | | | | |
|---|---|---|---|---|
| 31 | 0.656 | 0.606 | 2.135954543 | 10.67977272 |
| 32 | 0.788 | 0.738 | 2.658892713 | 13.29446356 |
| 33 | 0.712 | 0.662 | 2.348928233 | 11.74464116 |
| 34 | 0.744 | 0.694 | 2.476502223 | 12.38251112 |
| 35 | 0.785 | 0.735 | 2.646200395 | 13.23100198 |
| 36 | 0.788 | 0.738 | 2.658892713 | 13.29446356 |
| 37 | 0.803 | 0.753 | 2.722917576 | 13.61458788 |
| 38 | 0.731 | 0.681 | 2.424159998 | 12.12079999 |
| 39 | 0.720 | 0.67 | 2.38042118 | 11.9021059 |
| 40 | 0.752 | 0.702 | 2.509063305 | 12.54531652 |
| 41 | 0.722 | 0.672 | 2.388336141 | 11.9416807 |
| 42 | 0.763 | 0.713 | 2.554270808 | 12.77135404 |
| 43 | 0.761 | 0.711 | 2.54601371 | 12.73006855 |
| 44 | 0.683 | 0.633 | 2.237004792 | 11.18502396 |
| 45 | 0.702 | 0.652 | 2.309937565 | 11.54968782 |
| 46 | 0.777 | 0.727 | 2.6125378 | 13.062689 |

| | | | | | |
|---|---|---|---|---|---|
| 0.048 | 0.329 | 0.573 | 1.058 | 1.712 | 2.488 |
| 0 | 0.281 | 0.525 | 1.01 | 1.664 | 2.44 |
| 0 | 1 | 2 | 4 | 8 | 16 |

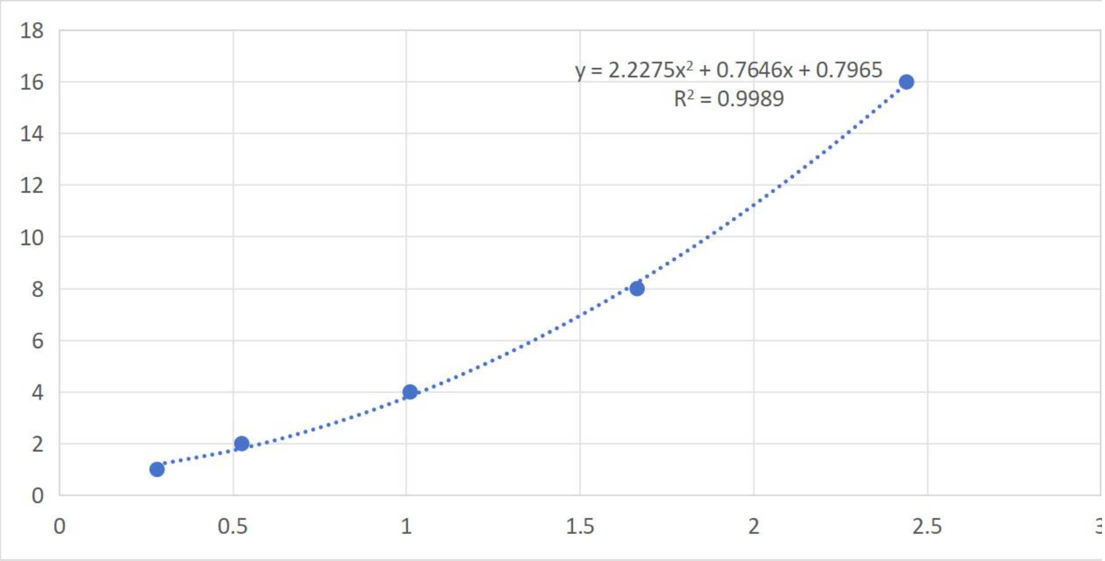


| Sample ID | OD (450n | Blank Cor | Dilution Fact | Concentration | |
|---|---|---|---|---|---|
| 1 | 1.181 | 1.133 | 4.522209048 | 22.61104524 | 23.23129 |
| 2 | 1.22 | 1.172 | 4.75226956 | 23.7613478 | 24.39313 |
| 3 | 1.144 | 1.096 | 4.31021024 | 21.5510512 | 22.15836 |
| 4 | 1.453 | 1.405 | 6.267903688 | 31.33951844 | 31.99544 |
| 5 | 1.328 | 1.28 | 5.424724 | 27.12362 | 27.77613 |
| 6 | 1.255 | 1.207 | 4.964503348 | 24.82251674 | 25.46282 |
| 7 | 1.324 | 1.276 | 5.39889164 | 26.9944582 | 27.64649 |
| 8 | 1.274 | 1.226 | 5.08200139 | 25.41000695 | 26.05421 |
| 9 | 1.211 | 1.163 | 4.698577248 | 23.49288624 | 24.12219 |
| 10 | 1.219 | 1.171 | 4.746285928 | 23.73142964 | 24.36294 |
| 11 | 1.082 | 1.034 | 3.96864139 | 19.84320695 | 20.42451 |
| 12 | 1.311 | 1.263 | 5.315428748 | 26.57714374 | 27.22748 |
| 13 | 0.733 | 0.685 | 2.365449688 | 11.82724844 | 12.16083 |
| 14 | 0.761 | 0.713 | 2.474051748 | 12.37025874 | 12.73007 |
| 15 | 0.721 | 0.673 | 2.319975148 | 11.59987574 | 11.92188 |
| 16 | 0.709 | 0.661 | 2.275142128 | 11.37571064 | 11.68594 |
| 17 | 0.695 | 0.647 | 2.223647748 | 11.11823874 | 11.41446 |
| 18 | 0.701 | 0.653 | 2.245609848 | 11.22804924 | 11.53031 |
| 19 | 0.672 | 0.624 | 2.14094544 | 10.7047272 | 10.97735 |
| 20 | 0.683 | 0.635 | 2.180204688 | 10.90102344 | 11.18502 |
| 21 | 0.671 | 0.623 | 2.137403148 | 10.68701574 | 10.95859 |
| 22 | 0.643 | 0.595 | 2.040027688 | 10.20013844 | 10.44193 |
| 23 | 0.692 | 0.644 | 2.21272684 | 11.0636342 | 11.35682 |
| 24 | 0.727 | 0.679 | 2.342632228 | 11.71316114 | 12.04098 |
| 25 | 0.699 | 0.651 | 2.238271328 | 11.19135664 | 11.49161 |
| 26 | 0.679 | 0.631 | 2.165866228 | 10.82933114 | 11.10921 |
| 27 | 0.705 | 0.657 | 2.260340348 | 11.30170174 | 11.60795 |
| 28 | 0.662 | 0.614 | 2.10572299 | 10.52861495 | 10.79074 |
| 29 | 0.687 | 0.639 | 2.194614428 | 10.97307214 | 11.26117 |
| 30 | 0.717 | 0.669 | 2.304959528 | 11.52479764 | 11.8429 |

| | | | | | |
|---|---|---|---|---|---|
| 31 | 0.656 | 0.608 | 2.08480336 | 10.4240168 | 10.67977 |
| 32 | 0.788 | 0.74 | 2.582083 | 12.910415 | 13.29446 |
| 33 | 0.712 | 0.664 | 2.28629024 | 11.4314512 | 11.74464 |
| 34 | 0.744 | 0.696 | 2.40769824 | 12.0384912 | 12.38251 |
| 35 | 0.785 | 0.737 | 2.569919148 | 12.84959574 | 13.231 |
| 36 | 0.788 | 0.74 | 2.582083 | 12.910415 | 13.29446 |
| 37 | 0.803 | 0.755 | 2.643503688 | 13.21751844 | 13.61459 |
| 38 | 0.731 | 0.683 | 2.357826048 | 11.78913024 | 12.1208 |
| 39 | 0.720 | 0.672 | 2.31621456 | 11.5810728 | 11.90211 |
| 40 | 0.752 | 0.704 | 2.43876304 | 12.1938152 | 12.54532 |
| 41 | 0.722 | 0.674 | 2.32374019 | 11.61870095 | 11.94168 |
| 42 | 0.763 | 0.715 | 2.481942688 | 12.40971344 | 12.77135 |
| 43 | 0.761 | 0.713 | 2.474051748 | 12.37025874 | 12.73007 |
| 44 | 0.683 | 0.635 | 2.180204688 | 10.90102344 | 11.18502 |
| 45 | 0.702 | 0.654 | 2.24928579 | 11.24642895 | 11.54969 |
| 46 | 0.777 | 0.729 | 2.537678228 | 12.68839114 | 13.06269 |

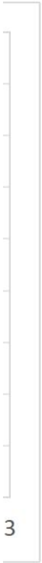


| | |
|---|---|
| 22.61105 | 22.92116632 |
| 23.76135 | 24.07723685 |
| 21.55105 | 21.85470676 |
| 31.33952 | 31.66747986 |
| 27.12362 | 27.4498737 |
| 24.82252 | 25.14266726 |
| 26.99446 | 27.32047498 |
| 25.41001 | 25.73210841 |
| 23.49289 | 23.8075396 |
| 23.73143 | 24.04718422 |
| 19.84321 | 20.13386015 |
| 26.57714 | 26.90231245 |
| 11.82725 | 11.9940411 |
| 12.37026 | 12.55016365 |
| 11.59988 | 11.76087931 |
| 11.37571 | 11.53082338 |
| 11.11824 | 11.26635026 |
| 11.22805 | 11.37917824 |
| 10.70473 | 10.8410361 |
| 10.90102 | 11.0430237 |
| 10.68702 | 10.82280301 |
| 10.20014 | 10.32103308 |
| 11.06363 | 11.21022744 |
| 11.71316 | 11.87707197 |
| 11.19136 | 11.34148264 |
| 10.82933 | 10.96927171 |
| 11.3017 | 11.45482826 |
| 10.52861 | 10.65967571 |
| 10.97307 | 11.11712079 |
| 11.5248 | 11.6838489 |

| | |
|---|---|
| 10.42402 | 10.55189476 |
| 12.91042 | 13.10243928 |
| 11.43145 | 11.58804618 |
| 12.03849 | 12.21050116 |
| 12.8496 | 13.04029886 |
| 12.91042 | 13.10243928 |
| 13.21752 | 13.41605316 |
| 11.78913 | 11.95496512 |
| 11.58107 | 11.74158935 |
| 12.19382 | 12.36956586 |
| 11.6187 | 11.78019083 |
| 12.40971 | 12.59053374 |
| 12.37026 | 12.55016365 |
| 10.90102 | 11.0430237 |
| 11.24643 | 11.39805839 |
| 12.68839 | 12.87554007 |

| | | | | | | | | |
|---|---|---|---|---|---|---|---|---|
| 0.025 | 0.338 | 0.546 | 1.025 | 1.554 | 2.509 | 0.033 | 0.362 | 0.603 |
| 0 | 0.313 | 0.521 | 1 | 1.529 | 2.484 | 0 | 0.329 | 0.57 |
| 0 | 1 | 2 | 4 | 8 | 16 | 0 | 1 | 2 |

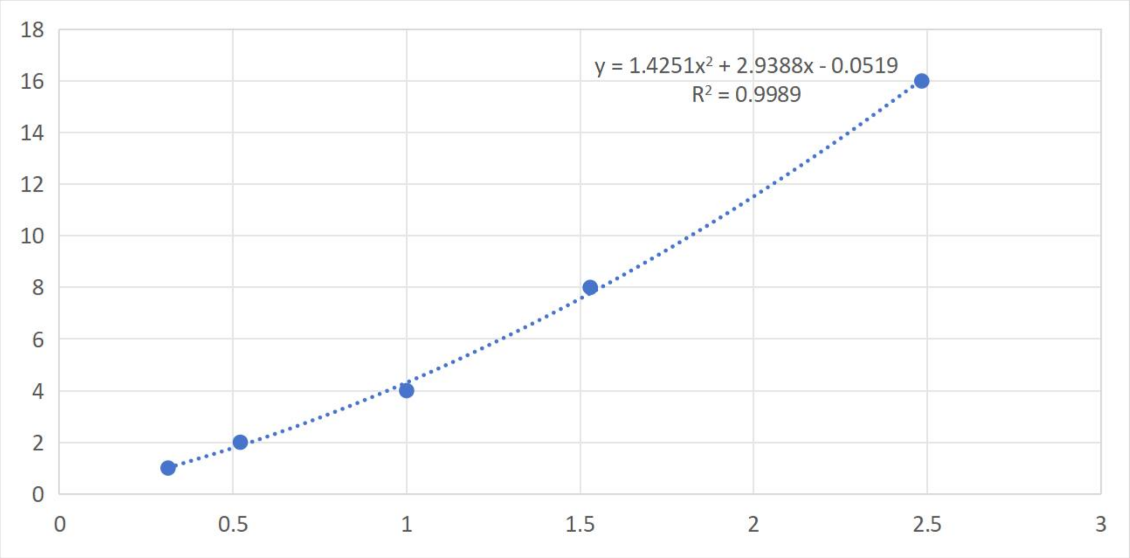


| Sample ID | OD（450n | Blank Cor | Dilution Fact | Concentration |
|---|---|---|---|---|
| 1 | 1.218 | 1.193 | 5.48236055 | 27.41180275 |
| 2 | 1.112 | 1.087 | 4.826429582 | 24.13214791 |
| 3 | 1.162 | 1.137 | 5.131840702 | 25.65920351 |
| 4 | 1.335 | 1.31 | 6.24354211 | 31.21771055 |
| 5 | 1.282 | 1.257 | 5.89389943 | 29.46949715 |
| 6 | 1.364 | 1.339 | 6.438244917 | 32.19122459 |
| 7 | 1.122 | 1.097 | 4.886941766 | 24.43470883 |
| 8 | 1.274 | 1.249 | 5.841818625 | 29.20909313 |
| 9 | 1.098 | 1.073 | 4.742191358 | 23.71095679 |
| 10 | 1.108 | 1.083 | 4.802304514 | 24.01152257 |
| 11 | 1.032 | 1.007 | 4.35259283 | 21.76296415 |
| 12 | 1.267 | 1.242 | 5.796397556 | 28.98198778 |
| 13 | 0.821 | 0.796 | 3.190350962 | 15.95175481 |
| 14 | 0.688 | 0.663 | 2.522954182 | 12.61477091 |
| 15 | 0.702 | 0.677 | 2.590832258 | 12.95416129 |
| 16 | 0.692 | 0.667 | 2.542290914 | 12.71145457 |
| 17 | 0.712 | 0.687 | 2.639658622 | 13.19829311 |
| 18 | 0.679 | 0.654 | 2.479613272 | 12.39806636 |
| 19 | 0.783 | 0.758 | 2.994521556 | 14.97260778 |
| 20 | 0.674 | 0.649 | 2.455634745 | 12.27817373 |
| 21 | 0.663 | 0.638 | 2.403132804 | 12.01566402 |
| 22 | 0.705 | 0.68 | 2.60545024 | 13.0272512 |
| 23 | 0.677 | 0.652 | 2.47001331 | 12.35006655 |
| 24 | 0.696 | 0.671 | 2.561673249 | 12.80836625 |
| 25 | 0.648 | 0.623 | 2.332095038 | 11.66047519 |
| 26 | 0.688 | 0.663 | 2.522954182 | 12.61477091 |
| 27 | 0.711 | 0.686 | 2.63476316 | 13.1738158 |
| 28 | 0.680 | 0.655 | 2.484417528 | 12.42208764 |
| 29 | 0.655 | 0.63 | 2.36516619 | 11.82583095 |
| 30 | 0.702 | 0.677 | 2.590832258 | 12.95416129 |
| 31 | 0.677 | 0.652 | 2.47001331 | 12.35006655 |
| 32 | 0.802 | 0.777 | 3.091921798 | 15.45960899 |

| | | | | |
|---|---|---|---|---|
| 33 | 0.692 | 0.667 | 2.542290914 | 12.71145457 |
| 34 | 0.752 | 0.727 | 2.837814278 | 14.18907139 |
| 35 | 0.794 | 0.769 | 3.050785761 | 15.25392881 |
| 36 | 0.763 | 0.738 | 2.893106564 | 14.46553282 |
| 37 | 0.811 | 0.786 | 3.13841788 | 15.6920894 |
| 38 | 0.740 | 0.715 | 2.777888748 | 13.88944374 |
| 39 | 0.771 | 0.746 | 2.933535752 | 14.66767876 |
| 40 | 0.733 | 0.708 | 2.743121726 | 13.71560863 |
| 41 | 0.747 | 0.722 | 2.812795428 | 14.06397714 |
| 42 | 0.756 | 0.731 | 2.857880661 | 14.28940331 |
| 43 | 0.749 | 0.724 | 2.822794418 | 14.11397209 |
| 44 | 0.723 | 0.698 | 2.69369682 | 13.4684841 |
| 45 | 0.698 | 0.673 | 2.571381518 | 12.85690759 |
| 46 | 0.757 | 0.732 | 2.862904382 | 14.31452191 |

| | | |
|---|---|---|
| 1.044 | 1.623 | 2.492 |
| 1.011 | 1.59 | 2.459 |
| 4 | 8 | 16 |

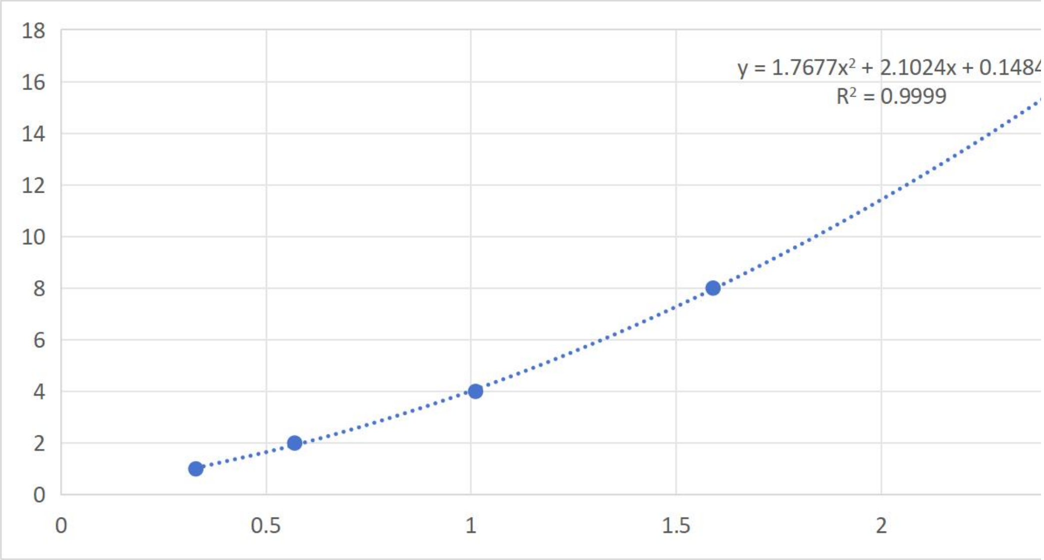


| Sample ID | OD (450n | Blank Correct | Dilution Fact | Concentration | |
|---|---|---|---|---|---|
| 1 | 1.218 | 1.185 | 5.121992533 | 25.60996266 | 27.4118 |
| 2 | 1.112 | 1.079 | 4.474918416 | 22.37459208 | 24.13215 |
| 3 | 1.162 | 1.129 | 4.775192496 | 23.87596248 | 25.6592 |
| 4 | 1.335 | 1.302 | 5.882336911 | 29.41168455 | 31.21771 |
| 5 | 1.282 | 1.249 | 5.531911368 | 27.65955684 | 29.4695 |
| 6 | 1.364 | 1.331 | 6.07828278 | 30.3914139 | 32.19122 |
| 7 | 1.122 | 1.089 | 4.534266152 | 22.67133076 | 24.43471 |
| 8 | 1.274 | 1.241 | 5.479879584 | 27.39939792 | 29.20909 |
| 9 | 1.098 | 1.065 | 4.392425533 | 21.96212766 | 23.71096 |
| 10 | 1.108 | 1.075 | 4.451278313 | 22.25639156 | 24.01152 |
| 11 | 1.032 | 0.999 | 4.012863968 | 20.06431984 | 21.76296 |
| 12 | 1.267 | 1.234 | 5.434537381 | 27.17268691 | 28.98199 |
| 13 | 0.821 | 0.788 | 2.902733909 | 14.51366954 | 15.95175 |
| 14 | 0.688 | 0.655 | 2.283859493 | 11.41929746 | 12.61477 |
| 15 | 0.702 | 0.669 | 2.34605918 | 11.7302959 | 12.95416 |
| 16 | 0.692 | 0.659 | 2.301560124 | 11.50780062 | 12.71145 |
| 17 | 0.712 | 0.679 | 2.390911776 | 11.95455888 | 13.19829 |
| 18 | 0.679 | 0.646 | 2.244239893 | 11.22119947 | 12.39807 |
| 19 | 0.783 | 0.75 | 2.71953125 | 13.59765625 | 14.97261 |
| 20 | 0.674 | 0.641 | 2.222352744 | 11.11176372 | 12.27817 |
| 21 | 0.663 | 0.63 | 2.17451213 | 10.87256065 | 12.01566 |
| 22 | 0.705 | 0.672 | 2.359477837 | 11.79738918 | 13.02725 |
| 23 | 0.677 | 0.644 | 2.235474427 | 11.17737214 | 12.35007 |
| 24 | 0.696 | 0.663 | 2.319317321 | 11.59658661 | 12.80837 |
| 25 | 0.648 | 0.615 | 2.109964333 | 10.54982166 | 11.66048 |
| 26 | 0.688 | 0.655 | 2.283859493 | 11.41929746 | 12.61477 |
| 27 | 0.711 | 0.678 | 2.386410607 | 11.93205303 | 13.17382 |
| 28 | 0.680 | 0.647 | 2.248627929 | 11.24313965 | 12.42209 |
| 29 | 0.655 | 0.622 | 2.139987647 | 10.69993823 | 11.82583 |
| 30 | 0.702 | 0.669 | 2.34605918 | 11.7302959 | 12.95416 |
| 31 | 0.677 | 0.644 | 2.235474427 | 11.17737214 | 12.35007 |
| 32 | 0.802 | 0.769 | 2.81049444 | 14.0524722 | 15.45961 |

| | | | | | |
|---|---|---|---|---|---|
| 33 | 0.692 | 0.659 | 2.301560124 | 11.50780062 | 12.71145 |
| 34 | 0.752 | 0.719 | 2.57385756 | 12.8692878 | 14.18907 |
| 35 | 0.794 | 0.761 | 2.772038592 | 13.86019296 | 15.25393 |
| 36 | 0.763 | 0.73 | 2.62515933 | 13.12579665 | 14.46553 |
| 37 | 0.811 | 0.778 | 2.854027727 | 14.27013863 | 15.69209 |
| 38 | 0.740 | 0.707 | 2.518379877 | 12.59189939 | 13.88944 |
| 39 | 0.771 | 0.738 | 2.662738399 | 13.31369199 | 14.66768 |
| 40 | 0.733 | 0.7 | 2.486253 | 12.431265 | 13.71561 |
| 41 | 0.747 | 0.714 | 2.550679989 | 12.75339995 | 14.06398 |
| 42 | 0.756 | 0.723 | 2.592463253 | 12.96231627 | 14.2894 |
| 43 | 0.749 | 0.716 | 2.559940411 | 12.79970206 | 14.11397 |
| 44 | 0.723 | 0.69 | 2.44065797 | 12.20328985 | 13.46848 |
| 45 | 0.698 | 0.665 | 2.328217133 | 11.64108566 | 12.85691 |
| 46 | 0.757 | 0.724 | 2.597123515 | 12.98561758 | 14.31452 |

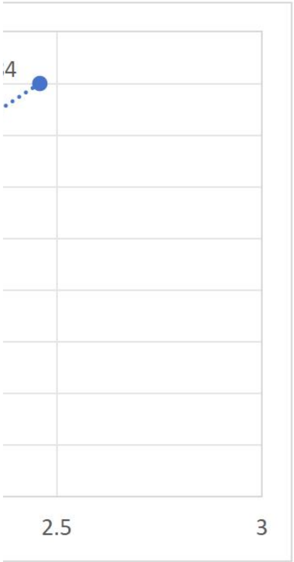


| | |
|---|---|
| 25.60996 | 26.51088271 |
| 22.37459 | 23.25337 |
| 23.87596 | 24.767583 |
| 29.41168 | 30.31469755 |
| 27.65956 | 28.564527 |
| 30.39141 | 31.29131925 |
| 22.67133 | 23.5530198 |
| 27.3994 | 28.30424553 |
| 21.96213 | 22.83654223 |
| 22.25639 | 23.13395707 |
| 20.06432 | 20.913642 |
| 27.17269 | 28.07733735 |
| 14.51367 | 15.23271218 |
| 11.4193 | 12.01703419 |
| 11.7303 | 12.3422286 |
| 11.5078 | 12.1096276 |
| 11.95456 | 12.576426 |
| 11.2212 | 11.80963292 |
| 13.59766 | 14.28513202 |
| 11.11176 | 11.69496873 |
| 10.87256 | 11.44411234 |
| 11.79739 | 12.41232019 |
| 11.17737 | 11.76371935 |
| 11.59659 | 12.20247643 |
| 10.54982 | 11.10514843 |
| 11.4193 | 12.01703419 |
| 11.93205 | 12.55293442 |
| 11.24314 | 11.83261365 |
| 10.69994 | 11.26288459 |
| 11.7303 | 12.3422286 |
| 11.17737 | 11.76371935 |
| 14.05247 | 14.7560406 |

| | |
|---|---|
| 11.5078 | 12.1096276 |
| 12.86929 | 13.5291796 |
| 13.86019 | 14.55706089 |
| 13.1258 | 13.79566474 |
| 14.27014 | 14.98111402 |
| 12.5919 | 13.24067157 |
| 13.31369 | 13.99068538 |
| 12.43127 | 13.07343682 |
| 12.7534 | 13.40868855 |
| 12.96232 | 13.62585979 |
| 12.7997 | 13.45683708 |
| 12.20329 | 12.83588698 |
| 11.64109 | 12.24899663 |
| 12.98562 | 13.65006975 |

| | | | | | |
|---|---|---|---|---|---|
| 0.047 | 0.326 | 0.557 | 1.039 | 1.597 | 2.496 |
| 0 | 0.279 | 0.51 | 0.992 | 1.55 | 2.449 |
| 0 | 1 | 2 | 4 | 8 | 16 |

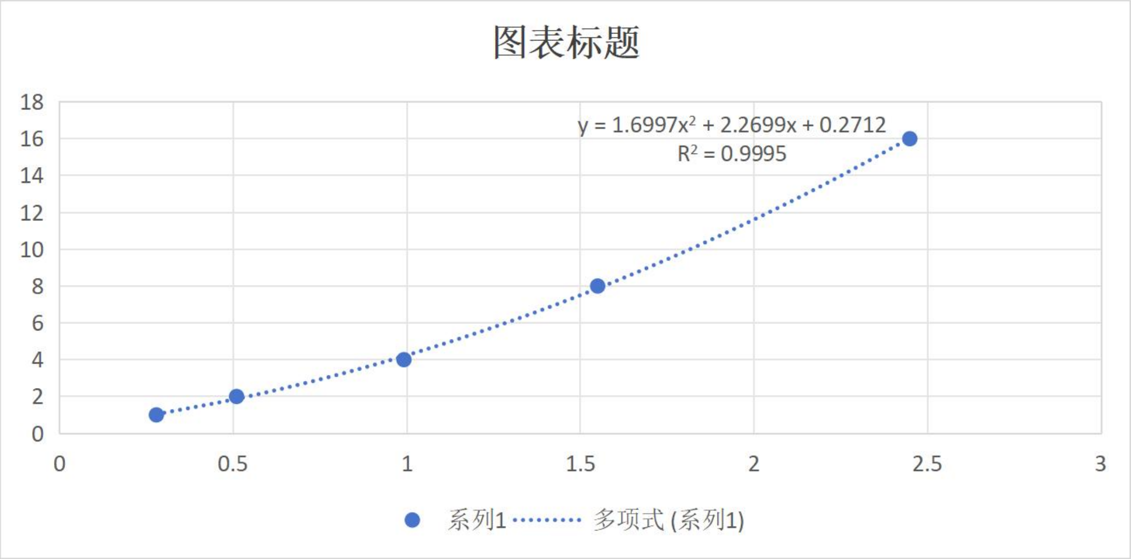


| | | | | |
|---|---|---|---|---|
| 1 | 1.171 | 1.124 | 4.969927787 | 24.84963894 |
| 2 | 1.204 | 1.157 | 5.172776005 | 25.86388003 |
| 3 | 1.255 | 1.208 | 5.493550221 | 27.4677511 |
| 4 | 1.422 | 1.375 | 6.605807813 | 33.02903906 |
| 5 | 1.230 | 1.183 | 5.335203153 | 26.67601577 |
| 6 | 1.411 | 1.364 | 6.529628651 | 32.64814326 |
| 7 | 1.209 | 1.162 | 5.203833527 | 26.01916763 |
| 8 | 1.361 | 1.314 | 6.188543821 | 30.94271911 |
| 9 | 1.076 | 1.029 | 4.406639148 | 22.03319574 |
| 10 | 1.125 | 1.078 | 4.693346375 | 23.46673187 |
| 11 | 1.129 | 1.082 | 4.717111383 | 23.58555691 |
| 12 | 1.284 | 1.237 | 5.679894549 | 28.39947275 |
| 13 | 0.797 | 0.75 | 2.92970625 | 14.64853125 |
| 14 | 0.677 | 0.63 | 2.37584793 | 11.87923965 |
| 15 | 0.698 | 0.651 | 2.46923946 | 12.3461973 |
| 16 | 0.701 | 0.654 | 2.482703485 | 12.41351743 |
| 17 | 0.680 | 0.633 | 2.389097793 | 11.94548897 |
| 18 | 0.652 | 0.605 | 2.266622193 | 11.33311096 |
| 19 | 0.709 | 0.662 | 2.518757127 | 12.59378563 |
| 20 | 0.684 | 0.637 | 2.406811869 | 12.03405935 |
| 21 | 0.683 | 0.636 | 2.402378251 | 12.01189126 |
| 22 | 0.699 | 0.652 | 2.473724069 | 12.36862034 |
| 23 | 0.684 | 0.637 | 2.406811869 | 12.03405935 |
| 24 | 0.673 | 0.626 | 2.358229037 | 11.79114519 |
| 25 | 0.714 | 0.667 | 2.541401133 | 12.70700567 |
| 26 | 0.661 | 0.614 | 2.305698701 | 11.52849351 |
| 27 | 0.693 | 0.646 | 2.446867405 | 12.23433703 |
| 28 | 0.659 | 0.612 | 2.296991237 | 11.48495618 |
| 29 | 0.643 | 0.596 | 2.227821035 | 11.13910518 |
| 30 | 0.691 | 0.644 | 2.437942379 | 12.1897119 |
| 31 | 0.694 | 0.647 | 2.451335017 | 12.25667509 |
| 32 | 0.792 | 0.745 | 2.905651493 | 14.52825746 |
| 33 | 0.715 | 0.668 | 2.545940133 | 12.72970066 |

| | | | | |
|---|---|---|---|---|
| 34 | 0.741 | 0.694 | 2.665147309 | 13.32573655 |
| 35 | 0.788 | 0.741 | 2.886468876 | 14.43234438 |
| 36 | 0.771 | 0.724 | 2.805549547 | 14.02774774 |
| 37 | 0.802 | 0.755 | 2.953845993 | 14.76922996 |
| 38 | 0.813 | 0.766 | 3.007252573 | 15.03626287 |
| 39 | 0.808 | 0.761 | 2.982925864 | 14.91462932 |
| 40 | 0.757 | 0.71 | 2.73964777 | 13.69823885 |
| 41 | 0.763 | 0.716 | 2.767809803 | 13.83904902 |
| 42 | 0.782 | 0.735 | 2.857796933 | 14.28898466 |
| 43 | 0.804 | 0.757 | 2.963525685 | 14.81762843 |
| 44 | 0.732 | 0.685 | 2.623623233 | 13.11811616 |
| 45 | 0.765 | 0.718 | 2.777224343 | 13.88612171 |
| 46 | 0.744 | 0.697 | 2.679049857 | 13.39524929 |

| | | | | | |
|---|---|---|---|---|---|
| 0.04 | 0.351 | 0.585 | 1.029 | 1.611 | 2.503 |
| 0 | 0.311 | 0.545 | 0.989 | 1.571 | 2.463 |
| 0 | 1 | 2 | 4 | 8 | 16 |

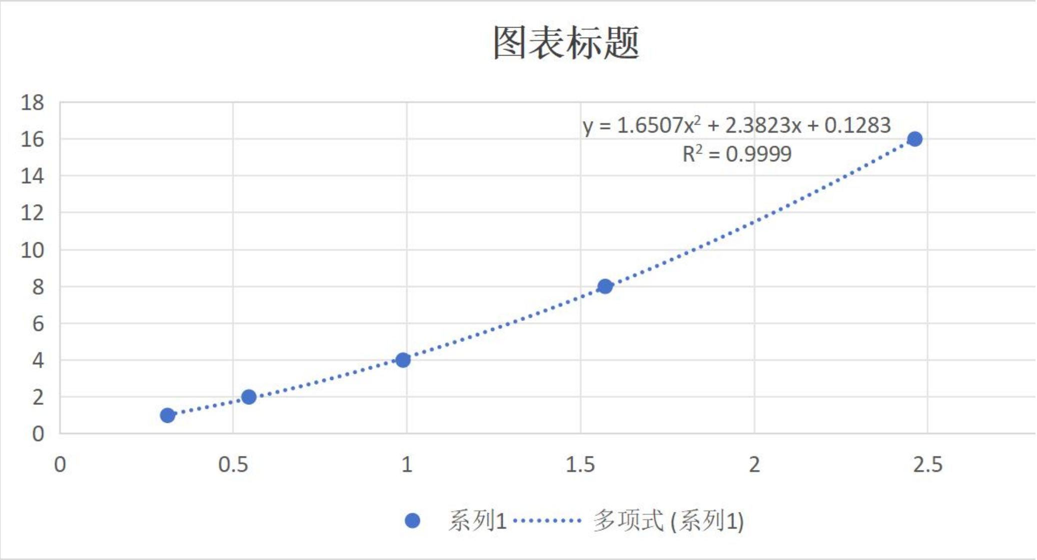


| | | | | | |
|---|---|---|---|---|---|
| 1 | 1.171 | 1.131 | 4.934192363 | 24.67096181 | 24.84964 |
| 2 | 1.204 | 1.164 | 5.137824027 | 25.68912014 | 25.86388 |
| 3 | 1.255 | 1.215 | 5.459599108 | 27.29799554 | 27.46775 |
| 4 | 1.422 | 1.382 | 6.573350147 | 32.86675073 | 33.02904 |
| 5 | 1.230 | 1.19 | 5.30079327 | 26.50396635 | 26.67602 |
| 6 | 1.411 | 1.371 | 6.497156699 | 32.48578349 | 32.64814 |
| 7 | 1.209 | 1.169 | 5.168990943 | 25.84495471 | 26.01917 |
| 8 | 1.361 | 1.321 | 6.155857479 | 30.77928739 | 30.94272 |
| 9 | 1.076 | 1.036 | 4.368052507 | 21.84026254 | 22.0332 |
| 10 | 1.125 | 1.085 | 4.656340808 | 23.28170404 | 23.46673 |
| 11 | 1.129 | 1.089 | 4.680224495 | 23.40112247 | 23.58556 |
| 12 | 1.284 | 1.244 | 5.646398875 | 28.23199438 | 28.39947 |
| 13 | 0.797 | 0.757 | 2.877633084 | 14.38816542 | 14.64853 |
| 14 | 0.677 | 0.637 | 2.315627988 | 11.57813994 | 11.87924 |
| 15 | 0.698 | 0.658 | 2.410547075 | 12.05273537 | 12.3462 |
| 16 | 0.701 | 0.661 | 2.424225795 | 12.12112897 | 12.41352 |
| 17 | 0.680 | 0.64 | 2.32909872 | 11.6454936 | 11.94549 |
| 18 | 0.652 | 0.612 | 2.204527381 | 11.0226369 | 11.33311 |
| 19 | 0.709 | 0.669 | 2.460847643 | 12.30423821 | 12.59379 |
| 20 | 0.684 | 0.644 | 2.347105915 | 11.73552958 | 12.03406 |
| 21 | 0.683 | 0.643 | 2.342599164 | 11.71299582 | 12.01189 |
| 22 | 0.699 | 0.659 | 2.415103347 | 12.07551673 | 12.36862 |
| 23 | 0.684 | 0.644 | 2.347105915 | 11.73552958 | 12.03406 |
| 24 | 0.673 | 0.633 | 2.297713232 | 11.48856616 | 11.79115 |
| 25 | 0.714 | 0.674 | 2.483843593 | 12.41921797 | 12.70701 |
| 26 | 0.661 | 0.621 | 2.244285899 | 11.22142949 | 11.52849 |
| 27 | 0.693 | 0.653 | 2.387815236 | 11.93907618 | 12.23434 |
| 28 | 0.659 | 0.619 | 2.235427563 | 11.17713781 | 11.48496 |
| 29 | 0.643 | 0.603 | 2.165036276 | 10.82518138 | 11.13911 |
| 30 | 0.691 | 0.651 | 2.378745611 | 11.89372805 | 12.18971 |
| 31 | 0.694 | 0.654 | 2.392355001 | 11.96177501 | 12.25668 |
| 32 | 0.792 | 0.752 | 2.853267053 | 14.26633526 | 14.52826 |
| 33 | 0.715 | 0.675 | 2.488452688 | 12.44226344 | 12.7297 |

| | | | | | |
|---|---|---|---|---|---|
| 34 | 0.741 | 0.701 | 2.609447931 | 13.04723965 | 13.32574 |
| 35 | 0.788 | 0.748 | 2.833833653 | 14.16916826 | 14.43234 |
| 36 | 0.771 | 0.731 | 2.751831003 | 13.75915501 | 14.02775 |
| 37 | 0.802 | 0.762 | 2.902081651 | 14.51040825 | 14.76923 |
| 38 | 0.813 | 0.773 | 2.95615902 | 14.7807951 | 15.03626 |
| 39 | 0.808 | 0.768 | 2.931528877 | 14.65764438 | 14.91463 |
| 40 | 0.757 | 0.717 | 2.685015812 | 13.42507906 | 13.69824 |
| 41 | 0.763 | 0.723 | 2.71357166 | 13.5678583 | 13.83905 |
| 42 | 0.782 | 0.742 | 2.804782595 | 14.02391297 | 14.28898 |
| 43 | 0.804 | 0.764 | 2.911884187 | 14.55942094 | 14.81763 |
| 44 | 0.732 | 0.692 | 2.567312405 | 12.83656202 | 13.11812 |
| 45 | 0.765 | 0.725 | 2.723116688 | 13.61558344 | 13.88612 |
| 46 | 0.744 | 0.704 | 2.623552531 | 13.11776266 | 13.39525 |

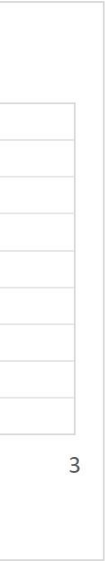


| | |
|---|---|
| 24.67096 | 24.76030038 |
| 25.68912 | 25.77650009 |
| 27.298 | 27.38287332 |
| 32.86675 | 32.9478949 |
| 26.50397 | 26.58999106 |
| 32.48578 | 32.56696338 |
| 25.84495 | 25.93206117 |
| 30.77929 | 30.86100325 |
| 21.84026 | 21.93672914 |
| 23.2817 | 23.37421796 |
| 23.40112 | 23.49333969 |
| 28.23199 | 28.31573357 |
| 14.38817 | 14.51834834 |
| 11.57814 | 11.7286898 |
| 12.05274 | 12.19946634 |
| 12.12113 | 12.2673232 |
| 11.64549 | 11.79549129 |
| 11.02264 | 11.17787393 |
| 12.30424 | 12.44901192 |
| 11.73553 | 11.88479447 |
| 11.713 | 11.86244354 |
| 12.07552 | 12.22206854 |
| 11.73553 | 11.88479447 |
| 11.48857 | 11.63985568 |
| 12.41922 | 12.56311182 |
| 11.22143 | 11.3749615 |
| 11.93908 | 12.08670661 |
| 11.17714 | 11.331047 |
| 10.82518 | 10.98214328 |
| 11.89373 | 12.04171998 |
| 11.96178 | 12.10922505 |
| 14.26634 | 14.39729636 |
| 12.44226 | 12.58598205 |

| | |
|---|---|
| 13.04724 | 13.1864881 |
| 14.16917 | 14.30075632 |
| 13.75916 | 13.89345138 |
| 14.51041 | 14.63981911 |
| 14.7808 | 14.90852899 |
| 14.65764 | 14.78613685 |
| 13.42508 | 13.56165896 |
| 13.56786 | 13.70345366 |
| 14.02391 | 14.15644882 |
| 14.55942 | 14.68852469 |
| 12.83656 | 12.97733909 |
| 13.61558 | 13.75085258 |
| 13.11776 | 13.25650598 |

| | | | |
|---|---|---|---|
| 22.92117 | 25.60996 | 24.7603 | 24.43047645 |
| 24.07724 | 22.37459 | 25.7765 | 24.07610967 |
| 21.85471 | 23.87596 | 27.38287 | 24.37118085 |
| 31.66748 | 29.41168 | 32.94789 | 31.3423531 |
| 27.44987 | 27.65956 | 26.58999 | 27.23314053 |
| 25.14267 | 30.39141 | 32.56696 | 29.36701485 |
| 27.32047 | 22.67133 | 25.93206 | 25.30795564 |
| 25.73211 | 27.3994 | 30.861 | 27.99750319 |
| 23.80754 | 21.96213 | 21.93673 | 22.5687988 |
| 24.04718 | 22.25639 | 23.37422 | 23.22593125 |
| 20.13386 | 20.06432 | 23.49334 | 21.23050656 |
| 26.90231 | 27.17269 | 28.31573 | 27.46357764 |
| 11.99404 | 14.51367 | 14.51835 | 13.67535299 |
| 12.55016 | 11.4193 | 11.72869 | 11.89938364 |
| 11.76088 | 11.7303 | 12.19947 | 11.89688052 |
| 11.53082 | 11.5078 | 12.26732 | 11.76864907 |
| 11.26635 | 11.95456 | 11.79549 | 11.67213348 |
| 11.37918 | 11.2212 | 11.17787 | 11.25941721 |
| 10.84104 | 13.59766 | 12.44901 | 12.29590142 |
| 11.04302 | 11.11176 | 11.88479 | 11.3465273 |
| 10.8228 | 10.87256 | 11.86244 | 11.18593573 |
| 10.32103 | 11.79739 | 12.22207 | 11.44683027 |
| 11.21023 | 11.17737 | 11.88479 | 11.42413135 |
| 11.87707 | 11.59659 | 11.63986 | 11.70450475 |
| 11.34148 | 10.54982 | 12.56311 | 11.48480537 |
| 10.96927 | 11.4193 | 11.37496 | 11.25451022 |
| 11.45483 | 11.93205 | 12.08671 | 11.8245293 |
| 10.65968 | 11.24314 | 11.33105 | 11.07795412 |
| 11.11712 | 10.69994 | 10.98214 | 10.93306743 |
| 11.68385 | 11.7303 | 12.04172 | 11.81862159 |
| 10.55189 | 11.17737 | 12.10923 | 11.27949732 |
| 13.10244 | 14.05247 | 14.3973 | 13.85073595 |
| 11.58805 | 11.5078 | 12.58598 | 11.89394295 |
| 12.2105 | 12.86929 | 13.18649 | 12.75542569 |
| 13.0403 | 13.86019 | 14.30076 | 13.73374938 |
| 13.10244 | 13.1258 | 13.89345 | 13.37389577 |
| 13.41605 | 14.27014 | 14.63982 | 14.1086703 |
| 11.95497 | 12.5919 | 14.90853 | 13.15179783 |
| 11.74159 | 13.31369 | 14.78614 | 13.28047273 |
| 12.36957 | 12.43127 | 13.56166 | 12.78749661 |
| 11.78019 | 12.7534 | 13.70345 | 12.74568148 |
| 12.59053 | 12.96232 | 14.15645 | 13.23643294 |
| 12.55016 | 12.7997 | 14.68852 | 13.34613013 |
| 11.04302 | 12.20329 | 12.97734 | 12.07455088 |
| 11.39806 | 11.64109 | 13.75085 | 12.26333221 |
| 12.87554 | 12.98562 | 13.25651 | 13.03922121 |

Report

FT3 (90)

| Group | Mean | Std. Deviation |
|---|---|---|
| 1 | 25.71787 | 2.9861579919834 |
| 2 | 11.66495 | 1.6083741346917 |
| 3 | 12.93256 | 4.7567279977337 |
| Total | 15.77184 | 6.2050798424557 |

Tests of Normality

| | Group | Kolmogorov-Smirnova | | | Shapiro-Wilk | | |
|---|---|---|---|---|---|---|---|
| | | Statistic | df | Sig. | Statistic | df | Sig. |
| FT3 (90) | 1 | 0.167 | 12 | .200* | 0.968 | 12 | 0.89 |
| | 2 | 0.239 | 18 | 0.008 | 0.797 | 18 | 0.001 |
| | 3 | 0.152 | 16 | .200* | 0.963 | 16 | 0.719 |

* This is a lower bound of the true significance.
a Lilliefors Significance Correction

**Hypothesis Test Summary**

| | Null Hypothesis | Test | Sig. | Decision |
|---|---|---|---|---|
| 1 | The distribution of FT3 (90) is the same across categories of Group. | Independent-Samples Kruskal-Wallis Test | .000 | Reject the null hypothesis. |

Asymptotic significances are displayed. The significance level is .05.

Multiple Comparisons

Dependent Variable: FT3（90）

Bonferroni

| (I) Grou | (J) Grou | Mean Dif | Std. Err | Sig. | 95% Confidence Interval | |
|---|---|---|---|---|---|---|
| | | | | | Lower Bo | Upper Bound |
| 1 | 2 | 14.05292 | .6040617 | 0 | 12.54805 | 15.557802442635 |
| | 3 | 12.78531 | .6189790 | 0 | 11.24327 | 14.327352855954 |
| 2 | 1 | -14.0529 | .6040617 | 0 | -15.5578 | -12.5481 |
| | 3 | -1.26761 | .5569173 | 0.084 | -2.65504 | 0.119814 |
| 3 | 1 | -12.7853 | .6189790 | 0 | -14.3274 | -11.2433 |
| | 2 | 1.267612 | .5569173 | 0.084 | -0.11981 | 2.655039 |

* The mean difference is significant at the 0.05 level.

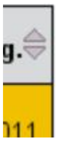
g.
011

| |
|---|
| )11 |
| )00 |
| )06 |

vel

| | | | | | |
|---|---|---|---|---|---|
| 0.068 | 0.547 | 0.988 | 1.529 | 1.892 | 2.741 |
| 0 | 0.479 | 0.92 | 1.461 | 1.824 | 2.673 |
| 0 | 7.5 | 15 | 30 | 60 | 120 |

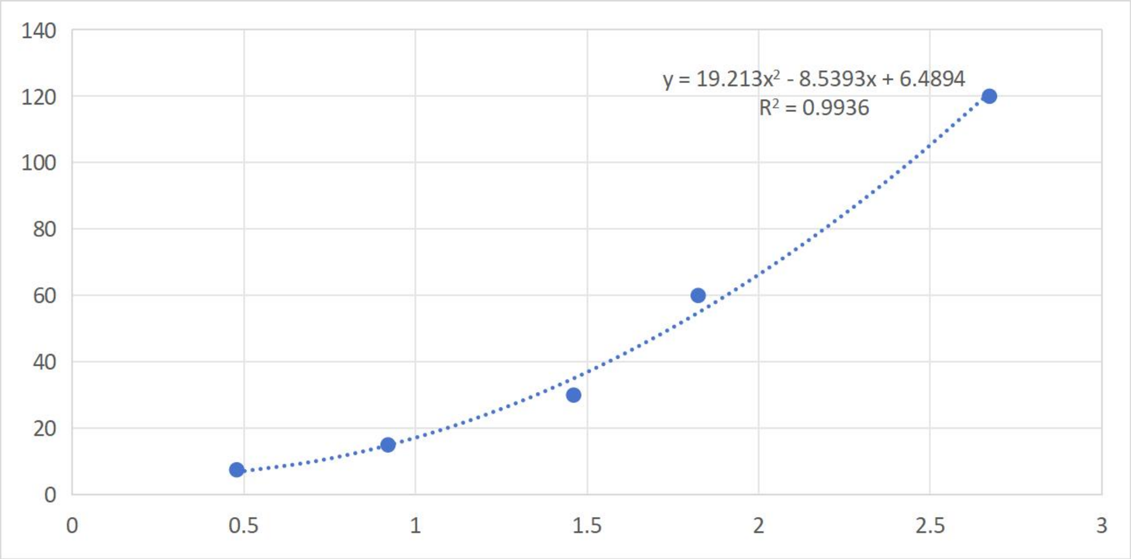


| Sample ID | OD (450n | Blank Cor | Dilution Fact | Concentration |
|---|---|---|---|---|
| 1 | 0.856 | 0.788 | 11.69062867 | 58.45314336 |
| 2 | 0.742 | 0.674 | 9.461916588 | 47.30958294 |
| 3 | 0.786 | 0.718 | 10.26294521 | 51.31472606 |
| 4 | 0.739 | 0.671 | 9.410010033 | 47.05005017 |
| 5 | 0.850 | 0.782 | 11.56087801 | 57.80439006 |
| 6 | 0.891 | 0.823 | 12.47507818 | 62.37539089 |
| 7 | 0.823 | 0.755 | 10.99411883 | 54.97059413 |
| 8 | 0.799 | 0.731 | 10.51384959 | 52.56924797 |
| 9 | 0.765 | 0.697 | 9.871356217 | 49.35678109 |
| 10 | 0.842 | 0.774 | 11.39002899 | 56.95014494 |
| 11 | 0.805 | 0.737 | 10.6318419 | 53.15920949 |
| 12 | 0.874 | 0.806 | 12.08818067 | 60.44090334 |
| 13 | 0.301 | 0.233 | 5.542797657 | 27.71398829 |
| 14 | 0.315 | 0.247 | 5.552358817 | 27.76179409 |
| 15 | 0.343 | 0.275 | 5.594075625 | 27.97037813 |
| 16 | 0.312 | 0.244 | 5.549675968 | 27.74837984 |
| 17 | 0.327 | 0.259 | 5.566548553 | 27.83274277 |
| 18 | 0.361 | 0.293 | 5.636801937 | 28.18400969 |
| 19 | 0.322 | 0.254 | 5.559963708 | 27.79981854 |
| 20 | 0.292 | 0.224 | 5.540628288 | 27.70314144 |
| 21 | 0.402 | 0.334 | 5.780599228 | 28.90299614 |
| 22 | 0.352 | 0.284 | 5.613882528 | 28.06941264 |
| 23 | 0.309 | 0.241 | 5.547338953 | 27.73669477 |
| 24 | 0.407 | 0.339 | 5.802554473 | 29.01277237 |
| 25 | 0.312 | 0.244 | 5.549675968 | 27.74837984 |
| 26 | 0.357 | 0.289 | 5.626231273 | 28.13115637 |
| 27 | 0.362 | 0.294 | 5.639540668 | 28.19770334 |
| 28 | 0.301 | 0.233 | 5.542797657 | 27.71398829 |
| 29 | 0.331 | 0.263 | 5.572508097 | 27.86254049 |

| | | | | |
|---|---|---|---|---|
| 30 | 0.295 | 0.227 | 5.541005577 | 27.70502789 |
| 31 | 0.418 | 0.35 | 5.8542375 | 29.2711875 |
| 32 | 0.423 | 0.355 | 5.879266825 | 29.39633413 |
| 33 | 0.400 | 0.332 | 5.772086112 | 28.86043056 |
| 34 | 0.358 | 0.29 | 5.6288163 | 28.1440815 |
| 35 | 0.435 | 0.367 | 5.943256657 | 29.71628329 |
| 36 | 0.498 | 0.43 | 6.3699847 | 31.8499235 |
| 37 | 0.429 | 0.361 | 5.910570073 | 29.55285037 |
| 38 | 0.393 | 0.325 | 5.743500625 | 28.71750313 |
| 39 | 0.472 | 0.404 | 6.175391808 | 30.87695904 |
| 40 | 0.461 | 0.393 | 6.100883737 | 30.50441869 |
| 41 | 0.388 | 0.32 | 5.7242352 | 28.621176 |
| 42 | 0.429 | 0.361 | 5.910570073 | 29.55285037 |
| 43 | 0.468 | 0.4 | 6.14776 | 30.7388 |
| 44 | 0.502 | 0.434 | 6.402227628 | 32.01113814 |
| 45 | 0.451 | 0.383 | 6.037183857 | 30.18591929 |
| 46 | 0.434 | 0.366 | 5.937712828 | 29.68856414 |

| | | | | | |
|---|---|---|---|---|---|
| 0.071 | 0.562 | 0.982 | 1.508 | 1.882 | 2.783 |
| 0 | 0.491 | 0.911 | 1.437 | 1.811 | 2.712 |
| 0 | 7.5 | 15 | 30 | 60 | 120 |

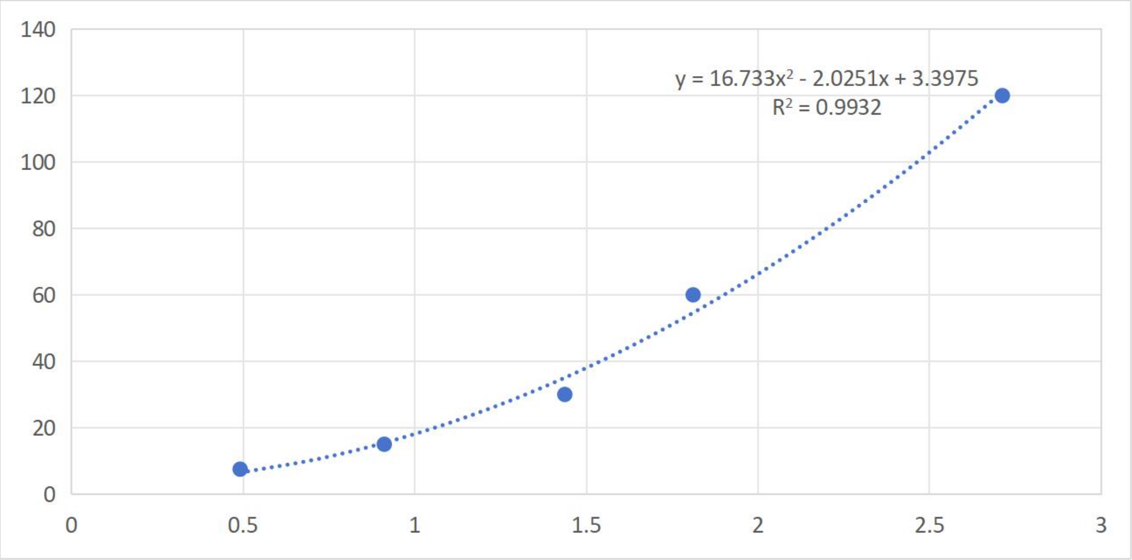


| Sample ID | OD (450n | Blank Cor | Dilution Fact | Concentration | |
|---|---|---|---|---|---|
| 1 | 0.856 | 0.785 | 12.11908943 | 60.59544713 | 58.45314 |
| 2 | 0.742 | 0.671 | 9.572540553 | 47.86270277 | 47.30958 |
| 3 | 0.786 | 0.715 | 10.50388143 | 52.51940713 | 51.31473 |
| 4 | 0.739 | 0.668 | 9.511399392 | 47.55699696 | 47.05005 |
| 5 | 0.850 | 0.779 | 11.97421755 | 59.87108777 | 57.80439 |
| 6 | 0.891 | 0.82 | 12.9881872 | 64.940936 | 62.37539 |
| 7 | 0.823 | 0.752 | 11.33720323 | 56.68601616 | 54.97059 |
| 8 | 0.799 | 0.728 | 10.79144947 | 53.95724736 | 52.56925 |
| 9 | 0.765 | 0.694 | 10.05129579 | 50.25647894 | 49.35678 |
| 10 | 0.842 | 0.771 | 11.78292915 | 58.91464577 | 56.95014 |
| 11 | 0.805 | 0.734 | 10.92608075 | 54.63040374 | 53.15921 |
| 12 | 0.874 | 0.803 | 12.5609337 | 62.80466849 | 60.4409 |
| 13 | 0.301 | 0.23 | 3.8169027 | 19.0845135 | 27.71399 |
| 14 | 0.315 | 0.244 | 3.899591488 | 19.49795744 | 27.76179 |
| 15 | 0.343 | 0.272 | 4.084647072 | 20.42323536 | 27.97038 |
| 16 | 0.312 | 0.241 | 3.881320273 | 19.40660137 | 27.74838 |
| 17 | 0.327 | 0.256 | 3.975688288 | 19.87844144 | 27.83274 |
| 18 | 0.361 | 0.29 | 4.2174663 | 21.0873315 | 28.18401 |
| 19 | 0.322 | 0.251 | 3.943395633 | 19.71697817 | 27.79982 |
| 20 | 0.292 | 0.221 | 3.767209353 | 18.83604677 | 27.70314 |
| 21 | 0.402 | 0.331 | 4.560476113 | 22.80238057 | 28.903 |
| 22 | 0.352 | 0.281 | 4.149701313 | 20.74850657 | 28.06941 |
| 23 | 0.309 | 0.238 | 3.863350252 | 19.31675126 | 27.73669 |
| 24 | 0.407 | 0.336 | 4.606155168 | 23.03077584 | 29.01277 |
| 25 | 0.312 | 0.241 | 3.881320273 | 19.40660137 | 27.74838 |
| 26 | 0.357 | 0.286 | 4.187013868 | 20.93506934 | 28.13116 |
| 27 | 0.362 | 0.291 | 4.225163073 | 21.12581537 | 28.1977 |
| 28 | 0.301 | 0.23 | 3.8169027 | 19.0845135 | 27.71399 |
| 29 | 0.331 | 0.26 | 4.0021248 | 20.010624 | 27.86254 |

| | | | | | |
|---|---|---|---|---|---|
| 30 | 0.295 | 0.224 | 3.783472608 | 18.91736304 | 27.70503 |
| 31 | 0.418 | 0.347 | 4.709594097 | 23.54797049 | 29.27119 |
| 32 | 0.423 | 0.352 | 4.757950432 | 23.78975216 | 29.39633 |
| 33 | 0.400 | 0.329 | 4.542438753 | 22.71219377 | 28.86043 |
| 34 | 0.358 | 0.287 | 4.194576777 | 20.97288389 | 28.14408 |
| 35 | 0.435 | 0.364 | 4.877419168 | 24.38709584 | 29.71628 |
| 36 | 0.498 | 0.427 | 5.583693457 | 27.91846729 | 31.84992 |
| 37 | 0.429 | 0.358 | 4.817082412 | 24.08541206 | 29.55285 |
| 38 | 0.393 | 0.322 | 4.480362172 | 22.40181086 | 28.7175 |
| 39 | 0.472 | 0.401 | 5.276118033 | 26.38059017 | 30.87696 |
| 40 | 0.461 | 0.39 | 5.1528003 | 25.7640015 | 30.50442 |
| 41 | 0.388 | 0.317 | 4.437025737 | 22.18512869 | 28.62118 |
| 42 | 0.429 | 0.358 | 4.817082412 | 24.08541206 | 29.55285 |
| 43 | 0.468 | 0.397 | 5.230806697 | 26.15403349 | 30.7388 |
| 44 | 0.502 | 0.431 | 5.633020713 | 28.16510357 | 32.01114 |
| 45 | 0.451 | 0.38 | 5.0442072 | 25.221036 | 30.18592 |
| 46 | 0.434 | 0.363 | 4.867279377 | 24.33639689 | 29.68856 |

| | |
|---|---|
| 60.59545 | 59.52429525 |
| 47.8627 | 47.58614286 |
| 52.51941 | 51.9170666 |
| 47.557 | 47.30352357 |
| 59.87109 | 58.83773892 |
| 64.94094 | 63.65816345 |
| 56.68602 | 55.82830515 |
| 53.95725 | 53.26324767 |
| 50.25648 | 49.80663002 |
| 58.91465 | 57.93239536 |
| 54.6304 | 53.89480662 |
| 62.80467 | 61.62278592 |
| 19.08451 | 23.3992509 |
| 19.49796 | 23.62987577 |
| 20.42324 | 24.19680675 |
| 19.4066 | 23.57749061 |
| 19.87844 | 23.85559211 |
| 21.08733 | 24.6356706 |
| 19.71698 | 23.75839836 |
| 18.83605 | 23.26959411 |
| 22.80238 | 25.85268836 |
| 20.74851 | 24.40895961 |
| 19.31675 | 23.52672302 |
| 23.03078 | 26.02177411 |
| 19.4066 | 23.57749061 |
| 20.93507 | 24.53311286 |
| 21.12582 | 24.66175936 |
| 19.08451 | 23.3992509 |
| 20.01062 | 23.93658225 |

| | |
|---|---|
| 18.91736 | 23.31119547 |
| 23.54797 | 26.409579 |
| 23.78975 | 26.59304315 |
| 22.71219 | 25.78631217 |
| 20.97288 | 24.5584827 |
| 24.3871 | 27.05168957 |
| 27.91847 | 29.8841954 |
| 24.08541 | 26.81913122 |
| 22.40181 | 25.559657 |
| 26.38059 | 28.62877461 |
| 25.764 | 28.1342101 |
| 22.18513 | 25.40315235 |
| 24.08541 | 26.81913122 |
| 26.15403 | 28.44641675 |
| 28.1651 | 30.08812086 |
| 25.22104 | 27.70347765 |
| 24.3364 | 27.01248052 |

| | | | | | |
|---|---|---|---|---|---|
| 0.074 | 0.572 | 0.977 | 1.511 | 1.986 | 2.777 |
| 0.009 | 0.507 | 0.912 | 1.446 | 1.921 | 2.712 |
| 0 | 7.5 | 15 | 30 | 60 | 120 |

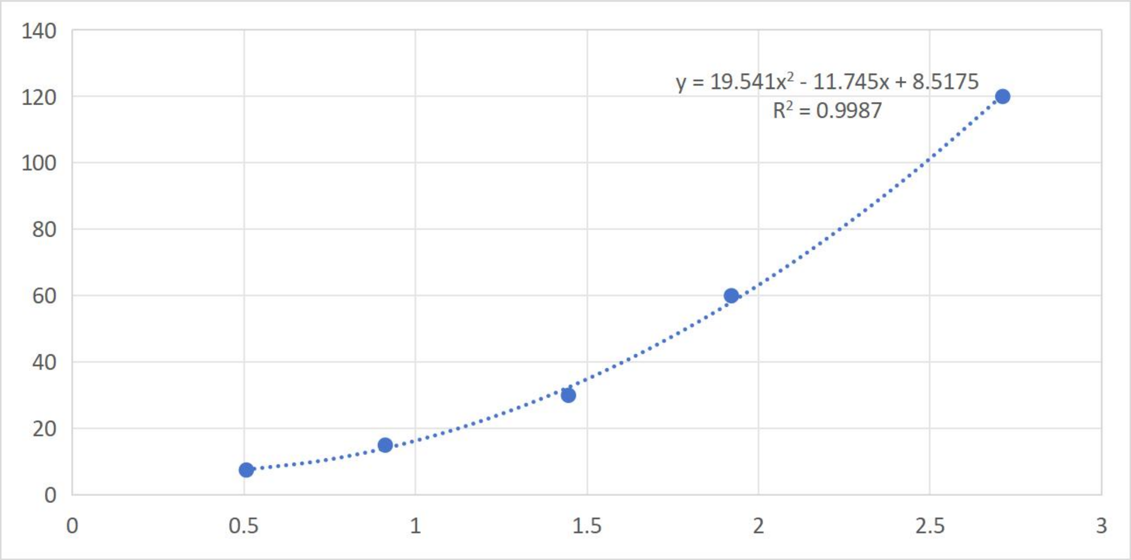


| Sample ID | OD (450n | Blank Cor | Dilution Fact | Concentration | Sample ID |
|---|---|---|---|---|---|
| 1 | 0.885 | 0.82 | 12.0259684 | 60.129842 | 1 |
| 2 | 0.763 | 0.698 | 9.839943364 | 49.19971682 | 2 |
| 3 | 0.792 | 0.727 | 10.30687019 | 51.53435095 | 3 |
| 4 | 0.724 | 0.659 | 9.263830021 | 46.31915011 | 4 |
| 5 | 0.866 | 0.801 | 11.64728014 | 58.23640071 | 5 |
| 6 | 0.901 | 0.836 | 12.35580674 | 61.77903368 | 6 |
| 7 | 0.859 | 0.794 | 11.51131988 | 57.55659938 | 7 |
| 8 | 0.788 | 0.723 | 10.24051239 | 51.20256195 | 8 |
| 9 | 0.758 | 0.693 | 9.762760709 | 48.81380355 | 9 |
| 10 | 0.874 | 0.809 | 11.80500822 | 59.02504111 | 10 |
| 11 | 0.823 | 0.758 | 10.84234512 | 54.21172562 | 11 |
| 12 | 0.892 | 0.827 | 12.16904159 | 60.84520795 | 12 |
| 13 | 0.325 | 0.26 | 6.7847716 | 33.923858 | 13 |
| 14 | 0.309 | 0.244 | 6.815112976 | 34.07556488 | 14 |
| 15 | 0.348 | 0.283 | 6.758684149 | 33.79342075 | 15 |
| 16 | 0.316 | 0.251 | 6.800607541 | 34.00303771 | 16 |
| 17 | 0.337 | 0.272 | 6.768581344 | 33.84290672 | 17 |
| 18 | 0.402 | 0.337 | 6.778686829 | 33.89343415 | 18 |
| 19 | 0.317 | 0.252 | 6.798691664 | 33.99345832 | 19 |
| 20 | 0.308 | 0.243 | 6.817341509 | 34.08670755 | 20 |
| 21 | 0.401 | 0.336 | 6.777280736 | 33.88640368 | 21 |
| 22 | 0.363 | 0.298 | 6.752808964 | 33.76404482 | 22 |
| 23 | 0.327 | 0.262 | 6.781682404 | 33.90841202 | 23 |
| 24 | 0.345 | 0.28 | 6.7609144 | 33.804572 | 24 |
| 25 | 0.348 | 0.283 | 6.758684149 | 33.79342075 | 25 |
| 26 | 0.379 | 0.314 | 6.756234436 | 33.78117218 | 26 |
| 27 | 0.404 | 0.339 | 6.781616261 | 33.90808131 | 27 |
| 28 | 0.321 | 0.256 | 6.791418976 | 33.95709488 | 28 |
| 29 | 0.342 | 0.277 | 6.763496389 | 33.81748195 | 29 |
| 30 | 0.357 | 0.292 | 6.754103824 | 33.77051912 | 30 |
| 31 | 0.432 | 0.367 | 6.839042749 | 34.19521375 | 31 |
| 32 | 0.419 | 0.354 | 6.808569956 | 34.04284978 | 32 |

| | | | | | |
|---|---|---|---|---|---|
| 33 | 0.407 | 0.342 | 6.786303524 | 33.93151762 | 33 |
| 34 | 0.338 | 0.273 | 6.767486189 | 33.83743095 | 34 |
| 35 | 0.382 | 0.317 | 6.757990549 | 33.78995275 | 35 |
| 36 | 0.447 | 0.382 | 6.882410884 | 34.41205442 | 36 |
| 37 | 0.403 | 0.338 | 6.780132004 | 33.90066002 | 37 |
| 38 | 0.505 | 0.44 | 7.1328376 | 35.664188 | 38 |
| 39 | 0.423 | 0.358 | 6.817242724 | 34.08621362 | 39 |
| 40 | 0.411 | 0.346 | 6.793100356 | 33.96550178 | 40 |
| 41 | 0.427 | 0.362 | 6.826540804 | 34.13270402 | 41 |
| 42 | 0.399 | 0.334 | 6.774585796 | 33.87292898 | 42 |
| 43 | 0.489 | 0.424 | 7.050622816 | 35.25311408 | 43 |
| 44 | 0.502 | 0.437 | 7.116660229 | 35.58330115 | 44 |
| 45 | 0.414 | 0.349 | 6.798608341 | 33.99304171 | 45 |
| 46 | 0.471 | 0.406 | 6.970090276 | 34.85045138 | 46 |

| | | | | | |
|---|---|---|---|---|---|
| 0.062 | 0.554 | 0.996 | 1.512 | 1.905 | 2.806 |
| 0 | 0.492 | 0.934 | 1.45 | 1.843 | 2.744 |
| 0 | 7.5 | 15 | 30 | 60 | 120 |

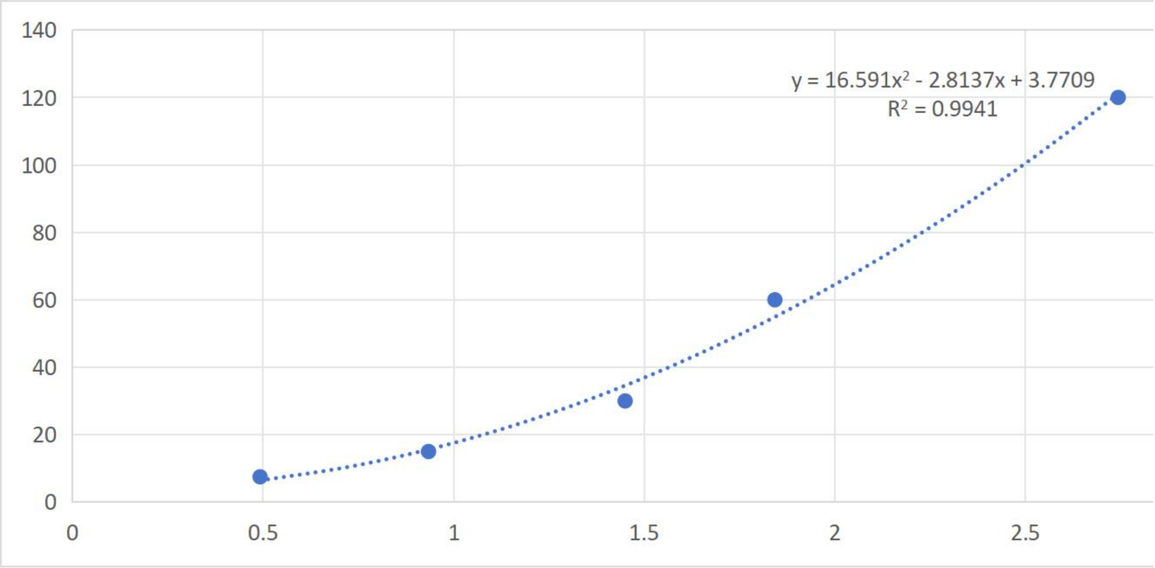


| OD (450n | Blank Cor | Dilution Fact | Concentration | | |
|---|---|---|---|---|---|
| 0.885 | 0.823 | 12.69279034 | 63.4639517 | 60.129842 | 63.46395 |
| 0.763 | 0.701 | 9.951330291 | 49.75665146 | 49.1997168 | 49.75665 |
| 0.792 | 0.73 | 10.5582429 | 52.7912145 | 51.534351 | 52.79121 |
| 0.724 | 0.662 | 9.179136804 | 45.89568402 | 46.3191501 | 45.89568 |
| 0.866 | 0.804 | 12.23337306 | 61.16686528 | 58.2364007 | 61.16687 |
| 0.901 | 0.839 | 13.08895901 | 65.44479506 | 61.7790337 | 65.4448 |
| 0.859 | 0.797 | 12.06713362 | 60.3356681 | 57.5565994 | 60.33567 |
| 0.788 | 0.726 | 10.47287172 | 52.36435858 | 51.202562 | 52.36436 |
| 0.758 | 0.696 | 9.849510656 | 49.24755328 | 48.8138036 | 49.24755 |
| 0.874 | 0.812 | 12.4253519 | 62.12675952 | 59.0250411 | 62.12676 |
| 0.823 | 0.761 | 11.23787081 | 56.18935406 | 54.2117256 | 56.18935 |
| 0.892 | 0.83 | 12.8650689 | 64.3253445 | 60.845208 | 64.32534 |
| 0.325 | 0.263 | 4.178479779 | 20.8923989 | 33.923858 | 20.8924 |
| 0.309 | 0.247 | 4.088116419 | 20.4405821 | 34.0755649 | 20.44058 |
| 0.348 | 0.286 | 4.323259236 | 21.61629618 | 33.7934208 | 21.6163 |
| 0.316 | 0.254 | 4.126605156 | 20.63302578 | 34.0030377 | 20.63303 |
| 0.337 | 0.275 | 4.251826875 | 21.25913438 | 33.8429067 | 21.25913 |
| 0.402 | 0.34 | 4.7321616 | 23.660808 | 33.8934342 | 23.66081 |
| 0.317 | 0.255 | 4.132236275 | 20.66118138 | 33.9934583 | 20.66118 |
| 0.308 | 0.246 | 4.082750756 | 20.41375378 | 34.0867076 | 20.41375 |
| 0.401 | 0.339 | 4.723710011 | 23.61855006 | 33.8864037 | 23.61855 |
| 0.363 | 0.301 | 4.427137491 | 22.13568746 | 33.7640448 | 22.13569 |
| 0.327 | 0.265 | 4.190372475 | 20.95186238 | 33.908412 | 20.95186 |
| 0.345 | 0.283 | 4.303379499 | 21.5168975 | 33.804572 | 21.5169 |
| 0.348 | 0.286 | 4.323259236 | 21.61629618 | 33.7934208 | 21.6163 |
| 0.379 | 0.317 | 4.546170099 | 22.7308505 | 33.7811722 | 22.73085 |
| 0.404 | 0.342 | 4.749164324 | 23.74582162 | 33.9080813 | 23.74582 |
| 0.321 | 0.259 | 4.155092571 | 20.77546286 | 33.9570949 | 20.77546 |
| 0.342 | 0.28 | 4.2837984 | 21.418992 | 33.817482 | 21.41899 |
| 0.357 | 0.295 | 4.384690275 | 21.92345138 | 33.7705191 | 21.92345 |
| 0.432 | 0.37 | 5.0011389 | 25.0056945 | 34.1952138 | 25.00569 |
| 0.419 | 0.357 | 4.880915459 | 24.4045773 | 34.0428498 | 24.40458 |

| | | | | | |
|---|---|---|---|---|---|
| 0.407 | 0.345 | 4.774917275 | 23.87458638 | 33.9315176 | 23.87459 |
| 0.338 | 0.276 | 4.258154816 | 21.29077408 | 33.837431 | 21.29077 |
| 0.382 | 0.32 | 4.5694344 | 22.847172 | 33.7899528 | 22.84717 |
| 0.447 | 0.385 | 5.146826475 | 25.73413238 | 34.4120544 | 25.73413 |
| 0.403 | 0.341 | 4.740646371 | 23.70323186 | 33.90066 | 23.70323 |
| 0.505 | 0.443 | 5.780398059 | 28.9019903 | 35.664188 | 28.90199 |
| 0.423 | 0.361 | 4.917310011 | 24.58655006 | 34.0862136 | 24.58655 |
| 0.411 | 0.349 | 4.809719091 | 24.04859546 | 33.9655018 | 24.0486 |
| 0.427 | 0.365 | 4.954235475 | 24.77117738 | 34.132704 | 24.77118 |
| 0.399 | 0.337 | 4.706906379 | 23.5345319 | 33.872929 | 23.53453 |
| 0.489 | 0.427 | 5.594470539 | 27.9723527 | 35.2531141 | 27.97235 |
| 0.502 | 0.44 | 5.7448896 | 28.724448 | 35.5833012 | 28.72445 |
| 0.414 | 0.352 | 4.836168864 | 24.18084432 | 33.9930417 | 24.18084 |
| 0.471 | 0.409 | 5.395455771 | 26.97727886 | 34.8504514 | 26.97728 |

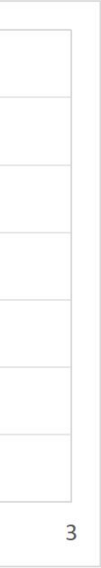


61.79689685
49.47818414
52.16278273
46.10741707
59.701633
63.61191437
58.94613374
51.78346027
49.03067842
60.57590032
55.20053984
62.58527623
27.40812845
27.25807349
27.70485847
27.31803175
27.55102055
28.77712108
27.32731985
27.25023067
28.75247687
27.94986614
27.4301372
27.66073475
27.70485847
28.25601134
28.82695147
27.36627887
27.61823698
27.84698525
29.60045413
29.22371354

| |
|---|
| 28.903052 |
| 27.56410252 |
| 28.31856238 |
| 30.0730934 |
| 28.80194594 |
| 32.28308915 |
| 29.33638184 |
| 29.00704862 |
| 29.4519407 |
| 28.70373044 |
| 31.61273339 |
| 32.15387458 |
| 29.08694302 |
| 30.91386512 |

| | | | | | |
|---|---|---|---|---|---|
| 0.06 | 0.561 | 0.991 | 1.511 | 1.978 | 2.833 |
| 0 | 0.501 | 0.931 | 1.451 | 1.918 | 2.773 |
| 0 | 7.5 | 15 | 30 | 60 | 120 |

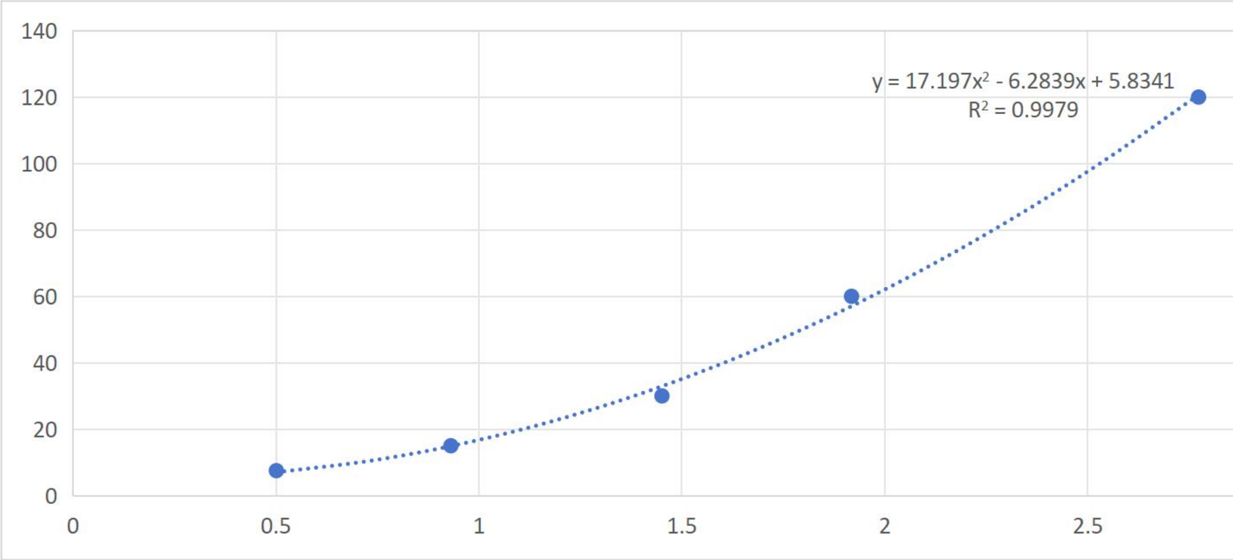


| Sample ID | OD （450n | Blank Correct | Dilution Fact | Concentration |
|---|---|---|---|---|
| 1 | 0.823 | 0.763 | 11.05104459 | 55.25522297 |
| 2 | 0.799 | 0.739 | 10.58194074 | 52.90970369 |
| 3 | 0.819 | 0.759 | 10.97148486 | 54.85742429 |
| 4 | 0.791 | 0.731 | 10.42997522 | 52.14987609 |
| 5 | 0.872 | 0.812 | 12.07031197 | 60.35155984 |
| 6 | 0.884 | 0.824 | 12.33251667 | 61.66258336 |
| 7 | 0.865 | 0.805 | 11.91964643 | 59.59823213 |
| 8 | 0.818 | 0.758 | 10.95168091 | 54.75840454 |
| 9 | 0.845 | 0.785 | 11.49845983 | 57.49229913 |
| 10 | 0.908 | 0.848 | 12.87178429 | 64.35892144 |
| 11 | 0.791 | 0.731 | 10.42997522 | 52.14987609 |
| 12 | 0.922 | 0.862 | 13.19550587 | 65.97752934 |
| 13 | 0.304 | 0.244 | 5.324668992 | 26.62334496 |
| 14 | 0.298 | 0.238 | 5.312638668 | 26.56319334 |
| 15 | 0.306 | 0.246 | 5.328954252 | 26.64477126 |
| 16 | 0.314 | 0.254 | 5.347471052 | 26.73735526 |
| 17 | 0.317 | 0.257 | 5.354982353 | 26.77491177 |
| 18 | 0.321 | 0.261 | 5.365478937 | 26.82739469 |
| 19 | 0.292 | 0.232 | 5.301846528 | 26.50923264 |
| 20 | 0.281 | 0.221 | 5.285276777 | 26.42638389 |
| 21 | 0.352 | 0.292 | 5.465486208 | 27.32743104 |
| 22 | 0.312 | 0.252 | 5.342635488 | 26.71317744 |
| 23 | 0.305 | 0.245 | 5.326794425 | 26.63397213 |
| 24 | 0.400 | 0.34 | 5.6855472 | 28.427736 |
| 25 | 0.335 | 0.275 | 5.406550625 | 27.03275313 |
| 26 | 0.323 | 0.263 | 5.370933593 | 26.85466797 |
| 27 | 0.382 | 0.322 | 5.593737948 | 27.96868974 |
| 28 | 0.331 | 0.271 | 5.394127977 | 26.97063989 |
| 29 | 0.371 | 0.311 | 5.543118137 | 27.71559069 |
| 30 | 0.302 | 0.242 | 5.320521308 | 26.60260654 |

| | | | | |
|---|---|---|---|---|
| 31 | 0.444 | 0.384 | 5.956883232 | 29.78441616 |
| 32 | 0.401 | 0.341 | 5.690974457 | 28.45487229 |
| 33 | 0.372 | 0.312 | 5.547547968 | 27.73773984 |
| 34 | 0.363 | 0.303 | 5.508917673 | 27.54458837 |
| 35 | 0.391 | 0.331 | 5.638249617 | 28.19124809 |
| 36 | 0.389 | 0.329 | 5.628117377 | 28.14058689 |
| 37 | 0.396 | 0.336 | 5.664182112 | 28.32091056 |
| 38 | 0.403 | 0.343 | 5.701932153 | 28.50966077 |
| 39 | 0.398 | 0.338 | 5.674795868 | 28.37397934 |
| 40 | 0.421 | 0.361 | 5.806742337 | 29.03371169 |
| 41 | 0.463 | 0.403 | 6.094635873 | 30.47317937 |
| 42 | 0.433 | 0.373 | 5.882806713 | 29.41403357 |
| 43 | 0.415 | 0.355 | 5.770567425 | 28.85283713 |
| 44 | 0.463 | 0.403 | 6.094635873 | 30.47317937 |
| 45 | 0.432 | 0.372 | 5.876278848 | 29.38139424 |
| 46 | 0.377 | 0.317 | 5.570213033 | 27.85106517 |

| | |
|---|---|
| 0.077 | 0.544 |
| 0 | 0.467 |
| 0 | 7.5 |

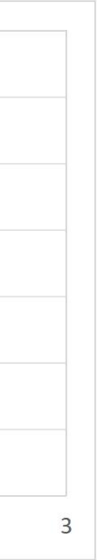


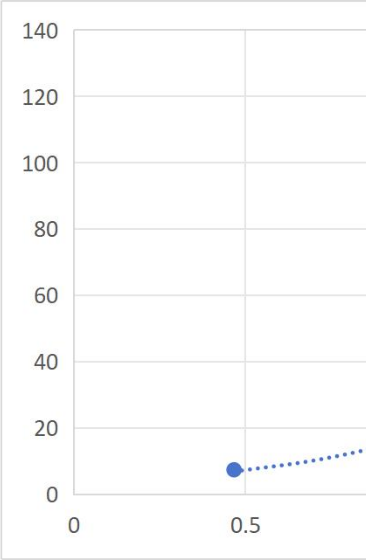


| Sample ID | OD （450n | Blank Cor |
|---|---|---|
| 1 | 0.823 | 0.746 |
| 2 | 0.799 | 0.722 |
| 3 | 0.819 | 0.742 |
| 4 | 0.791 | 0.714 |
| 5 | 0.872 | 0.795 |
| 6 | 0.884 | 0.807 |
| 7 | 0.865 | 0.788 |
| 8 | 0.818 | 0.741 |
| 9 | 0.845 | 0.768 |
| 10 | 0.908 | 0.831 |
| 11 | 0.791 | 0.714 |
| 12 | 0.922 | 0.845 |
| 13 | 0.304 | 0.227 |
| 14 | 0.298 | 0.221 |
| 15 | 0.306 | 0.229 |
| 16 | 0.314 | 0.237 |
| 17 | 0.317 | 0.24 |
| 18 | 0.321 | 0.244 |
| 19 | 0.292 | 0.215 |
| 20 | 0.281 | 0.204 |
| 21 | 0.352 | 0.275 |
| 22 | 0.312 | 0.235 |
| 23 | 0.305 | 0.228 |
| 24 | 0.400 | 0.323 |
| 25 | 0.335 | 0.258 |
| 26 | 0.323 | 0.246 |
| 27 | 0.382 | 0.305 |
| 28 | 0.331 | 0.254 |
| 29 | 0.371 | 0.294 |
| 30 | 0.302 | 0.225 |

| | | |
|---|---|---|
| 31 | 0.444 | 0.367 |
| 32 | 0.401 | 0.324 |
| 33 | 0.372 | 0.295 |
| 34 | 0.363 | 0.286 |
| 35 | 0.391 | 0.314 |
| 36 | 0.389 | 0.312 |
| 37 | 0.396 | 0.319 |
| 38 | 0.403 | 0.326 |
| 39 | 0.398 | 0.321 |
| 40 | 0.421 | 0.344 |
| 41 | 0.463 | 0.386 |
| 42 | 0.433 | 0.356 |
| 43 | 0.415 | 0.338 |
| 44 | 0.463 | 0.386 |
| 45 | 0.432 | 0.355 |
| 46 | 0.377 | 0.3 |

| | | | |
|---|---|---|---|
| 0.988 | 1.501 | 1.963 | 2.81 |
| 0.911 | 1.424 | 1.886 | 2.733 |
| 15 | 30 | 60 | 120 |

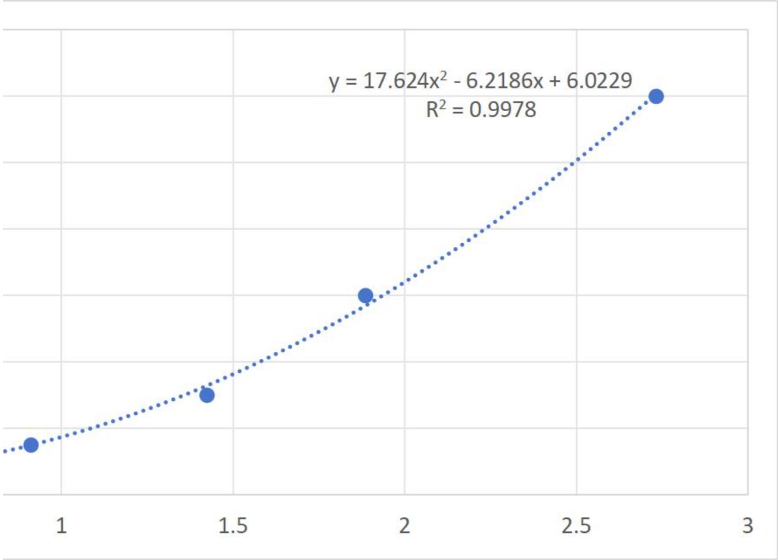


| Dilution Fact | Concentration | | |
|---|---|---|---|
| 11.19186238 | 55.95931192 | 55.25522 | 55.95931 |
| 10.72018002 | 53.60090008 | 52.9097 | 53.6009 |
| 11.11183874 | 55.55919368 | 54.85742 | 55.55919 |
| 10.5674643 | 52.83732152 | 52.14988 | 52.83732 |
| 12.2179216 | 61.089608 | 60.35156 | 61.08961 |
| 12.48210218 | 62.41051088 | 61.66258 | 62.41051 |
| 12.06616026 | 60.33080128 | 59.59823 | 60.3308 |
| 11.09192094 | 55.45960472 | 54.7584 | 55.4596 |
| 11.64207338 | 58.21036688 | 57.4923 | 58.21037 |
| 13.02569046 | 65.12845232 | 64.35892 | 65.12845 |
| 10.5674643 | 52.83732152 | 52.14988 | 52.83732 |
| 13.3521596 | 66.760798 | 65.97753 | 66.7608 |
| 5.519424896 | 27.59712448 | 26.62334 | 27.59712 |
| 5.509363184 | 27.54681592 | 26.56319 | 27.54682 |
| 5.523060784 | 27.61530392 | 26.64477 | 27.6153 |
| 5.539014256 | 27.69507128 | 26.73736 | 27.69507 |
| 5.5455784 | 27.727892 | 26.77491 | 27.72789 |
| 5.554824064 | 27.77412032 | 26.82739 | 27.77412 |
| 5.5005704 | 27.502852 | 26.50923 | 27.50285 |
| 5.487745984 | 27.43872992 | 26.42638 | 27.43873 |
| 5.6456 | 28.228 | 27.32743 | 28.228 |
| 5.5348144 | 27.674072 | 26.71318 | 27.67407 |
| 5.521225216 | 27.60612608 | 26.63397 | 27.60613 |
| 5.852986496 | 29.26493248 | 28.42774 | 29.26493 |
| 5.591625136 | 27.95812568 | 27.03275 | 27.95813 |
| 5.559658384 | 27.79829192 | 26.85467 | 27.79829 |
| 5.7656996 | 28.828498 | 27.96869 | 28.8285 |
| 5.580405584 | 27.90202792 | 26.97064 | 27.90203 |
| 5.717979664 | 28.58989832 | 27.71559 | 28.5899 |
| 5.51593 | 27.57965 | 26.60261 | 27.57965 |

| | | | |
|---|---|---|---|
| 6.114432736 | 30.57216368 | 29.78442 | 30.57216 |
| 5.858170624 | 29.29085312 | 28.45487 | 29.29085 |
| 5.7221416 | 28.610708 | 27.73774 | 28.61071 |
| 5.685953104 | 28.42976552 | 27.54459 | 28.42977 |
| 5.807915504 | 29.03957752 | 28.19125 | 29.03958 |
| 5.798287456 | 28.99143728 | 28.14059 | 28.99144 |
| 5.832602464 | 29.16301232 | 28.32091 | 29.16301 |
| 5.868644624 | 29.34322312 | 28.50966 | 29.34322 |
| 5.842723984 | 29.21361992 | 28.37398 | 29.21362 |
| 5.969255264 | 29.84627632 | 29.03371 | 29.84628 |
| 6.248425904 | 31.24212952 | 30.47318 | 31.24213 |
| 6.042673664 | 30.21336832 | 29.41403 | 30.21337 |
| 5.934449456 | 29.67224728 | 28.85284 | 29.67225 |
| 6.248425904 | 31.24212952 | 30.47318 | 31.24213 |
| 6.0363616 | 30.181808 | 29.38139 | 30.18181 |
| 5.74348 | 28.7174 | 27.85107 | 28.7174 |

55.60726745
53.25530189
55.20830899
52.49359881
60.72058392
62.03654712
59.96451671
55.10900463
57.85133301
64.74368688
52.49359881
66.36916367
27.11023472
27.05500463
27.13003759
27.21621327
27.25140189
27.30075751
27.00604232
26.93255691
27.77771552
27.19362472
27.12004911
28.84633424
27.49543941
27.32647995
28.39859387
27.43633391
28.15274451
27.09112827

30.17828992
28.87286271
28.17422392
27.98717695
28.61541281
28.56601209
28.74196144
28.92644195
28.79379963
29.43999401
30.85765445
29.81370095
29.26254221
30.85765445
29.78160112
28.28423259

| | | | |
|---|---|---|---|
| 59.5243 | 61.7969 | 55.60727 | 58.97615318 |
| 47.58614 | 49.47818 | 53.2553 | 50.10654296 |
| 51.91707 | 52.16278 | 55.20831 | 53.09605277 |
| 47.30352 | 46.10742 | 52.4936 | 48.63484648 |
| 58.83774 | 59.70163 | 60.72058 | 59.75331861 |
| 63.65816 | 63.61191 | 62.03655 | 63.10220831 |
| 55.82831 | 58.94613 | 59.96452 | 58.24631853 |
| 53.26325 | 51.78346 | 55.109 | 53.38523752 |
| 49.80663 | 49.03068 | 57.85133 | 52.22954715 |
| 57.9324 | 60.5759 | 64.74369 | 61.08399419 |
| 53.89481 | 55.20054 | 52.4936 | 53.86298176 |
| 61.62279 | 62.58528 | 66.36916 | 63.52574194 |
| 23.39925 | 27.40813 | 27.11023 | 25.97253802 |
| 23.62988 | 27.25807 | 27.055 | 25.98098463 |
| 24.19681 | 27.70486 | 27.13004 | 26.34390094 |
| 23.57749 | 27.31803 | 27.21621 | 26.03724521 |
| 23.85559 | 27.55102 | 27.2514 | 26.21933818 |
| 24.63567 | 28.77712 | 27.30076 | 26.9045164 |
| 23.7584 | 27.32732 | 27.00604 | 26.03058684 |
| 23.26959 | 27.25023 | 26.93256 | 25.81746056 |
| 25.85269 | 28.75248 | 27.77772 | 27.46096025 |
| 24.40896 | 27.94987 | 27.19362 | 26.51748349 |
| 23.52672 | 27.43014 | 27.12005 | 26.02563644 |
| 26.02177 | 27.66073 | 28.84633 | 27.50961437 |
| 23.57749 | 27.70486 | 27.49544 | 26.25926283 |
| 24.53311 | 28.25601 | 27.32648 | 26.70520138 |
| 24.66176 | 28.82695 | 28.39859 | 27.29576823 |
| 23.39925 | 27.36628 | 27.43633 | 26.06728789 |
| 23.93658 | 27.61824 | 28.15274 | 26.56918791 |
| 23.3112 | 27.84699 | 27.09113 | 26.083103 |
| 26.40958 | 29.60045 | 30.17829 | 28.72944102 |
| 26.59304 | 29.22371 | 28.87286 | 28.22987313 |
| 25.78631 | 28.90305 | 28.17422 | 27.62119603 |
| 24.55848 | 27.5641 | 27.98718 | 26.70325406 |
| 27.05169 | 28.31856 | 28.61541 | 27.99522159 |
| 29.8842 | 30.07309 | 28.56601 | 29.50776696 |
| 26.81913 | 28.80195 | 28.74196 | 28.12101287 |
| 25.55966 | 32.28309 | 28.92644 | 28.9230627 |
| 28.62877 | 29.33638 | 28.7938 | 28.91965203 |
| 28.13421 | 29.00705 | 29.43999 | 28.86041758 |
| 25.40315 | 29.45194 | 30.85765 | 28.57091583 |
| 26.81913 | 28.70373 | 29.8137 | 28.44552087 |
| 28.44642 | 31.61273 | 29.26254 | 29.77389745 |
| 30.08812 | 32.15387 | 30.85765 | 31.03321663 |
| 27.70348 | 29.08694 | 29.7816 | 28.8573406 |
| 27.01248 | 30.91387 | 28.28423 | 28.73685941 |

Report

FT4 (90)

| Group | Mean | Std. Deviation |
|---|---|---|
| 1 | 56.33357[illegible] | 5.0687282496286 |
| 2 | 26.43333[illegible] | .5369826781860 |
| 3 | 28.68929[illegible] | .9551167425621 |
| Total | 35.01807[illegible] | 13.0986183961301 |

Tests of Normality

| | Group | Kolmogorov-Smirnova | | | Shapiro-Wilk | | |
|---|---|---|---|---|---|---|---|
| | | Statisti[illegible] | df | Sig. | Statisti[illegible] | df | Sig. |
| FT4 (90) | 1 | 0.187 | 12 | .200* | 0.935 | 12 | 0.439 |
| | 2 | 0.187 | 18 | 0.095 | 0.854 | 18 | 0.01 |
| | 3 | 0.216 | 16 | 0.045 | 0.943 | 16 | 0.394 |

* This is a lower bound of the true significance.
a Lilliefors Significance Correction

**Hypothesis Test Summary**

| | Null Hypothesis | Test | Sig. | Decision |
|---|---|---|---|---|
| 1 | The distribution of FT4 (90) is the same across categories of Group. | Independent-Samples Kruskal-Wallis Test | .000 | Reject the null hypothesis. |

Asymptotic significances are displayed. The significance level is .05.

Multiple Comparisons

Dependent Variable: FT4（90）

Bonferroni

| (I) Grou | (J) Grou | Mean Dif | Std. Err | Sig. | 95% Confidence Interval | |
|---|---|---|---|---|---|---|
| | | | | | Lower Bo | Upper Bound |
| 1 | 2 | 29.90024 | .98633712 | 0 | 27.44301 | 32.357464208018 |
| | 3 | 27.64428 | 1.010694 | 0 | 25.12638 | 30.162192562349 |
| 2 | 1 | -29.9002 | .98633712 | 0 | -32.3575 | -27.443 |
| | 3 | -2.25595 | .90935789 | 0.051 | -4.5214 | 0.009495 |
| 3 | 1 | -27.6442 | 1.010694 | 0 | -30.1622 | -25.1264 |
| | 2 | 2.255952 | .90935789 | 0.051 | -0.00949 | 4.521401 |

* The mean difference is significant at the 0.05 level.

| | | | | | |
|---|---|---|---|---|---|
| 0.07 | 0.463 | 0.952 | 1.511 | 1.936 | 2.598 |
| 0 | 0.393 | 0.882 | 1.441 | 1.866 | 2.528 |
| 0 | 1.25 | 2.5 | 5 | 10 | 20 |

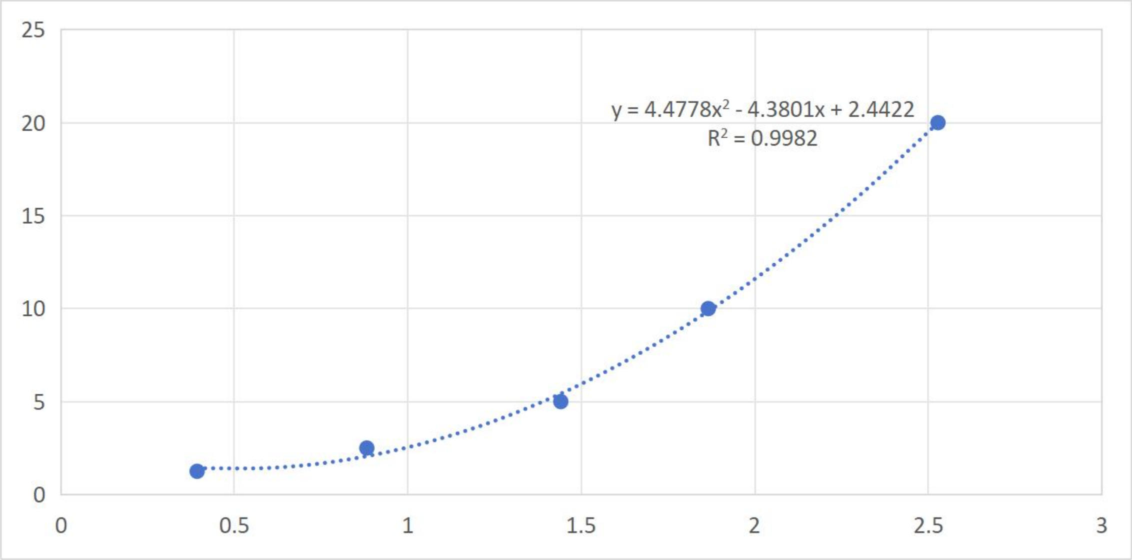


| Sample ID | OD (450n | Blank Cor | Dilution Fact | Concentration |
|---|---|---|---|---|
| 1 | 0.782 | 0.712 | 1.593562643 | 7.967813216 |
| 2 | 0.766 | 0.696 | 1.562768365 | 7.813841824 |
| 3 | 0.775 | 0.705 | 1.579808045 | 7.899040225 |
| 4 | 0.77 | 0.7 | 1.570252 | 7.85126 |
| 5 | 0.762 | 0.692 | 1.555428019 | 7.777140096 |
| 6 | 0.757 | 0.687 | 1.546454088 | 7.732270441 |
| 7 | 0.792 | 0.722 | 1.613973295 | 8.069866476 |
| 8 | 0.778 | 0.708 | 1.585649139 | 7.928245696 |
| 9 | 0.79 | 0.72 | 1.60981952 | 8.0490976 |
| 10 | 0.743 | 0.673 | 1.522518176 | 7.612590881 |
| 11 | 0.769 | 0.699 | 1.568367658 | 7.841838289 |
| 12 | 0.797 | 0.727 | 1.624514456 | 8.122572281 |
| 13 | 1.126 | 1.056 | 2.810170381 | 14.0508519 |
| 14 | 1.238 | 1.168 | 3.434965427 | 17.17482714 |
| 15 | 1.155 | 1.085 | 2.961169605 | 14.80584803 |
| 16 | 1.163 | 1.093 | 3.004149992 | 15.02074996 |
| 17 | 1.230 | 1.16 | 3.38661168 | 16.9330584 |
| 18 | 1.151 | 1.081 | 2.939894346 | 14.69947173 |
| 19 | 1.226 | 1.156 | 3.362649741 | 16.8132487 |
| 20 | 1.282 | 1.212 | 3.711156243 | 18.55578122 |
| 21 | 1.167 | 1.097 | 3.02585512 | 15.1292756 |
| 22 | 1.198 | 1.128 | 3.198928275 | 15.99464138 |
| 23 | 1.265 | 1.195 | 3.602390845 | 18.01195423 |
| 24 | 1.222 | 1.152 | 3.338831091 | 16.69415546 |
| 25 | 1.257 | 1.187 | 3.552102688 | 17.76051344 |
| 26 | 1.282 | 1.212 | 3.711156243 | 18.55578122 |
| 27 | 1.213 | 1.143 | 3.285763032 | 16.42881516 |
| 28 | 1.176 | 1.106 | 3.075215561 | 15.3760778 |
| 29 | 1.188 | 1.118 | 3.142157887 | 15.71078944 |
| 30 | 1.160 | 1.09 | 2.98796518 | 14.9398259 |
| 31 | 1.032 | 0.962 | 2.372496943 | 11.86248472 |

| | | | | |
|---|---|---|---|---|
| 32 | 1.057 | 0.987 | 2.481175248 | 12.40587624 |
| 33 | 1.011 | 0.941 | 2.285532722 | 11.42766361 |
| 34 | 1.083 | 1.013 | 2.600138248 | 13.00069124 |
| 35 | 1.045 | 0.975 | 2.428311125 | 12.14155563 |
| 36 | 1.104 | 1.034 | 2.700643337 | 13.50321668 |
| 37 | 1.118 | 1.048 | 2.769840851 | 13.84920426 |
| 38 | 1.066 | 0.996 | 2.521669645 | 12.60834822 |
| 39 | 0.998 | 0.928 | 2.233676915 | 11.16838458 |
| 40 | 1.015 | 0.945 | 2.301792845 | 11.50896423 |
| 41 | 1.004 | 0.934 | 2.257422297 | 11.28711148 |
| 42 | 1.038 | 0.968 | 2.398069267 | 11.99034634 |
| 43 | 1.077 | 1.007 | 2.572147912 | 12.86073956 |
| 44 | 0.952 | 0.882 | 2.062339887 | 10.31169944 |
| 45 | 1.033 | 0.963 | 2.376736608 | 11.88368304 |
| 46 | 1.061 | 0.991 | 2.499083202 | 12.49541601 |

| | | | | | |
|---|---|---|---|---|---|
| 0.066 | 0.457 | 0.96 | 1.528 | 1.944 | 2.621 |
| 0 | 0.391 | 0.894 | 1.462 | 1.878 | 2.555 |
| 0 | 1.25 | 2.5 | 5 | 10 | 20 |

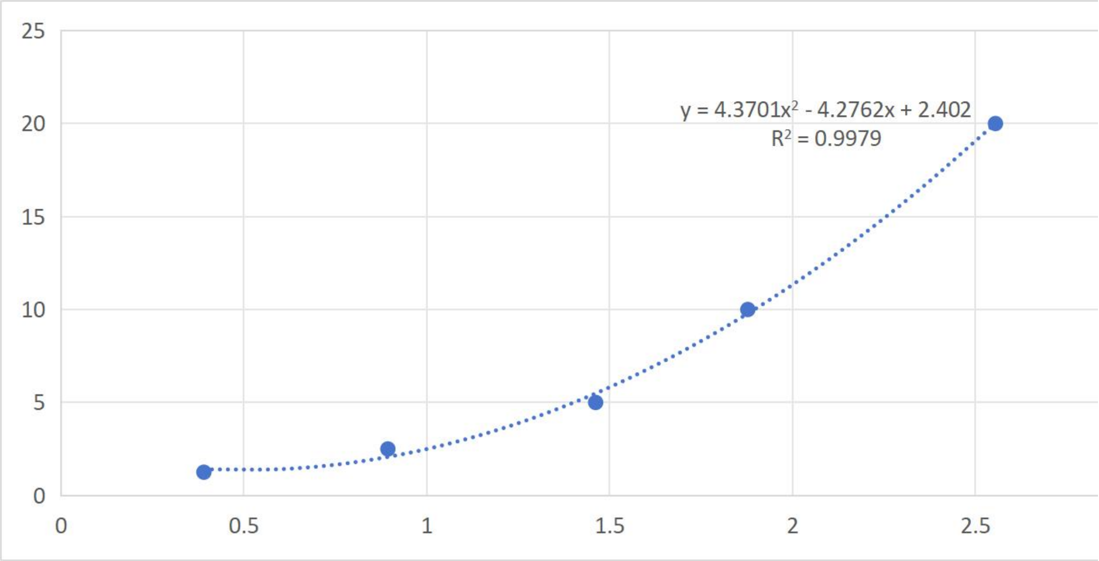


| Sample ID | OD (450n | Blank Cor | Dilution Fact | Concentration | |
|---|---|---|---|---|---|
| 1 | 0.782 | 0.716 | 1.580598786 | 7.902993928 | 15.87080714 |
| 2 | 0.766 | 0.7 | 1.550009 | 7.750045 | 15.56388682 |
| 3 | 0.775 | 0.709 | 1.566940438 | 7.834702191 | 15.73374242 |
| 4 | 0.77 | 0.704 | 1.557446682 | 7.787233408 | 15.63849341 |
| 5 | 0.762 | 0.696 | 1.542711162 | 7.713555808 | 15.4906959 |
| 6 | 0.757 | 0.691 | 1.533785518 | 7.668927591 | 15.40119803 |
| 7 | 0.792 | 0.726 | 1.600853628 | 8.004268138 | 16.07413461 |
| 8 | 0.778 | 0.712 | 1.572741574 | 7.863707872 | 15.79195357 |
| 9 | 0.79 | 0.724 | 1.596732738 | 7.983663688 | 16.03276129 |
| 10 | 0.743 | 0.677 | 1.509956163 | 7.549780815 | 15.1623717 |
| 11 | 0.769 | 0.703 | 1.555574151 | 7.777870755 | 15.61970904 |
| 12 | 0.797 | 0.731 | 1.611308806 | 8.056544031 | 16.17911631 |
| 13 | 1.126 | 1.06 | 2.77947236 | 13.8973618 | 27.9482137 |
| 14 | 1.238 | 1.172 | 3.392993038 | 16.96496519 | 34.13979233 |
| 15 | 1.155 | 1.089 | 2.927811562 | 14.63905781 | 29.44490584 |
| 16 | 1.163 | 1.097 | 2.970026271 | 14.85013135 | 29.87088132 |
| 17 | 1.230 | 1.164 | 3.34553421 | 16.72767105 | 33.66072945 |
| 18 | 1.151 | 1.085 | 2.906913973 | 14.53456986 | 29.23404159 |
| 19 | 1.226 | 1.16 | 3.32201456 | 16.6100728 | 33.4233215 |
| 20 | 1.282 | 1.216 | 3.664015386 | 18.32007693 | 36.87585814 |
| 21 | 1.167 | 1.101 | 2.99134339 | 14.95671695 | 30.08599255 |
| 22 | 1.198 | 1.132 | 3.161292622 | 15.80646311 | 31.80110449 |
| 23 | 1.265 | 1.199 | 3.55729633 | 17.78648165 | 35.79843588 |
| 24 | 1.222 | 1.156 | 3.298634754 | 16.49317377 | 33.18732922 |
| 25 | 1.257 | 1.191 | 3.507949618 | 17.53974809 | 35.30026153 |
| 26 | 1.282 | 1.216 | 3.664015386 | 18.32007693 | 36.87585814 |
| 27 | 1.213 | 1.147 | 3.246541491 | 16.23270745 | 32.66152262 |
| 28 | 1.176 | 1.11 | 3.03981821 | 15.19909105 | 30.57516885 |
| 29 | 1.188 | 1.122 | 3.105552568 | 15.52776284 | 31.23855228 |
| 30 | 1.160 | 1.094 | 2.954130204 | 14.77065102 | 29.71047692 |
| 31 | 1.032 | 0.966 | 2.349175836 | 11.74587918 | 23.60836389 |

| | | | | | |
|---|---|---|---|---|---|
| 32 | 1.057 | 0.991 | 2.456077978 | 12.28038989 | 24.68626613 |
| 33 | 1.011 | 0.945 | 2.263599553 | 11.31799776 | 22.74566137 |
| 34 | 1.083 | 1.017 | 2.573050959 | 12.86525479 | 25.86594604 |
| 35 | 1.045 | 0.979 | 2.404083214 | 12.02041607 | 24.1619717 |
| 36 | 1.104 | 1.038 | 2.671842424 | 13.35921212 | 26.86242881 |
| 37 | 1.118 | 1.052 | 2.73984475 | 13.69922375 | 27.54842801 |
| 38 | 1.066 | 1 | 2.4959 | 12.4795 | 25.08784822 |
| 39 | 0.998 | 0.932 | 2.212555342 | 11.06277671 | 22.23116129 |
| 40 | 1.015 | 0.949 | 2.27960263 | 11.39801315 | 22.90697738 |
| 41 | 1.004 | 0.938 | 2.235930664 | 11.17965332 | 22.46676481 |
| 42 | 1.038 | 0.972 | 2.374334158 | 11.87167079 | 23.86201713 |
| 43 | 1.077 | 1.011 | 2.545532782 | 12.72766391 | 25.58840347 |
| 44 | 0.952 | 0.886 | 2.04379782 | 10.2189891 | 20.53068853 |
| 45 | 1.033 | 0.967 | 2.353347039 | 11.76673519 | 23.65041824 |
| 46 | 1.061 | 0.995 | 2.473689253 | 12.36844626 | 24.86386227 |

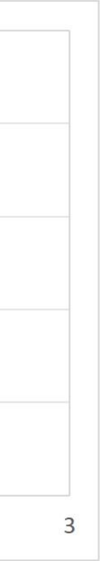


7.935403572
7.781943412
7.866871208
7.819246704
7.745347952
7.700599016
8.037067307
7.895976784
8.016380644
7.581185848
7.809854522
8.089558156
13.97410685
17.06989616
14.72245292
14.93544066
16.83036472
14.6170208
16.71166075
18.43792907
15.04299628
15.90055224
17.89921794
16.59366461
17.65013077
18.43792907
16.33076131
15.28758443
15.61927614
14.85523846
11.80418195

| |
|---|
| 12.34313307 |
| 11.37283069 |
| 12.93297302 |
| 12.08098585 |
| 13.4312144 |
| 13.774214 |
| 12.54392411 |
| 11.11558064 |
| 11.45348869 |
| 11.2333824 |
| 11.93100856 |
| 12.79420174 |
| 10.26534427 |
| 11.82520912 |
| 12.43193114 |

| | | | | | |
|---|---|---|---|---|---|
| 0.078 | 0.506 | 0.994 | 1.52 | 1.991 | 2.652 |
| 0 | 0.428 | 0.916 | 1.442 | 1.913 | 2.574 |
| 0 | 1.25 | 2.5 | 5 | 10 | 20 |

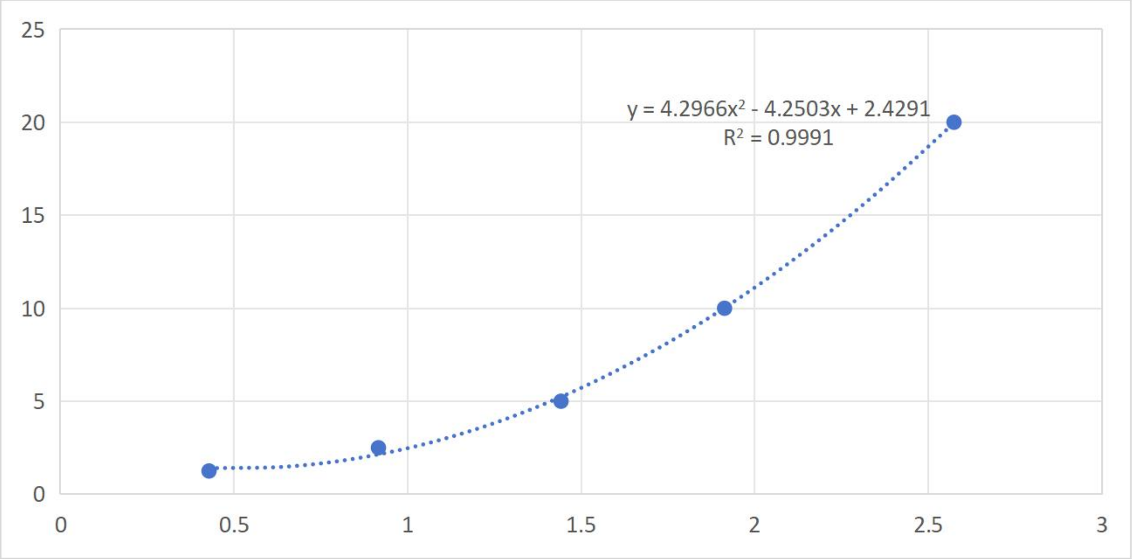


| Sample ID | OD （450n | Blank Cor | Dilution Fact | Concentration |
|---|---|---|---|---|
| 1 | 0.774 | 0.696 | 1.552232986 | 7.761164928 |
| 2 | 0.732 | 0.654 | 1.487128366 | 7.435641828 |
| 3 | 0.768 | 0.69 | 1.54200426 | 7.7100213 |
| 4 | 0.75 | 0.672 | 1.513174214 | 7.565871072 |
| 5 | 0.806 | 0.728 | 1.612010854 | 8.060054272 |
| 6 | 0.787 | 0.709 | 1.575456485 | 7.877282423 |
| 7 | 0.799 | 0.721 | 1.598182541 | 7.990912703 |
| 8 | 0.761 | 0.683 | 1.530461737 | 7.652308687 |
| 9 | 0.810 | 0.732 | 1.620101798 | 8.100508992 |
| 10 | 0.762 | 0.684 | 1.53208489 | 7.660424448 |
| 11 | 0.753 | 0.675 | 1.517785875 | 7.588929375 |
| 12 | 0.817 | 0.739 | 1.634591789 | 8.172958943 |
| 13 | 1.213 | 1.135 | 3.139997035 | 15.69998518 |
| 14 | 1.242 | 1.164 | 3.303196954 | 16.51598477 |
| 15 | 1.225 | 1.147 | 3.206651529 | 16.03325765 |
| 16 | 1.203 | 1.125 | 3.085396875 | 15.42698438 |
| 17 | 1.134 | 1.056 | 2.732076538 | 13.66038269 |
| 18 | 1.201 | 1.123 | 3.074579961 | 15.37289981 |
| 19 | 1.209 | 1.131 | 3.118053853 | 15.59026926 |
| 20 | 1.293 | 1.215 | 3.607733835 | 18.03866918 |
| 21 | 1.267 | 1.189 | 3.449686949 | 17.24843474 |
| 22 | 1.224 | 1.146 | 3.201049726 | 16.00524863 |
| 23 | 1.253 | 1.175 | 3.366990875 | 16.83495438 |
| 24 | 1.241 | 1.163 | 3.297449065 | 16.48724533 |
| 25 | 1.196 | 1.118 | 3.047688058 | 15.23844029 |
| 26 | 1.302 | 1.224 | 3.663795802 | 18.31897901 |
| 27 | 1.263 | 1.185 | 3.425887635 | 17.12943818 |
| 28 | 1.157 | 1.079 | 2.845304181 | 14.2265209 |
| 29 | 1.228 | 1.15 | 3.2235085 | 16.1175425 |
| 30 | 1.217 | 1.139 | 3.162077709 | 15.81038854 |
| 31 | 1.056 | 0.978 | 2.381935754 | 11.90967877 |
| 32 | 1.042 | 0.964 | 2.324623994 | 11.62311997 |

| | | | | |
|---|---|---|---|---|
| 33 | 1.038 | 0.96 | 2.30855856 | 11.5427928 |
| 34 | 1.110 | 1.032 | 2.618772518 | 13.09386259 |
| 35 | 1.125 | 1.047 | 2.689007489 | 13.44503745 |
| 36 | 1.113 | 1.035 | 2.632664835 | 13.16332418 |
| 37 | 1.104 | 1.026 | 2.591219902 | 12.95609951 |
| 38 | 0.966 | 0.888 | 2.04289175 | 10.21445875 |
| 39 | 0.972 | 0.894 | 2.063329198 | 10.31664599 |
| 40 | 1.002 | 0.924 | 2.170156762 | 10.85078381 |
| 41 | 1.013 | 0.935 | 2.211264635 | 11.05632318 |
| 42 | 1.062 | 0.984 | 2.40701353 | 12.03506765 |
| 43 | 1.050 | 0.972 | 2.357167334 | 11.78583667 |
| 44 | 0.973 | 0.895 | 2.066765515 | 10.33382758 |
| 45 | 1.133 | 1.055 | 2.727256715 | 13.63628358 |
| 46 | 1.121 | 1.043 | 2.670089113 | 13.35044557 |

| | | | | | |
|---|---|---|---|---|---|
| 0.074 | 0.498 | 0.987 | 1.502 | 1.943 | 2.647 |
| 0 | 0.424 | 0.913 | 1.428 | 1.869 | 2.573 |
| 0 | 1.25 | 2.5 | 5 | 10 | 20 |

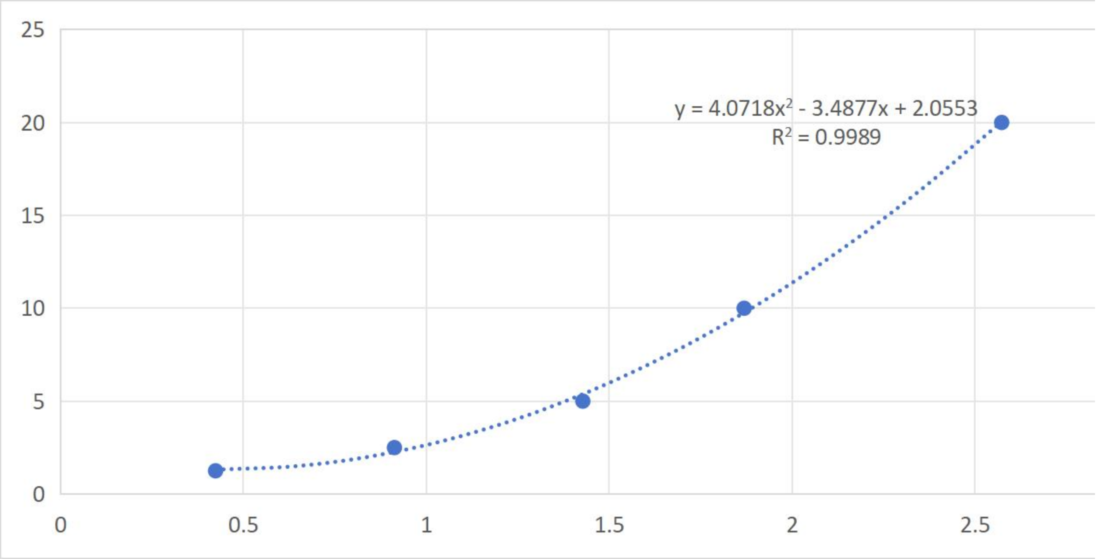


| Sample ID | OD （450n | Blank Cor | Dilution Fact | Concentration | |
|---|---|---|---|---|---|
| 1 | 0.774 | 0.7 | 1.609092 | 8.04546 | 15.80662493 |
| 2 | 0.732 | 0.658 | 1.523336215 | 7.616681076 | 15.0523229 |
| 3 | 0.768 | 0.694 | 1.595961665 | 7.979808324 | 15.68982962 |
| 4 | 0.75 | 0.676 | 1.558329677 | 7.791648384 | 15.35751946 |
| 5 | 0.806 | 0.732 | 1.684071763 | 8.420358816 | 16.48041309 |
| 6 | 0.787 | 0.713 | 1.638546794 | 8.192733971 | 16.07001639 |
| 7 | 0.799 | 0.725 | 1.666957375 | 8.334786875 | 16.32569958 |
| 8 | 0.761 | 0.687 | 1.581013474 | 7.905067371 | 15.55737606 |
| 9 | 0.810 | 0.736 | 1.694030573 | 8.470152864 | 16.57066186 |
| 10 | 0.762 | 0.688 | 1.583124499 | 7.915622496 | 15.57604694 |
| 11 | 0.753 | 0.679 | 1.564418444 | 7.822092219 | 15.41102159 |
| 12 | 0.817 | 0.743 | 1.711772018 | 8.558860091 | 16.73181903 |
| 13 | 1.213 | 1.139 | 3.365241348 | 16.82620674 | 32.52619191 |
| 14 | 1.242 | 1.168 | 3.536513683 | 17.68256842 | 34.19855318 |
| 15 | 1.225 | 1.151 | 3.435282012 | 17.17641006 | 33.20966771 |
| 16 | 1.203 | 1.129 | 3.307769924 | 16.53884962 | 31.96583399 |
| 17 | 1.134 | 1.06 | 2.93341248 | 14.6670624 | 28.32744509 |
| 18 | 1.201 | 1.127 | 3.296373362 | 16.48186681 | 31.85476662 |
| 19 | 1.209 | 1.135 | 3.342155055 | 16.71077528 | 32.30104454 |
| 20 | 1.293 | 1.219 | 3.8543297 | 19.2716485 | 37.31031767 |
| 21 | 1.267 | 1.193 | 3.689659178 | 18.44829589 | 35.69673063 |
| 22 | 1.224 | 1.15 | 3.4294005 | 17.1470025 | 33.15225113 |
| 23 | 1.253 | 1.179 | 3.603270644 | 18.01635322 | 34.85130759 |
| 24 | 1.241 | 1.167 | 3.53049373 | 17.65246865 | 34.13971398 |
| 25 | 1.196 | 1.122 | 3.268024471 | 16.34012236 | 31.57856265 |
| 26 | 1.302 | 1.228 | 3.912613651 | 19.56306826 | 37.88204726 |
| 27 | 1.263 | 1.189 | 3.664813868 | 18.32406934 | 35.45350751 |
| 28 | 1.157 | 1.083 | 3.05389033 | 15.26945165 | 29.49597255 |
| 29 | 1.228 | 1.154 | 3.452975409 | 17.26487704 | 33.38241954 |
| 30 | 1.217 | 1.143 | 3.388457938 | 16.94228969 | 32.75267823 |
| 31 | 1.056 | 0.982 | 2.556913063 | 12.78456532 | 24.69424409 |
| 32 | 1.042 | 0.968 | 2.494580723 | 12.47290362 | 24.09602358 |

| | | | | | |
|---|---|---|---|---|---|
| 33 | 1.038 | 0.964 | 2.477064653 | 12.38532326 | 23.92811606 |
| 34 | 1.110 | 1.036 | 2.812289453 | 14.06144726 | 27.15530986 |
| 35 | 1.125 | 1.051 | 2.887441652 | 14.43720826 | 27.88224571 |
| 36 | 1.113 | 1.039 | 2.827173308 | 14.13586654 | 27.29919071 |
| 37 | 1.104 | 1.03 | 2.78274162 | 13.9137081 | 26.86980761 |
| 38 | 0.966 | 0.892 | 2.184056275 | 10.92028138 | 21.13474013 |
| 39 | 0.972 | 0.898 | 2.206861207 | 11.03430604 | 21.35095202 |
| 40 | 1.002 | 0.928 | 2.325283411 | 11.62641706 | 22.47720086 |
| 41 | 1.013 | 0.939 | 2.370541268 | 11.85270634 | 22.90902951 |
| 42 | 1.062 | 0.988 | 2.584115539 | 12.9205777 | 24.95564534 |
| 43 | 1.050 | 0.976 | 2.530003757 | 12.65001878 | 24.43585546 |
| 44 | 0.973 | 0.899 | 2.210690532 | 11.05345266 | 21.38728023 |
| 45 | 1.133 | 1.059 | 2.928272036 | 14.64136018 | 28.27764375 |
| 46 | 1.121 | 1.047 | 2.867221906 | 14.33610953 | 27.6865551 |

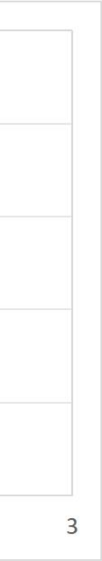


| |
|---|
| 7.903312464 |
| 7.526161452 |
| 7.844914812 |
| 7.678759728 |
| 8.240206544 |
| 8.035008197 |
| 8.162849789 |
| 7.778688029 |
| 8.285330928 |
| 7.788023472 |
| 7.705510797 |
| 8.365909517 |
| 16.26309596 |
| 17.09927659 |
| 16.60483385 |
| 15.982917 |
| 14.16372254 |
| 15.92738331 |
| 16.15052227 |
| 18.65515884 |
| 17.84836532 |
| 16.57612556 |
| 17.4256538 |
| 17.06985699 |
| 15.78928132 |
| 18.94102363 |
| 17.72675376 |
| 14.74798628 |
| 16.69120977 |
| 16.37633912 |
| 12.34712204 |
| 12.04801179 |

| |
|---|
| 11.96405803 |
| 13.57765493 |
| 13.94112285 |
| 13.64959536 |
| 13.4349038 |
| 10.56737006 |
| 10.67547601 |
| 11.23860043 |
| 11.45451476 |
| 12.47782267 |
| 12.21792773 |
| 10.69364012 |
| 14.13882188 |
| 13.84327755 |

| | | | | | |
|---|---|---|---|---|---|
| 0.073 | 0.561 | 1.054 | 1.599 | 2.036 | 2.734 |
| 0 | 0.488 | 0.981 | 1.526 | 1.963 | 2.661 |
| 0 | 1.25 | 2.5 | 5 | 10 | 20 |

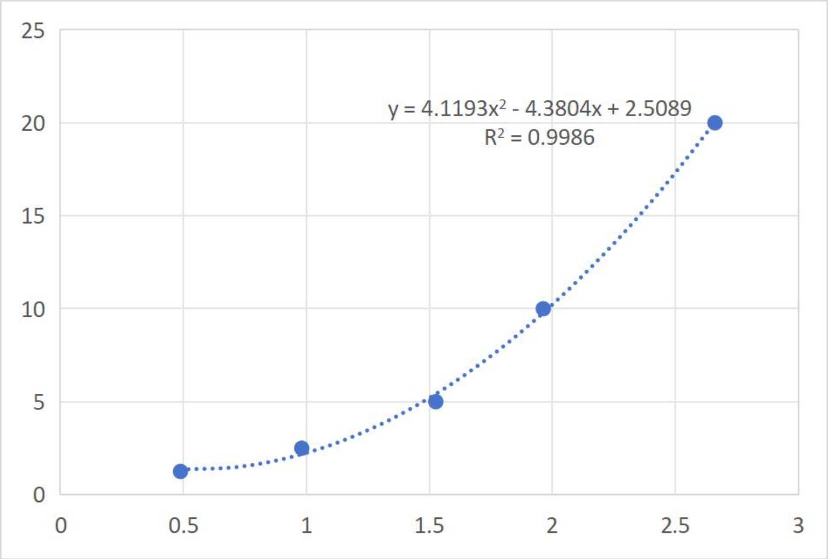


| Sample ID | OD （450n | Blank Cor | Dilution | Concentration |
|---|---|---|---|---|
| 1 | 0.804 | 0.731 | 1.508021 | 7.5401043 |
| 2 | 0.821 | 0.748 | 1.537126 | 7.6856281 |
| 3 | 0.768 | 0.695 | 1.454247 | 7.2712344 |
| 4 | 0.853 | 0.78 | 1.59837 | 7.9918506 |
| 5 | 0.822 | 0.749 | 1.538912 | 7.6945591 |
| 6 | 0.799 | 0.726 | 1.499914 | 7.4995688 |
| 7 | 0.821 | 0.748 | 1.537126 | 7.6856281 |
| 8 | 0.833 | 0.76 | 1.559104 | 7.7955184 |
| 9 | 0.809 | 0.736 | 1.516334 | 7.5816697 |
| 10 | 0.796 | 0.723 | 1.495148 | 7.4757418 |
| 11 | 0.837 | 0.764 | 1.566693 | 7.8334667 |
| 12 | 0.841 | 0.768 | 1.574415 | 7.872074 |
| 13 | 1.303 | 1.23 | 3.353097 | 16.765485 |
| 14 | 1.312 | 1.239 | 3.405208 | 17.026042 |
| 15 | 1.298 | 1.225 | 3.324435 | 16.622173 |
| 16 | 1.309 | 1.236 | 3.387764 | 16.938819 |
| 17 | 1.264 | 1.191 | 3.134992 | 15.674962 |
| 18 | 1.241 | 1.168 | 3.012241 | 15.061204 |
| 19 | 1.289 | 1.216 | 3.273361 | 16.366806 |
| 20 | 1.317 | 1.244 | 3.434447 | 17.172237 |
| 21 | 1.283 | 1.21 | 3.239683 | 16.198416 |
| 22 | 1.274 | 1.201 | 3.189722 | 15.94861 |
| 23 | 1.288 | 1.215 | 3.267728 | 16.338638 |
| 24 | 1.239 | 1.166 | 3.001773 | 15.008863 |
| 25 | 1.266 | 1.193 | 3.145872 | 15.729362 |
| 26 | 1.313 | 1.24 | 3.41104 | 17.055198 |
| 27 | 1.283 | 1.21 | 3.239683 | 16.198416 |
| 28 | 1.249 | 1.176 | 3.054443 | 15.272213 |
| 29 | 1.255 | 1.182 | 3.08644 | 15.4322 |
| 30 | 1.271 | 1.198 | 3.173217 | 15.866083 |
| 31 | 1.094 | 1.021 | 2.330639 | 11.653194 |
| 32 | 1.073 | 1 | 2.2478 | 11.239 |

| | | | | |
|---|---|---|---|---|
| 33 | 1.065 | 0.992 | 2.217198 | 11.08599 |
| 34 | 1.121 | 1.048 | 2.442484 | 12.212422 |
| 35 | 1.132 | 1.059 | 2.489773 | 12.448865 |
| 36 | 1.147 | 1.074 | 2.555864 | 12.77932 |
| 37 | 1.151 | 1.078 | 2.573801 | 12.869007 |
| 38 | 1.066 | 0.993 | 2.220994 | 11.104972 |
| 39 | 1.072 | 0.999 | 2.243946 | 11.21973 |
| 40 | 1.102 | 1.029 | 2.363152 | 11.815761 |
| 41 | 1.122 | 1.049 | 2.446742 | 12.233711 |
| 42 | 1.145 | 1.072 | 2.546945 | 12.734724 |
| 43 | 1.023 | 0.95 | 2.065188 | 10.325941 |
| 44 | 0.994 | 0.921 | 1.968711 | 9.8435538 |
| 45 | 1.109 | 1.036 | 2.392034 | 11.960169 |
| 46 | 1.139 | 1.066 | 2.520385 | 12.601924 |

| | | | | | |
|---|---|---|---|---|---|
| 0.069 | 0.541 | 1.039 | 1.621 | 2.036 | 2.667 |
| 0 | 0.472 | 0.97 | 1.552 | 1.967 | 2.598 |
| 0 | 1.25 | 2.5 | 5 | 10 | 20 |

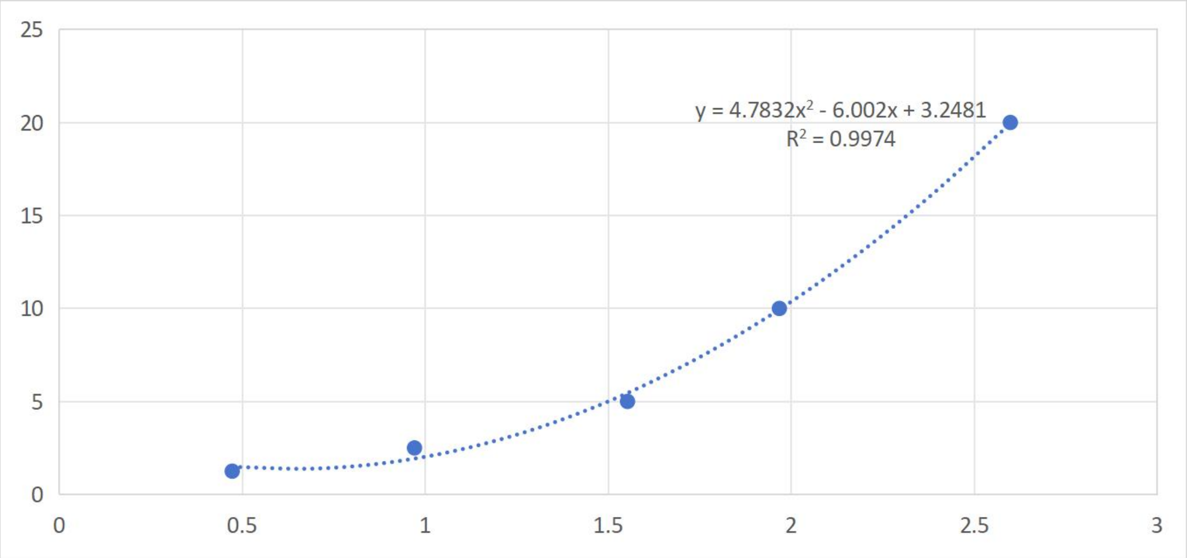


| Sample ID | OD (450n | Blank Cor | Dilution Fact | Concentration | | |
|---|---|---|---|---|---|---|
| 1 | 0.804 | 0.735 | 1.42063422 | 7.1031711 | 14.64327544 | 7.321637718 |
| 2 | 0.821 | 0.752 | 1.439514733 | 7.197573664 | 14.8832018 | 7.4416009 |
| 3 | 0.768 | 0.699 | 1.389778303 | 6.948891516 | 14.22012593 | 7.110062964 |
| 4 | 0.853 | 0.784 | 1.482554579 | 7.412772896 | 15.4046235 | 7.702311748 |
| 5 | 0.822 | 0.753 | 1.440711449 | 7.203557244 | 14.89811634 | 7.44905817 |
| 6 | 0.799 | 0.73 | 1.41560728 | 7.0780364 | 14.57760523 | 7.288802617 |
| 7 | 0.821 | 0.752 | 1.439514733 | 7.197573664 | 14.8832018 | 7.4416009 |
| 8 | 0.833 | 0.764 | 1.454506707 | 7.272533536 | 15.06805194 | 7.534025968 |
| 9 | 0.809 | 0.74 | 1.42590032 | 7.1295016 | 14.71117126 | 7.355585632 |
| 10 | 0.796 | 0.727 | 1.412705913 | 7.063529564 | 14.53927141 | 7.269635706 |
| 11 | 0.837 | 0.768 | 1.459810157 | 7.299050784 | 15.13251745 | 7.566258724 |
| 12 | 0.841 | 0.772 | 1.465266669 | 7.326333344 | 15.19840736 | 7.59920368 |
| 13 | 1.303 | 1.234 | 3.125278499 | 15.6263925 | 32.39187735 | 16.19593867 |
| 14 | 1.312 | 1.243 | 3.177892377 | 15.88946188 | 32.91550356 | 16.45775178 |
| 15 | 1.298 | 1.229 | 3.096383391 | 15.48191696 | 32.10408977 | 16.05204488 |
| 16 | 1.309 | 1.24 | 3.16026832 | 15.8013416 | 32.74016026 | 16.37008013 |
| 17 | 1.264 | 1.195 | 2.90623918 | 14.5311959 | 30.20615782 | 15.10307891 |
| 18 | 1.241 | 1.172 | 2.783882989 | 13.91941494 | 28.98061856 | 14.49030928 |
| 19 | 1.289 | 1.22 | 3.04497488 | 15.2248744 | 31.5916807 | 15.79584035 |
| 20 | 1.317 | 1.248 | 3.207457133 | 16.03728566 | 33.20952289 | 16.60476144 |
| 21 | 1.283 | 1.214 | 3.011133027 | 15.05566514 | 31.25408079 | 15.62704039 |
| 22 | 1.274 | 1.205 | 2.96101598 | 14.8050799 | 30.7536901 | 15.37684505 |
| 23 | 1.288 | 1.219 | 3.039310655 | 15.19655328 | 31.53519149 | 15.76759574 |
| 24 | 1.239 | 1.17 | 2.77348248 | 13.8674124 | 28.87627555 | 14.43813778 |
| 25 | 1.266 | 1.197 | 2.917118009 | 14.58559004 | 30.31495207 | 15.15747604 |
| 26 | 1.313 | 1.244 | 3.183786195 | 15.91893098 | 32.97412938 | 16.48706469 |
| 27 | 1.283 | 1.214 | 3.011133027 | 15.05566514 | 31.25408079 | 15.62704039 |
| 28 | 1.249 | 1.18 | 2.82586768 | 14.1293384 | 29.40155158 | 14.70077579 |
| 29 | 1.255 | 1.186 | 2.857757987 | 14.28878994 | 29.7209904 | 14.8604952 |
| 30 | 1.271 | 1.202 | 2.944482493 | 14.72241246 | 30.58849565 | 15.29424783 |
| 31 | 1.094 | 1.025 | 2.1213995 | 10.6069975 | 22.26019156 | 11.13009578 |
| 32 | 1.073 | 1.004 | 2.043634131 | 10.21817066 | 21.45717066 | 10.72858533 |

| | | | | | | |
|---|---|---|---|---|---|---|
| 33 | 1.065 | 0.996 | 2.015118931 | 10.07559466 | 21.16158483 | 10.58079242 |
| 34 | 1.121 | 1.052 | 2.227582573 | 11.13791286 | 23.3503352 | 11.6751676 |
| 35 | 1.132 | 1.063 | 2.272841721 | 11.3642086 | 23.81307402 | 11.90653701 |
| 36 | 1.147 | 1.078 | 2.336424189 | 11.68212094 | 24.46144138 | 12.23072069 |
| 37 | 1.151 | 1.082 | 2.353743037 | 11.76871518 | 24.63772229 | 12.31886115 |
| 38 | 1.066 | 0.997 | 2.018649849 | 10.09324924 | 21.19822147 | 10.59911074 |
| 39 | 1.072 | 1.003 | 2.040036249 | 10.20018124 | 21.41991084 | 10.70995542 |
| 40 | 1.102 | 1.033 | 2.152134105 | 10.76067052 | 22.57643118 | 11.28821559 |
| 41 | 1.122 | 1.053 | 2.231649209 | 11.15824604 | 23.39195724 | 11.69597862 |
| 42 | 1.145 | 1.076 | 2.327822163 | 11.63911082 | 24.37383507 | 12.18691754 |
| 43 | 1.023 | 0.954 | 1.875458851 | 9.377294256 | 19.70323551 | 9.851617753 |
| 44 | 0.994 | 0.925 | 1.7888755 | 8.9443775 | 18.78793126 | 9.393965628 |
| 45 | 1.109 | 1.04 | 2.17952912 | 10.8976456 | 22.85781466 | 11.42890733 |
| 46 | 1.139 | 1.07 | 2.30224568 | 11.5112284 | 24.11315275 | 12.05657638 |

| | | | |
|---|---|---|---|
| 7.935404 | 7.903312 | 7.321638 | 7.720117918 |
| 7.781943 | 7.526161 | 7.441601 | 7.583235255 |
| 7.866871 | 7.844915 | 7.110063 | 7.607282995 |
| 7.819247 | 7.67876 | 7.702312 | 7.733439393 |
| 7.745348 | 8.240207 | 7.449058 | 7.811537555 |
| 7.700599 | 8.035008 | 7.288803 | 7.674803277 |
| 8.037067 | 8.16285 | 7.441601 | 7.880505999 |
| 7.895977 | 7.778688 | 7.534026 | 7.73623026 |
| 8.016381 | 8.285331 | 7.355586 | 7.885765735 |
| 7.581186 | 7.788023 | 7.269636 | 7.546281675 |
| 7.809855 | 7.705511 | 7.566259 | 7.693874681 |
| 8.089558 | 8.36591 | 7.599204 | 8.018223784 |
| 13.97411 | 16.2631 | 16.19594 | 15.47771383 |
| 17.0699 | 17.09928 | 16.45775 | 16.87564151 |
| 14.72245 | 16.60483 | 16.05204 | 15.79311055 |
| 14.93544 | 15.98292 | 16.37008 | 15.7628126 |
| 16.83036 | 14.16372 | 15.10308 | 15.36572206 |
| 14.61702 | 15.92738 | 14.49031 | 15.01157113 |
| 16.71166 | 16.15052 | 15.79584 | 16.21934112 |
| 18.43793 | 18.65516 | 16.60476 | 17.89928312 |
| 15.043 | 17.84837 | 15.62704 | 16.17280066 |
| 15.90055 | 16.57613 | 15.37685 | 15.95117428 |
| 17.89922 | 17.42565 | 15.7676 | 17.03082249 |
| 16.59366 | 17.06986 | 14.43814 | 16.03388646 |
| 17.65013 | 15.78928 | 15.15748 | 16.19896271 |
| 18.43793 | 18.94102 | 16.48706 | 17.95533913 |
| 16.33076 | 17.72675 | 15.62704 | 16.56151849 |
| 15.28758 | 14.74799 | 14.70078 | 14.9121155 |
| 15.61928 | 16.69121 | 14.8605 | 15.72366037 |
| 14.85524 | 16.37634 | 15.29425 | 15.50860847 |
| 11.80418 | 12.34712 | 11.1301 | 11.76046659 |
| 12.34313 | 12.04801 | 10.72859 | 11.70657673 |
| 11.37283 | 11.96406 | 10.58079 | 11.30589371 |
| 12.93297 | 13.57765 | 11.67517 | 12.72859852 |
| 12.08099 | 13.94112 | 11.90654 | 12.6428819 |
| 13.43121 | 13.6496 | 12.23072 | 13.10384348 |
| 13.77421 | 13.4349 | 12.31886 | 13.17599298 |
| 12.54392 | 10.56737 | 10.59911 | 11.23680164 |
| 11.11558 | 10.67548 | 10.70996 | 10.83367069 |
| 11.45349 | 11.2386 | 11.28822 | 11.32676824 |
| 11.23338 | 11.45451 | 11.69598 | 11.46129193 |
| 11.93101 | 12.47782 | 12.18692 | 12.19858292 |
| 12.7942 | 12.21793 | 9.851618 | 11.62124907 |
| 10.26534 | 10.69364 | 9.393966 | 10.11765001 |
| 11.82521 | 14.13882 | 11.42891 | 12.46431278 |
| 12.43193 | 13.84328 | 12.05658 | 12.77726169 |

Report
TSH（90）

| Group | Mean | Std. Deviation |
|---|---|---|
| 1 | 7.740942 | .1381868065395 |
| 2 | 16.13633[illegible] | .8614025334787 |
| 3 | 11.90386[illegible] | .8662202805163 |
| Total | 12.47407[illegible] | 3.4625978776952 |

Tests of Normality

| | Group | Kolmogorov-Smirnova | | | Shapiro-Wilk | | |
|---|---|---|---|---|---|---|---|
| | | Statisti[illegible] | df | Sig. | Statisti[illegible] | df | Sig. |
| TSH (90) | 1 | 0.18 | 12 | .200* | 0.96 | 12 | 0.786 |
| | 2 | 0.184 | 18 | 0.11 | 0.926 | 18 | 0.166 |
| | 3 | 0.128 | 16 | .200* | 0.958 | 16 | 0.633 |

* This is a lower bound of the true significance.

a Lilliefors Significance Correction

ANOVA

TSH (90)

| | Sum of S[illegible] | df | Mean Squ[illegible] | F | Sig. |
|---|---|---|---|---|---|
| Between ( | 515.452 | 2 | 257.726 | 460.237 | 0 |
| Within G[illegible] | 24.079 | 43 | 0.56 | | |
| Total | 539.531 | 45 | | | |

Robust Tests of Equality of Means

TSH (90)

| | Statisti[illegible] | df1 | df2 | Sig. |
|---|---|---|---|---|
| Welch | 948.346 | 2 | 22.676 | 0 |

a Asymptotically F distributed.

Multiple Comparisons

Dependent Variable: TSH (90)

Games-Howell

| (I) Grou[illegible] | (J) Grou[illegible] | Mean Dif[illegible] | Std. Err[illegible] | Sig. | 95% Confidence Interval | |
|---|---|---|---|---|---|---|
| | | | | | Lower Bo[illegible] | Upper Bound |
| 1 | 2 | -8.39539[illegible] | .2069162[illegible] | 0 | -8.92273 | -7.86806 |
| | 3 | -4.16292[illegible] | .2201985[illegible] | 0 | -4.73107 | -3.59477 |
| 2 | 1 | 8.395396[illegible] | .2069162[illegible] | 0 | 7.868059 | 8.922734 |
| | 3 | 4.232472[illegible] | .2968486[illegible] | 0 | 3.502438 | 4.962508 |
| 3 | 1 | 4.162923[illegible] | .2201985[illegible] | 0 | 3.594774 | 4.731073 |
| | 2 | -4.23247[illegible] | .2968486[illegible] | 0 | -4.96251 | -3.50244 |

* The mean difference is significant at the 0.05 level.

| | 1 | 2 | 3 | 4 | 5 | 6 | 7 | 8 |
|---|---|---|---|---|---|---|---|---|
| A | UD | UD | UD | UD | UD | UD | UD | UD |
| B | UD | UD | UD | UD | UD | UD | UD | UD |
| C | UD | UD | UD | UD | UD | UD | UD | UD |
| D | UD | UD | UD | UD | UD | UD | UD | UD |
| E | UD | UD | UD | UD | UD | UD | UD | UD |
| F | UD | UD | UD | UD | UD | UD | UD | UD |
| G | UD | UD | UD | UD | UD | UD | UD | UD |
| H | UD | UD | UD | UD | UD | UD | UD | UD |

Absorbance Data:
Filter1:450nm

Reading: 1

| | 1 | 2 | 3 | 4 | 5 | 6 | 7 | 8 |
|---|---|---|---|---|---|---|---|---|
| A | 0.215 | 0.227 | 0.208 | 0.216 | 0.212 | 0.215 | 0.229 | 0.202 |
| B | 0.223 | 0.244 | 0.210 | 0.236 | 0.229 | 0.234 | 0.260 | 0.222 |
| C | 0.244 | 0.249 | 0.232 | 0.245 | 0.245 | 0.245 | 0.254 | 0.218 |
| D | 0.190 | 0.211 | 0.187 | 0.195 | 0.216 | 0.226 | 0.224 | 0.217 |
| E | 0.220 | 0.232 | 0.200 | 0.218 | 0.203 | 0.202 | 0.235 | 0.207 |
| F | 0.207 | 0.219 | 0.199 | 0.207 | 0.195 | 0.215 | 0.232 | 0.210 |
| G | 0.228 | 0.236 | 0.198 | 0.217 | 0.220 | 0.230 | 0.231 | 0.215 |
| H | 0.195 | 0.208 | 0.184 | 0.192 | 0.193 | 0.232 | 0.189 | 0.108 |

Quantitative Analysis Results (Concentration)
Filter1: 450nm

| | 1 | 2 | 3 | 4 | 5 | 6 | 7 | 8 |
|---|---|---|---|---|---|---|---|---|
| A | 0 | 0 | 0 | 0 | 0 | 0 | 0 | 0 |
| B | 0 | 0 | 0 | 0 | 0 | 0 | 0 | 0 |
| C | 0 | 0 | 0 | 0 | 0 | 0 | 0 | 0 |
| D | 0 | 0 | 0 | 0 | 0 | 0 | 0 | 0 |
| E | 0 | 0 | 0 | 0 | 0 | 0 | 0 | 0 |
| F | 0 | 0 | 0 | 0 | 0 | 0 | 0 | 0 |
| G | 0 | 0 | 0 | 0 | 0 | 0 | 0 | 0 |
| H | 0 | 0 | 0 | 0 | 0 | 0 | 0 | 0 |

| 9 | 10 | 11 | 12 |
|---|---|---|---|
| UD | UD | UD | UD |
| UD | UD | UD | UD |
| UD | UD | UD | UD |
| UD | UD | UD | UD |
| UD | UD | UD | UD |
| UD | UD | UD | UD |
| UD | UD | UD | UD |

| 9 | 10 | 11 | 12 |
|---|---|---|---|
| 0.210 | 0.196 | 0.195 | 0.209 |
| 0.226 | 0.224 | 0.236 | 0.242 |
| 0.242 | 0.225 | 0.228 | 0.225 |
| 0.215 | 0.221 | 0.209 | 0.229 |
| 0.202 | 0.203 | 0.195 | 0.213 |
| 0.210 | 0.202 | 0.201 | 0.218 |
| 0.228 | 0.221 | 0.224 | 0.231 |
| | | | |

| 9 | 10 | 11 | 12 |
|---|---|---|---|
| 0 | 0 | 0 | 0 |
| 0 | 0 | 0 | 0 |
| 0 | 0 | 0 | 0 |
| 0 | 0 | 0 | 0 |
| 0 | 0 | 0 | 0 |
| 0 | 0 | 0 | 0 |
| 0 | 0 | 0 | 0 |

| | 1 | 2 | 3 | 4 | 5 | 6 | 7 | 8 |
|---|---|---|---|---|---|---|---|---|
| A | 3.302469 | 3.67284 | 3.08642 | 3.333333 | 3.209877 | 3.302469 | 3.734568 | 2.901235 |
| B | 3.549383 | 4.197531 | 3.148148 | 3.950617 | 3.734568 | 3.888889 | 4.691358 | 3.518519 |
| C | 4.197531 | 4.351852 | 3.82716 | 4.228395 | 4.228395 | 4.228395 | 4.506173 | 3.395062 |
| D | 2.530864 | 3.179012 | 2.438272 | 2.685185 | 3.333333 | 3.641975 | 3.580247 | 3.364198 |
| E | 3.45679 | 3.82716 | 2.839506 | 3.395062 | 2.932099 | 2.901235 | 3.919753 | 3.055556 |
| F | 3.055556 | 3.425926 | 2.808642 | 3.055556 | 2.685185 | 3.302469 | 3.82716 | 3.148148 |
| G | 3.703704 | 3.950617 | 2.777778 | 3.364198 | 3.45679 | 3.765432 | 3.796296 | 3.302469 |
| H | 2.685185 | 3.08642 | 2.345679 | 2.592593 | 2.623457 | 3.82716 | 2.5 | 0 |

| W-Sham group | | | W-SI group | | | W-SII group | | |
|---|---|---|---|---|---|---|---|---|
| 3.302469 | 3.67284 | 3.08642 | 4.506173 | 3.395062 | 4.135802 | 3.055556 | 3.425926 | 2.808642 |
| 3.333333 | 3.209877 | 3.302469 | 3.611111 | 3.703704 | 3.611111 | 3.055556 | 2.685185 | 3.302469 |
| 3.734568 | 2.901235 | 3.148148 | 2.530864 | 3.179012 | 2.438272 | 3.82716 | 3.148148 | 3.148148 |
| 2.716049 | 2.685185 | 3.117284 | 2.685185 | 3.333333 | 3.641975 | 2.901235 | 2.87037 | 3.395062 |
| 3.549383 | 4.197531 | 3.148148 | 3.580247 | 3.364198 | 3.302469 | 3.703704 | 3.950617 | 2.777778 |
| 3.950617 | 3.734568 | 3.888889 | 3.487654 | 3.117284 | 3.734568 | 3.364198 | 3.45679 | 3.765432 |
| 4.691358 | 3.518519 | 3.641975 | 3.45679 | 3.82716 | 2.839506 | 3.796296 | 3.302469 | 3.703704 |
| 3.580247 | 3.950617 | 4.135802 | 3.395062 | 2.932099 | 2.901235 | 3.487654 | 3.580247 | 3.796296 |
| 4.197531 | 4.351852 | 3.82716 | 3.919753 | 3.055556 | 2.901235 | 2.685185 | 3.08642 | 2.345679 |
| 4.228395 | 4.228395 | 4.228395 | 2.932099 | 2.685185 | 3.240741 | 2.592593 | 2.623457 | 3.82716 |

| | | | |
|---|---|---|---|
| 3.302469 | 3.67284 | 3.08642 | 3.353909667 |
| 3.333333 | 3.209877 | 3.302469 | 3.281893 |
| 3.734568 | 2.901235 | 3.148148 | 3.261317 |
| 2.716049 | 2.685185 | 3.117284 | 2.839506 |
| 3.549383 | 4.197531 | 3.148148 | 3.631687333 |
| 3.950617 | 3.734568 | 3.888889 | 3.858024667 |
| 4.691358 | 3.518519 | 3.641975 | 3.950617333 |
| 3.580247 | 3.950617 | 4.135802 | 3.888888667 |
| ~~4.197531~~ | ~~4.351852~~ | ~~3.82716~~ | ~~4.125514333~~ |
| ~~4.228395~~ | ~~4.228395~~ | ~~4.228395~~ | ~~4.228395~~ |

| | | | |
|---|---|---|---|
| 4.506173 | 3.395062 | 4.135802 | 4.012345667 |
| 3.611111 | 3.703704 | 3.611111 | 3.641975333 |
| 2.530864 | 3.179012 | 2.438272 | 2.716049333 |
| 2.685185 | 3.333333 | 3.641975 | 3.220164333 |
| 3.580247 | 3.364198 | 3.302469 | 3.415638 |
| 3.487654 | 3.117284 | 3.734568 | 3.446502 |

| | | | |
|---|---|---|---|
| 3.45679 | 3.82716 | 2.839506 | 3.374485333 |
| 3.395062 | 2.932099 | 2.901235 | 3.076132 |
| 3.919753 | 3.055556 | 2.901235 | 3.292181333 |
| 2.932099 | 2.685185 | 3.240741 | 2.952675 |
| | | | |
| 3.055556 | 3.425926 | 2.808642 | 3.096708 |
| 3.055556 | 2.685185 | 3.302469 | 3.014403333 |
| 3.82716 | 3.148148 | 3.148148 | 3.374485333 |
| 2.901235 | 2.87037 | 3.395062 | 3.055555667 |
| 3.703704 | 3.950617 | 2.777778 | 3.477366333 |
| 3.364198 | 3.45679 | 3.765432 | 3.528806667 |
| 3.796296 | 3.302469 | 3.703704 | 3.600823 |
| 3.487654 | 3.580247 | 3.796296 | 3.621399 |
| 2.685185 | 3.08642 | 2.345679 | 2.705761333 |
| 2.592593 | 2.623457 | 3.82716 | 3.014403333 |

| 9 | 10 | 11 | 12 |
|---|---|---|---|
| 3.148148 | 2.716049 | 2.685185 | 3.117284 |
| 3.641975 | 3.580247 | 3.950617 | 4.135802 |
| 4.135802 | 3.611111 | 3.703704 | 3.611111 |
| 3.302469 | 3.487654 | 3.117284 | 3.734568 |
| 2.901235 | 2.932099 | 2.685185 | 3.240741 |
| 3.148148 | 2.901235 | 2.87037 | 3.395062 |
| 3.703704 | 3.487654 | 3.580247 | 3.796296 |
| | | | |

Report

Ca

| Group | Mean | Std. Deviation |
|---|---|---|
| 1 | 3.50823 | 0.389396 |
| 2 | 3.314815 | 0.362209 |
| 3 | 3.248971 | 0.311706 |
| Total | 3.346561 | 0.356475 |

Tests of Normality

| | Group | Kolmogorov-Smirnova | | | Shapiro-Wilk | | |
|---|---|---|---|---|---|---|---|
| | | Statisticdf | | Sig. | Statisticdf | | Sig. |
| Ca | 1 | 0.19 | 8 | .200* | 0.918 | 8 | 0.418 |
| | 2 | 0.158 | 10 | .200* | 0.983 | 10 | 0.978 |
| | 3 | 0.187 | 10 | .200* | 0.909 | 10 | 0.272 |

* This is a lower bound of the true significance.

a Lilliefors Significance Correction

ANOVA

Ca

| | Sum of Sq | df | Mean Squa | F | Sig. |
|---|---|---|---|---|---|
| Between G | 0.314 | 2 | 0.157 | 1.261 | 0.301 |
| Within Gr | 3.117 | 25 | 0.125 | | |
| Total | 3.431 | 27 | | | |

Multiple Comparisons

Dependent Variable: Ca

Tukey HSD

| (I) Group | (J) Group | Mean Diff | Std. Errc | Sig. | 95% Confidence Interval | |
|---|---|---|---|---|---|---|
| | | | | | Lower Bou | Upper Bound |
| 1 | 2 | 0.193416 | 0.16748 | 0.49 | -0.22375 | 0.610579 |
| | 3 | 0.259259 | 0.16748 | 0.286 | -0.1579 | 0.676423 |
| 2 | 1 | -0.19342 | 0.16748 | 0.49 | -0.61058 | 0.223748 |
| | 3 | 0.065844 | 0.157901 | 0.909 | -0.32746 | 0.459149 |
| 3 | 1 | -0.25926 | 0.16748 | 0.286 | -0.67642 | 0.157904 |
| | 2 | -0.06584 | 0.157901 | 0.909 | -0.45915 | 0.327462 |

| | Animal ID | Start Time | End Time | Total Duration (s) |
|---|---|---|---|---|
| 1 | 25 | 0:00:00 | 0:05:10 | 309.98 |
| 2 | 26 | 0:00:00 | 0:05:10 | 309.98 |
| 3 | 27 | 0:00:00 | 0:05:10 | 309.98 |
| 4 | 29 | 0:00:00 | 0:05:10 | 309.98 |
| 5 | 30 | 0:00:00 | 0:05:10 | 309.98 |
| 6 | 50 | 0:00:00 | 0:05:10 | 309.98 |
| 7 | 35 | 0:00:00 | 0:05:10 | 309.98 |
| 8 | 37 | 0:00:00 | 0:05:10 | 309.98 |
| 9 | 49 | 0:00:00 | 0:05:10 | 309.98 |
| 10 | 42 | 0:00:00 | 0:05:10 | 309.98 |
| 11 | 40 | 0:00:00 | 0:05:10 | 310 |
| 12 | 47 | 0:00:00 | 0:05:10 | 309.98 |
| 1 | 1 | 0:00:00 | 0:05:10 | 309.98 |
| 2 | 3 | 0:00:00 | 0:05:10 | 309.98 |
| 3 | 4 | 0:00:00 | 0:05:10 | 309.98 |
| 4 | 6 | 0:00:00 | 0:05:10 | 309.98 |
| 5 | 13 | 0:00:00 | 0:05:10 | 309.98 |
| 6 | 16 | 0:00:00 | 0:05:10 | 309.98 |
| 7 | 17 | 0:00:00 | 0:05:10 | 309.98 |
| 8 | 18 | 0:00:00 | 0:05:10 | 309.98 |
| 9 | 19 | 0:00:00 | 0:05:10 | 309.98 |
| 10 | 20 | 0:00:00 | 0:05:10 | 310 |
| 11 | 28 | 0:00:00 | 0:05:10 | 309.98 |
| 12 | 43 | 0:00:00 | 0:05:10 | 309.98 |
| 13 | 44 | 0:00:00 | 0:05:10 | 309.98 |
| 14 | 45 | 0:00:00 | 0:05:10 | 309.98 |
| 15 | 7 | 0:00:00 | 0:05:10 | 309.98 |
| 16 | 21 | 0:00:00 | 0:05:10 | 309.98 |
| 17 | 22 | 0:00:00 | 0:05:10 | 309.98 |
| 18 | 23 | 0:00:00 | 0:05:10 | 309.98 |
| 1 | 38 | 0:00:00 | 0:05:10 | 309.98 |
| 2 | 2 | 0:00:00 | 0:05:10 | 309.98 |
| 3 | 10 | 0:00:00 | 0:05:10 | 310 |
| 4 | 12 | 0:00:00 | 0:05:10 | 309.98 |
| 5 | 39 | 0:00:00 | 0:05:10 | 309.98 |
| 6 | 8 | 0:00:00 | 0:05:10 | 309.98 |
| 7 | 9 | 0:00:00 | 0:05:10 | 309.98 |
| 8 | 15 | 0:00:00 | 0:05:10 | 309.98 |
| 9 | 24 | 0:00:00 | 0:05:10 | 309.98 |
| 10 | 31 | 0:00:00 | 0:05:10 | 309.98 |
| 11 | 32 | 0:00:00 | 0:05:10 | 310 |
| 12 | 33 | 0:00:00 | 0:05:10 | 309.98 |
| 13 | 34 | 0:00:00 | 0:05:10 | 309.98 |
| 14 | 36 | 0:00:00 | 0:05:10 | 309.98 |
| 15 | 48 | 0:00:00 | 0:05:10 | 309.98 |
| 16 | 51 | 0:00:00 | 0:05:10 | 309.98 |

| Total Distance Travelled (mm) | Total Arm Entries | Total Time in Arms (s) |
|---|---|---|
| 13997.01 | 25 | 253.01 |
| 14327.78 | 26 | 241.45 |
| 14759.72 | 25 | 204.42 |
| 15834.02 | 34 | 244.25 |
| 10530.26 | 19 | 250.17 |
| 16965.52 | 34 | 247.87 |
| 9759.15 | 21 | 269.41 |
| 10578.48 | 25 | 246.53 |
| 8035.44 | 18 | 256.83 |
| 10679.56 | 30 | 272.16 |
| 11595.2 | 25 | 245.76 |
| 12579.95 | 25 | 222.78 |
| 16071.13 | 23 | 222.14 |
| 11415.37 | 14 | 233.78 |
| 10546.77 | 14 | 252.02 |
| 6042.57 | 6 | 286.46 |
| 8309.73 | 10 | 259.62 |
| 6865.89 | 7 | 245.74 |
| 11308.22 | 14 | 261.14 |
| 10934.16 | 16 | 255.62 |
| 11080.16 | 15 | 233.18 |
| 5486.85 | 10 | 268.05 |
| 13420.72 | 17 | 258.85 |
| 11133.26 | 22 | 244.67 |
| 8033.48 | 11 | 284.89 |
| 11954.17 | 29 | 246.31 |
| 9786.63 | 14 | 260.26 |
| 14354.8 | 25 | 258.73 |
| 15745.25 | 27 | 252.21 |
| 12637.36 | 19 | 246.29 |
| 13433.87 | 21 | 232.68 |
| 13008 | 20 | 250.02 |
| 12663.76 | 23 | 247.05 |
| 10627.35 | 16 | 209.26 |
| 12601.72 | 24 | 239.81 |
| 11974.73 | 19 | 222.66 |
| 11319.36 | 18 | 228.62 |
| 11354.3 | 17 | 222.49 |
| 16404.45 | 24 | 233.09 |
| 11420.14 | 17 | 257.57 |
| 13400.79 | 20 | 234.01 |
| 12247.43 | 19 | 243.81 |
| 12049.26 | 20 | 243.29 |
| 8779.19 | 12 | 268.52 |
| 5016.25 | 14 | 239.66 |
| 11439.72 | 19 | 260.69 |

| Time in Open Arms (s) | Distance in Open Arms (mm) | Open Arm Entries |
|---|---|---|
| 100.87 | 881.23 | 10 |
| 80.59 | 905.35 | 9 |
| 53.39 | 850.47 | 8 |
| 119.51 | 1023.75 | 14 |
| 101.11 | 643.98 | 8 |
| 65.34 | 1098.34 | 12 |
| 137.1 | 703.66 | 10 |
| 96.99 | 1209.53 | 12 |
| 99.21 | 1198.46 | 11 |
| 153.85 | 1305.63 | 12 |
| 123.12 | 1003.79 | 12 |
| 79.38 | 577.79 | 9 |
| 28.04 | 125.36 | 2 |
| 12.64 | 0 | 0 |
| 15.44 | 40.35 | 1 |
| 4.44 | 0 | 0 |
| 57.56 | 30.62 | 1 |
| 25.68 | 41.45 | 1 |
| 37.48 | 100.9 | 2 |
| 39.56 | 200.29 | 3 |
| 25.28 | 49.56 | 2 |
| 86.15 | 301.8 | 5 |
| 29.52 | 20.45 | 1 |
| 112.44 | 289.35 | 6 |
| 146.53 | 155.81 | 3 |
| 44.71 | 230.65 | 5 |
| 33.72 | 212.46 | 3 |
| 63.71 | 517.21 | 8 |
| 48.95 | 450.84 | 7 |
| 108.83 | 405.22 | 6 |
| 53.08 | 389.23 | 6 |
| 60.76 | 402.45 | 5 |
| 80.15 | 365.12 | 6 |
| 54.8 | 244.37 | 4 |
| 94.35 | 570.71 | 9 |
| 64.51 | 395.74 | 5 |
| 77.51 | 401.63 | 6 |
| 50.59 | 277.63 | 4 |
| 55.27 | 473.66 | 6 |
| 31.12 | 383.76 | 4 |
| 56.91 | 327.87 | 5 |
| 59.47 | 302.76 | 4 |
| 80.39 | 400.51 | 6 |
| 51.04 | 233.98 | 4 |
| 47.56 | 520.67 | 9 |
| 55.97 | 937.26 | 7 |

| Time in Closed Arms (s) | Distance in Closed Arms (mm) | Closed Arm Entries |
|---|---|---|
| 152.14 | 9838.14 | 15 |
| 160.86 | 10011.07 | 17 |
| 151.03 | 9679.84 | 17 |
| 124.74 | 11347.3 | 20 |
| 149.06 | 7317.89 | 11 |
| 182.53 | 13357.64 | 22 |
| 132.31 | 7090.37 | 11 |
| 149.54 | 6653.36 | 13 |
| 157.62 | 4781.57 | 7 |
| 118.31 | 6386.34 | 18 |
| 122.64 | 7860.25 | 13 |
| 143.4 | 10324.57 | 14 |
| 194.1 | 12905.89 | 21 |
| 221.14 | 9402.96 | 14 |
| 236.58 | 9072.61 | 13 |
| 282.02 | 5642.49 | 6 |
| 202.06 | 7025.58 | 9 |
| 220.06 | 5608.99 | 6 |
| 223.66 | 9546.53 | 12 |
| 216.06 | 9284.51 | 13 |
| 207.9 | 8812.67 | 13 |
| 181.9 | 4212.12 | 5 |
| 229.33 | 11197.08 | 16 |
| 132.23 | 8723.45 | 16 |
| 138.36 | 6345.23 | 8 |
| 201.6 | 10394.54 | 24 |
| 226.54 | 8191.44 | 11 |
| 195.02 | 11443.41 | 17 |
| 203.26 | 13036.59 | 20 |
| 137.46 | 9492.96 | 13 |
| 179.6 | 10061.58 | 15 |
| 189.26 | 10602.49 | 15 |
| 166.9 | 10415.23 | 17 |
| 154.46 | 7808.29 | 12 |
| 145.46 | 9471.2 | 15 |
| 158.15 | 9479.53 | 14 |
| 151.11 | 8614.4 | 12 |
| 171.9 | 8895.23 | 13 |
| 177.82 | 11989.83 | 18 |
| 226.45 | 9180.12 | 13 |
| 177.1 | 9942.71 | 15 |
| 184.34 | 9126.47 | 15 |
| 162.9 | 8921.74 | 14 |
| 217.48 | 7225.18 | 8 |
| 192.1 | 3468.24 | 5 |
| 204.72 | 8275.75 | 12 |

| Time in Center (s) | Distance in Center (mm) | Center Entries | Time in ( |
|---|---|---|---|
| 56.97 | 3277.64 | 25 | 32.54 |
| 68.53 | 3411.36 | 26 | 26 |
| 105.56 | 4229.41 | 24 | 17.22 |
| 65.73 | 3462.97 | 34 | 38.55 |
| 59.81 | 2568.39 | 19 | 32.62 |
| 62.11 | 2509.54 | 20 | 21.08 |
| 40.57 | 1965.12 | 21 | 44.23 |
| 63.45 | 2715.59 | 25 | 31.29 |
| 53.15 | 2055.41 | 17 | 32.01 |
| 37.82 | 2987.59 | 33 | 49.63 |
| 64.24 | 2731.16 | 25 | 39.72 |
| 87.2 | 1677.59 | 15 | 25.61 |
| 87.84 | 3039.88 | 22 | 9.05 |
| 76.2 | 2012.41 | 14 | 4.08 |
| 57.96 | 1433.81 | 13 | 4.98 |
| 23.52 | 400.08 | 6 | 1.43 |
| 50.36 | 1253.53 | 10 | 18.57 |
| 64.24 | 1215.45 | 7 | 8.28 |
| 48.84 | 1660.79 | 14 | 12.09 |
| 54.36 | 1449.36 | 16 | 12.76 |
| 76.8 | 2217.93 | 15 | 8.16 |
| 41.95 | 972.93 | 10 | 27.79 |
| 51.13 | 2203.19 | 17 | 9.52 |
| 65.31 | 2120.46 | 23 | 36.27 |
| 25.09 | 1532.44 | 11 | 47.27 |
| 63.67 | 1328.98 | 14 | 14.42 |
| 49.72 | 1382.73 | 14 | 10.88 |
| 51.25 | 2394.18 | 25 | 20.55 |
| 57.77 | 2257.82 | 27 | 15.79 |
| 63.69 | 2739.18 | 19 | 35.11 |
| 77.3 | 2983.06 | 21 | 17.12 |
| 59.96 | 2003.06 | 20 | 19.6 |
| 62.95 | 1883.41 | 22 | 25.85 |
| 100.72 | 2574.69 | 16 | 17.68 |
| 70.17 | 2559.81 | 23 | 30.44 |
| 87.32 | 2099.46 | 18 | 20.81 |
| 81.36 | 2303.33 | 19 | 25 |
| 87.49 | 2181.44 | 16 | 16.32 |
| 76.89 | 3940.96 | 25 | 17.83 |
| 52.41 | 1856.26 | 17 | 10.04 |
| 75.99 | 3130.21 | 20 | 18.36 |
| 66.17 | 2818.2 | 18 | 19.19 |
| 66.69 | 2727.01 | 20 | 25.93 |
| 41.46 | 1320.03 | 13 | 16.47 |
| 70.32 | 1027.34 | 9 | 15.34 |
| 49.29 | 2226.71 | 16 | 18.06 |

| Distance in Open Arms(%) | Open Arm Entries(%) | Time in Closed Arms(%) |
|---|---|---|
| 6.3 | 40 | 49.08 |
| 6.32 | 34.62 | 51.89 |
| 5.76 | 32 | 48.72 |
| 6.47 | 41.18 | 40.24 |
| 6.12 | 42.11 | 48.09 |
| 6.47 | 35.29 | 58.88 |
| 7.21 | 47.62 | 42.68 |
| 11.43 | 48 | 48.24 |
| 14.91 | 61.11 | 50.85 |
| 12.23 | 40 | 38.17 |
| 8.66 | 48 | 39.56 |
| 4.59 | 36 | 46.26 |
| 0.78 | 8.7 | 62.62 |
| 0 | 0 | 71.34 |
| 0.38 | 7.14 | 76.32 |
| 0 | 0 | 90.98 |
| 0.37 | 10 | 65.18 |
| 0.6 | 14.29 | 70.99 |
| 0.89 | 14.29 | 72.15 |
| 1.83 | 18.75 | 69.7 |
| 0.45 | 13.33 | 67.07 |
| 5.5 | 50 | 58.68 |
| 0.15 | 5.88 | 73.98 |
| 2.6 | 27.27 | 42.66 |
| 1.94 | 27.27 | 44.64 |
| 1.93 | 17.24 | 65.04 |
| 2.17 | 21.43 | 73.08 |
| 3.6 | 32 | 62.91 |
| 2.86 | 25.93 | 65.57 |
| 3.21 | 31.58 | 44.34 |
| 2.9 | 28.57 | 57.94 |
| 3.09 | 25 | 61.06 |
| 2.88 | 26.09 | 53.84 |
| 2.3 | 25 | 49.83 |
| 4.53 | 37.5 | 46.93 |
| 3.3 | 26.32 | 51.02 |
| 3.55 | 33.33 | 48.75 |
| 2.45 | 23.53 | 55.46 |
| 2.89 | 25 | 57.36 |
| 3.36 | 23.53 | 73.05 |
| 2.45 | 25 | 57.13 |
| 2.47 | 21.05 | 59.47 |
| 3.32 | 30 | 52.55 |
| 2.67 | 33.33 | 70.16 |
| 10.38 | 64.29 | 61.97 |
| 8.19 | 36.84 | 66.04 |

| Distance in Closed Arms(%) | Closed Arm Entries(%) |
|---|---|
| 70.29 | 60 |
| 69.87 | 65.38 |
| 65.58 | 68 |
| 71.66 | 58.82 |
| 69.49 | 57.89 |
| 78.73 | 64.71 |
| 72.65 | 52.38 |
| 62.9 | 52 |
| 59.51 | 38.89 |
| 59.8 | 60 |
| 67.79 | 52 |
| 82.07 | 56 |
| 80.3 | 91.3 |
| 82.37 | 100 |
| 86.02 | 92.86 |
| 93.38 | 100 |
| 84.55 | 90 |
| 81.69 | 85.71 |
| 84.42 | 85.71 |
| 84.91 | 81.25 |
| 79.54 | 86.67 |
| 76.77 | 50 |
| 83.43 | 94.12 |
| 78.35 | 72.73 |
| 78.98 | 72.73 |
| 86.95 | 82.76 |
| 83.7 | 78.57 |
| 79.72 | 68 |
| 82.8 | 74.07 |
| 75.12 | 68.42 |
| 74.9 | 71.43 |
| 81.51 | 75 |
| 82.24 | 73.91 |
| 73.47 | 75 |
| 75.16 | 62.5 |
| 79.16 | 73.68 |
| 76.1 | 66.67 |
| 78.34 | 76.47 |
| 73.09 | 75 |
| 80.39 | 76.47 |
| 74.19 | 75 |
| 74.52 | 78.95 |
| 74.04 | 70 |
| 82.3 | 66.67 |
| 69.14 | 35.71 |
| 72.34 | 63.16 |

Report

| Group | | Time | Distance | Number |
|---|---|---|---|---|
| 1 | Mean | 100.8717 | 950.165 | 10.5833 |
| | Std. Devi | 29.18546 | 232.65246 | 1.88092 |
| 2 | Mean | 51.1489 | 176.24 | 3.1111 |
| | Std. Devi | 38.75709 | 162.05823 | 2.47074 |
| 3 | Mean | 60.8425 | 414.2094 | 5.625 |
| | Std. Devi | 15.48949 | 166.85049 | 1.62788 |
| Total | Mean | 67.4917 | 460.9054 | 5.9348 |
| | Std. Devi | 35.71184 | 359.79811 | 3.61111 |

Tests of Normality

| | Group | Kolmogorov-Smirnova | | | Shapiro-Wilk | | |
|---|---|---|---|---|---|---|---|
| | | Statistic | df | Sig. | Statistic | df | Sig. |
| Time | 1 | 0.163 | 12 | .200* | 0.978 | 12 | 0.975 |
| | 2 | 0.189 | 18 | 0.088 | 0.879 | 18 | 0.025 |
| | 3 | 0.19 | 16 | 0.127 | 0.931 | 16 | 0.249 |
| Distance | 1 | 0.107 | 12 | .200* | 0.965 | 12 | 0.856 |
| | 2 | 0.172 | 18 | 0.171 | 0.9 | 18 | 0.057 |
| | 3 | 0.278 | 16 | 0.002 | 0.786 | 16 | 0.002 |
| Number | 1 | 0.191 | 12 | .200* | 0.928 | 12 | 0.362 |
| | 2 | 0.185 | 18 | 0.107 | 0.914 | 18 | 0.101 |
| | 3 | 0.221 | 16 | 0.035 | 0.842 | 16 | 0.011 |

* This is a lower bound of the true significance.
a Lilliefors Significance Correction

**Hypothesis Test Summary**

| | Null Hypothesis | Test | Sig. | Decision |
|---|---|---|---|---|
| 1 | The distribution of Time is the same across categories of Group. | Independent-Samples Kruskal-Wallis Test | .000 | Reject the null hypothesis. |
| 2 | The distribution of Distance is the same across categories of Group. | Independent-Samples Kruskal-Wallis Test | .000 | Reject the null hypothesis. |
| 3 | The distribution of Number is the same across categories of Group. | Independent-Samples Kruskal-Wallis Test | .000 | Reject the null hypothesis. |

Asymptotic significances are displayed. The significance level is .05.

Multiple Comparisons
Bonferroni

| Dependen | (I) Grou | (J) Grou | Mean Diff | Std. Erro | Sig. | 95% Confidence Interval | |
|---|---|---|---|---|---|---|---|
| | | | | | | Lower Boun | Upper Bound |
| Time | 1 | 2 | 49.72278* | 11.15206 | 0 | 21.9401 | 77.5055 |

| | | | | | | | |
|---|---|---|---|---|---|---|---|
| | | 3 | 40.02917* | 11.42746 | 0.003 | 11.5604 | 68.498 |
| | 2 | 1 | -49.72278: | 11.15206 | 0 | -77.5055 | -21.9401 |
| | | 3 | -9.69361 | 10.28169 | 1 | -35.308 | 15.9208 |
| | 3 | 1 | -40.02917: | 11.42746 | 0.003 | -68.498 | -11.5604 |
| | | 2 | 9.69361 | 10.28169 | 1 | -15.9208 | 35.308 |
| Distance | 1 | 2 | 773.92500: | 68.65856 | 0 | 602.8786 | 944.9714 |
| | | 3 | 535.95563: | 70.35409 | 0 | 360.6852 | 711.226 |
| | 2 | 1 | -773.9250( | 68.65856 | 0 | -944.9714 | -602.8786 |
| | | 3 | -237.9693' | 63.30007 | 0.002 | -395.6664 | -80.2724 |
| | 3 | 1 | -535.9556: | 70.35409 | 0 | -711.226 | -360.6852 |
| | | 2 | 237.96937: | 63.30007 | 0.002 | 80.2724 | 395.6664 |
| Number | 1 | 2 | 7.47222* | 0.76765 | 0 | 5.5598 | 9.3846 |
| | | 3 | 4.95833* | 0.78661 | 0 | 2.9987 | 6.918 |
| | 2 | 1 | -7.47222* | 0.76765 | 0 | -9.3846 | -5.5598 |
| | | 3 | -2.51389* | 0.70774 | 0.003 | -4.2771 | -0.7507 |
| | 3 | 1 | -4.95833* | 0.78661 | 0 | -6.918 | -2.9987 |
| | | 2 | 2.51389* | 0.70774 | 0.003 | 0.7507 | 4.2771 |

* The mean difference is significant at the 0.05 level.

Report

Median

| Group | Time | Distance | Number |
|---|---|---|---|
| 1 | 100.04 | 954.57 | 10.5 |
| 2 | 38.52 | 140.585 | 2.5 |
| 3 | 56.44 | 392.485 | 5.5 |
| Total | 58.515 | 392.485 | 6 |

Percentile(P)

| | | Group | Percentile(P) | | | | | |
|---|---|---|---|---|---|---|---|---|
| | | | 5 | 10 | 25 | 50 | 75 | 90 |
| Weighted | Time | 1 | 53.39 | 56.975 | 79.6825 | 100.04 | 122.2175 | 148.825 |
| | | 2 | 4.44 | 11.82 | 25.58 | 38.52 | 69.32 | 115.849 |
| | | 3 | 31.12 | 42.628 | 51.55 | 56.44 | 74.26 | 84.578 |
| | Distance | 1 | 577.79 | 597.647 | 740.3625 | 954.57 | 1173.43 | 1276.8 |
| | | 2 | 0 | 0 | 37.9175 | 140.585 | 292.4625 | 457.477 |
| | | 3 | 233.98 | 241.253 | 309.0375 | 392.485 | 455.8575 | 680.675 |
| | Number | 1 | 8 | 8 | 9 | 10.5 | 12 | 13.4 |
| | | 2 | 0 | 0 | 1 | 2.5 | 5.25 | 7.1 |
| | | 3 | 4 | 4 | 4 | 5.5 | 6 | 9 |
| Tukey's I | Time | 1 | | | 79.985 | 100.04 | 121.315 | |
| | | 2 | | | 25.68 | 38.52 | 63.71 | |
| | | 3 | | | 52.06 | 56.44 | 71.01 | |
| | Distance | 1 | | | 777.065 | 954.57 | 1148.4 | |
| | | 2 | | | 40.35 | 140.585 | 289.35 | |

| | | | | |
|---|---|---|---|---|
| | 3 | 315.315 | 392.485 | 438.055 |
| Number | 1 | 9 | 10.5 | 12 |
| | 2 | 1 | 2.5 | 5 |
| | 3 | 4 | 5.5 | 6 |

95

.    122.22-79.68
.    69.32-25.58
.    74.26-51.55
.    1173.43-740.3625
.    292.4625-37.9175
.    455.8575-309.0375
.    12-9
.    5.25-1
.    6-4

| Animal ID | Start Time | End Time | Total Duration (s) | Struggle Time (s) | Struggle Time (%) |
|---|---|---|---|---|---|
| 1 | 0:00:00 | 0:04:00 | 239.98 | 179.09 | 74.63 |
| 2 | 0:00:00 | 0:04:00 | 239.98 | 103.57 | 43.16 |
| 3 | 0:00:00 | 0:04:00 | 240 | 165.48 | 68.95 |
| 4 | 0:00:00 | 0:04:00 | 239.98 | 180.03 | 75.02 |
| 5 | 0:00:00 | 0:04:00 | 240 | 159.75 | 66.57 |
| 6 | 0:00:00 | 0:04:00 | 239.98 | 99.76 | 41.57 |
| 7 | 0:00:00 | 0:04:00 | 239.98 | 120 | 50 |
| 8 | 0:00:00 | 0:04:00 | 239.98 | 163.2 | 68 |
| 9 | 0:00:00 | 0:04:00 | 239.98 | 140 | 58.34 |
| 10 | 0:00:00 | 0:04:00 | 240 | 113.74 | 47.4 |
| 11 | 0:00:00 | 0:04:00 | 239.98 | 181.66 | 75.7 |
| 12 | 0:00:00 | 0:04:00 | 240 | 126.61 | 52.75 |
| 13 | 0:00:00 | 0:04:00 | 240 | 169.28 | 70.53 |
| 14 | 0:00:00 | 0:04:00 | 239.98 | 141.59 | 59 |
| 15 | 0:00:00 | 0:04:00 | 239.98 | 96.19 | 40.08 |
| 16 | 0:00:00 | 0:04:00 | 239.98 | 102.55 | 42.73 |
| 17 | 0:00:00 | 0:04:00 | 239.98 | 109.92 | 45.8 |
| 18 | 0:00:00 | 0:04:00 | 239.98 | 65.87 | 27.45 |
| 19 | 0:00:00 | 0:04:00 | 239.98 | 143.27 | 59.7 |
| 20 | 0:00:00 | 0:04:00 | 239.98 | 81.73 | 34.06 |
| 21 | 0:00:00 | 0:04:00 | 239.98 | 64.94 | 27.06 |
| 22 | 0:00:00 | 0:04:00 | 239.98 | 36.5 | 15.21 |
| 23 | 0:00:00 | 0:04:00 | 240 | 70.67 | 29.45 |
| 24 | 0:00:00 | 0:04:00 | 239.98 | 88.65 | 36.94 |
| 25 | 0:00:00 | 0:04:00 | 239.98 | 128.67 | 53.62 |
| 26 | 0:00:00 | 0:04:00 | 239.98 | 86.25 | 35.94 |
| 27 | 0:00:00 | 0:04:00 | 239.98 | 181.43 | 75.6 |
| 28 | 0:00:00 | 0:04:00 | 239.98 | 57.71 | 24.05 |
| 29 | 0:00:00 | 0:04:00 | 239.98 | 89.7 | 37.38 |
| 30 | 0:00:00 | 0:04:00 | 240 | 61.6 | 25.67 |
| 31 | 0:00:00 | 0:04:00 | 239.98 | 129.81 | 54.09 |
| 32 | 0:00:00 | 0:04:00 | 240 | 150.66 | 62.78 |
| 33 | 0:00:00 | 0:04:00 | 239.98 | 149.48 | 62.29 |
| 34 | 0:00:00 | 0:04:00 | 240 | 141.64 | 59.01 |
| 35 | 0:00:00 | 0:04:00 | 239.98 | 118.41 | 49.34 |
| 36 | 0:00:00 | 0:04:00 | 240 | 80.04 | 33.35 |
| 37 | 0:00:00 | 0:04:00 | 239.98 | 143.72 | 59.9 |
| 38 | 0:00:00 | 0:04:00 | 239.98 | 171.38 | 71.41 |
| 39 | 0:00:00 | 0:04:00 | 239.98 | 77.31 | 32.22 |
| 40 | 0:00:00 | 0:04:00 | 239.98 | 152.93 | 63.73 |
| 41 | 0:00:00 | 0:04:00 | 239.98 | 154.75 | 64.48 |
| 42 | 0:00:00 | 0:04:00 | 239.98 | 105.32 | 43.89 |
| 43 | 0:00:00 | 0:04:00 | 239.98 | 63.94 | 26.64 |
| 44 | 0:00:00 | 0:04:00 | 239.98 | 137.96 | 57.49 |
| 45 | 0:00:00 | 0:04:00 | 239.98 | 127.14 | 52.98 |
| 46 | 0:00:00 | 0:04:00 | 239.98 | 105.95 | 44.15 |

| Swimming Time (s) | Swimming Time (%) | Immobility Time (s) | Immobility Time (%) |
|---|---|---|---|
| 40.53 | 16.89 | 20.36 | 8.48 |
| 37.62 | 15.67 | 98.79 | 41.17 |
| 42.5 | 17.71 | 32.02 | 13.34 |
| 30.28 | 12.62 | 29.67 | 12.36 |
| 12.33 | 5.14 | 67.92 | 28.3 |
| 50.63 | 21.1 | 89.59 | 37.33 |
| 42.15 | 17.57 | 77.83 | 32.43 |
| 29.58 | 12.33 | 47.2 | 19.67 |
| 51.5 | 21.46 | 48.48 | 20.2 |
| 47.78 | 19.91 | 78.48 | 32.7 |
| 31.54 | 76.34 | 40 | 16.67 |
| 60.55 | 25.23 | 52.84 | 22.02 |
| 10.32 | 4.3 | 60.4 | 25.17 |
| 23.04 | 9.6 | 75.35 | 31.4 |
| 9.08 | 3.78 | 134.71 | 56.13 |
| 14.52 | 6.05 | 122.91 | 51.22 |
| 28.55 | 11.9 | 101.51 | 42.3 |
| 30 | 12.5 | 144.11 | 60.05 |
| 2.32 | 0.97 | 94.39 | 39.33 |
| 14.38 | 5.99 | 143.87 | 59.95 |
| 7.69 | 3.2 | 167.35 | 69.73 |
| 10.66 | 4.44 | 192.82 | 80.35 |
| 30.33 | 12.64 | 139 | 57.92 |
| 19.54 | 8.14 | 131.79 | 54.92 |
| 29.36 | 12.23 | 81.95 | 34.15 |
| 4.98 | 2.08 | 148.75 | 61.98 |
| 12.39 | 5.16 | 46.16 | 19.23 |
| 21.88 | 9.12 | 160.39 | 66.83 |
| 19.45 | 8.1 | 130.83 | 54.52 |
| 17.2 | 7.17 | 161.2 | 67.17 |
| 18.98 | 7.91 | 91.19 | 38 |
| 27.66 | 11.53 | 61.68 | 25.7 |
| 15.38 | 6.41 | 75.12 | 31.3 |
| 24.72 | 10.3 | 73.64 | 30.68 |
| 20.58 | 8.58 | 100.99 | 42.08 |
| 9.56 | 3.99 | 150.4 | 62.67 |
| 30.83 | 12.85 | 65.43 | 27.26 |
| 10.96 | 4.57 | 57.64 | 24.02 |
| 27.32 | 11.38 | 135.35 | 56.4 |
| 36.53 | 15.22 | 50.52 | 21.05 |
| 14.32 | 5.97 | 70.91 | 29.55 |
| 20.83 | 8.68 | 113.83 | 47.43 |
| 17.73 | 7.39 | 158.31 | 65.97 |
| 27.55 | 11.48 | 74.47 | 31.03 |
| 8.64 | 3.6 | 104.2 | 43.42 |
| 16.52 | 6.88 | 117.51 | 48.97 |

Test for Normality

| | Group | Kolmogorov-Smirnov(K)a | | | Shapiro-Wilk | | |
|---|---|---|---|---|---|---|---|
| | | Statistic | df | Sig. | Statistic | df | Sig. |
| Struggle | 1 | 0.191 | 12 | .200* | 0.899 | 12 | 0.153 |
| | 2 | 0.144 | 18 | .200* | 0.944 | 18 | 0.341 |
| | 3 | 0.153 | 16 | .200* | 0.928 | 16 | 0.226 |
| Swimming | 1 | 0.129 | 12 | .200* | 0.967 | 12 | 0.878 |
| | 2 | 0.126 | 18 | .200* | 0.948 | 18 | 0.389 |
| | 3 | 0.114 | 16 | .200* | 0.967 | 16 | 0.795 |
| Immobilit | 1 | 0.148 | 12 | .200* | 0.955 | 12 | 0.707 |
| | 2 | 0.176 | 18 | 0.144 | 0.957 | 18 | 0.551 |
| | 3 | 0.211 | 16 | 0.054 | 0.923 | 16 | 0.188 |

* This is a lower bound of the true significance.
a Lilliefors Significance Correction

Test of Homogeneity of Variances

| | Levene's | df1 | df2 | Sig. |
|---|---|---|---|---|
| Struggle | 0.526 | 2 | 43 | 0.595 |
| Swimming | 1.074 | 2 | 43 | 0.351 |
| Immobilit | 1.216 | 2 | 43 | 0.306 |

ANOVA

| | | Sum of | df | Mean Squa | F | Sig. |
|---|---|---|---|---|---|---|
| Struggle | Between ( | 15829.75 | 2 | 7914.876 | 6.497 | 0.003 |
| | Within Gr | 52384.21 | 43 | 1218.238 | | |
| | Total | 68213.97 | 45 | | | |
| Swimming | Between ( | 4057.009 | 2 | 2028.504 | 21.331 | 0 |
| | Within Gr | 4089.17 | 43 | 95.097 | | |
| | Total | 8146.179 | 45 | | | |
| Immobilit | Between ( | 32812.12 | 2 | 16406.06 | 13.913 | 0 |
| | Within Gr | 50706.52 | 43 | 1179.221 | | |
| | Total | 83518.64 | 45 | | | |

Multiple Comparisons

| Dependent Variable | | (I) Group | (J) Group | Mean Diff | Std. Error | Sig. | 95% Confidence Interval | |
|---|---|---|---|---|---|---|---|---|
| | | | | | | | Lower Bou | Upper Bound |
| Struggle | Tukey HSI | 1 | 2 | 45.71194* | 13.00768 | 0.003 | 14.1366 | 77.2873 |
| | | | 3 | 18.755 | 13.3289 | 0.346 | -13.6001 | 51.1101 |
| | | 2 | 1 | -45.7119 | 13.00768 | 0.003 | -77.2873 | -14.1366 |
| | | | 3 | -26.9569 | 11.99249 | 0.075 | -56.068 | 2.1541 |
| | | 3 | 1 | -18.755 | 13.3289 | 0.346 | -51.1101 | 13.6001 |
| | | | 2 | 26.95694 | 11.99249 | 0.075 | -2.1541 | 56.068 |
| | Bonferror | 1 | 2 | 45.71194* | 13.00768 | 0.003 | 13.3064 | 78.1175 |

| | | | | | | | | |
|---|---|---|---|---|---|---|---|---|
| | | | 3 | 18.755 | 13.3289 | 0.5 | -14.4508 | 51.9608 |
| | | 2 | 1 | -45.7119 | 13.00768 | 0.003 | -78.1175 | -13.3064 |
| | | | 3 | -26.9569 | 11.99249 | 0.089 | -56.8334 | 2.9195 |
| | | 3 | 1 | -18.755 | 13.3289 | 0.5 | -51.9608 | 14.4508 |
| | | | 2 | 26.95694 | 11.99249 | 0.089 | -2.9195 | 56.8334 |
| Swimming | Tukey HSI | 1 | 2 | 22.76639* | 3.63427 | 0 | 13.9444 | 31.5884 |
| | | | 3 | 19.24229* | 3.72402 | 0 | 10.2025 | 28.2821 |
| | | 2 | 1 | -22.7663 | 3.63427 | 0 | -31.5884 | -13.9444 |
| | | | 3 | -3.5241 | 3.35063 | 0.549 | -11.6576 | 4.6094 |
| | | 3 | 1 | -19.2422 | 3.72402 | 0 | -28.2821 | -10.2025 |
| | | | 2 | 3.5241 | 3.35063 | 0.549 | -4.6094 | 11.6576 |
| | Bonferror | 1 | 2 | 22.76639* | 3.63427 | 0 | 13.7125 | 31.8203 |
| | | | 3 | 19.24229* | 3.72402 | 0 | 9.9648 | 28.5198 |
| | | 2 | 1 | -22.7663 | 3.63427 | 0 | -31.8203 | -13.7125 |
| | | | 3 | -3.5241 | 3.35063 | 0.896 | -11.8714 | 4.8232 |
| | | 3 | 1 | -19.2422 | 3.72402 | 0 | -28.5198 | -9.9648 |
| | | | 2 | 3.5241 | 3.35063 | 0.896 | -4.8232 | 11.8714 |
| Immobilit | Tukey HSI | 1 | 2 | -67.3733 | 12.79769 | 0 | -98.4389 | -36.3077 |
| | | | 3 | -36.8927 | 13.11373 | 0.02 | -68.7255 | -5.0599 |
| | | 2 | 1 | 67.37333* | 12.79769 | 0 | 36.3077 | 98.4389 |
| | | | 3 | 30.48062* | 11.79888 | 0.035 | 1.8395 | 59.1217 |
| | | 3 | 1 | 36.89271* | 13.11373 | 0.02 | 5.0599 | 68.7255 |
| | | | 2 | -30.4806 | 11.79888 | 0.035 | -59.1217 | -1.8395 |
| | Bonferror | 1 | 2 | -67.3733 | 12.79769 | 0 | -99.2557 | -35.491 |
| | | | 3 | -36.8927 | 13.11373 | 0.022 | -69.5624 | -4.223 |
| | | 2 | 1 | 67.37333* | 12.79769 | 0 | 35.491 | 99.2557 |
| | | | 3 | 30.48062* | 11.79888 | 0.04 | 1.0865 | 59.8747 |
| | | 3 | 1 | 36.89271* | 13.11373 | 0.022 | 4.223 | 69.5624 |
| | | | 2 | -30.4806 | 11.79888 | 0.04 | -59.8747 | -1.0865 |

* The mean difference is significant at the 0.05 level.

Report

| Group | | Struggle | Swimming | Immobility Time |
|---|---|---|---|---|
| 1 | Mean | 144.4075 | 39.7492 | 56.9317 |
| | N | 12 | 12 | 12 |
| | Standard | 30.78619 | 12.73648 | 25.24667 |
| 2 | Mean | 98.6956 | 16.9828 | 124.305 |
| | N | 18 | 18 | 18 |
| | Standard | 40.03725 | 8.85875 | 39.70734 |
| 3 | Mean | 125.6525 | 20.5069 | 93.8244 |
| | N | 16 | 16 | 16 |
| | Standard | 31.3133 | 8.04429 | 33.55766 |
| Total | Mean | 119.9967 | 24.1476 | 96.1274 |
| | N | 46 | 46 | 46 |
| | Standard | 38.93412 | 13.4546 | 43.08097 |

| Group | Total Distance | Average Speed | Movement Time | Center Distance |
|---|---|---|---|---|
| 1 | 10738.2 | 35.79 | 239.49 | 511.74 |
| 1 | 5925.88 | 19.62 | 150.8 | 144.04 |
| 1 | 22615.37 | 75.13 | 284.49 | 589.51 |
| 1 | 27500.01 | 91.36 | 280.37 | 4324.67 |
| 1 | 24893.29 | 82.7 | 283.53 | 1748.22 |
| 1 | 18209.28 | 60.5 | 251.37 | 683.42 |
| 1 | 11654.08 | 38.72 | 251.37 | 75.27 |
| 1 | 42901.86 | 142.53 | 260.29 | 0 |
| 1 | 9419.78 | 31.29 | 227.85 | 766.47 |
| 1 | 20988.98 | 69.73 | 275.09 | 770.68 |
| 1 | 10977.69 | 36.47 | 203.06 | 255.31 |
| 1 | 19318.02 | 64.18 | 99.67 | 0 |
| 2 | 2603.79 | 8.65 | 113.43 | 864.14 |
| 2 | 3354.43 | 11.14 | 109.59 | 0 |
| 2 | 1782.67 | 5.94 | 88.07 | 499.54 |
| 2 | 2618.73 | 8.73 | 125.58 | 0 |
| 2 | 1812.74 | 6.04 | 77.07 | 0 |
| 2 | 4594.01 | 15.31 | 132.82 | 1300.29 |
| 2 | 2975.02 | 9.92 | 201.62 | 0 |
| 2 | 7034.84 | 23.45 | 263.93 | 307.35 |
| 2 | 2873.25 | 9.58 | 233.73 | 0 |
| 2 | 2989.13 | 9.96 | 132.58 | 0 |
| 2 | 7118.11 | 23.73 | 204.22 | 12.74 |
| 2 | 9099.41 | 30.33 | 122.63 | 141.66 |
| 2 | 15501.35 | 51.67 | 246.17 | 271.86 |
| 2 | 2311.76 | 7.71 | 141.42 | 0 |
| 2 | 2403.17 | 8.01 | 121.67 | 423.2 |
| 2 | 7131.94 | 23.77 | 173.18 | 0 |
| 2 | 9163.97 | 30.55 | 214.45 | 1005.26 |
| 2 | 2676.64 | 8.92 | 159.74 | 6.74 |
| 3 | 13353.06 | 44.51 | 245.25 | 61.57 |
| 3 | 19371.46 | 64.57 | 260.09 | 201.3 |
| 3 | 52287.97 | 174.29 | 280.73 | 145.32 |
| 3 | 45705.89 | 152.35 | 221.73 | 74.77 |
| 3 | 35171.71 | 117.24 | 273.01 | 6326.12 |
| 3 | 2090.41 | 6.97 | 70.55 | 300.21 |
| 3 | 1462.05 | 4.87 | 67.63 | 184.41 |
| 3 | 12316.26 | 41.05 | 140.9 | 2263.72 |
| 3 | 2441.99 | 8.14 | 115.23 | 84.84 |
| 3 | 24495.38 | 81.65 | 256.17 | 5259.03 |
| 3 | 11772.64 | 39.24 | 228.25 | 0 |
| 3 | 2865.56 | 9.55 | 132.78 | 0 |
| 3 | 1199.54 | 4 | 57.51 | 115.63 |
| 3 | 29030.36 | 96.77 | 293.28 | 2201.32 |
| 3 | 10749.95 | 35.71 | 190.1 | 43.43 |
| 3 | 13261.45 | 44.2 | 237.05 | 6.81 |

| Periphery Distance | Rearing Frequency | Hanging Time |
|---|---|---|
| 10226.47 | 6 | 0 |
| 5781.84 | 1 | 0 |
| 22025.85 | 18 | 4 |
| 23175.35 | 27 | 16 |
| 23145.07 | 24 | 18 |
| 17525.86 | 13 | 10 |
| 11578.82 | 8 | 6 |
| 42901.86 | 0 | 0 |
| 8653.31 | 2 | 0 |
| 20218.3 | 3 | 0 |
| 10722.38 | 8 | 0 |
| 19318.02 | 0 | 0 |
| 1739.65 | 3 | 1 |
| 3354.43 | 10 | 1 |
| 1283.13 | 0 | 0 |
| 2618.73 | 2 | 10 |
| 1812.74 | 0 | 4 |
| 3293.72 | 0 | 3 |
| 2975.02 | 0 | 0 |
| 6727.5 | 4 | 6 |
| 2873.25 | 0 | 0 |
| 2989.13 | 3 | 0 |
| 7105.37 | 0 | 3 |
| 8957.75 | 0 | 11 |
| 15229.48 | 4 | 4 |
| 2311.76 | 0 | 0 |
| 1979.97 | 0 | 2 |
| 7131.94 | 3 | 1 |
| 8158.71 | 2 | 2 |
| 2669.9 | 0 | 0 |
| 13291.49 | 17 | 19 |
| 19170.16 | 14 | 14 |
| 52142.65 | 2 | 2 |
| 45631.12 | 0 | 0 |
| 28845.55 | 36 | 0 |
| 1790.2 | 0 | 0 |
| 1277.64 | 2 | 0 |
| 10052.54 | 7 | 0 |
| 2357.15 | 0 | 0 |
| 19236.34 | 18 | 1 |
| 11772.64 | 0 | 0 |
| 2865.56 | 0 | 0 |
| 1083.91 | 12 | 0 |
| 26829.06 | 12 | 0 |
| 10706.52 | 1 | 1 |
| 13254.64 | 23 | 2 |

Statistic

| | | Total Dis | Average S | Movement | Center Di | Periphery | Rearing | Hanging T |
|---|---|---|---|---|---|---|---|---|
| N | Valid N | 46 | 46 | 46 | 46 | 46 | 46 | 46 |
| | Missing N | 0 | 0 | 0 | 0 | 0 | 0 | 0 |
| Mean | | 12842.68 | 42.7509 | 189.99 | 695.0128 | 12147.66 | 6.1957 | 3.0652 |
| Percentil | 25 | 2818.33 | 9.3925 | 124.8425 | 0 | 2816.645 | 0 | 0 |
| | 50 | 9291.875 | 30.92 | 203.64 | 144.68 | 8805.53 | 2 | 0.5 |
| | 75 | 19331.38 | 64.2775 | 252.57 | 704.1825 | 19186.71 | 10.5 | 4 |
| | Interquar | 16513.05 | 54.885 | 127.7275 | 704.1825 | 16370.06 | 10.5 | 4 |

Percentile (P)

| | | Group | Percentile (P) | | | | | |
|---|---|---|---|---|---|---|---|---|
| | | | 5 | 10 | 25 | 50 | 75 | 90 |
| Weight | Total Dis | W-Sham | 5925.88 | 6974.05 | 10798.07 | 18763.65 | 24323.81 | 38281.31 |
| | | W-SⅠ | 1782.67 | 1809.733 | 2553.635 | 2982.075 | 7121.568 | 9797.708 |
| | | W-SⅡ | 1199.54 | 1383.297 | 2547.883 | 12788.86 | 27896.62 | 47680.51 |
| | Average S | W-Sham | 19.62 | 23.121 | 35.96 | 62.34 | 80.8075 | 127.179 |
| | | W-SⅠ | 5.94 | 6.03 | 8.49 | 9.94 | 23.74 | 32.662 |
| | | W-SⅡ | 4 | 4.609 | 8.4925 | 42.625 | 92.99 | 158.932 |
| | Movement | W-Sham | 99.67 | 115.009 | 209.2575 | 251.37 | 279.05 | 284.202 |
| | | W-SⅠ | 77.07 | 86.97 | 119.61 | 137.12 | 206.7775 | 247.946 |
| | | W-SⅡ | 57.51 | 64.594 | 119.6175 | 224.99 | 259.11 | 284.495 |
| | Center Di | W-Sham | 0 | 0 | 92.4625 | 550.625 | 769.6275 | 3551.735 |
| | | W-SⅠ | 0 | 0 | 0 | 9.74 | 442.285 | 1034.763 |
| | | W-SⅡ | 0 | 0 | 47.965 | 130.475 | 1726.043 | 5579.157 |
| | Periphery | W-Sham | 5781.84 | 6643.281 | 10350.45 | 18421.94 | 22865.27 | 36983.91 |
| | | W-SⅠ | 1283.13 | 1693.998 | 2228.813 | 2982.075 | 7112.013 | 9584.923 |
| | | W-SⅡ | 1083.91 | 1219.521 | 2484.253 | 12513.64 | 24930.88 | 47584.58 |
| | Rearing | W-Sham | 0 | 0 | 1.25 | 7 | 16.75 | 26.1 |
| | | W-SⅠ | 0 | 0 | 0 | 0 | 3 | 4.6 |
| | | W-SⅡ | 0 | 0 | 0 | 4.5 | 16.25 | 26.9 |
| | Hanging T | W-Sham | 0 | 0 | 0 | 0 | 9 | 17.4 |
| | | W-SⅠ | 0 | 0 | 0 | 1.5 | 4 | 10.1 |
| | | W-SⅡ | 0 | 0 | 0 | 0 | 1.75 | 15.5 |
| Tukey's H | Total Dis | W-Sham | | | 10857.95 | 18763.65 | 23754.33 | |
| | | W-SⅠ | | | 2603.79 | 2982.075 | 7118.11 | |
| | | W-SⅡ | | | 2653.775 | 12788.86 | 26762.87 | |
| | Average S | W-Sham | | | 36.13 | 62.34 | 78.915 | |
| | | W-SⅠ | | | 8.65 | 9.94 | 23.73 | |
| | | W-SⅡ | | | 8.845 | 42.625 | 89.21 | |
| | Movement | W-Sham | | | 215.455 | 251.37 | 277.73 | |
| | | W-SⅠ | | | 121.67 | 137.12 | 204.22 | |
| | | W-SⅡ | | | 124.005 | 224.99 | 258.13 | |
| | Center Di | W-Sham | | | 109.655 | 550.625 | 768.575 | |
| | | W-SⅠ | | | 0 | 9.74 | 423.2 | |
| | | W-SⅡ | | | 52.5 | 130.475 | 1250.765 | |
| | Periphery | W-Sham | | | 10474.43 | 18421.94 | 22585.46 | |
| | | W-SⅠ | | | 2311.76 | 2982.075 | 7105.37 | |

| | | | | |
|---|---|---|---|---|
| | W-SⅡ | 2611.355 | 12513.64 | 23032.7 |
| Rearing | W-Sham | 1.5 | 7 | 15.5 |
| | W-SⅠ | 0 | 0 | 3 |
| | W-SⅡ | 0 | 4.5 | 15.5 |
| Hanging T | W-Sham | 0 | 0 | 8 |
| | W-SⅠ | 0 | 1.5 | 4 |
| | W-SⅡ | 0 | 0 | 1.5 |

`ime

95

Report

| Group | | | Total Dis |
|---|---|---|---|
| | 1 | Mean | 18761.87 |
| | | N | 12 |
| | | Standard | 10200.91 |
| | | Median | 18763.65 |
| | | Median by | 18763.65 |
| | 2 | Mean | 4891.387 |
| | | N | 18 |
| | | Standard | 3631.659 |
| | | Median | 2982.075 |
| | | Median by | 2982.075 |
| | 3 | Mean | 17348.48 |
| | | N | 16 |
| | | Standard | 16009.93 |
| | | Median | 12788.86 |
| | | Median by | 12788.86 |
| Total | | Mean | 12842.68 |
| | | N | 46 |
| | | Standard | 12558.24 |
| | | Median | 9291.875 |
| | | Median by | 9291.875 |

| Average S | Center Distance |
|---|---|
| 62.335 | 822.4442 |
| 12 | 12 |
| 33.8892 | 1205.727 |
| 62.34 | 550.625 |
| 62.34 | 550.625 |
| 16.3006 | 268.4878 |
| 18 | 18 |
| 12.10769 | 403.4982 |
| 9.94 | 9.74 |
| 9.94 | 9.74 |
| 57.8194 | 1079.28 |
| 16 | 16 |
| 53.36948 | 1987.084 |
| 42.625 | 130.475 |
| 42.625 | 130.475 |
| 42.7509 | 695.0128 |
| 46 | 46 |
| 41.814 | 1364.787 |
| 30.92 | 144.68 |
| 30.92 | 144.68 |

| | 1 | 2 | 3 | 4 | 5 | 6 | 7 |
|---|---|---|---|---|---|---|---|
| A | UD | UD | UD | UD | UD | UD | UD |
| B | UD | UD | UD | UD | UD | UD | UD |
| C | UD | UD | UD | UD | UD | UD | UD |
| D | UD | UD | UD | UD | UD | UD | UD |
| E | UD | UD | UD | UD | UD | UD | UD |
| F | UD | UD | UD | UD | UD | UD | UD |
| G | UD | UD | UD | UD | UD | UD | UD |
| H | UD | UD | UD | UD | UD | UD | UD |

Absorbance Data:
Filter1:600nm

| | 1 | 2 | 3 | 4 | 5 | 6 | 7 |
|---|---|---|---|---|---|---|---|
| 读数: 1 | | | | | | | |
| A | 0.407 | 0.529 | 0.412 | 0.431 | 0.422 | 0.394 | 0.341 |
| B | 0.327 | 0.413 | 0.393 | 0.367 | 0.498 | 0.476 | 0.351 |
| C | 0.486 | 0.522 | 0.477 | 0.655 | 0.676 | 0.592 | 0.47 |
| D | 0.597 | 0.523 | 0.694 | 0.861 | 0.476 | 0.443 | 0.504 |
| E | ~~1.659~~ | ~~1.654~~ | ~~1.62~~ | 0.451 | 0.569 | 0.448 | 0.394 |
| F | 0.452 | 1.309 | 1.262 | 0.508 | 0.482 | 0.558 | 0.501 |
| G | 0.482 | 0.557 | 0.67 | 0.751 | 0.787 | 0.687 | 0.885 |
| H | 0.51 | 0.488 | 0.599 | 0.477 | 0.396 | 0.427 | 0.353 |

Quantitative Analysis Results (Concentration)
Filter1: 600nm

| | 1 | 2 | 3 | 4 | 5 | 6 | 7 |
|---|---|---|---|---|---|---|---|
| A | 0 | 0 | 0 | 0 | 0 | 0 | 0 |
| B | 0 | 0 | 0 | 0 | 0 | 0 | 0 |
| C | 0 | 0 | 0 | 0 | 0 | 0 | 0 |
| D | 0 | 0 | 0 | 0 | 0 | 0 | 0 |
| E | 0 | 0 | 0 | 0 | 0 | 0 | 0 |
| F | 0 | 0 | 0 | 0 | 0 | 0 | 0 |
| G | 0 | 0 | 0 | 0 | 0 | 0 | 0 |
| H | 0 | 0 | 0 | 0 | 0 | 0 | 0 |

| 8 | 9 | 10 | 11 | 12 |
|---|---|---|---|---|
| UD | UD | UD | UD | UD |
| UD | UD | UD | UD | UD |
| UD | UD | UD | UD | UD |
| UD | UD | UD | UD | UD |
| UD | UD | UD | UD | UD |
| UD | UD | UD | UD | UD |
| UD | UD | UD | UD | UD |
| UD | UD | UD | UD | UD |

| 8 | 9 | 10 | 11 | 12 |
|---|---|---|---|---|
| 0.3 | 0.291 | 0.297 | 0.357 | 0.373 |
| 0.429 | 0.622 | 0.372 | 0.409 | 0.418 |
| 0.688 | 0.511 | 0.48 | 0.333 | 0.34 |
| 0.679 | 0.654 | 0.543 | 0.5 | 0.437 |
| 0.613 | 0.62 | ~~0.603~~ | ~~1.592~~ | ~~1.444~~ |
| 0.569 | 0.531 | 0.357 | 0.371 | 0.348 |
| 0.719 | 0.663 | 0.615 | 0.599 | 0.342 |
| 0.187 | 0.04 | 0.04 | 0.039 | 0.039 |

| 8 | 9 | 10 | 11 | 12 |
|---|---|---|---|---|
| 0 | 0 | 0 | 0 | 0 |
| 0 | 0 | 0 | 0 | 0 |
| 0 | 0 | 0 | 0 | 0 |
| 0 | 0 | 0 | 0 | 0 |
| 0 | 0 | 0 | 0 | 0 |
| 0 | 0 | 0 | 0 | 0 |
| 0 | 0 | 0 | 0 | 0 |
| 0 | 0 | 0 | 0 | 0 |

| | | | | | | | | |
|---|---|---|---|---|---|---|---|---|
| 3.445783 | 5.356627 | 3.524096 | 3.821687 | 3.680723 | 3.242169 | 2.412048 | 1.76988 | 1.628916 |
| 2.192771 | 3.539759 | 3.226506 | 2.819277 | 4.871084 | 4.526506 | 2.568675 | 3.790361 | 6.813253 |
| 7.609119 | 5.246988 | 4.542169 | 7.33012 | 7.659036 | 6.343373 | 4.43253 | 7.846988 | 5.074699 |
| 6.421687 | 5.262651 | 7.940964 | 10.55663 | 4 526506 | 4.009639 | 4.96506 | 7.706024 | 7.314458 |
| ~~23.05542~~ | ~~22.97711~~ | ~~22.44458~~ | 4.13494 | 5.983133 | 4.087952 | 3.242169 | 6.672289 | 6.781928 |
| 4.150602 | 17.57349 | 16.83735 | 5.027711 | 4.620482 | 5.810843 | 4.918072 | 5.983133 | 5.387952 |
| 4.620482 | 5.795181 | 7.56506 | 8.833735 | 9.39759 | 7.831325 | 10.93253 | 8.33253 | 7.455422 |
| 5.059036 | 4.714458 | 6.453012 | 4.542169 | 3.273494 | 3.759036 | 2.6 | 0 | |

| | | | | | |
|---|---|---|---|---|---|
| 3.445783 | 5.356627 | 3.524096 | 4.108835333 | 4.1088353 | 2 |
| 3.821687 | 3.680723 | 3.242169 | 3.581526333 | 3.5815263 | 3 |
| 2.412048 | 1.76988 | 1.628916 | 1.936948 | 1.936948 | 1 |
| 1.722892 | 2.662651 | 2.91325 | 2.432931 | 2.432931 | 2 |
| 2.192771 | 3.539759 | 3.226506 | 2.986345333 | 2.9863453 | 3 |
| 2.819277 | 4.871084 | 4.526506 | 4.072289 | 4.072289 | 1 |
| 2.568675 | 3.790361 | 6.813253 | 4.390763 | 4.390763 | 2 |
| 2.89759 | 3.477108 | 3.61807 | 3.330922667 | 3.3309227 | 3 |
| 7.609119 | 5.246988 | 4.542169 | 5.799425333 | 5.7994253 | 1 |
| 7.33012 | 7.659036 | 6.343373 | 7.110843 | 7.110843 | 2 |
| | | | | 5.784739 | 3 |
| 4.43253 | 7.846988 | 5.074699 | 5.784739 | 3.0907613 | 1 |
| 4.589157 | 2.286747 | 2.39638 | 3.090761333 | 6.5417673 | 2 |
| 6.421687 | 5.262651 | 7.940964 | 6.541767333 | 6.3642583 | 3 |
| 10.55663 | 4.526506 | 4.009639 | 6.364258333 | 6.6618473 | 1 |
| 4.96506 | 7.706024 | 7.314458 | 6.661847333 | 4.7979913 | 2 |
| 5.575904 | 4.90241 | 3.91566 | 4.797991333 | 4.7353417 | 3 |
| 4.13494 | 5.983133 | 4.087952 | 4.735341667 | 5.565462 | 1 |
| 3.242169 | 6.672289 | 6.781928 | 5.565462 | 12.853814 | 2 |
| | | | | 5.153012 | 3 |
| | | | | 5.429719 | 1 |
| 4.150602 | 17.57349 | 16.83735 | 12.853814 | 2.688753 | 2 |
| 5.027711 | 4.620482 | 5.810843 | 5.153012 | 5.9935743 | 3 |
| 4.918072 | 5.983133 | 5.387952 | 5.429719 | 8.68755 | 1 |
| 2.662651 | 2.881928 | 2.52168 | 2.688753 | 8.9068273 | 2 |
| 4.620482 | 5.795181 | 7.56506 | 5.993574333 | 5.1947787 | 3 |
| 8.833735 | 9.39759 | 7.831325 | 8.68755 | 5.4088353 | 2 |
| 10.93253 | 8.33253 | 7.455422 | 8.906827333 | 3.858233 | 3 |
| 6.703614 | 6.453012 | 2.42771 | 5.194778667 | | |
| 5.059036 | 4.714458 | 6.453012 | 5.408835333 | | |
| 4.542169 | 3.273494 | 3.759036 | 3.858233 | | |

| | | |
|---|---|---|
| 1.722892 | 2.662651 | 2.91325 |
| 2.89759 | 3.477108 | 3.61807 |
| 4.589157 | 2.286747 | 2.39638 |
| 5.575904 | 4.90241 | 3.91566 |
| 6.515663 | 22.00602 | 19.6879 |
| 2.662651 | 2.881928 | 2.52168 |
| 6.703614 | 6.453012 | 2.42771 |
| | | |

| | | | | | |
|---|---|---|---|---|---|
| 4.108835 | 2 | 3.581526 | 3 | 1.936948 | 1 |
| 2.432931 | 2 | 2.986345 | 3 | 4.072289 | 1 |
| 4.390763 | 2 | 3.330923 | 3 | 5.799425 | 1 |
| 7.110843 | 2 | 5.784739 | 3 | 3.090761 | 1 |
| 6.541767 | 2 | 6.364258 | 3 | 6.661847 | 1 |
| 4.797991 | 2 | 4.735342 | 3 | 5.565462 | 1 |
| 12.85381 | 2 | 5.153012 | 3 | 5.429719 | 1 |
| 2.688753 | 2 | 5.993574 | 3 | 8.68755 | 1 |
| 8.906827 | 2 | 5.194779 | 3 | | |
| 5.408835 | 2 | 3.858233 | 3 | | |

Report

P

| Group P | Mean | Std. Deviation |
|---|---|---|
| 1 | 5.1555 | 2.111125 |
| 2 | 5.924136 | 3.136579 |
| 3 | 4.698273 | 1.194503 |
| Total | 5.266718 | 2.279009 |

Tests of Normality

| | Group P | Kolmogorov-Smirnova | | | Shapiro-Wilk | | |
|---|---|---|---|---|---|---|---|
| | | Statisti | df | Sig. | Statisti | df | Sig. |
| P | 1 | 0.177 | 8 | .200* | 0.978 | 8 | 0.953 |
| | 2 | 0.165 | 10 | .200* | 0.906 | 10 | 0.254 |
| | 3 | 0.159 | 10 | .200* | 0.936 | 10 | 0.51 |

* This is a lower bound of the true significance.

a Lilliefors Significance Correction

ANOVA

P

| | Sum of Sc | df | Mean Squa | F | Sig. |
|---|---|---|---|---|---|
| Between ( | 7.652 | 2 | 3.826 | 0.721 | 0.496 |
| Within Gı | 132.583 | 25 | 5.303 | | |
| Total | 140.235 | 27 | | | |

Multiple Comparisons

Dependent Variable: P

Tukey HSD

| (I) Grouı | (J) Grouı | Mean Dif | Std. Erro | Sig. | 95% Confidence Interval | |
|---|---|---|---|---|---|---|
| | | | | | Lower Bou | Upper Bound |
| 1 | 2 | -0.76864 | 1.092357 | 0.764 | -3.48951 | 1.952239 |
| | 3 | 0.457227 | 1.092357 | 0.908 | -2.26365 | 3.178102 |
| 2 | 1 | 0.768636 | 1.092357 | 0.764 | -1.95224 | 3.489511 |
| | 3 | 1.225863 | 1.029884 | 0.47 | -1.3394 | 3.791129 |
| 3 | 1 | -0.45723 | 1.092357 | 0.908 | -3.1781 | 2.263648 |
| | 2 | -1.22586 | 1.029884 | 0.47 | -3.79113 | 1.339403 |

Absorbance Data:
Filter1:450nm

| | 1 | 2 | 3 | 4 | 5 | 6 | 7 | 8 |
|---|---|---|---|---|---|---|---|---|
| A | 0.217 | 0.343 | 0.733 | 1.011 | 1.245 | 0.039 | 0.404 | 0.393 |
| B | 0.205 | 0.367 | 0.653 | 1.007 | 1.214 | 0.04 | 0.385 | 0.542 |
| C | 0.494 | 0.568 | 0.422 | 0.429 | 0.313 | 0.234 | 0.446 | 0.327 |
| D | 0.604 | 0.173 | 0.441 | 0.406 | 0.452 | 0.425 | 0.299 | 0.263 |
| E | 0.464 | 0.431 | 0.337 | 0.352 | 0.327 | 0.246 | 0.286 | 0.423 |
| F | 0.454 | 0.494 | 0.515 | 0.29 | 0.396 | 0.391 | 0.458 | 0.051 |
| G | 0.45 | 0.371 | 0.457 | 0.471 | 0.43 | 0.295 | 0.397 | 0.325 |
| H | 0.458 | 0.588 | 0.433 | 0.413 | 0.43 | 0.3 | 0.232 | 0.372 |

| 9 | 10 | 11 | 12 |
|---|---|---|---|
| 0.37 | 0.363 | 0.416 | 0.394 |
| 0.361 | 0.318 | 0.308 | 0.381 |
| 0.178 | 0.242 | 0.118 | 0.227 |
| 0.081 | 0.3 | 0.267 | 0.349 |
| 0.232 | 0.141 | 0.242 | 0.289 |
| 0.38 | 0.305 | 0.264 | 0.301 |
| 0.046 | 0.343 | 0.165 | 0.16 |
| 0.063 | 0.36 | 0.264 | 0.284 |

| | | | | | |
|---|---|---|---|---|---|
| 0.217 | 0.343 | 0.733 | 1.011 | 1.245 | 0.039 |
| 0.178 | 0.304 | 0.694 | 0.972 | 1.206 | 0 |
| 3 | 6 | 12 | 24 | 48 | |

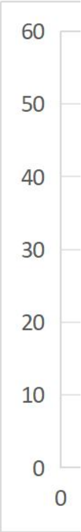


| | | | | | |
|---|---|---|---|---|---|
| 0.205 | 0.367 | 0.653 | 1.007 | 1.214 | 0.04 |
| 0.165 | 0.327 | 0.613 | 0.967 | 1.174 | 0 |
| 3 | 6 | 12 | 24 | 48 | |

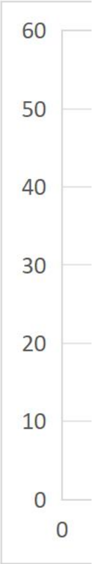


| Sample ID | OD （450n | Blank Cor | Dilution | Concentration | | |
|---|---|---|---|---|---|---|
| 1 | 0.404 | 0.365 | 3.694984 | 18.47492 | 22.3853 | 20.43011 |
| 2 | 0.393 | 0.354 | 3.657163 | 18.28581 | 21.91787 | 20.10184 |
| 3 | 0.37 | 0.331 | 3.621783 | 18.10892 | 21.14143 | 19.62517 |
| 4 | 0.363 | 0.324 | 3.622752 | 18.11376 | 20.95907 | 19.53642 |
| 5 | 0.416 | 0.377 | 3.751668 | 18.75834 | 22.96614 | 20.86224 |
| 6 | 0.394 | 0.355 | 3.660042 | 18.30021 | 21.95779 | 20.129 |
| 7 | 0.385 | 0.346 | 3.638151 | 18.19075 | 21.61697 | 19.90386 |
| 8 | 0.542 | 0.503 | 5.318579 | 26.59289 | 33.53244 | 30.06267 |
| 9 | 0.361 | 0.322 | 3.624034 | 18.12017 | 20.9116 | 19.51589 |
| 10 | 0.318 | 0.279 | 3.759747 | 18.79874 | 20.38803 | 19.59338 |
| 11 | 0.308 | 0.269 | 3.820927 | 19.10464 | 20.40244 | 19.75354 |
| 12 | 0.381 | 0.342 | 3.631327 | 18.15664 | 21.47885 | 19.81774 |
| 13 | 0.494 | 0.455 | 4.512427 | 22.56214 | 28.54523 | 25.55368 |
| 14 | 0.568 | 0.529 | 5.862767 | 29.31384 | 36.72817 | 33.021 |
| 15 | 0.422 | 0.383 | 3.786046 | 18.93023 | 23.2843 | 21.10727 |
| 16 | 0.429 | 0.39 | 3.831239 | 19.15619 | 23.67888 | 21.41754 |
| 17 | 0.313 | 0.274 | 3.78894 | 18.9447 | 20.38881 | 19.66676 |
| 18 | 0.234 | 0.195 | 4.621042 | 23.10521 | 22.10617 | 22.60569 |
| 19 | 0.446 | 0.407 | 3.963793 | 19.81897 | 24.74196 | 22.28047 |
| 20 | 0.327 | 0.288 | 3.714241 | 18.57121 | 20.41899 | 19.4951 |
| 21 | 0.178 | 0.139 | 5.633377 | 28.16689 | 25.26592 | 26.71641 |

| | | | | | | |
|---|---|---|---|---|---|---|
| 22 | 0.242 | 0.203 | 4.505036 | 22.52518 | 21.78632 | 22.15575 |
| 23 | 0.118 | 0.079 | 7.106981 | 35.53491 | 30.43961 | 32.98726 |
| 24 | 0.227 | 0.188 | 4.728415 | 23.64208 | 22.41301 | 23.02754 |
| 25 | 0.604 | 0.565 | 6.740994 | 33.70497 | 41.72651 | 37.71574 |
| 26 | 0.173 | 0.134 | 5.740809 | 28.70405 | 25.62641 | 27.16523 |
| 27 | 0.441 | 0.402 | 3.921454 | 19.60727 | 24.41388 | 22.01057 |
| 28 | 0.406 | 0.367 | 3.703314 | 18.51657 | 22.47697 | 20.49677 |
| 29 | 0.452 | 0.413 | 4.01829 | 20.09145 | 25.15263 | 22.62204 |
| 30 | 0.425 | 0.386 | 3.804743 | 19.02372 | 23.45032 | 21.23702 |
| 31 | 0.299 | 0.26 | 3.885546 | 19.42773 | 20.45935 | 19.94354 |
| 32 | 0.263 | 0.224 | 4.234554 | 21.17277 | 21.1032 | 21.13798 |
| 33 | 0.081 | 0.042 | 8.216275 | 41.08138 | 34.55217 | 37.81678 |
| 34 | 0.3 | 0.261 | 3.877919 | 19.3896 | 20.45097 | 19.92028 |
| 35 | 0.267 | 0.228 | 4.188622 | 20.94311 | 20.99877 | 20.97094 |
| 36 | 0.349 | 0.31 | 3.641119 | 18.20559 | 20.66991 | 19.43775 |
| 37 | 0.464 | 0.425 | 4.139353 | 20.69677 | 26.02945 | 23.36311 |
| 38 | 0.431 | 0.392 | 3.845157 | 19.22578 | 23.79624 | 21.51101 |
| 39 | 0.337 | 0.298 | 3.674298 | 18.37149 | 20.50221 | 19.43685 |
| 40 | 0.352 | 0.313 | 3.635339 | 18.17669 | 20.72339 | 19.45004 |
| 41 | 0.327 | 0.288 | 3.714241 | 18.57121 | 20.41899 | 19.4951 |
| 42 | 0.246 | 0.207 | 4.449715 | 22.24858 | 21.63873 | 21.94365 |
| 43 | 0.286 | 0.247 | 3.994867 | 19.97433 | 20.61503 | 20.29468 |
| 44 | 0.423 | 0.384 | 3.792167 | 18.96083 | 23.33913 | 21.14998 |
| 45 | 0.232 | 0.193 | 4.651161 | 23.25581 | 22.19127 | 22.72354 |
| 46 | 0.141 | 0.102 | 6.494542 | 32.47271 | 28.23772 | 30.35521 |
| 47 | 0.242 | 0.203 | 4.505036 | 22.52518 | 21.78632 | 22.15575 |
| 48 | 0.289 | 0.25 | 3.967963 | 19.83981 | 20.57139 | 20.2056 |
| 49 | 0.454 | 0.415 | 4.037349 | 20.18675 | 25.29362 | 22.74018 |
| 50 | 0.494 | 0.455 | 4.512427 | 22.56214 | 28.54523 | 25.55368 |
| 51 | 0.515 | 0.476 | 4.833432 | 24.16716 | 30.58146 | 27.37431 |
| 52 | 0.29 | 0.251 | 3.959218 | 19.79609 | 20.55788 | 20.17698 |
| 53 | 0.396 | 0.357 | 3.666136 | 18.33068 | 22.03918 | 20.18493 |
| 54 | 0.391 | 0.352 | 3.651739 | 18.2587 | 21.83956 | 20.04913 |
| 55 | 0.458 | 0.419 | 4.076809 | 20.38405 | 25.58179 | 22.98292 |
| 56 | 0.051 | 0.012 | 9.228031 | 46.14016 | 38.40311 | 42.27164 |
| 57 | 0.38 | 0.341 | 3.629901 | 18.1495 | 21.44561 | 19.79756 |
| 58 | 0.305 | 0.266 | 3.841461 | 19.20731 | 20.41678 | 19.81204 |
| 59 | 0.264 | 0.225 | 4.222903 | 21.11452 | 21.07632 | 21.09542 |
| 60 | 0.301 | 0.262 | 3.870404 | 19.35202 | 20.4431 | 19.89756 |
| 61 | 0.45 | 0.411 | 3.999677 | 19.99839 | 25.01368 | 22.50603 |
| 62 | 0.371 | 0.332 | 3.622092 | 18.11046 | 21.16953 | 19.64 |
| 63 | 0.457 | 0.418 | 4.066777 | 20.33388 | 25.50898 | 22.92143 |
| 64 | 0.471 | 0.432 | 4.217406 | 21.08703 | 26.5751 | 23.83106 |
| 65 | 0.43 | 0.391 | 3.838142 | 19.19071 | 23.7373 | 21.46401 |
| 66 | 0.295 | 0.256 | 3.917171 | 19.58586 | 20.498 | 20.04193 |
| 67 | 0.397 | 0.358 | 3.669351 | 18.34676 | 22.08065 | 20.2137 |
| 68 | 0.325 | 0.286 | 3.723571 | 18.61786 | 20.40851 | 19.51319 |
| 69 | 0.046 | 0.007 | 9.406437 | 47.03219 | 39.0899 | 43.06104 |
| 70 | 0.343 | 0.304 | 3.655696 | 18.27848 | 20.57681 | 19.42765 |
| 71 | 0.165 | 0.126 | 5.918512 | 29.59256 | 26.22991 | 27.91123 |
| 72 | 0.16 | 0.121 | 6.033209 | 30.16605 | 26.62379 | 28.39492 |
| 73 | 0.458 | 0.419 | 4.076809 | 20.38405 | 25.58179 | 22.98292 |
| 74 | 0.588 | 0.549 | 6.332788 | 31.66394 | 39.42281 | 35.54337 |
| 75 | 0.433 | 0.394 | 3.859522 | 19.29761 | 23.91566 | 21.60663 |

| | | | | | | |
|---|---|---|---|---|---|---|
| 76 | 0.413 | 0.374 | 3.735988 | 18.67994 | 22.81399 | 20.74697 |
| 77 | 0.43 | 0.391 | 3.838142 | 19.19071 | 23.7373 | 21.46401 |
| 78 | 0.3 | 0.261 | 3.877919 | 19.3896 | 20.45097 | 19.92028 |
| 79 | 0.232 | 0.193 | 4.651161 | 23.25581 | 22.19127 | 22.72354 |
| 80 | 0.372 | 0.333 | 3.622513 | 18.11256 | 21.19815 | 19.65536 |
| 81 | 0.063 | 0.024 | 8.811258 | 44.05629 | 36.80724 | 40.43176 |
| 82 | 0.36 | 0.321 | 3.624843 | 18.12422 | 20.88863 | 19.50642 |
| 83 | 0.264 | 0.225 | 4.222903 | 21.11452 | 21.07632 | 21.09542 |
| 84 | 0.284 | 0.245 | 4.013362 | 20.06681 | 20.64668 | 20.35675 |

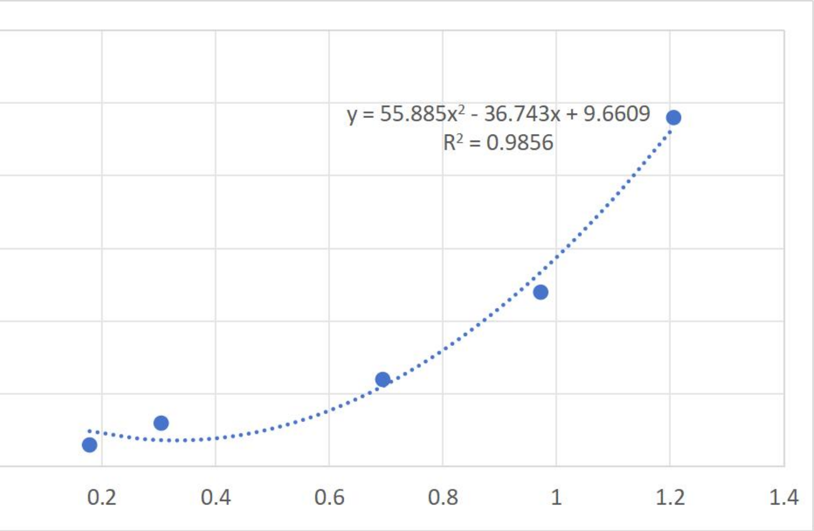


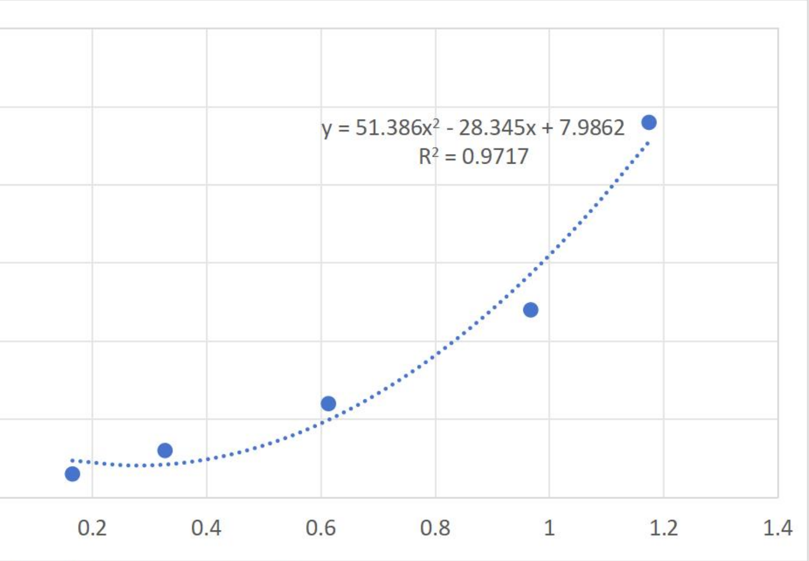


| Blank Cor | Dilution | Concentration | | | | | |
|---|---|---|---|---|---|---|---|
| 0.364 | 4.477059 | 22.38529728 | 18.47492 | 22.3853 | 20.43011 | 1 | 20.43011 |
| 0.353 | 4.383573 | 21.91786537 | 18.28581 | 21.91787 | 20.10184 | | 20.10184 |
| 0.33 | 4.228285 | 21.141427 | 18.10892 | 21.14143 | 19.62517 | | 19.62517 |
| 0.323 | 4.191815 | 20.95907497 | 18.11376 | 20.95907 | 19.53642 | 2 | 19.53642 |
| 0.376 | 4.593227 | 22.96613568 | 18.75834 | 22.96614 | 20.86224 | | 20.86224 |
| 0.354 | 4.391558 | 21.95778988 | 18.30021 | 21.95779 | 20.129 | | 20.129 |
| 0.345 | 4.323394 | 21.61696825 | 18.19075 | 21.61697 | 19.90386 | 3 | 19.90386 |
| 0.502 | 6.706488 | 33.53243772 | 26.59289 | 33.53244 | 30.06267 | | 30.06267 |
| 0.321 | 4.18232 | 20.91159913 | 18.12017 | 20.9116 | 19.51589 | | 19.51589 |
| 0.278 | 4.077606 | 20.38802812 | 18.79874 | 20.38803 | 19.59338 | 4 | 19.59338 |
| 0.268 | 4.080488 | 20.40244032 | 19.10464 | 20.40244 | 19.75354 | | 19.75354 |
| 0.341 | 4.29577 | 21.47885233 | 18.15664 | 21.47885 | 19.81774 | | 19.81774 |
| 0.454 | 5.709047 | 28.54523388 | 22.56214 | 28.54523 | 25.55368 | 5 | 25.55368 |
| 0.528 | 7.345635 | 36.72817312 | 29.31384 | 36.72817 | 33.021 | | 33.021 |
| 0.382 | 4.656861 | 23.28430332 | 18.93023 | 23.2843 | 21.10727 | | 21.10727 |
| 0.389 | 4.735776 | 23.67887953 | 19.15619 | 23.67888 | 21.41754 | 6 | 21.41754 |
| 0.273 | 4.077762 | 20.38881097 | 18.9447 | 20.38881 | 19.66676 | | 19.66676 |
| 0.194 | 4.421233 | 22.10616748 | 23.10521 | 22.10617 | 22.60569 | | 22.60569 |
| 0.406 | 4.948393 | 24.74196348 | 19.81897 | 24.74196 | 22.28047 | 7 | 22.28047 |
| 0.287 | 4.083798 | 20.41899217 | 18.57121 | 20.41899 | 19.4951 | | 19.4951 |
| 0.138 | 5.053185 | 25.26592492 | 28.16689 | 25.26592 | 26.71641 | | 26.71641 |

| | | | | | | | |
|---|---|---|---|---|---|---|---|
| 0.202 | 4.357264 | 21.78632172 | 22.52518 | 21.78632 | 22.15575 | 8 | 22.15575 |
| 0.078 | 6.087922 | 30.43961212 | 35.53491 | 30.43961 | 32.98726 | | 32.98726 |
| 0.187 | 4.482602 | 22.41301017 | 23.64208 | 22.41301 | 23.02754 | | 23.02754 |
| 0.564 | 8.345301 | 41.72650528 | 33.70497 | 41.72651 | 37.71574 | 9 | 37.71574 |
| 0.133 | 5.125282 | 25.62640977 | 28.70405 | 25.62641 | 27.16523 | | 27.16523 |
| 0.401 | 4.882775 | 24.41387593 | 19.60727 | 24.41388 | 22.01057 | | 22.01057 |
| 0.366 | 4.495393 | 22.47696508 | 18.51657 | 22.47697 | 20.49677 | 10 | 20.49677 |
| 0.412 | 5.030525 | 25.15262592 | 20.09145 | 25.15263 | 22.62204 | | 22.62204 |
| 0.385 | 4.690065 | 23.45032425 | 19.02372 | 23.45032 | 21.23702 | | 21.23702 |
| 0.259 | 4.091869 | 20.45934633 | 19.42773 | 20.45935 | 19.94354 | 11 | 19.94354 |
| 0.223 | 4.220639 | 21.10319697 | 21.17277 | 21.1032 | 21.13798 | | 21.13798 |
| 0.041 | 6.910435 | 34.55217433 | 41.08138 | 34.55217 | 37.81678 | | 37.81678 |
| 0.26 | 4.090194 | 20.450968 | 19.3896 | 20.45097 | 19.92028 | 12 | 19.92028 |
| 0.227 | 4.199754 | 20.99877097 | 20.94311 | 20.99877 | 20.97094 | | 20.97094 |
| 0.309 | 4.133982 | 20.66990833 | 18.20559 | 20.66991 | 19.43775 | | 19.43775 |
| 0.424 | 5.20589 | 26.02944768 | 20.69677 | 26.02945 | 23.36311 | 13 | 23.36311 |
| 0.391 | 4.759248 | 23.79624033 | 19.22578 | 23.79624 | 21.51101 | | 21.51101 |
| 0.297 | 4.100443 | 20.50221337 | 18.37149 | 20.50221 | 19.43685 | | 19.43685 |
| 0.312 | 4.144679 | 20.72339392 | 18.17669 | 20.72339 | 19.45004 | 14 | 19.45004 |
| 0.287 | 4.083798 | 20.41899217 | 18.57121 | 20.41899 | 19.4951 | | 19.4951 |
| 0.206 | 4.327746 | 21.63873148 | 22.24858 | 21.63873 | 21.94365 | | 21.94365 |
| 0.246 | 4.123005 | 20.61502588 | 19.97433 | 20.61503 | 20.29468 | 15 | 20.29468 |
| 0.383 | 4.667826 | 23.33912977 | 18.96083 | 23.33913 | 21.14998 | | 21.14998 |
| 0.192 | 4.438254 | 22.19126752 | 23.25581 | 22.19127 | 22.72354 | | 22.72354 |
| 0.101 | 5.647544 | 28.23771793 | 32.47271 | 28.23772 | 30.35521 | 16 | 30.35521 |
| 0.202 | 4.357264 | 21.78632172 | 22.52518 | 21.78632 | 22.15575 | | 22.15575 |
| 0.249 | 4.114278 | 20.57139193 | 19.83981 | 20.57139 | 20.2056 | | 20.2056 |
| 0.414 | 5.058725 | 25.29362428 | 20.18675 | 25.29362 | 22.74018 | 17 | 22.74018 |
| 0.454 | 5.709047 | 28.54523388 | 22.56214 | 28.54523 | 25.55368 | | 25.55368 |
| 0.475 | 6.116291 | 30.58145625 | 24.16716 | 30.58146 | 27.37431 | | 27.37431 |
| 0.25 | 4.111575 | 20.557875 | 19.79609 | 20.55788 | 20.17698 | 18 | 20.17698 |
| 0.356 | 4.407836 | 22.03918048 | 18.33068 | 22.03918 | 20.18493 | | 20.18493 |
| 0.351 | 4.367912 | 21.83955793 | 18.2587 | 21.83956 | 20.04913 | | 20.04913 |
| 0.418 | 5.116357 | 25.58178732 | 20.38405 | 25.58179 | 22.98292 | 19 | 22.98292 |
| 0.011 | 7.680623 | 38.40311353 | 46.14016 | 38.40311 | 42.27164 | | 42.27164 |
| 0.34 | 4.289122 | 21.445608 | 18.1495 | 21.44561 | 19.79756 | | 19.79756 |
| 0.265 | 4.083357 | 20.41678425 | 19.20731 | 20.41678 | 19.81204 | 20 | 19.81204 |
| 0.224 | 4.215264 | 21.07631968 | 21.11452 | 21.07632 | 21.09542 | | 21.09542 |
| 0.261 | 4.088621 | 20.44310353 | 19.35202 | 20.4431 | 19.89756 | | 19.89756 |
| 0.41 | 5.002737 | 25.013683 | 19.99839 | 25.01368 | 22.50603 | 21 | 22.50603 |
| 0.331 | 4.233907 | 21.16953273 | 18.11046 | 21.16953 | 19.64 | | 19.64 |
| 0.417 | 5.101795 | 25.50897577 | 20.33388 | 25.50898 | 22.92143 | | 22.92143 |
| 0.431 | 5.31502 | 26.57509873 | 21.08703 | 26.5751 | 23.83106 | 22 | 23.83106 |
| 0.39 | 4.747461 | 23.737303 | 19.19071 | 23.7373 | 21.46401 | | 21.46401 |
| 0.255 | 4.0996 | 20.49799825 | 19.58586 | 20.498 | 20.04193 | | 20.04193 |
| 0.357 | 4.416129 | 22.08064657 | 18.34676 | 22.08065 | 20.2137 | 23 | 20.2137 |
| 0.285 | 4.081703 | 20.40851425 | 18.61786 | 20.40851 | 19.51319 | | 19.51319 |
| 0.006 | 7.81798 | 39.08989948 | 47.03219 | 39.0899 | 43.06104 | | 43.06104 |
| 0.303 | 4.115362 | 20.57681137 | 18.27848 | 20.57681 | 19.42765 | 24 | 19.42765 |
| 0.125 | 5.245981 | 26.22990625 | 29.59256 | 26.22991 | 27.91123 | | 27.91123 |
| 0.12 | 5.324758 | 26.623792 | 30.16605 | 26.62379 | 28.39492 | | 28.39492 |
| 0.418 | 5.116357 | 25.58178732 | 20.38405 | 25.58179 | 22.98292 | 25 | 22.98292 |
| 0.548 | 7.884561 | 39.42280672 | 31.66394 | 39.42281 | 35.54337 | | 35.54337 |
| 0.393 | 4.783131 | 23.91565657 | 19.29761 | 23.91566 | 21.60663 | | 21.60663 |

| | | | | | | | |
|---|---|---|---|---|---|---|---|
| 0.373 | 4.562798 | 22.81398897 | 18.67994 | 22.81399 | 20.74697 | 26 | 20.74697 |
| 0.39 | 4.747461 | 23.737303 | 19.19071 | 23.7373 | 21.46401 | | 21.46401 |
| 0.26 | 4.090194 | 20.450968 | 19.3896 | 20.45097 | 19.92028 | | 19.92028 |
| 0.192 | 4.438254 | 22.19126752 | 23.25581 | 22.19127 | 22.72354 | 27 | 22.72354 |
| 0.332 | 4.23963 | 21.19815232 | 18.11256 | 21.19815 | 19.65536 | | 19.65536 |
| 0.023 | 7.361448 | 36.80724097 | 44.05629 | 36.80724 | 40.43176 | | 40.43176 |
| 0.32 | 4.177726 | 20.888632 | 18.12422 | 20.88863 | 19.50642 | 28 | 19.50642 |
| 0.224 | 4.215264 | 21.07631968 | 21.11452 | 21.07632 | 21.09542 | | 21.09542 |
| 0.244 | 4.129337 | 20.64668448 | 20.06681 | 20.64668 | 20.35675 | | 20.35675 |

| | | | | | |
|---|---|---|---|---|---|
| 20.43011 | 20.10184 | 19.62517 | 20.05237 | 1 | 1 |
| 19.53642 | 20.86224 | 20.129 | 20.17589 | 2 | 1 |
| 19.90386 | 30.06267 | 19.51589 | 23.1608 | 3 | 1 |
| 19.59338 | 19.75354 | 19.81774 | 19.72156 | 4 | 1 |
| 25.55368 | 33.021 | 21.10727 | 26.56065 | 5 | 1 |
| 21.41754 | 19.66676 | 22.60569 | 21.22999 | 6 | 1 |
| 22.28047 | 19.4951 | 26.71641 | 22.83066 | 7 | 1 |
| 22.15575 | 32.98726 | 23.02754 | 26.05685 | 8 | 1 |
| 37.71574 | 27.16523 | 22.01057 | 28.96385 | 9 | 2 |
| 20.49677 | 22.62204 | 21.23702 | 21.45194 | 10 | 2 |
| 19.94354 | 21.13798 | 37.81678 | 26.29943 | 11 | 2 |
| 19.92028 | 20.97094 | 19.43775 | 20.10966 | 12 | 2 |
| 23.36311 | 21.51101 | 19.43685 | 21.43699 | 13 | 2 |
| 19.45004 | 19.4951 | 21.94365 | 20.29627 | 14 | 2 |
| 20.29468 | 21.14998 | 22.72354 | 21.3894 | 15 | 2 |
| 30.35521 | 22.15575 | 20.2056 | 24.23886 | 16 | 2 |
| 22.74018 | 25.55368 | 27.37431 | 25.22273 | 17 | 2 |
| 20.17698 | 20.18493 | 20.04913 | 20.13701 | 18 | 2 |
| 22.98292 | 42.27164 | 19.79756 | 28.3507 | 19 | 3 |
| 19.81204 | 21.09542 | 19.89756 | 20.26834 | 20 | 3 |
| 22.50603 | 19.64 | 22.92143 | 21.68915 | 21 | 3 |

| | | | | | |
|---|---|---|---|---|---|
| 23.83106 | 21.46401 | 20.04193 | 21.779 | 22 | 3 |
| 20.2137 | 19.51319 | 43.06104 | 27.59598 | 23 | 3 |
| 19.42765 | 27.91123 | 28.39492 | 25.2446 | 24 | 3 |
| 22.98292 | 35.54337 | 21.60663 | 26.71097 | 25 | 3 |
| 20.74697 | 21.46401 | 19.92028 | 20.71042 | 26 | 3 |
| 22.72354 | 19.65536 | 40.43176 | 27.60355 | 27 | 3 |
| 19.50642 | 21.09542 | 20.35675 | 20.31953 | 28 | 3 |

21.68915364
21.77899941
27.59597669
25.2445997
26.71097447
20.71041753
27.60355338
20.31952981
26.29943203
20.10965741
21.43698966
20.29626574
21.38939961
24.23885527
25.22272575
20.13701332
28.35070282
20.26834135
20.05237351
20.17588514
23.16080416

19.72155506
26.56065187
21.22999374
22.83065668
26.05685125
28.96384577
21.45194154

Report

PTH

| 分组 | Mean | Std. Deviation |
|---|---|---|
| 1 | 23.95665C | 3.1496052155310 |
| 2 | 22.774938 | 3.0156068618596 |
| 3 | 23.020455 | 3.1724880427210 |
| Total | 23.200255 | 3.0342274119352 |

Tests of Normality

| | 分组 | Kolmogorov-Smirnova | | | Shapiro-Wilk | | |
|---|---|---|---|---|---|---|---|
| | | Statistic | df | Sig. | Statistic | df | Sig. |
| PTH | 1 | 0.255 | 8 | 0.133 | 0.845 | 8 | 0.085 |
| | 2 | 0.271 | 10 | 0.035 | 0.842 | 10 | 0.047 |
| | 3 | 0.189 | 10 | .200* | 0.894 | 10 | 0.186 |

* This is a lower bound of the true significance.

a Lilliefors Significance Correction

**Hypothesis Test Summary**

| | Null Hypothesis | Test | Sig. | Decision |
|---|---|---|---|---|
| 1 | The distribution of PTH is the same across categories of 分组. | Independent-Samples Kruskal-Wallis Test | .438 | Retain the null hypothesis. |

Asymptotic significances are displayed. The significance level is .05.

Multiple Comparisons

Dependent Variable: PTH

Tukey HSD

| (I) 分组 | (J) 分组 | Mean Diff | Std. Errc | Sig. | 95% Confidence Interval | |
|---|---|---|---|---|---|---|
| | | | | | Lower Bou | Upper Bound |
| 1 | 2 | 1.1817122 | 1.4753998 | 0.706 | -2.49326 | 4.856683 |
| | 3 | .9361947C | 1.4753998 | 0.803 | -2.73878 | 4.611165 |
| 2 | 1 | -1.18171 | 1.4753998 | 0.706 | -4.85668 | 2.493258 |
| | 3 | -0.24552 | 1.3910202 | 0.983 | -3.71031 | 3.219278 |
| 3 | 1 | -0.93619 | 1.4753998 | 0.803 | -4.61116 | 2.738776 |
| | 2 | .24551757 | 1.3910202 | 0.983 | -3.21928 | 3.710313 |

| | 1 | 15 | 29 | 43 |
|---|---|---|---|---|
| 1 | 234 | 256 | 286 | 387 |
| 1 | 225 | 249 | 277 | 364 |
| 1 | 237 | 284 | 368 | 413 |
| 1 | 224 | 296 | 387 | 402 |
| 1 | 231 | 283 | 386 | 414 |
| 1 | 229 | 269 | 364 | 373 |
| 1 | 228 | 231 | 255 | 296 |
| 1 | 232 | 264 | 281 | 313 |
| 1 | 237 | 255 | 274 | 304 |
| 1 | 230 | 287 | 322 | 383 |
| 1 | 226 | 247 | 267 | 309 |
| 1 | 229 | 276 | 344 | 383 |
| 2 | 234 | 244 | 273 | 273 |
| 2 | 226 | 230 | 228 | 240 |
| 2 | 228 | 266 | 300 | 326 |
| 2 | 231 | 247 | 218 | 308 |
| 2 | 238 | 263 | 276 | 314 |
| 2 | 228 | 250 | 228 | 237 |
| 2 | 234 | 278 | 332 | 224 |
| 2 | 229 | 262 | 258 | 332 |
| 2 | 232 | 266 | 295 | 276 |
| 2 | 233 | 293 | 296 | 296 |
| 2 | 221 | 237 | 240 | 275 |
| 2 | 230 | 246 | 224 | 266 |
| 2 | 235 | 252 | 222 | 228 |
| 2 | 234 | 287 | 308 | 258 |
| 2 | 223 | 267 | 288 | 295 |
| 2 | 235 | 266 | 275 | 308 |
| 2 | 227 | 278 | 326 | 267 |
| 2 | 226 | 266 | 267 | 270 |
| 3 | 233 | 292 | 308 | 267 |
| 3 | 231 | 263 | 270 | 262 |
| 3 | 224 | 271 | 266 | 291 |
| 3 | 235 | 288 | 314 | 321 |
| 3 | 229 | 244 | 237 | 317 |
| 3 | 221 | 284 | 299 | 287 |
| 3 | 232 | 267 | 260 | 299 |
| 3 | 228 | 259 | 291 | 272 |
| 3 | 231 | 247 | 259 | 260 |
| 3 | 230 | 263 | 252 | 262 |
| 3 | 237 | 255 | 240 | 314 |
| 3 | 224 | 272 | 262 | 265 |
| 3 | 233 | 279 | 314 | 261 |
| 3 | 236 | 283 | 291 | 240 |
| 3 | 229 | 246 | 265 | 291 |
| 3 | 230 | 255 | 267 | 299 |
| 3 | 239 | 270 | | |
| 3 | 222 | 275 | | |

| | | | | | |
|---|---|---|---|---|---|
| 1 | 235 | 255.5 | 287 | 387.5 | 431 |
| 1 | 225.5 | 249 | 277 | 364 | 390.5 |
| 1 | 237.5 | 284.5 | 368.5 | 412.5 | 442.5 |
| 1 | 223 | 295.5 | 387.5 | 402.5 | 433 |
| 1 | 230.5 | 283.5 | 386.5 | 413.5 | 449.5 |
| 1 | 231 | 269.5 | 365 | 372.5 | 403.5 |
| 1 | 228 | 230.5 | 254.5 | 296.5 | 349.5 |
| 1 | 231 | 265 | 281.5 | 313 | 365 |
| 1 | 237.5 | 254.5 | 273.5 | 304 | 358.5 |
| 1 | 230 | 286.5 | 321.5 | 383 | 429.5 |
| 1 | 226.5 | 247.5 | 266.5 | 309.5 | 353.5 |
| 1 | 229.5 | 276.5 | 344.5 | 383.5 | 394 |
| 2 | 233.5 | 244 | 273.5 | 272.5 | 291 |
| 2 | 226.5 | 230.5 | 228.5 | 240.5 | 274 |
| 2 | 227.5 | 265.5 | 300.5 | 325.5 | 350.5 |
| 2 | 230.5 | 247.5 | 218.5 | 309 | 335 |
| 2 | 237.5 | 263.5 | 276 | 314 | 315 |
| 2 | 229 | 249.5 | 229 | 237 | 259.5 |
| 2 | 233.5 | 277.5 | 331.5 | 224.5 | 254.5 |
| 2 | 229.5 | 262.5 | 258.5 | 331 | 334.5 |
| 2 | 231.5 | 265.5 | 295 | 275.5 | 310.5 |
| 2 | 232.5 | 294 | 296 | 296.5 | 310 |
| 2 | 221.5 | 237.5 | 240.5 | 276.5 | 326.5 |
| 2 | 230 | 246.5 | 223.5 | 265 | 291 |
| 2 | 235.5 | 251.5 | 223 | 229 | 271.5 |
| 2 | 233.5 | 288 | 307 | 258.5 | 265 |
| 2 | 223.5 | 266.5 | 289 | 295 | 304.5 |
| 2 | 235.5 | 266 | 274 | 308 | 344 |
| 2 | 226 | 278.5 | 326 | 267 | 321.5 |
| 2 | 225 | 266.5 | 267.5 | 270.5 | 280 |
| 3 | 233.5 | 292.5 | 308.5 | 267.5 | 299.5 |
| 3 | 231.5 | 263 | 270.5 | 262.5 | 325 |
| 3 | 224.5 | 272 | 266 | 291 | 330 |
| 3 | 234 | 289 | 314.5 | 321 | 383.5 |
| 3 | 229.5 | 244.5 | 237.5 | 317.5 | 366 |
| 3 | 222 | 284 | 299.5 | 287.5 | 345.5 |
| 3 | 231.5 | 266.5 | 259.5 | 299.5 | 361.5 |
| 3 | 228.5 | 263.5 | 291.5 | 272.5 | 341.5 |
| 3 | 230.5 | 246.5 | 259 | 260 | 296 |
| 3 | 230 | 263.5 | 252.5 | 261.5 | 329 |
| 3 | 237.5 | 255 | 241 | 314 | 341.5 |
| 3 | 223.5 | 272.5 | 262.5 | 265.5 | 311 |
| 3 | 233.5 | 279.5 | 363.5 | 261 | 321 |
| 3 | 235.5 | 282.5 | 291 | 240 | 259.5 |

| | | | | | |
|---|---|---|---|---|---|
| 3 | 229.5 | 246.5 | 265.5 | 291.5 | 324 |
| 3 | 231.5 | 255.5 | 267.5 | 299.5 | 341 |
| 3 | 239.5 | 269.5 | 278.125 | 282 | 329.71875 |

| | | | | | |
|---|---|---|---|---|---|
| 57 | | | 1 | | |
| 431 | 197 | 1 | 236 | 234 | 235 |
| 390 | 165 | 1 | 226 | 225 | 225.5 |
| 443 | 206 | 1 | 238 | 237 | 237.5 |
| 434 | 210 | 1 | 222 | 224 | 223 |
| 449 | 218 | 1 | 230 | 231 | 230.5 |
| 404 | 175 | 1 | 233 | 229 | 231 |
| 349 | 121 | 1 | 228 | 228 | 228 |
| 365 | 133 | 1 | 230 | 232 | 231 |
| 358 | 121 | 1 | 238 | 237 | 237.5 |
| 429 | 199 | 1 | 230 | 230 | 230 |
| 354 | 128 | 1 | 227 | 226 | 226.5 |
| 393 | 164 | 1 | 230 | 229 | 229.5 |
| 292 | 58 | 2 | 233 | 234 | 233.5 |
| 273 | 47 | 2 | 227 | 226 | 226.5 |
| 351 | 123 | 2 | 227 | 228 | 227.5 |
| 335 | 104 | 2 | 230 | 231 | 230.5 |
| 315 | 77 | 2 | 237 | 238 | 237.5 |
| 259 | 31 | 2 | 230 | 228 | 229 |
| 254 | 20 | 2 | 233 | 234 | 233.5 |
| 334 | 105 | 2 | 230 | 229 | 229.5 |
| 311 | 79 | 2 | 231 | 232 | 231.5 |
| 310 | 77 | 2 | 232 | 233 | 232.5 |
| 327 | 106 | 2 | 222 | 221 | 221.5 |
| 292 | 62 | 2 | 230 | 230 | 230 |
| 272 | 37 | 2 | 236 | 235 | 235.5 |
| 265 | 31 | 2 | 233 | 234 | 233.5 |
| 304 | 81 | 2 | 224 | 223 | 223.5 |
| 343 | 108 | 2 | 236 | 235 | 235.5 |
| 322 | 95 | 2 | 225 | 227 | 226 |
| 280 | 54 | 2 | 224 | 226 | 225 |
| 299 | 66 | 3 | 234 | 233 | 233.5 |
| 325 | 94 | 3 | 232 | 231 | 231.5 |
| 330 | 106 | 3 | 225 | 224 | 224.5 |
| 383 | 148 | 3 | 233 | 235 | 234 |
| 367 | 138 | 3 | 230 | 229 | 229.5 |
| 346 | 125 | 3 | 223 | 221 | 222 |
| 362 | 130 | 3 | 231 | 232 | 231.5 |
| 342 | 114 | 3 | 229 | 228 | 228.5 |
| 297 | 66 | 3 | 230 | 231 | 230.5 |
| 328 | 98 | 3 | 230 | 230 | 230 |
| 342 | 105 | 3 | 238 | 237 | 237.5 |
| 312 | 88 | 3 | 223 | 224 | 223.5 |
| 322 | 89 | 3 | 234 | 233 | 233.5 |
| 259 | 23 | 3 | 235 | 236 | 235.5 |
| 323 | 94 | 3 | 230 | 229 | 229.5 |
| 342 | 112 | 3 | 233 | 230 | 231.5 |
| | | 3 | 240 | 239 | 239.5 |
| | | 3 | 221 | 222 | 221.5 |

| | |
|---|---|
| 462.5 | 492 |
| 413 | 443.5 |
| 464 | 489 |
| 456.5 | 481.5 |
| 472.5 | 500.5 |
| 453.5 | 490 |
| 381 | 412.5 |
| 400.5 | 441.5 |
| 384 | 432.5 |
| 459.5 | 482.5 |
| 392 | 452 |
| 440.5 | 487.5 |
| 341 | 369.5 |
| 299.5 | 341 |
| 370.5 | 392.5 |
| 362 | 385.5 |
| 333 | 362.5 |
| 294 | 337.5 |
| 286.5 | 342 |
| 361.5 | 386.5 |
| 352 | 393 |
| 344.5 | 381.5 |
| 353.5 | 395.5 |
| 320.5 | 372 |
| 336.5 | 374.5 |
| 301.5 | 342.5 |
| 346.5 | 382.5 |
| 378 | 400.5 |
| 366.5 | 399 |
| 356 | 388.5 |
| 350.5 | 399 |
| 369.5 | 412.5 |
| 377.5 | 404 |
| 412 | 454.5 |
| 392 | 422.5 |
| 377.5 | 409.5 |
| 397 | 432.5 |
| 389 | 420 |
| 345 | 389.5 |
| 368 | 401.5 |
| 382 | 423 |
| 359 | 404.5 |
| 371.5 | 435 |
| 312.5 | 345 |

| | |
|---|---|
| 372.5 | 405.5 |
| 382.5 | 423.5 |
| 372.375 | 411.375 |

| | | | | | |
|---|---|---|---|---|---|
| 15 | | | 29 | | |
| 255 | 256 | 255.5 | 288 | 286 | 287 |
| 249 | 249 | 249 | 277 | 277 | 277 |
| 285 | 284 | 284.5 | 369 | 368 | 368.5 |
| 295 | 296 | 295.5 | 388 | 387 | 387.5 |
| 284 | 283 | 283.5 | 387 | 386 | 386.5 |
| 270 | 269 | 269.5 | 366 | 364 | 365 |
| 230 | 231 | 230.5 | 254 | 255 | 254.5 |
| 266 | 264 | 265 | 282 | 281 | 281.5 |
| 254 | 255 | 254.5 | 273 | 274 | 273.5 |
| 286 | 287 | 286.5 | 321 | 322 | 321.5 |
| 248 | 247 | 247.5 | 266 | 267 | 266.5 |
| 277 | 276 | 276.5 | 345 | 344 | 344.5 |
| 244 | 244 | 244 | 274 | 273 | 273.5 |
| 231 | 230 | 230.5 | 229 | 228 | 228.5 |
| 265 | 266 | 265.5 | 301 | 300 | 300.5 |
| 248 | 247 | 247.5 | 219 | 218 | 218.5 |
| 264 | 263 | 263.5 | 276 | 276 | 276 |
| 249 | 250 | 249.5 | 230 | 228 | 229 |
| 277 | 278 | 277.5 | 331 | 332 | 331.5 |
| 263 | 262 | 262.5 | 259 | 258 | 258.5 |
| 265 | 266 | 265.5 | 295 | 295 | 295 |
| 295 | 293 | 294 | 296 | 296 | 296 |
| 238 | 237 | 237.5 | 241 | 240 | 240.5 |
| 247 | 246 | 246.5 | 223 | 224 | 223.5 |
| 251 | 252 | 251.5 | 224 | 222 | 223 |
| 289 | 287 | 288 | 306 | 308 | 307 |
| 266 | 267 | 266.5 | 290 | 288 | 289 |
| 266 | 266 | 266 | 273 | 275 | 274 |
| 279 | 278 | 278.5 | 326 | 326 | 326 |
| 267 | 266 | 266.5 | 268 | 267 | 267.5 |
| 293 | 292 | 292.5 | 309 | 308 | 308.5 |
| 263 | 263 | 263 | 271 | 270 | 270.5 |
| 273 | 271 | 272 | 266 | 266 | 266 |
| 290 | 288 | 289 | 315 | 314 | 314.5 |
| 245 | 244 | 244.5 | 238 | 237 | 237.5 |
| 284 | 284 | 284 | 300 | 299 | 299.5 |
| 266 | 267 | 266.5 | 259 | 260 | 259.5 |
| 268 | 259 | 263.5 | 292 | 291 | 291.5 |
| 246 | 247 | 246.5 | 259 | 259 | 259 |
| 264 | 263 | 263.5 | 253 | 252 | 252.5 |
| 255 | 255 | 255 | 242 | 240 | 241 |
| 273 | 272 | 272.5 | 263 | 262 | 262.5 |
| 280 | 279 | 279.5 | 413 | 314 | 363.5 |
| 282 | 283 | 282.5 | 291 | 291 | 291 |
| 247 | 246 | 246.5 | 266 | 265 | 265.5 |
| 256 | 255 | 255.5 | 268 | 267 | 267.5 |
| 269 | 270 | 269.5 | | | |
| 277 | 275 | 276 | | | |

| 43 | | | 57 | | | 71 | |
|---|---|---|---|---|---|---|---|
| 388 | 387 | 387.5 | 431 | 431 | 431 | 462 | 463 |
| 364 | 364 | 364 | 391 | 390 | 390.5 | 411 | 415 |
| 412 | 413 | 412.5 | 442 | 443 | 442.5 | 463 | 465 |
| 403 | 402 | 402.5 | 432 | 434 | 433 | 458 | 455 |
| 413 | 414 | 413.5 | 450 | 449 | 449.5 | 472 | 473 |
| 372 | 373 | 372.5 | 403 | 404 | 403.5 | 455 | 452 |
| 297 | 296 | 296.5 | 350 | 349 | 349.5 | 380 | 382 |
| 313 | 313 | 313 | 365 | 365 | 365 | 400 | 401 |
| 304 | 304 | 304 | 359 | 358 | 358.5 | 383 | 385 |
| 383 | 383 | 383 | 430 | 429 | 429.5 | 459 | 460 |
| 310 | 309 | 309.5 | 353 | 354 | 353.5 | 392 | 392 |
| 384 | 383 | 383.5 | 395 | 393 | 394 | 440 | 441 |
| 272 | 273 | 272.5 | 290 | 292 | 291 | 340 | 342 |
| 241 | 240 | 240.5 | 275 | 273 | 274 | 299 | 300 |
| 325 | 326 | 325.5 | 350 | 351 | 350.5 | 370 | 371 |
| 310 | 308 | 309 | 335 | 335 | 335 | 361 | 363 |
| 314 | 314 | 314 | 315 | 315 | 315 | 332 | 334 |
| 237 | 237 | 237 | 260 | 259 | 259.5 | 294 | 294 |
| 225 | 224 | 224.5 | 255 | 254 | 254.5 | 287 | 286 |
| 330 | 332 | 331 | 335 | 334 | 334.5 | 361 | 362 |
| 275 | 276 | 275.5 | 310 | 311 | 310.5 | 351 | 353 |
| 297 | 296 | 296.5 | 310 | 310 | 310 | 344 | 345 |
| 278 | 275 | 276.5 | 326 | 327 | 326.5 | 352 | 355 |
| 264 | 266 | 265 | 290 | 292 | 291 | 320 | 321 |
| 230 | 228 | 229 | 271 | 272 | 271.5 | 337 | 336 |
| 259 | 258 | 258.5 | 265 | 265 | 265 | 301 | 302 |
| 295 | 295 | 295 | 305 | 304 | 304.5 | 346 | 347 |
| 308 | 308 | 308 | 345 | 343 | 344 | 377 | 379 |
| 267 | 267 | 267 | 321 | 322 | 321.5 | 366 | 367 |
| 271 | 270 | 270.5 | 280 | 280 | 280 | 357 | 355 |
| 268 | 267 | 267.5 | 300 | 299 | 299.5 | 350 | 351 |
| 263 | 262 | 262.5 | 325 | 325 | 325 | 369 | 370 |
| 291 | 291 | 291 | 330 | 330 | 330 | 377 | 378 |
| 321 | 321 | 321 | 384 | 383 | 383.5 | 411 | 413 |
| 318 | 317 | 317.5 | 365 | 367 | 366 | 392 | 392 |
| 288 | 287 | 287.5 | 345 | 346 | 345.5 | 377 | 378 |
| 300 | 299 | 299.5 | 361 | 362 | 361.5 | 398 | 396 |
| 273 | 272 | 272.5 | 341 | 342 | 341.5 | 388 | 390 |
| 260 | 260 | 260 | 295 | 297 | 296 | 344 | 346 |
| 261 | 262 | 261.5 | 330 | 328 | 329 | 367 | 369 |
| 314 | 314 | 314 | 341 | 342 | 341.5 | 381 | 383 |
| 266 | 265 | 265.5 | 310 | 312 | 311 | 358 | 360 |
| 261 | 261 | 261 | 320 | 322 | 321 | 371 | 372 |
| 240 | 240 | 240 | 260 | 259 | 259.5 | 312 | 313 |
| 292 | 291 | 291.5 | 325 | 323 | 324 | 372 | 373 |
| 300 | 299 | 299.5 | 340 | 342 | 341 | 382 | 383 |

| | | | | | | | |
|---|---|---|---|---|---|---|---|
| | 85 | | | | Body Weight at | Preoperative Bo | Body Weight at |
| 462.5 | 492 | 492 | 492 | 1 | 236 | 255 | 288 |
| 413 | 442 | 445 | 443.5 | 1 | 226 | 249 | 277 |
| 464 | 488 | 490 | 489 | 1 | 238 | 285 | 369 |
| 456.5 | 483 | 480 | 481.5 | 1 | 222 | 295 | 388 |
| 472.5 | 501 | 500 | 500.5 | 1 | 230 | 284 | 387 |
| 453.5 | 491 | 489 | 490 | 1 | 233 | 270 | 366 |
| 381 | 413 | 412 | 412.5 | 1 | 228 | 230 | 254 |
| 400.5 | 440 | 443 | 441.5 | 1 | 230 | 266 | 282 |
| 384 | 432 | 433 | 432.5 | 1 | 238 | 254 | 273 |
| 459.5 | 482 | 483 | 482.5 | 1 | 230 | 286 | 321 |
| 392 | 452 | 452 | 452 | 1 | 227 | 248 | 266 |
| 440.5 | 489 | 486 | 487.5 | 1 | 230 | 277 | 345 |
| 341 | 369 | 370 | 369.5 | 2 | 233 | 244 | 274 |
| 299.5 | 340 | 342 | 341 | 2 | 227 | 231 | 229 |
| 370.5 | 392 | 393 | 392.5 | 2 | 227 | 265 | 301 |
| 362 | 386 | 385 | 385.5 | 2 | 230 | 248 | 219 |
| 333 | 362 | 363 | 362.5 | 2 | 237 | 264 | 276 |
| 294 | 337 | 338 | 337.5 | 2 | 230 | 249 | 230 |
| 286.5 | 341 | 343 | 342 | 2 | 233 | 277 | 331 |
| 361.5 | 388 | 385 | 386.5 | 2 | 230 | 263 | 259 |
| 352 | 392 | 394 | 393 | 2 | 231 | 265 | 295 |
| 344.5 | 381 | 382 | 381.5 | 2 | 232 | 295 | 296 |
| 353.5 | 395 | 396 | 395.5 | 2 | 222 | 238 | 241 |
| 320.5 | 371 | 373 | 372 | 2 | 230 | 247 | 223 |
| 336.5 | 374 | 375 | 374.5 | 2 | 236 | 251 | 224 |
| 301.5 | 341 | 344 | 342.5 | 2 | 233 | 289 | 306 |
| 346.5 | 382 | 383 | 382.5 | 2 | 224 | 266 | 290 |
| 378 | 400 | 401 | 400.5 | 2 | 236 | 266 | 273 |
| 366.5 | 398 | 400 | 399 | 2 | 225 | 279 | 326 |
| 356 | 387 | 390 | 388.5 | 2 | 224 | 267 | 268 |
| 350.5 | 399 | 399 | 399 | 3 | 234 | 293 | 309 |
| 369.5 | 412 | 413 | 412.5 | 3 | 232 | 263 | 271 |
| 377.5 | 406 | 402 | 404 | 3 | 225 | 273 | 266 |
| 412 | 454 | 455 | 454.5 | 3 | 233 | 290 | 315 |
| 392 | 423 | 422 | 422.5 | 3 | 230 | 245 | 238 |
| 377.5 | 409 | 410 | 409.5 | 3 | 223 | 284 | 300 |
| 397 | 433 | 432 | 432.5 | 3 | 231 | 266 | 259 |
| 389 | 419 | 421 | 420 | 3 | 229 | 268 | 292 |
| 345 | 389 | 390 | 389.5 | 3 | 230 | 246 | 259 |
| 368 | 400 | 403 | 401.5 | 3 | 230 | 264 | 253 |
| 382 | 421 | 425 | 423 | 3 | 238 | 255 | 242 |
| 359 | 404 | 405 | 404.5 | 3 | 223 | 273 | 263 |
| 371.5 | 434 | 436 | 435 | 3 | 234 | 280 | 413 |
| 312.5 | 344 | 346 | 345 | 3 | 235 | 282 | 291 |
| 372.5 | 405 | 406 | 405.5 | 3 | 230 | 247 | 266 |
| 382.5 | 423 | 424 | 423.5 | 3 | 233 | 256 | 268 |
| | | | | 3 | 240 | 269 | |
| | | | | 3 | 221 | 277 | |

| Body Weight at | Body Weight a | Body Wei | Body Weight at Postoperative Week 10 |
|---|---|---|---|
| 388 | 431 | 462.5 | 492 |
| 364 | 391 | 413 | 443.5 |
| 412 | 442 | 464 | 489 |
| 403 | 432 | 456.5 | 481.5 |
| 413 | 450 | 472.5 | 500.5 |
| 372 | 403 | 453.5 | 490 |
| 297 | 350 | 381 | 412.5 |
| 313 | 365 | 400.5 | 441.5 |
| 304 | 359 | 384 | 432.5 |
| 383 | 430 | 459.5 | 482.5 |
| 310 | 353 | 392 | 452 |
| 384 | 395 | 440.5 | 487.5 |
| 272 | 290 | 341 | 369.5 |
| 241 | 275 | 299.5 | 341 |
| 325 | 350 | 370.5 | 392.5 |
| 310 | 335 | 362 | 385.5 |
| 314 | 315 | 333 | 362.5 |
| 237 | 260 | 294 | 337.5 |
| 225 | 255 | 286.5 | 342 |
| 330 | 335 | 361.5 | 386.5 |
| 275 | 310 | 352 | 393 |
| 297 | 310 | 344.5 | 381.5 |
| 278 | 326 | 353.5 | 395.5 |
| 264 | 290 | 320.5 | 372 |
| 230 | 271 | 336.5 | 374.5 |
| 259 | 265 | 301.5 | 342.5 |
| 295 | 305 | 346.5 | 382.5 |
| 308 | 345 | 378 | 400.5 |
| 267 | 321 | 366.5 | 399 |
| 271 | 280 | 356 | 388.5 |
| 268 | 300 | 350.5 | 399 |
| 263 | 325 | 369.5 | 412.5 |
| 291 | 330 | 377.5 | 404 |
| 321 | 384 | 412 | 454.5 |
| 318 | 365 | 392 | 422.5 |
| 288 | 345 | 377.5 | 409.5 |
| 300 | 361 | 397 | 432.5 |
| 273 | 341 | 389 | 420 |
| 260 | 295 | 345 | 389.5 |
| 261 | 330 | 368 | 401.5 |
| 314 | 341 | 382 | 423 |
| 266 | 310 | 359 | 404.5 |
| 261 | 320 | 371.5 | 435 |
| 240 | 260 | 312.5 | 345 |
| 292 | 325 | 372.5 | 405.5 |
| 300 | 340 | 382.5 | 423.5 |

Normality Test

| | Group | Kolmogorov-Smirnov(K)a | | | Shapiro-Wilk | | |
|---|---|---|---|---|---|---|---|
| | | Statisti‹df | | Sig. | Statistic df | | Sig. |
| Day1 | 1 | 0.199 | 12 | .200* | 0.948 | 12 | 0.607 |
| | 2 | 0.11 | 18 | .200* | 0.977 | 18 | 0.91 |
| | 3 | 0.166 | 16 | .200* | 0.95 | 16 | 0.497 |
| Day15 | 1 | 0.141 | 12 | .200* | 0.963 | 12 | 0.832 |
| | 2 | 0.155 | 18 | .200* | 0.968 | 18 | 0.759 |
| | 3 | 0.1 | 16 | .200* | 0.952 | 16 | 0.52 |
| Day29 | 1 | 0.231 | 12 | 0.078 | 0.879 | 12 | 0.085 |
| | 2 | 0.148 | 18 | .200* | 0.936 | 18 | 0.252 |
| | 3 | 0.219 | 16 | 0.039 | 0.896 | 16 | 0.07 |
| Day43 | 1 | 0.2 | 12 | .200* | 0.866 | 12 | 0.059 |
| | 2 | 0.124 | 18 | .200* | 0.959 | 18 | 0.588 |
| | 3 | 0.164 | 16 | .200* | 0.938 | 16 | 0.329 |
| Day57 | 1 | 0.205 | 12 | 0.177 | 0.902 | 12 | 0.166 |
| | 2 | 0.103 | 18 | .200* | 0.955 | 18 | 0.515 |
| | 3 | 0.135 | 16 | .200* | 0.97 | 16 | 0.839 |
| Day71 | 1 | 0.235 | 12 | 0.066 | 0.858 | 12 | 0.047 |
| | 2 | 0.139 | 18 | .200* | 0.92 | 18 | 0.132 |
| | 3 | 0.175 | 16 | .200* | 0.945 | 16 | 0.409 |
| Day85 | 1 | 0.274 | 12 | 0.013 | 0.876 | 12 | 0.078 |
| | 2 | 0.179 | 18 | 0.132 | 0.88 | 18 | 0.026 |
| | 3 | 0.178 | 16 | 0.188 | 0.909 | 16 | 0.113 |

* This is a lower bound of the true significance.
a Lilliefors Significance Correction

ANOVA

| | | Sum of Sc | df | Mean Squa | F | Sig. |
|---|---|---|---|---|---|---|
| Day1 | Between ( | 1.05 | 2 | 0.525 | 0.024 | 0.977 |
| | Within Gı | 993.319 | 45 | 22.074 | | |
| | Total | 994.37 | 47 | | | |
| Day15 | Between ( | 441.701 | 2 | 220.851 | 0.781 | 0.464 |
| | Within Gı | 12722.28 | 45 | 282.717 | | |
| | Total | 13163.98 | 47 | | | |
| Day29 | Between ( | 17782.49 | 2 | 8891.244 | 5.84 | 0.006 |
| | Within Gı | 65469.38 | 43 | 1522.544 | | |
| | Total | 83251.87 | 45 | | | |
| Day43 | Between ( | 60101.08 | 2 | 30050.54 | 26.938 | 0 |
| | Within Gı | 47968.4 | 43 | 1115.544 | | |
| | Total | 108069.5 | 45 | | | |
| Day57 | Between ( | 70348.48 | 2 | 35174.241 | 34.583 | 0 |
| | Within Gı | 43735.39 | 43 | 1017.102 | | |
| | Total | 114083.9 | 45 | | | |

Multiple Comparisons
Tukey HSD

| Dependent | (I) Grouı | (J) Grouı | Mean Diffı | Std. Erro | Sig. | 95% Confidence Interval |
|---|---|---|---|---|---|---|

| | | | | | | | |
|---|---|---|---|---|---|---|---|
| | | | | | | Lower Boun | Upper Bound |
| Day1 | 1 | 2 | 0.30556 | 1.75094 | 0.983 | -3.9381 | 4.5492 |
| | | 3 | 0 | 1.75094 | 1 | -4.2436 | 4.2436 |
| | 2 | 1 | -0.30556 | 1.75094 | 0.983 | -4.5492 | 3.9381 |
| | | 3 | -0.30556 | 1.56609 | 0.979 | -4.1012 | 3.49 |
| | 3 | 1 | 0 | 1.75094 | 1 | -4.2436 | 4.2436 |
| | | 2 | 0.30556 | 1.56609 | 0.979 | -3.49 | 4.1012 |
| Day15 | 1 | 2 | 5.33333 | 6.26628 | 0.673 | -9.8537 | 20.5204 |
| | | 3 | -1.38889 | 6.26628 | 0.973 | -16.5759 | 13.7982 |
| | 2 | 1 | -5.33333 | 6.26628 | 0.673 | -20.5204 | 9.8537 |
| | | 3 | -6.72222 | 5.60473 | 0.46 | -20.3059 | 6.8615 |
| | 3 | 1 | 1.38889 | 6.26628 | 0.973 | -13.7982 | 16.5759 |
| | | 2 | 6.72222 | 5.60473 | 0.46 | -6.8615 | 20.3059 |
| Day29 | 1 | 2 | 47.93056* | 14.54182 | 0.005 | 12.6312 | 83.2299 |
| | | 3 | 39.66667* | 14.90093 | 0.029 | 3.4956 | 75.8378 |
| | 2 | 1 | -47.93056* | 14.54182 | 0.005 | -83.2299 | -12.6312 |
| | | 3 | -8.26389 | 13.40689 | 0.812 | -40.8083 | 24.2805 |
| | 3 | 1 | -39.66667* | 14.90093 | 0.029 | -75.8378 | -3.4956 |
| | | 2 | 8.26389 | 13.40689 | 0.812 | -24.2805 | 40.8083 |
| Day43 | 1 | 2 | 84.30556* | 12.44736 | 0 | 54.0903 | 114.5208 |
| | | 3 | 79.83333* | 12.75475 | 0 | 48.872 | 110.7947 |
| | 2 | 1 | -84.30556* | 12.44736 | 0 | -114.5208 | -54.0903 |
| | | 3 | -4.47222 | 11.4759 | 0.92 | -32.3293 | 23.3848 |
| | 3 | 1 | -79.83333* | 12.75475 | 0 | -110.7947 | -48.872 |
| | | 2 | 4.47222 | 11.4759 | 0.92 | -23.3848 | 32.3293 |
| Day57 | 1 | 2 | 97.86111* | 11.88546 | 0 | 69.0099 | 126.7124 |
| | | 3 | 70.28125* | 12.17897 | 0 | 40.7175 | 99.845 |
| | 2 | 1 | -97.86111* | 11.88546 | 0 | -126.7124 | -69.0099 |
| | | 3 | -27.57986* | 10.95785 | 0.041 | -54.1794 | -0.9803 |
| | 3 | 1 | -70.28125* | 12.17897 | 0 | -99.845 | -40.7175 |
| | | 2 | 27.57986* | 10.95785 | 0.041 | 0.9803 | 54.1794 |

* The mean difference is significant at the 0.05 level.

Multiple Comparisons

Games-Howell(A)

| Dependen | (I) Grou | (J) Grou | Mean Diff | Std. Erro | Sig. | 95% Confidence Interval | |
|---|---|---|---|---|---|---|---|
| | | | | | | Lower Boun | Upper Bound |
| Day43 | 1 | 2 | 84.30556* | 14.84041 | 0 | 46.5776 | 122.0335 |
| | | 3 | 79.83333* | 14.06061 | 0 | 43.5283 | 116.1384 |
| | 2 | 1 | -84.30556* | 14.84041 | 0 | -122.0335 | -46.5776 |
| | | 3 | -4.47222 | 9.74078 | 0.891 | -28.4444 | 19.4999 |
| | 3 | 1 | -79.83333* | 14.06061 | 0 | -116.1384 | -43.5283 |
| | | 2 | 4.47222 | 9.74078 | 0.891 | -19.4999 | 28.4444 |
| Day57 | 1 | 2 | 97.86111* | 12.80059 | 0 | 65.5192 | 130.2031 |
| | | 3 | 70.28125* | 13.00044 | 0 | 37.4847 | 103.0778 |
| | 2 | 1 | -97.86111* | 12.80059 | 0 | -130.2031 | -65.5192 |
| | | 3 | -27.57986* | 10.2943 | 0.03 | -52.8919 | -2.2678 |
| | 3 | 1 | -70.28125* | 13.00044 | 0 | -103.0778 | -37.4847 |
| | | 2 | 27.57986* | 10.2943 | 0.03 | 2.2678 | 52.8919 |

* The mean difference is significant at the 0.05 level.

Multiple Comparisons

| Dependent | (I) Group | (J) Group | Mean Diff | Std. Error | Sig. | 95% Confidence Interval | |
|---|---|---|---|---|---|---|---|
| | | | | | | Lower Bound | Upper Bound |
| Day71 | Bonferroni | 1 | 2 92.54167* | 10.57816 | 0 | 66.1887 | 118.8946 |
| | | | 3 59.25000* | 10.83939 | 0 | 32.2463 | 86.2537 |
| | | 2 | 1 -92.54167* | 10.57816 | 0 | -118.8946 | -66.1887 |
| | | | 3 -33.29167* | 9.75258 | 0.004 | -57.5879 | -8.9954 |
| | | 3 | 1 -59.25000* | 10.83939 | 0 | -86.2537 | -32.2463 |
| | | | 2 33.29167* | 9.75258 | 0.004 | 8.9954 | 57.5879 |
| | Games-Howell | 1 | 2 92.54167* | 12.0083 | 0 | 62.1644 | 122.919 |
| | | | 3 59.25000* | 11.6125 | 0 | 29.627 | 88.873 |
| | | 2 | 1 -92.54167* | 12.0083 | 0 | -122.919 | -62.1644 |
| | | | 3 -33.29167* | 8.76909 | 0.002 | -54.8442 | -11.7391 |
| | | 3 | 1 -59.25000* | 11.6125 | 0 | -88.873 | -29.627 |
| | | | 2 33.29167* | 8.76909 | 0.002 | 11.7391 | 54.8442 |
| Day85 | Bonferroni | 1 | 2 92.27778* | 9.0829 | 0 | 69.6499 | 114.9057 |
| | | | 3 55.70833* | 9.30721 | 0 | 32.5217 | 78.895 |
| | | 2 | 1 -92.27778* | 9.0829 | 0 | -114.9057 | -69.6499 |
| | | | 3 -36.56944* | 8.37402 | 0 | -57.4313 | -15.7076 |
| | | 3 | 1 -55.70833* | 9.30721 | 0 | -78.895 | -32.5217 |
| | | | 2 36.56944* | 8.37402 | 0 | 15.7076 | 57.4313 |
| | Games-Howell | 1 | 2 92.27778* | 9.74741 | 0 | 67.4909 | 117.0646 |
| | | | 3 55.70833* | 10.28387 | 0 | 29.7986 | 81.618 |
| | | 2 | 1 -92.27778* | 9.74741 | 0 | -117.0646 | -67.4909 |
| | | | 3 -36.56944* | 7.81598 | 0 | -55.829 | -17.3099 |
| | | 3 | 1 -55.70833* | 10.28387 | 0 | -81.618 | -29.7986 |
| | | | 2 36.56944* | 7.81598 | 0 | 17.3099 | 55.829 |

* The mean difference is significant at the 0.05 level.
