# Supplementary material for: Establishment and investigation of a surgical model of hypothyroidism in Wistar rats
Source: PLoS One. 2026 Jan 20;21(1):e0340302. doi: 10.1371/journal.pone.0340302 (PMC12818658; doi:10.1371/journal.pone.0340302)

Experimental Grouping
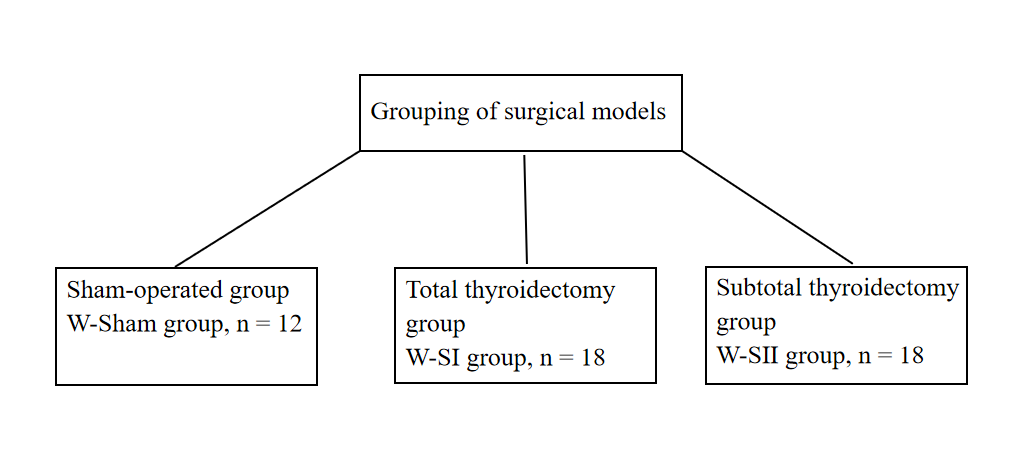


In this experiment, we chose a total of 48 healthy Wistar rats as study subjects and divided them into 3 groups for intervention operations:the sham operation group (W-Sham group, n = 12), the operation group of 36 rats was divided into the total thyroidectomy group (W-SⅠ group, n = 18) and the majority thyroidectomy group (W-SⅡ group, n = 18). The experiment used an electrocoagulation pen hemostat for cutting and hemostasis, which reduced the mortality rate of the rats and improved the postoperative quality of life compared with other experiments, only 2 rats died, and the rest of the rats did not experience any adverse events during the long-term observation.


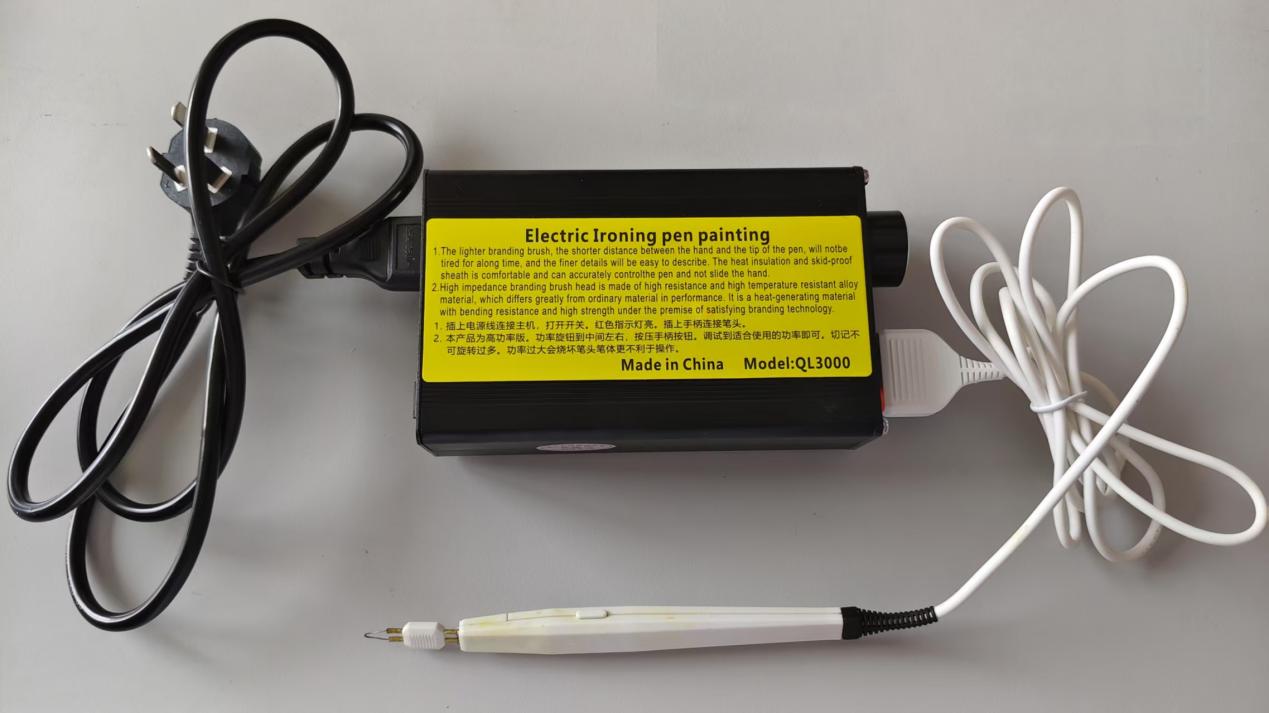

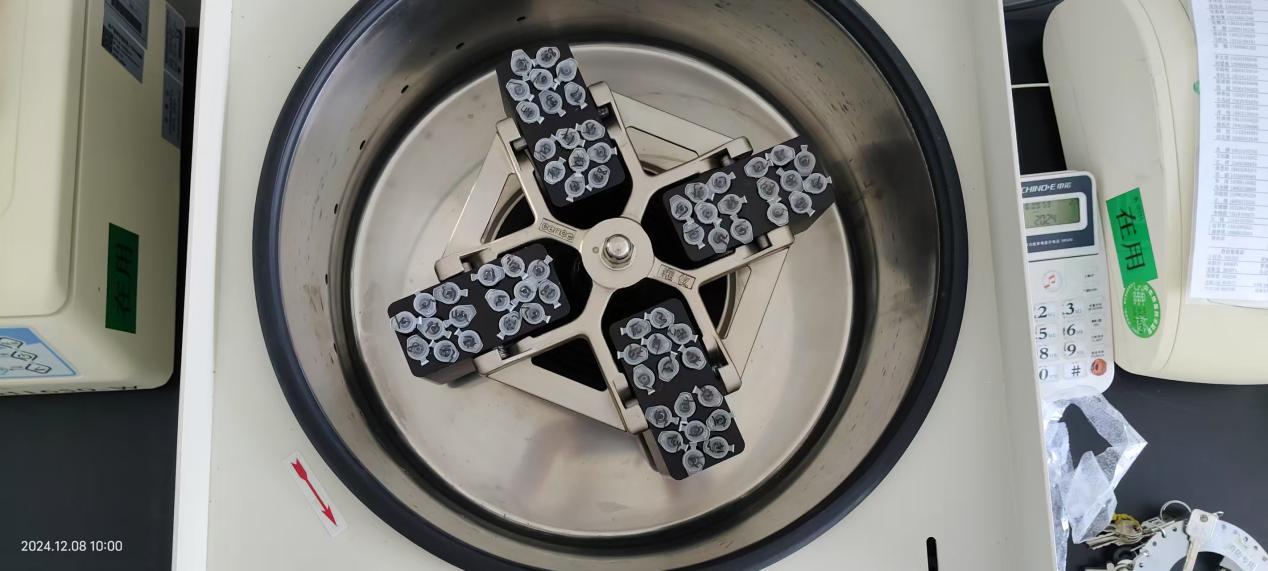

Supplement: S2 File — (DOCX) [file pone.0340302.s003.docx]
